# Visible‐light‐promoted catalyst‐free benzylic C‐H oxidation using molecular oxygen as a green oxidant

**DOI:** 10.1002/smo2.70058

**Published:** 2026-05-17

**Authors:** Jianing Li, Suwen Wang, Huinan Sun, Zhunchao Liu, Xiangmin Tian, Qilei Liu, Qingwei Meng

**Affiliations:** ^1^ Department of Pharmacy School of Chemical Engineering Dalian University of Technology Dalian China; ^2^ Ningbo Institute of Dalian University of Technology Dalian University of Technology Ningbo China

**Keywords:** benzylic C‐H bond, carbonylation, molecular oxygen, visible light

## Abstract

Herein, we report a catalyst‐ and additive‐free strategy for the visible‐light‐promoted carbonylative oxidation of benzylic C‐H bonds employing molecular oxygen as the sole oxidant. This operationally simple protocol enables the conversion of a broad range of aromatic alkanes into valuable ketones, including complex drug derivatives and intermediates under mild conditions using ethyl acetate as a green solvent. Mechanistic studies reveal that the reaction proceeds via a substrate‐oxygen charge‐transfer (CT) complex upon photoexcitation, generating reactive oxygen species (singlet oxygen and superoxide anion) and a key benzylic radical. Interestingly, the carbonyl product can further accelerate the transformation by participating in a separate CT complex with the starting material. To address the scalability limitations, a novel solvent‐free continuous‐flow photoreactor was developed, which demonstrated a significant efficiency enhancement of over 150‐fold compared to batch processes. This work presents a green, practical, and scalable method for benzylic oxidation, underpinned by a detailed mechanistic understanding of the photoinduced CT process.

## Graphical abstract

A green and simple photo‐oxygenation of benzylic C‐H bond with molecular oxygen was developed for the preparation of a series of carbonyl compounds. The straightforward C‐H carbonylation strategy features catalyst‐ and additive‐free reaction conditions, only oxygen as a green oxidant, broad scope and scalable, and the simple‐operation nature.

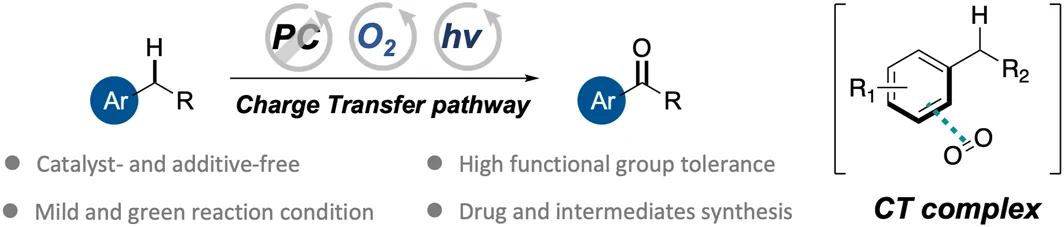

## 1 INTRODUCTION

Carbonyl‐containing compounds display a prominent role in the fields of chemical synthesis, pharmaceuticals, fine chemicals, and materials science.[^1^, ^2^, ^3^, ^4^] The synthesis of carbonyl compounds has been a long‐standing interest in organic chemistry. Among various synthetic approaches, direct oxidation of carbonylation of C‐H bonds is a facile and potential way to obtain the corresponding carbonyl compounds.[^5^, ^6^, ^7^, ^8^] Conventionally, benzylic carbonylation relied on highly transition metals and peroxide as the catalyst as well as the oxidant, which significantly reduced the atom efficiency.[^9^, ^10^, ^11^, ^12^, ^13^, ^14^, ^15^, ^16^] Also, the use of stoichiometric oxidants would add lots of operation risk of process, viewed as a latent environmental hazard.

Molecular oxygen was recognized as an ideal oxidant because of its outstanding characteristics including high atom efficiency, acquirement from abundant air, and generation of by‐product H_2_O, which widely employed in many oxidation protocols.[^17^, ^18^, ^19^, ^20^, ^21^, ^22^, ^23^, ^24^, ^25^, ^26^] Thus, in the past decades, scientists confirmed catalytic systems for carbonylation of benzylic C‐H bonds under mild photocatalyzed conditions with oxygen as oxidant (Scheme 1a). Toward the photooxygenation of benzylic carbonylation, homogeneous photocatalysis has revealed substantial synthetic capabilities through hydrogen atom transfer (HAT) and single‐electron transfer (SET) pathways utilizing a variety of catalysts such as organic dyes and tungsten salts.[^27^, ^28^, ^29^, ^30^, ^31^, ^32^, ^33^, ^34^, ^35^, ^36^] Additionally, to realize the recovery of photocatalysts, a few heterogeneous photocatalytic systems such as AgI/BiVO_4_,^[37]^ TiO_2_,[^38^, ^39^] Pt/WO_3_,^[40]^ CDs^[41]^ and g‐C_3_N_4_ ^[42]^ heterojunctions have also demonstrated excellent photocatalytic activity. However, the majority of these strategies are hampered by the limited availability of photocatalysts, primarily due to their high cost and the necessary preparation procedures, constraints that have significantly curtailed the practical application of these catalysts. In recent years, photocatalyst‐free strategies are attracting significant attention (Scheme 1b).[^43^, ^44^, ^45^, ^46^, ^47^, ^48^] For example, Wang group developed a photocatalyst‐free method for the N‐methyl oxidation of N,N‐dimethylaniline derivatives, which employed O_2_ as the oxidant under near‐UV irradiation.^[43]^ In 2021, Fu group designed a class of pyridine‐containing (Z)‐triaryl‐substituted highly conjugated alkenes capable of undergoing aerobic oxidative cleavage under light without any photocatalysts.^[44]^ Moreover, the scope of photocatalyst‐free green photooxidation has been extended to include reactions such as sulfide‐to‐sulfoxide and aldehyde‐to‐carboxylic acid oxidations.[^45^, ^46^, ^47^, ^48^] The proposed mechanism of these catalyst‐free photooxidative strategies was based on the generation of reactive oxygen species (ROS), such as singlet oxygen (^1^O_2_) and superoxide radical (O_2_^−^·), which undergoes energy transfer (EnT) and SET between the excited substrates and O_2_ under light irradiation. Considering this, there is an opportunity to develop an easy photo‐driven platform that would facilitate access to benzylic radicals with the absence of any photocatalysts, even addictives, achieving selective carbonylation through the use of molecular oxygen alone.

**SCHEME 1:**
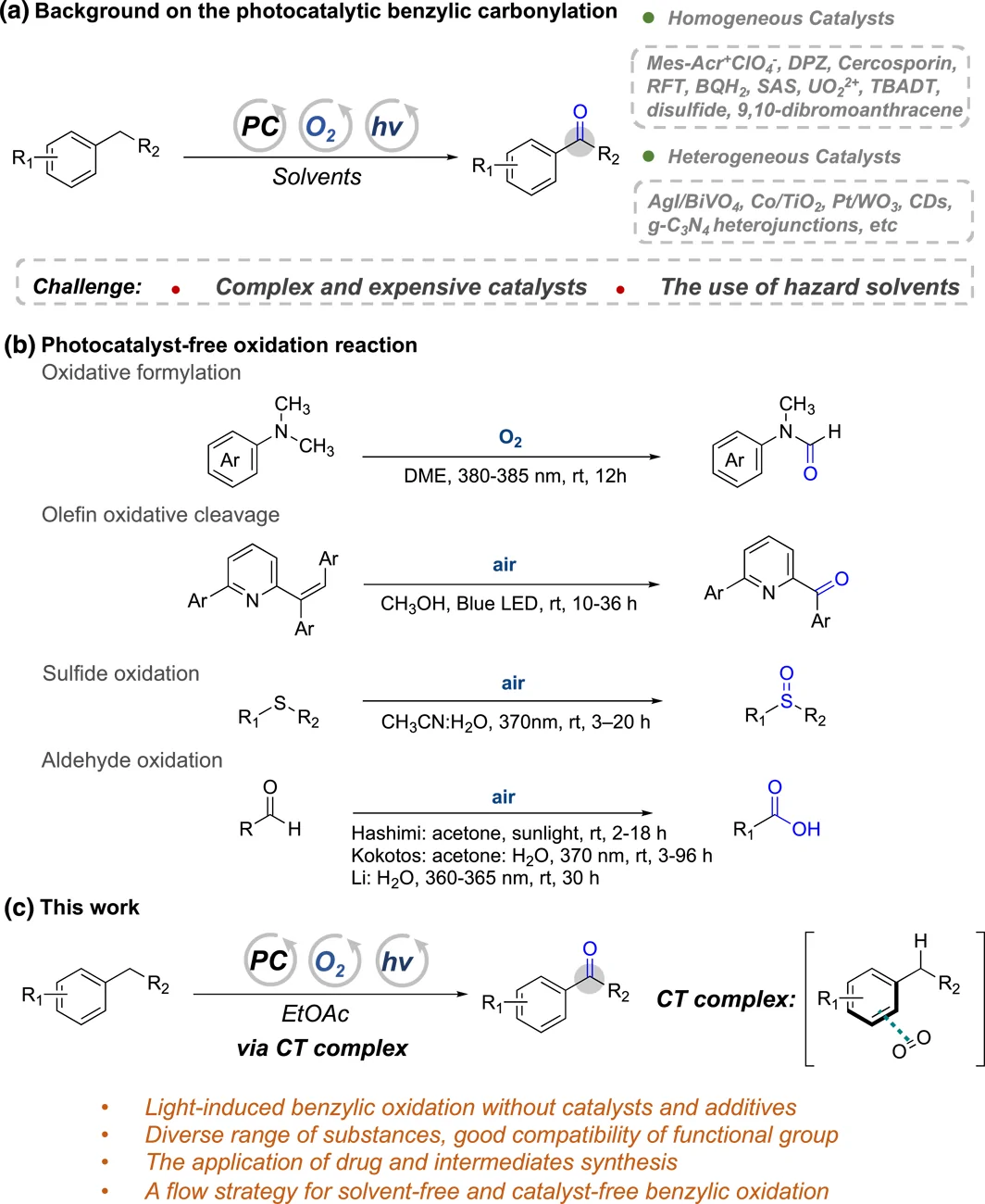
(a) Background on the photocatalytic benzylic carbonylation. (b) Photocatalyst‐free oxidation reaction. (c) This work.

Typically, charge transfer (CT) complexes are recognized as a useful photocatalyst‐free strategy and an innovation within a catalytic regime, which exploited an intracomplex SET or CT between an electron‐rich and electron‐poor molecules by photoactivation, generating a radical ion pair with respect to key radical intermediates generation by direct homolysis.[^49^, ^50^] In this complex, the interaction strength of donors and acceptors can be estimated based on their standard oxidation and reduction potentials.^[51]^ For example, oxygen (−0.78 V vs. SCE, in MeCN)^[52]^ can act as an electron acceptor, which makes it a potential partner to form CT complex with electron‐donors. For example, the CT complex of hexamethylbenzene (1.49 V vs. SCE, in MeCN)^[53]^ with oxygen was reported to afford the benzylic oxygenated products under UV irradiation (254 nm), accompanied by the formation of singlet oxygen. The photochemistry process of CCT pairs involving oxygen has been investigated and diverse types of behavior were discovered depending on the structure of substrates[^54^, ^55^, ^56^, ^57^, ^58^]: (1) the excited CCT pair could be converted to the substrates cation radical pair and the superoxide ion, facilitating the oxidation reaction; (2) singlet oxygen excited by the triplet‐state substrates reacts with substrates itself, if reactive; and (3) if not reactive, the oxygen and substrate are deactivated to the ground state. Based on the above special behavior of CCT pair involving oxygen, electron rich compounds, such as ethers,^[59]^ amines,^[60]^ and styrenes,^[61]^ have been proposed to be oxygenated through photo‐irradiation without any catalysts. However, during this process, high energy radiation (UV, ultraviolet) is required to deliver radical species. In this work, we successfully achieved a series of benzylic substrates oxygenated under visible‐light irradiation for the formation of the carbonyl compound with the absence of any photocatalyst or additives (Scheme 1c).

## 2 RESULTS AND DISCUSSION

We initiated our investigations by the photo‐oxidation carbonylation of 4‐bromoethylbenzene **1**, as the model reaction, utilizing molecular oxygen to optimize the reaction parameters, as shown in Table 1 and more details as shown in Supporting Information S1: Table S1–S4. When the model reaction was proceeded under the irradiation of LED (395–400 nm) in CH_3_CN at room temperature for 24 h, the oxidation product 4‐bromoacetophenone **2** was obtained in 48% yield (Table 1, entry 1). With the wavelength of 395–400 nm as the light source, we first studied the influence of different solvents on the reaction (Supporting Information S1: Table S1). The oxidation reaction did not occur in the water, and only trace amounts of carbonylation products were detected in some solvents, such as acetone, CH_3_OH, DMSO, DMF, toluene and THF (Table 1, entry 2, 3, 5–9). Other common solvents, such as EtOAc, DCE and CHCl_3_, were also tested, affording measurable numbers of the desired products **2**. In particular, among the tested solvents, the yield was the highest in the EtOAc. Furthermore, it is worth mentioning that the target product **2** was acquired with a yield of 70% in the absence of solvents (Table 1, entry 12). Having defined green eco‐friendly EtOAc as the optimum solvent, we next evaluated the effect of different wavelengths with the Kessil lamps (Supporting Information S1: Table S2). It was found that the product **2** was formed in 26% and 62% yield with 370 and 390 nm, respectively (Table 1, entry 13 and 14). Changing the reaction time from 24 to 12 h led to the formation of **2** in an increased yield; particularly, 84% yield was obtained with the wavelength of 390 nm (entry 14). Enhancement of light source intensity to 30 W could reduce the reaction time with 80% yield (Table 1, entry 17). When the wavelength of the kessil lamp was in the range of 427–456 nm, however, few products were detected or the reaction did not happen (Table 1, entry 15 and 16). The control experiments without light or molecular oxygen did not observe any oxidative products, suggesting that both light and oxygen play a crucial role in the reaction (Table 1, entry 18 and 19).

**TABLE 1:** Optimization of the reaction conditions.^a^

Entry	Light source	Solvent	Time [h]	Yield [%]^b^
1	395–400 nm	CH_3_CN	24	48
2	395–400 nm	Acetone	24	Trace
3	395–400 nm	CH_3_OH	24	Trace
4	395–400 nm	EtOAc	24	76
5	395–400 nm	DMSO	24	Trace
6	395–400 nm	DMF	24	Trace
7	395–400 nm	Toluene	24	Trace
8	395–400 nm	H_2_O	24	NR
9	395–400 nm	THF	24	Trace
10	395–400 nm	DCE	24	59
11	395–400 nm	CHCl_3_	24	11
12	395–400 nm	/	24	70
13	370 nm	EtOAc	24, 12	26, 49
14	390 nm	EtOAc	24, 12	62, 84
15	427 nm	EtOAc	24	Trace
16	456 nm	EtOAc	24	NR
17^c^	390 nm	EtOAc	10	82
18	Darkness	EtOAc	24	NR
19^d^	390 nm	EtOAc	24	NR

^a^ Reaction conditions: 4‐bromoethylbenzene (**1**, 0.50 mmol), solvents (1.5 mL), with the oxygen atmosphere by O_2_ balloon under the irradiation of the given light source for 24 h at the room temperature. Note: the power of the light source is 10 W.

^b^ Yield was determined by ^1^H NMR with the internal standard CH_3_NO_2_.

^c^ 30 W 390 nm Kessil lamp.

^d^ N_2_ atmosphere.

The viability of the catalyst‐free photoinduced oxidation of benzylic C‐H bonds strategy was explored, and the scope of the aromatic benzyl derivative is summarized in Scheme 2. An array of ethyl benzenes with electron‐withdrawing and ‐donating groups were examined (**3**–**9**). Electron‐withdrawing group, including 4‐CN (**4**), 4‐COOH (**5**), o‐, m‐ and p‐substituted halogen group (**2**, **3**, **6**, **7**), can smoothly undergo oxidative reaction in 28%–81% yield. The gram‐scale experiment with **2** was performed in slightly lower yield. For substituted compounds with electron‐donating groups (**8**, **9**), the desired products were obtained with 70%–79% yield. However, when arene substrates bearing methoxy‐ and nitro‐groups, no corresponding products were observed. In addition, the scope of cyclic benzylic compounds with various ring types and sizes were tested in a mixture of DMSO and water (Supporting Information S1: Table S5 and S6). It is notable that X = O and small ring sizes (**12**) have positive impacts on the carbonylation without any catalyst. The pyridinyl substrate systems furnished the corresponding products with low yield in long reactive time (**14**, **15**). Moreover, dihydrobenzofuran, as reactant, could also be oxidated successfully to the corresponding products **23**. Furthermore, this methodology was compatible with other aromatic rings, such as 1‐ and 2‐ substituted naphthalene and thiophene (**16**, **17**, **19**). The α‐site of ethylbenzene substituted with benzyloxy group afforded the carbonylated product 18 with low yield due to the oxidative cleavage. Moreover, a series of carbonyl compounds as photocatalysts, including benzophenone (**20**), 1‐acenaphthenone (**24**), fluorenone (**25**) and anthraquinone derivatives (**21**, **22**, **26**, **27**), were acquired with 80%–95% yield. When triphenylmethane was used as the substrate, similarly, the oxidative cleavage event occurred on the benzylic site to obtain the main product **20**. Notably, dapagliflozin intermediate (**29**) was good reactive to undergo the benzylic C‐H carbonylation; however, we were surprised to find that α‐site of ether structure was similarly oxygenated to afford the ester products. The substrate bearing similar structure with **29** was prepared for the oxidation of α‐site of ether, and the ester product **30** was obtained with 18% yield, which could be further expanded. When substrates bearing unreactive phenolic hydroxyl groups were modified into esters by carboxylic acids (**31**, **32**), this benzylic C‐H oxidation proceeded smoothly. Through this decoration, the products with hydroxyl were acquired from the hydrolysis of esters. We estimated the synthetic utility of this method for the late‐stage functionalization of pharmaceuticals, such as celestolide and dapagliflozin, resulting in the formation of corresponding carbonyl products (**28**, **33**).

**SCHEME 2:**
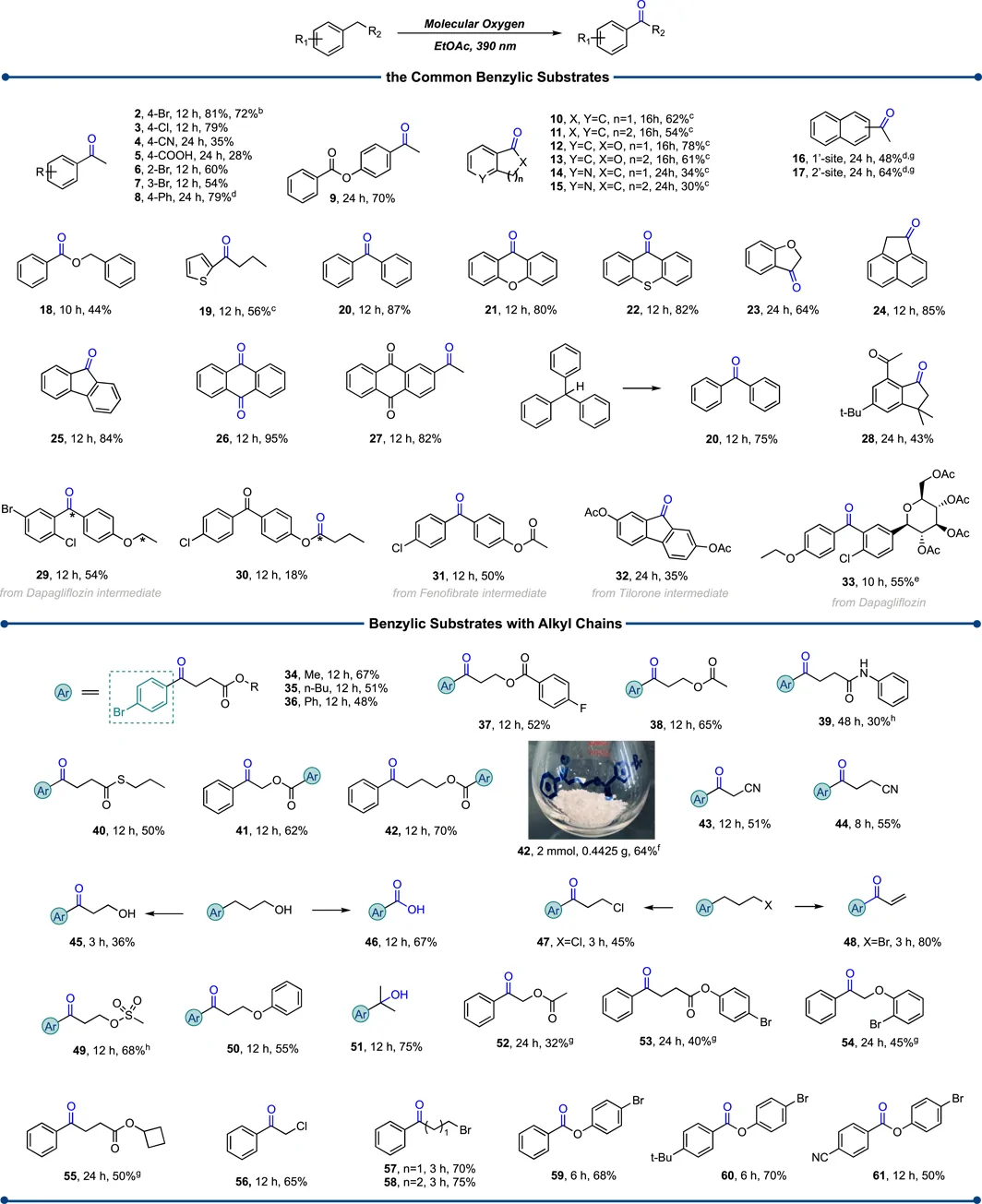
Substrate scope of carbonylation^a^. ^a^Reaction conditions: substrates (0.5 mmol), EtOAc (1.5 mL), with the oxygen atmosphere by O_2_ balloon under the irradiation of 30 W 390 nm Kessil lamp for the given time at the room temperature, isolated yield. ^b^Substrate (5 mmol). ^c^The mixture of DMSO and H_2_O (2:1) as solvent. ^d^DMSO as solvent. ^e^Substrate (0.2 mmol). ^f^Substrate (2 mmol). ^g^30 W 365 nm LED. ^h1^H NMR yield with CH_3_NO_2_ as an internal standard

To further probe the functional group tolerance of the protocol without photocatalyst, we explored alkylarenes containing a broad array of diverse functionalities in the alkyl chain. To our delight, different kinds of functional groups, such as ester (**34–38**, **41–42**, **52–53**, **55**), amide (**39**), thioester (**40**), hydroxyl (**45**), halogen (**47**, **56–58**), methylsulfonyl (**49**), cyano (**43**, **44**), ether (**50**, **54**), performed well in the benzylic oxygenation with moderate yields. Specially, for the hydroxyl substituted in the alkyl chain, the benzoic acid (**46**) as main oxidative products were formed as the reaction time prolongs, possibly due to a β‐scission pathway[^62^, ^63^]; dehalogenation behavior occurred to afford the carbonyl alkene (**48**) with a good yield. Noteworthy, the halo functionality (**56**–**58**) exhibited excellent activity in this benzylic C‐H oxidation, providing an opportunity for further functionalization. Apart from the oxidation of 2^o^ benzylic sites, the cleavage of the tertiary benzylic C‐H bond could also occur to form the hydroxyl product (**51**) under light irradiation. Benzyl ethers were also competent substrates and yielded the corresponding ester products (**59**–**61**), respectively.

To demonstrate the synthetic utility of the carbonylated products toward useful molecular scaffolds, a variety of transformations were carried out (Scheme 3). Using this strategy, the anti‐inflammatory drug fenbufen (**62**) and hypolipidemic medication fenofibrate (**63**) were obtained in good yields under our conditions. Furthermore, when the exposed hydroxyl groups were decorated through an esterification reaction, the benzylic C‐H bond of the pharmaceutical intermediates could be oxidated to obtain the natural product acetosyringone (**65**). Besides, the presence of hydroxyl groups within the alkyl chain of aromatic alkanes is not compatible under standard reaction conditions. As shown in the substrate scope, we discovered that the *p*‐bromobenzoyl structural unit possibly plays a catalytic role in the oxidation reaction.^[64]^ Consequently, we facilitated the conversion of these hydroxyl groups into esters through esterification with *p*‐bromobenzoic acid. The subsequent oxidation‐carbonylation reaction proceeded smoothly, culminating in the formation of the target products **67** and **68**, which were obtained in moderate yields after hydrolysis. Using the ketones **57** and **58** as reaction precursors, amination (**69**), reduction (**70**), cyanation (**71**) and azidation (**72**) reactions all proceeded well. After these modifications, the medical active ingredients were further synthesized, such as haloperidol analogs,^[65]^ inhibitor of mammalian 15‐lipoxygenase,^[66]^ inhibitor of AChE^[67]^ and anticonvulsant agents.^[68]^

**SCHEME 3:**
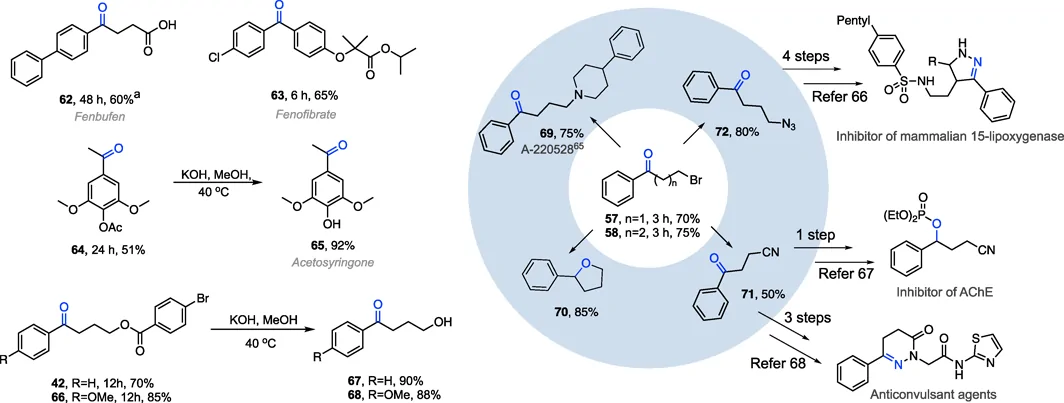
Application and extension of this strategy. Unless otherwise noted, substrates (0.5 mmol), EtOAc (1.5 mL), with O_2_ balloon under the irradiation of 30 W 390 nm Kessil lamp for the given time at the room temperature, isolated yield. ^a^DMSO as the solvent.

Photochemical reactions in batch systems suffer from light attenuation at larger scales due to the Bouguer‐Lambert‐Beer law, limiting their scalability.[^69^, ^70^] Continuous‐flow photoreactors overcome this limitation by ensuring uniform light penetration, enabling efficient scale‐up without compromising reaction performance.^[69]^ For solvent‐free and catalyst‐free reaction systems, the photocatalytic oxidation of liquid benzyl substrates ‐ owing to their inherent fluidity ‐ is particularly well‐suited for continuous‐flow scale‐up. Thus, we have developed an in‐house continuous‐flow platform specifically designed for the solvent‐free photocatalyst‐free oxidation of tetralin (**11a**) on 30 mmol scale (Supporting Information S1: Figure S3). Preliminary optimization studies demonstrated that under continuous flow conditions, the solvent‐free system exhibits superior performance compared to solvent‐based systems (Supporting Information S1: Table S7). With our continuous‐flow setup, 56% isolated yield of **11** over a residence time of 78.8 min was obtained (Scheme 4). Then, E‐factor (6.7) was also calculated, which demonstrated the greenness of our continuous‐flow strategy. Compared to batch processes, solvent‐free continuous reactions offer an efficiency increase of over 150‐fold.

**SCHEME 4:**
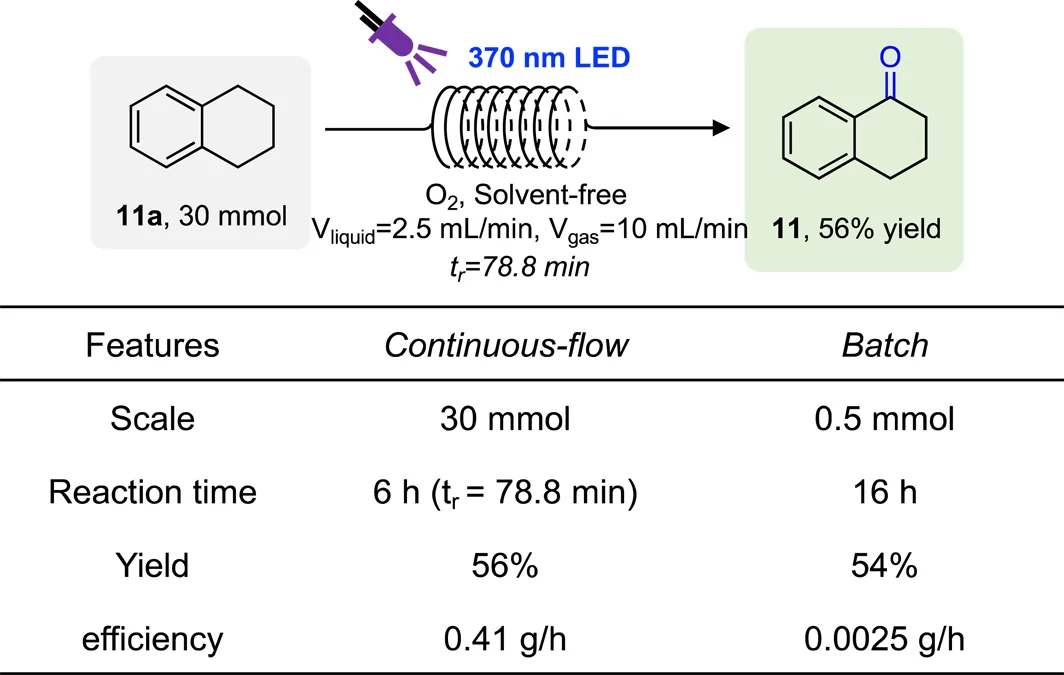
Circulation continuous‐flow synthesis

To elucidate the mechanism of photo‐oxidation of benzylic C‐H without catalyst, the reaction process was supervised via continuous sampling by NMR. As shown in Figure 1, the desired product **2** was not obtained during the initial stage of reaction, while the rate of reaction accelerated gradually after a product‐accumulation period of 3 h, which could be caused by carbonylation products as potential initiators. Thus, we divided the whole reaction process into the substrate‐induced stage and product‐induced stage to clearly identify the mechanism of the reaction.

**FIGURE 1:**
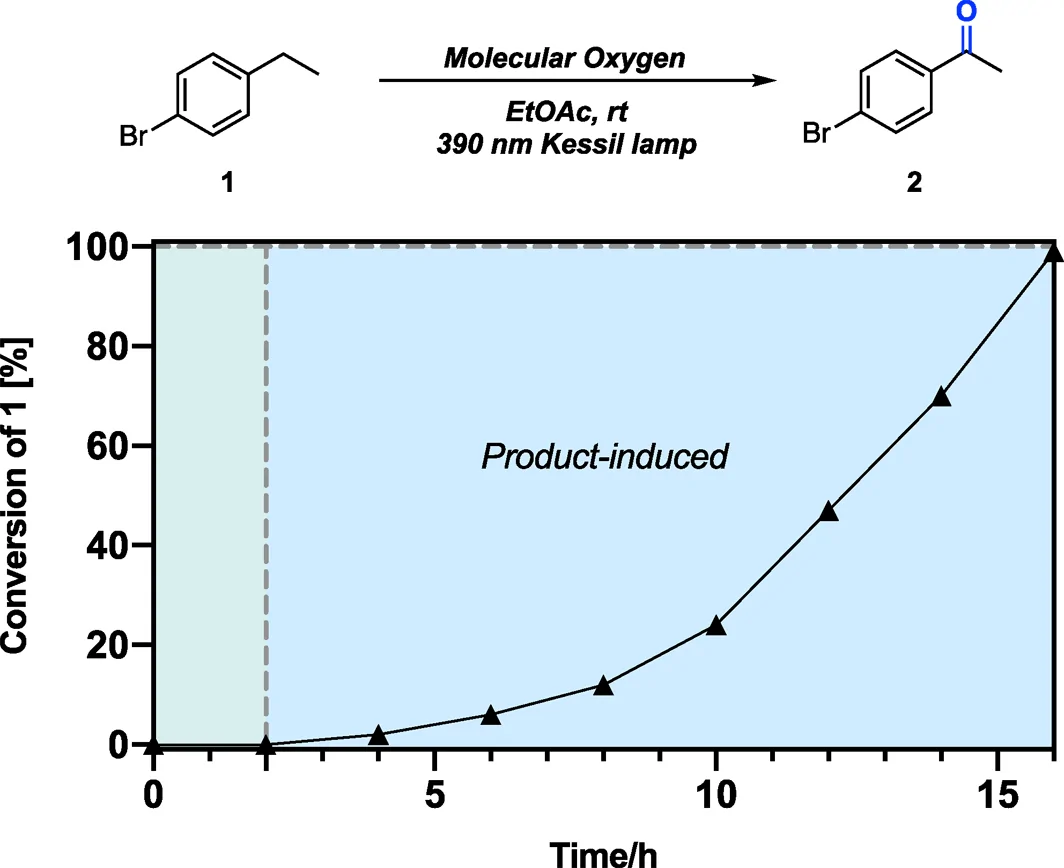
Analysis of the reaction process.

In the substrate‐induced stage, a series of inhibitor experiments were conducted to further investigate the reactive species of the oxidative benzylic C‐H bond (Scheme 5a and Supporting Information S1: Table S8). When radical scavengers TEMPO and BHT were added to the model reaction of **1**, respectively, the oxidative carbonylation was significantly inhibited along with the formation of benzylic radical with TEMPO detected by HRMS (Scheme 5b, Supporting Information S1: Figure S11 and S12), implying a free radical reaction process. The yield decreased significantly with the addition of singlet oxygen inhibitors anthracene and DABCO, while the participation of benzoquinone as a superoxide anion radical inhibitor also resulted in the cessation of the reaction. The electron‐spin resonance (ESR) spectra were recorded using 2,2,6,6‐Tetramethylpiperidine (TEMP) and 5,5‐dimethyl‐1‐pyrroline‐N‐oxide (DMPO) as the spin trap for capturing ROS, and the characteristic adduct of ^1^O_2_ with TEMP and O_2_^−·^ with DMPO were captured, respectively, after being exposed to visible irradiation (Scheme 5c,d), which was consistent with the previously reported signals.[^71^, ^72^, ^73^] The above results indicate that ROS of the reaction system involve ^1^O_2_ and O_2_^−·^. The addition of DDQ and AgNO_3_ as electron scavengers inhibited obviously the oxidative reaction, suggesting the process of ET. Light on/off experiments demonstrated that continuous photoirradiation is required to sustain the oxidative reaction (Supporting Information S1: Figure S16).

**SCHEME 5:**
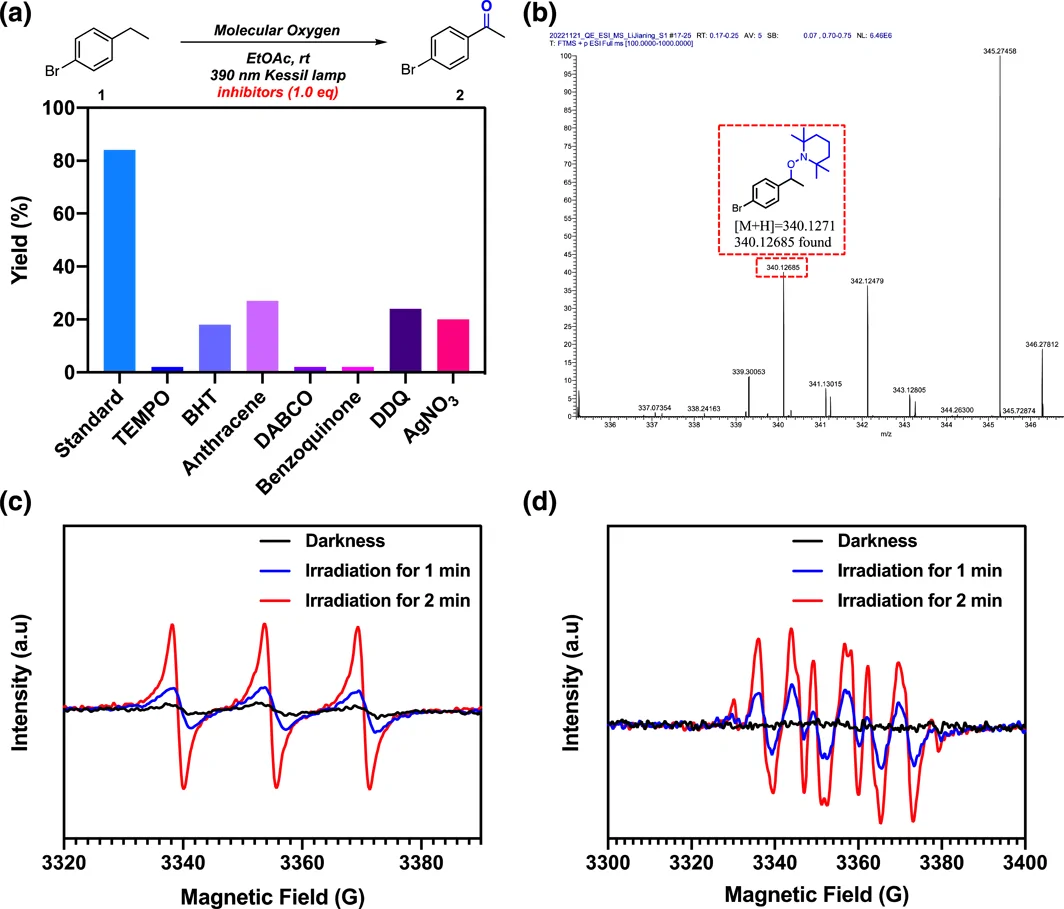
(a) Inhibitor experiments. (b) HRMS of benzylic radical. (c) and (d) electron‐spin resonance spectra of singlet oxygen and superoxide anion.

Inhibitor experiments revealed the occurrence of an ET process and the generation of O_2_^−^ during the reaction. This suggests that ET may take place between substrate **1** and O_2_, likely via the formation of a charge‐transfer (CT) complex under light excitation. To confirm the formation of such a CT complex, UV‐Vis spectroscopy was performed on substrate **1** under N_2_ and O_2_ bubbling, respectively. Given that solvent evaporation during bubbling could alter the substrate concentration and interfere with spectral observation, the experiments were conducted under solvent‐free conditions using pure substrate **1**. As shown in Figure 2, compared to the absorption profile after N_2_ bubbling, a red shift is observed in the 300–320 nm region following O_2_ bubbling. Furthermore, extending the bubbling time did not induce further red shifts consistent with the saturation of dissolved oxygen. These results support the possible formation of a CT complex between substrate **1** and O_2_.

**FIGURE 2:**
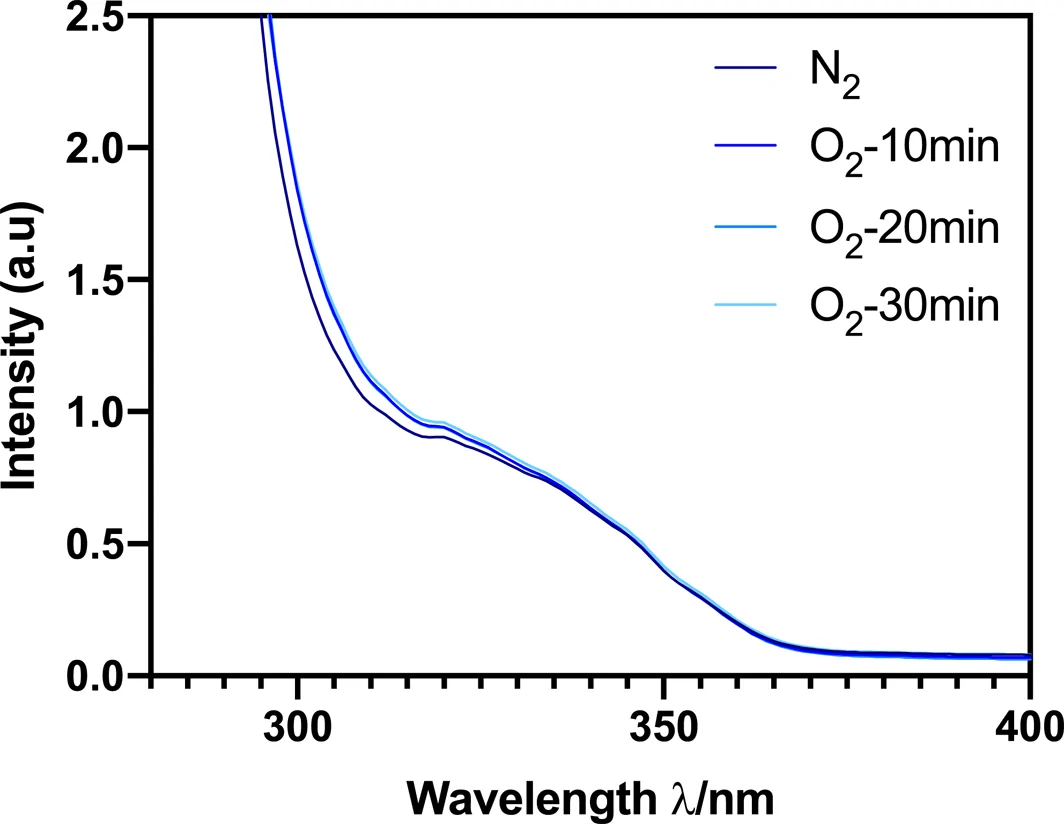
UV‐vis absorption spectra of **1** under O_2_/N_2_ atmosphere.

To further investigate the interaction between substrate **1** and O_2_, femtosecond transient absorption spectroscopy (fs‐TAS) was performed (Supporting Information S1: Figure S26 and Figure S27). Pure **1** was bubbled with O_2_/N_2_ and tested under O_2_ and N_2_ atmospheres, respectively. The results are shown in Scheme 6 and Supporting Information S1: Figure S26. A clear excited‐state absorption signal of **1** was observed in the range of 350–700 nm. However, depending on the gas atmosphere, the absorption peaks appeared at different positions. Under N_2_ atmosphere, the absorption mainly occurred between 550 and 590 nm, which can be attributed to the excited state of **1** (Scheme 6b). In contrast, under O_2_ atmosphere, photoinduced absorption appeared around 410 nm, likely resulting from reduced **1** after intermolecular ET between **1** and O_2_ upon photoexcitation (Scheme 6a). Subsequently, the decay kinetics of the transient spectra were fitted. The decay lifetime of **1** under N_2_ was 650.4 fs, while under O_2_ it shortened to 542.1 fs (Supporting Information S1: Figure S28 and S29). These results indicate that the presence of O_2_ shortens the excited‐state lifetime of the substrate, which may be attributed to ET between the substrate and O_2_.^[74]^

**SCHEME 6:**
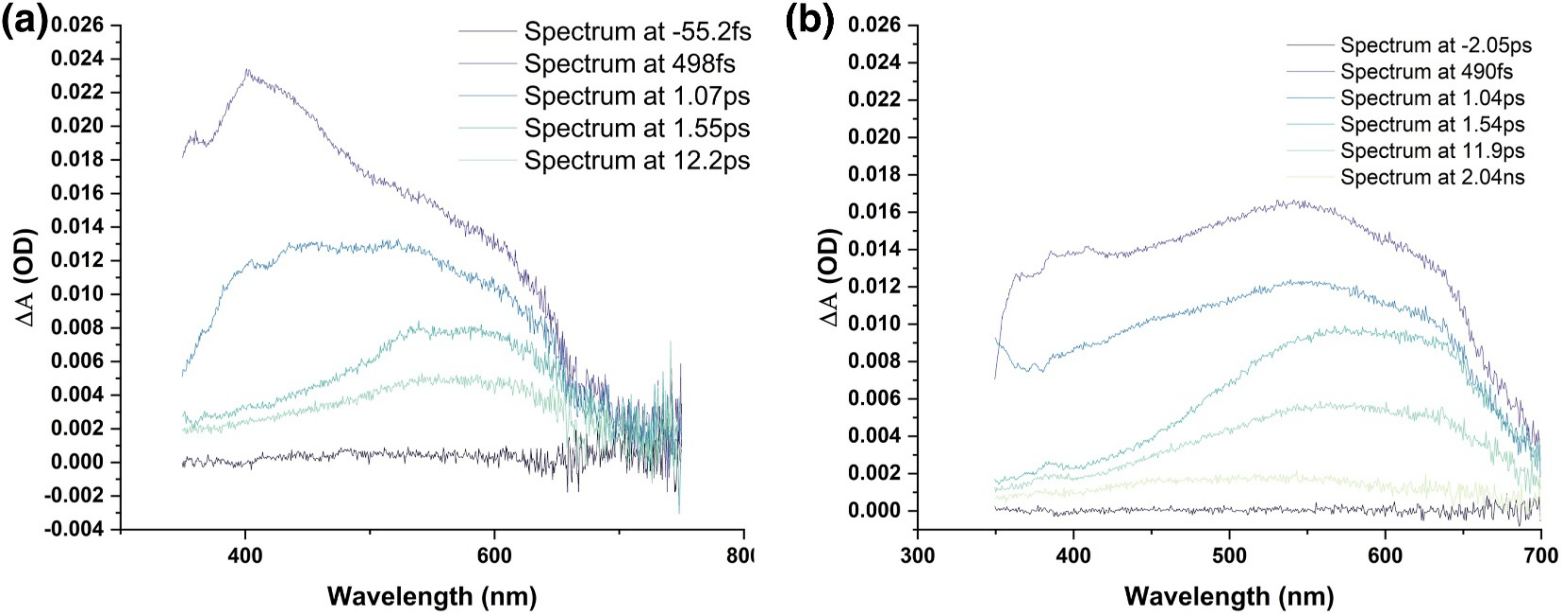
fs‐TAS of substrate **1** (a) Under O_2_ atmosphere. (b) Under N_2_ atmosphere.

On the other hand, carbonyl compounds as photosensitizers, photoinitiators and photocatalysts have been explored, which have enriched the development of the field of photocatalysis. Under the excitation of light irradiation, aromatic ketones could transition from ground state to singlet state due to the presence of an unpaired electron in a nonbonding orbital, then suffering an intersystem crossing (ISC) from singlet to triplet state, known as excellent triplet sensitizers. Thus, it was supposed that product ketones as a potential photoinitiator resulted in the experimental phenomenon of accelerated reaction rate.

To test this hypothesis, confirmation studies were performed by selecting neutral ethylbenzene as the reaction substrate along with aromatic ketone derivatives as the photoinitiator under purple (390 nm) irradiation (Table 2). Through screening aromatic ketones possessing benzoyl groups as structural unit, a few conclusions could be drawn from the confirmation studies: (1) aromatic ketones indeed could induce the benzylic oxidation reaction, apart from the substituent group R_2_ on aromatic rings as electron‐donating group such as 4‐OMe; (2) the electron‐withdrawing of aromatic rings was critical in the carbonylation reaction, whether the substitution of electron‐withdrawing group on R_1_ or R_2_ group or pyridine replacing benzene ring, presumably the interaction between aromatic ketone and ethylbenzene, thereby promoting the formation of CT complexes; (3) among the screening aromatic ketone derivatives, 4‐bromoacetophenone had an excellent performance on catalyzing the oxidative reaction, which could be a potential photocatalyst in the field of photocatalysis.

**TABLE 2:** Ethylbenzene oxidation catalyzed by aromatic ketone derivatives.^a^

Entry	Ar	Ar‐R_2_	R_1_	Conv. [%]	Yield [%]^b^
1	Ph	4‐OMe	Me	NR	ND
2	Ph	4‐F	Me	36	17
3	Ph	4‐Br	Me	99	73, 71^d^
4	Ph	4‐NO_2_	Me	78	46
5	Ph	4‐CN	Me	86	53
6	Ph	4‐SO_2_Me	Me	30	16
7	Ph	2‐Br	Me	99	48
8	Ph	3‐Br	Me	99	53
9	Ph	H	CF_3_	53	29
10	Ph	H	Cy	50	24
11	Ph	H	Ph	85	65
12	Ph	3,4,5‐F	Me	59	34
13	Ph	H	Me	29	8
14	Ph	4‐Br	H	65	51
15	Py	H	Me	71	42
16^c^	Ph	4‐Br	Me	99	71

^a^ Reaction conditions: ethylbenzene (**73a**, 0.5 mmol), aromatic ketones (0.5 mmol, 1.0 eq.), EtOAc (1.5 mL), O_2_ balloon, 30 W 390 nm Kessil for 18 h at the room temperature.

^b^ Yield was determined by GC‐FID with the internal standard cyclohexanone.

^c^ 0.2 eq. aromatic ketone.

^d^ Isolated yield.

The kinetic isotope effect (KIE) study can provide strong evidence of C‐H bond cleavage as a rate‐limiting step. We first conducted the oxidative reaction of diphenylmethane‐d2 in the absence of catalysts, and there was no the desired product obtained due to the higher dissociation energy of the benzylic C‐D bond needed (Scheme 7a). Thereby, the subsequent KIE study would use different carbonylation products as the initiator (Supporting Information S1: Figure S19–S22). When 4‐ethyl‐1,1′‐biphenyl and 4‐(ethyl‐1,1‐d2)‐1,1′‐biphenyl were used as substrates, a KIE value of 4.2 was measured via a parallel rate experiment, suggesting that benzylic C‐H bonds might be a rate‐determining step (Scheme 6a). Another observation is that the KIE of diphenylmethane (1.8) and 1‐ethyl‐4‐methoxybenzene (1.5) observed in both parallel reactions indicated the isotope effect was weakened and the cleavage of benzylic C‐H bond might not be involved in a rate‐determining step possibly related to the electrical effect of aromatic rings in substrates. Similarly, we observed the KIE of 1‐ethyl‐4‐methoxybenzene (1.3) when 4‐nitroacetophenone was used as the initiator (Scheme 7a). These results reveal that the other possible determining step would exist instead of C‐H bonds cleavage, such as the formation of the CT complexes. This speculation is consistent with the conclusion in earlier study by Wagner,^[75]^ which also demonstrates the possible existence of CT complexes. Subsequently, competition experiments of 1‐ethyl‐4‐methoxybenzene and 4‐acetylbenzonitrile with 2 as the initiator was carried out. The ratio of the products indicates that the election‐rich 1‐ethyl‐4‐methoxybenzene is significantly more reactive. When 4‐nitroacetophenone was used as the initiator, the experimental results suggested that the significant electronic preference was more obvious due to the stronger intermolecular interaction (Supporting Information S1: Figure S23).

**SCHEME 7:**
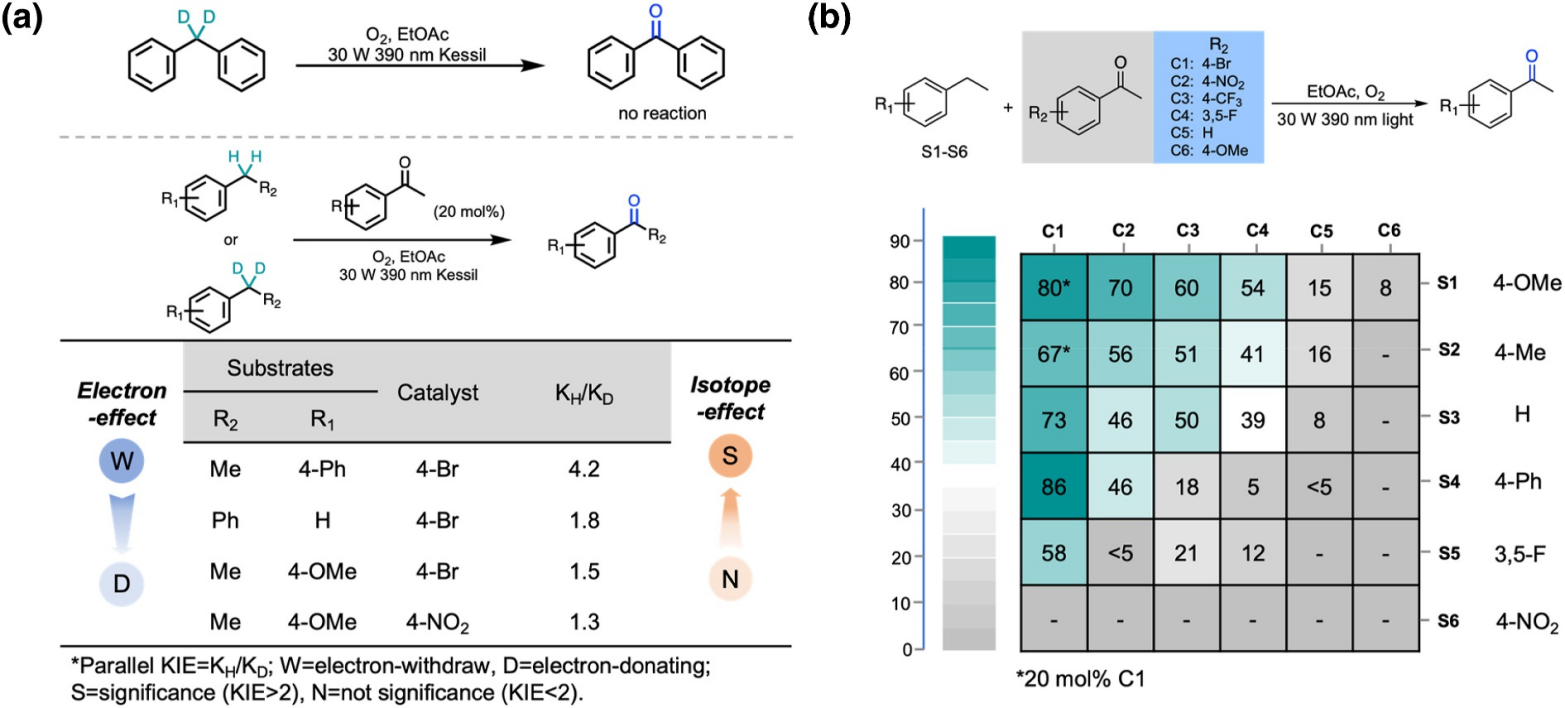
(a) The kinetic isotope effect study. (b) The competitive experiment.

To further demonstrate the interplay of electronic effects, we prepared substrates **S1–S6** that contain electron‐donating and ‐withdrawing groups reactions with **C1–C6** products as initiators providing the heat map shown in Scheme 7b. These results show that the reactivity depends on the electronic nature of the substrate and products. Specially, the reactivity increases when using strong electrophilic ketones and electron‐rich substrates due to the formation of CT complexes.

Based on the above mechanistic investigations and literature reports,[^43^, ^44^, ^45^, ^54^, ^64^] a plausible reaction mechanism is proposed. As illustrated in Scheme 8, the mechanism can be divided into three pathways (Paths a‐c). In Path a, substrate **1** and O_2_ can form a CT complex. Upon light excitation, the substrate forms excited‐state 1*, and the CT complex also reaches an excited state. Under these high‐energy excited states, O_2_ can be converted into ^1^O_2_ via EnT. Additionally, the excited CT complex may undergo intermolecular ET, generating benzyl radical cations **A** and O_2_^−.^ Subsequently, intermediate **A** undergoes deprotonation to form the key benzyl radical **B**. Radical **B** can then react with O_2_^−.^, followed by protonation to yield hydroperoxide intermediate **E**, which hydrolyzes to form product **2**. Alternatively, benzyl radical **B** may combine with ^1^O_2_ to form peroxy radical **C**, which further abstracts a hydrogen atom from **1** via HAT, affording intermediate **E** and ultimately product **2**. In Path b, substrate **1** and product **2** can also form a CT complex. Photoinduced ET within this complex generates the anion radical **F** of **2** and benzyl radical cation **A**. The former can further undergo ET with O_2_ to produce O_2_^−.^ Finally, intermediate **A** undergoes deprotonation to form **B**, which enters the same oxidation sequence as in Path a to yield product **2**. Given that ^1^O_2_ possesses HAT capability,^[76]^ Path c is proposed. The ^1^O_2_ generated via EnT engages in HAT with **1**, producing radical **B**, while the resulting hydroperoxyl radical can further undergo HAT to generate another radical **B** and hydrogen peroxide (H_2_O_2_, Supporting Information S1: Figure S17 and S18). Finally, radical **B** reacts with ROS to give product **2**.

**SCHEME 8:**
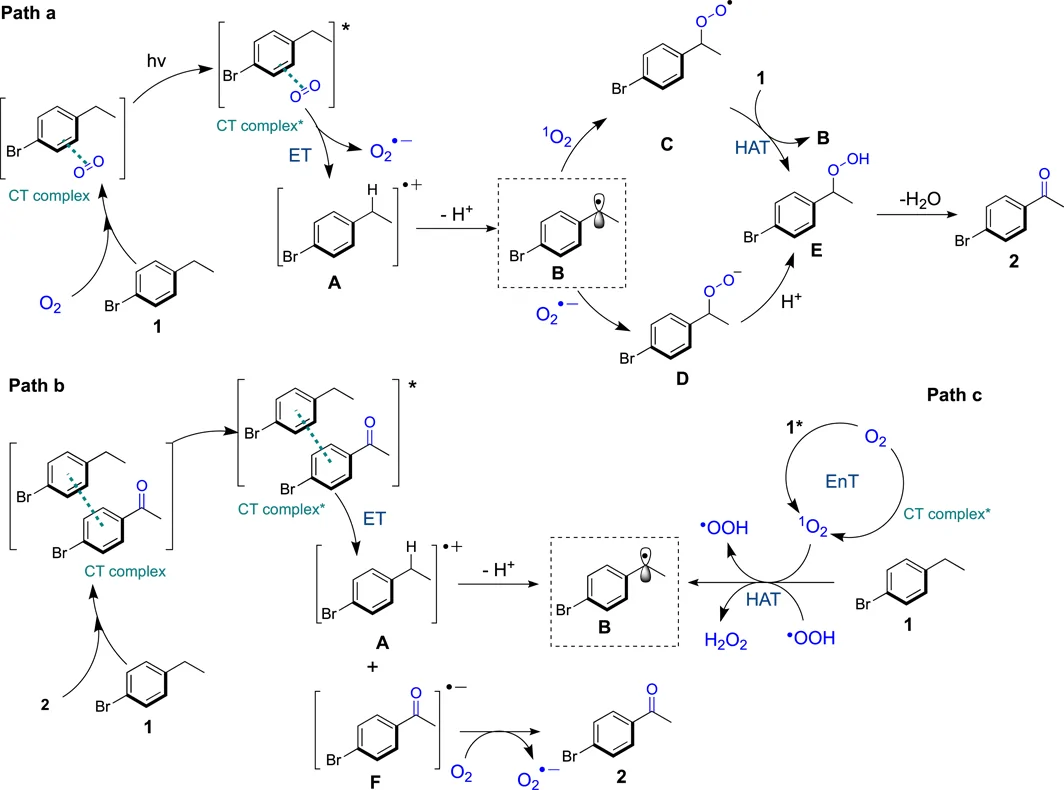
Possible mechanism

## 3 CONCLUSION

In summary, we developed a green and sustainable photoinduced protocol for the oxidation of benzylic C‐H bond utilizing molecular oxygen without any photocatalyst and additive. Employing ethyl acetate as a green solvent and visible‐wavelength irradiation of 390 nm, a wide range of aromatic alkanes undergo carbonylation to provide diverse and available ketones, such as drug derivatives and intermediates. This strategy features mild and metal‐free reaction conditions, high atom economy, and environmental Friendliness. Applying our catalyst‐free photo‐induced carbonylation strategy, some drugs were successfully synthesized. Finally, mechanistic studies indicate that the CT complex plays an important role in the reaction, which is composed of a substrate and oxygen. Moreover, the carbonylated product could promote the oxidation via the CT complex between substrate and product. During the reaction process, singlet oxygen and superoxide anion as major ROS participated in the whole oxidation transformation. Efforts to further explore the application of the CT complex mechanism in photocatalytic oxidations are ongoing.

## 4 EXPERIMENTAL SECTION

*General procedure*: A 10 mL quartz tube equipped with a magnetic stir bar was charged with aromatic alkanes (0.5 mmol) and EtOAc (1.5 mL, 0.33 M). Note: Ethyl acetate can be replaced with DMSO or a mixture of DMSO and H_2_O. The reaction tube was sealed and pumped vacuum, and oxygen was fed to keep a pure oxygen atmosphere with O_2_ balloon. The resulting mixture was performed under irradiation of light (30 W 390 nm Kessil lamps) at room temperature for a given time. After the reaction completed, the reaction solution was concentrated under reduced pressure to yield crude product, which was purified by column chromatography with petroleum ether/ethyl acetate as eluents to afford the target product.

## CONFLICT OF INTEREST STATEMENT

The authors declare no conflicts of interest.

## ETHICS STATEMENT

No animal or human experiments were involved in this study.

## Supporting information

Supporting Information S1

## Data Availability

The data that support the findings of this study are available from the corresponding author upon reasonable request.
