# Supplementary material for: Visible‐light‐promoted catalyst‐free benzylic C‐H oxidation using molecular oxygen as a green oxidant
Source: Smart Mol. 2026 May 17:e70058. Online ahead of print. doi: 10.1002/smo2.70058 (PMC13399385; doi:10.1002/smo2.70058)
Supplement: Supplementary file 1 — Supporting Information S1 [file SMO2-9999-0-s001.pdf]

Supporting Information

Visible-Light-Promoted Catalyst-Free Benzylic C-H Oxidation Using Molecular Oxygen as a Green Oxidant

Jianing Li, Suwen Wang, Huinan Sun, Zhunchao Liu, Xiangmin Tian, Qilei Liu and Qingwei Meng**

Department of Pharmacy, School of Chemical Engineering, Dalian University of Technology, Dalian 116023, China

* *E-mail: liuqilei@dlut.edu.cn; mengqw@dlut.edu.cn*

Content	Page
General information	S1
Investigations of the reaction conditions	S2
General procedure	S6
Synthesis of substrates	S12
The unsuccessful substrates	S23
The benzylic C-H bonds dissociation enthalpies	S24
The by-product of the oxidation reaction	S25
Gram-scale reaction	S26
General procedure for continuous-flow oxidation	S28
Characteriation data of the synthesized compounds	S31
Studies on reaction mechanism	S50
The synthetic application	S72
NMR spectra	S78
Reference	S148

1. General information

All ^{1}H NMR (400/500 MHz) and ^{13}C NMR (101/126 MHz) were recorded on a VARIAN INOVA-400/ 500 M and AVANCE II 400 spectrometer. All chemical shifts are given as δ value (ppm) with reference to tetramethylsilane (TMS) as an internal standard. The peak patterns are indicated as follows: s, singlet; d, doublet; t, triplet; m, multiplet; q, quartet. The coupling constants, J, are reported in Hertz (Hz). Analytical TLC was visualized with UV light at 254nm. Thin layer chromatography was carried out on TLC glass sheets with silica gel 60 F254. Purification of reaction products was carried out by chromatography using silica gel 60 (200-300 mesh). High resolution mass spectrometry data of the products were obtained with UPLC/Q-Tof Mass Spectrometer and were determined by electrospray ionization (ESI). UV-Vis absorption spectra was recorded using a PerkinElmer Lambda 1050+ absorption spectrometer. Fluorescence experiments were performed on a Hitachi F-7100 FL Spectrophotometer. Kessil lamps PR160L was used as irradiation source. The screening reactions were carried out on a WP-TEC-1020 photoreactor purchased from Wattcas Company.

2. Investigations of the reaction conditions

2.1 p-bromoethylbenzene as model substrate

Table S1. Solvents screening experiments[a]

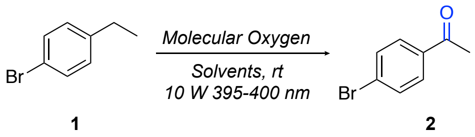

Entry	Solvent	Time [h]	Yield[b][%]
1	CH_3CN	24	48
2	acetone	24	trace
3	CH_3OH	24	trace
4	EtOAc	24	76
5	DMSO	24	trace
6	DMF	24	trace
7	Toluene	24	trace
8	H_2O	24	NR
9	THF	24	trace
10	DCE	24	59
11	$CHCl_3$	24	11
12	/	24	70

[a]Reaction conditions: 4-bromoethylbenzene (1, 0.50 mmol), solvents (1.5 mL), with the oxygen atmosphere by O_2 balloon under the irradiation of 10 W 395-400 nm LEDs for 24 h at the room temperature. [b]Yield was determined by ^1H NMR with the internal standard CH_3NO_2.

Table S2. Light source and reaction time screening experiments[a]

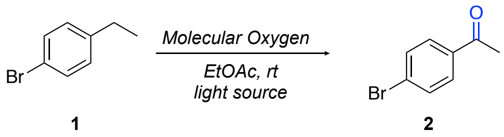

Entry	Light source	Time [h]	Yield[b][%]
1	395-400 nm	24	76
2	370 nm	24	26
3	370 nm	12	49
4	390 nm	24	62
5	390 nm	12	84
6	390 nm	6	51

7	427 nm	24	trace
8	456 nm	24	NR
9[c]	390 nm	10	82
10	Darkness	24	NR

[a]Reaction conditions: 4-bromoethylbenzene (1, 0.50 mmol), EtOAc (1.5 mL), with the oxygen atmosphere by O_2 balloon under the irradiation of 10 W Kessil lamps for given time at the room temperature. [b]Yield was determined by ^1H NMR with the internal standard CH_3NO_2. [c]30 W 390 nm Kessil lamp

Table S3. Atmosphere screening experiments[a]

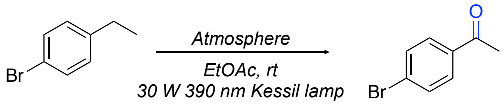

1 2

Entry	Atmosphere	Time [h]	Yield[b][%]
1	O_2	10	82
2	air	10	70
3	N_2	10	NR

[a]Reaction conditions: 4-bromoethylbenzene (1, 0.50 mmol), EtOAc (1.5 mL), with the different atmosphere by balloon under the irradiation of 30 W 390 nm Kessil lamp for 10 h at the room temperature. [b]Yield was determined by ^1H NMR with the internal standard CH_3NO_2.

Table S4. Amounts of EtOAc screening experiments[a]

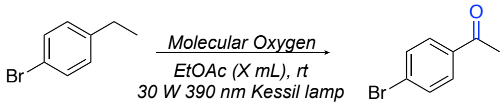

1 2

Entry	X	Time [h]	Yield[b][%]
1	1.5	8	82
2	1.0	8	77
3	0.5	8	63

[a]Reaction conditions: 4-bromoethylbenzene (1, 0.50 mmol), EtOAc (X mL), with the oxygen atmosphere by O_2 balloon under the irradiation of 30 W 390 nm Kessil lamp for 10 h at the room temperature. [b]Yield was determined by ^1H NMR with the internal standard CH_3NO_2.

2.2 Tetrahydronaphthalene as model substrate

Table S5. Solvents screening experiments[a]

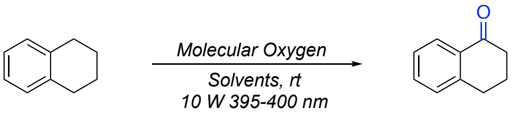

11a **11**

Entry	Solvents	Yield[b][%]
1	acetone	25
2	ethylene glycol	15
3	DMC	16
4	cyclohexane	9
5	1,2-dimethoxyethane	6
6	H_2O	32
7	CH_3OH	11
8	DMSO	18
9	DMF	10
10	PhMe	8
11	CH_3CN	22
12	EtOAc	5
12	CH_3CN:H_2O (1:2)	39
13	CH_3OH:H_2O (1:2)	39
14	DMSO:H_2O (1:2)	40
15	/	30

[a]Reaction conditions: tetrahydronaphthalene (0.50 mmol), solvents (1.5 mL), with the oxygen atmosphere by O_2 balloon under the irradiation of 10 W 395-400 nm LEDs for 16 h at the room temperature. [b]Yield was determined by ^1H NMR with the internal standard CH_3NO_2.

Table S6. Other conditions screening experiments[a]

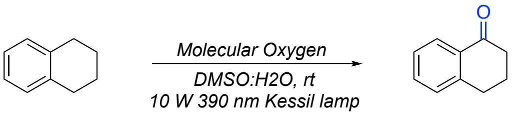

11a **11**

Entry	Light source	solvent	Time [h]	Yield [%][b]
1	395-400 nm	DMSO:H_2O (1:2)	16	40
2	390 nm	DMSO:H_2O (1:2)	16	42
3	390 nm	DMSO:H_2O (2:1)	16	56
4[c]	390 nm	DMSO:H_2O (2:1)	16	60
5	370 nm	DMSO:H_2O (2:1)	16	53

6	427 nm	DMSO:H_2O (2:1)	16	trace
7	darkness	DMSO:H_2O (2:1)	16	NR
8[d]	390 nm	DMSO:H_2O (2:1)	16	NR

[a]Reaction conditions: tetrahydronaphthalene (0.50 mmol), DMSO:H_2O (1.5 mL), with the oxygen atmosphere by O_2 balloon under the irradiation of 10 W Kessil lamps for 16 h at the room temperature. [b]Yield was determined by ^{1}H NMR with the internal standard CH_3NO_2. [c]30 W 390 nm Kessil lamp. [d]N_2 atmosphere.

3. General procedure of catalyst-free oxidation carbonylation of benzylic C-H bond

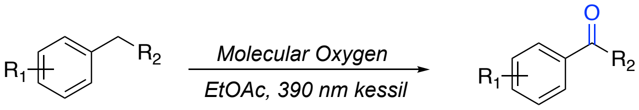

A 10 mL quartz tube equipped with magnetic stir bar was charged with aromatic alkanes (0.5 mmol) and EtOAc (1.5 mL, 0.33 M). The reaction tube was sealed and pumped vacuum, then feeding oxygen to keep pure oxygen atmosphere with O_2 balloon. The resulting mixture was performed under irradiation of light (30 W 390 nm Kessil lamps) at room temperature for a given time. After the reaction completed, the reaction solution was concentrated under reduced pressure to yield crude product, which was purified by column chromatography with petroleum ether/ethyl acetate as eluents to afford the target product.

Note: the temperature probe of stirrer was used to detect the temperature of light irradiation, and the real temperature was displayed on the screen of the stirrer (Figure S1).

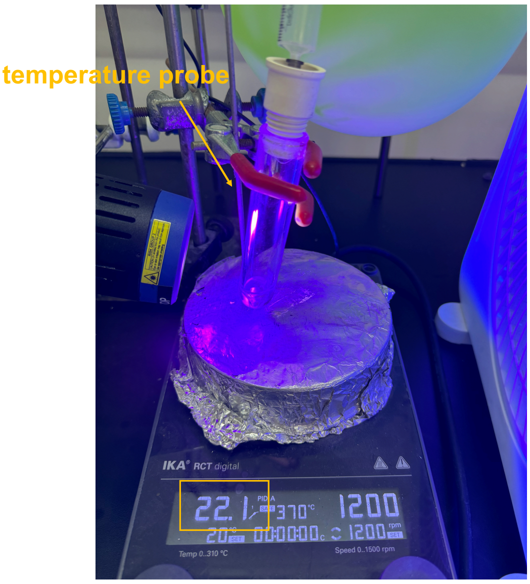

Figure S1. Photoreactor for the benzylic oxidation

4. Synthesis of substrates

The synthesis of ester[1]

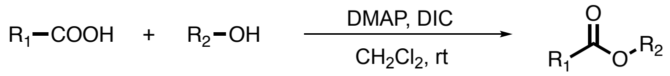

To a solution of carboxylic acid derivatives (5 mmol, 1.0 equiv) in CH_2Cl_2 (20 mL) was added hydroxyl compounds (5 mmol, 1.0 equiv), followed by DIC (6 mmol, 1.2 equiv) and DMAP (1 mmol, 0.2 equiv). The resulting reaction mixture was stirred at room temperature for 12 h, then diluted with CH_2Cl_2, and quenched with 1N HCl. The layers were separated, and the aqueous layer was extracted twice with CH_2Cl_2. The combined organic solution was washed with brine, dried over anhydrous Na_2SO_4, and concentrated under reduced pressure. The product was purified by column chromatography on silica gel.

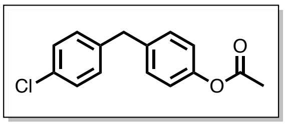

4-(4-chlorobenzyl)phenyl acetate (31a): Following the above procedure. The product was purified by column chromatography on silica gel (10:1, petroleum ether/ethyl acetate), colorless oil, 1.1 g, 85% yield.

^{1}H NMR (400 MHz, $CDCl_3$) δ 7.17 (d, *J* = 8.5 Hz, 2H), 7.06 (d, *J* = 8.5 Hz, 2H), 7.02 (d, *J* = 8.1 Hz, 2H), 6.92 (d, *J* = 8.6 Hz, 2H), 3.84 (s, 2H), 2.19 (s, 3H).

^{13}C NMR (101 MHz, $CDCl_3$) δ 169.61, 149.14, 139.23, 138.15, 132.06, 130.33, 129.80, 128.65, 121.64, 40.59, 21.15.

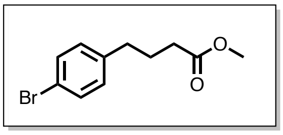

methyl 4-(4-bromophenyl)butanoate (34a): Following the above procedure. The product was purified by column chromatography on silica gel (10:1, petroleum ether/ethyl acetate), colorless oil, 1.2 g, 94% yield.

^{1}H NMR (400 MHz, $CDCl_3$) δ 7.40 (d, *J* = 8.4 Hz, 2H), 7.05 (d, *J* = 8.4 Hz, 2H), 3.66 (s, 3H), 2.60 (t, *J* = 7.6 Hz, 2H), 2.32 (t, *J* = 7.4 Hz, 2H), 1.93 (p, *J* = 7.5 Hz, 2H).

^{13}C NMR (101 MHz, $CDCl_3$) δ 173.73, 140.32, 131.45, 130.24, 119.76, 51.54, 34.50, 33.22, 26.29.

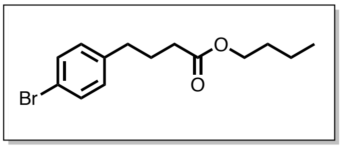

butyl 4-(4-bromophenyl)butanoate (35a): Following the above procedure. The product was purified by column chromatography on silica gel (10:1, petroleum ether/ethyl acetate), colorless oil, 1.4 g, 95% yield.

^{1}H NMR (400 MHz, $CDCl_3$) δ 7.40 (d, *J* = 8.4 Hz, 2H), 7.05 (d, *J* = 8.4 Hz, 2H), 4.07 (t, *J* = 6.7 Hz, 2H), 2.60 (t, *J* = 7.6 Hz, 2H), 2.30 (t, *J* = 7.4 Hz, 2H), 1.92 (p, *J* = 7.5 Hz, 2H), 1.74 – 1.53 (m, 2H), 1.53 – 1.28 (m, 2H), 0.93 (t, *J* = 7.4 Hz, 3H).

^{13}C NMR (101 MHz, $CDCl_3$) δ 173.39, 140.40, 131.45, 130.24, 119.74, 64.28, 34.53, 33.52, 30.70, 26.37, 19.15, 13.69.

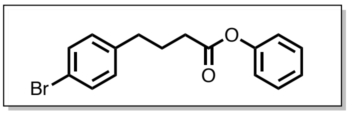

phenyl 4-(4-bromophenyl)butanoate (36a): Following the above procedure. The product was purified by column chromatography on silica gel (10:1, petroleum ether/ethyl acetate), colorless oil, 1.4 g, 90% yield.

^{1}H NMR (400 MHz, $CDCl_3$) δ 7.59 – 7.27 (m, 4H), 7.21 (t, *J* = 7.5 Hz, 1H), 7.13 – 6.86 (m, 4H), 2.68 (t, *J* = 7.6 Hz, 2H), 2.55 (t, *J* = 7.4 Hz, 2H), 2.04 (p, *J* = 7.5 Hz, 2H).

^{13}C NMR (101 MHz, $CDCl_3$) δ 171.78, 150.71, 140.19, 131.58, 130.33, 129.46, 125.84, 121.55, 119.93, 34.48, 33.53, 26.30.

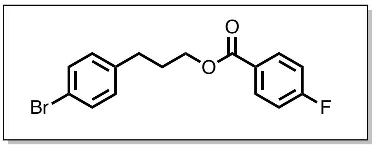

3-(4-bromophenyl)propyl 4-fluorobenzoate (37a): Following the above procedure. The product was purified by column chromatography on silica gel (10:1, petroleum ether/ethyl acetate), colorless oil, 1.6 g, 98% yield.

^{1}H NMR (400 MHz, $CDCl_3$) δ 8.00 (dd, *J* = 8.7, 5.6 Hz, 2H), 7.40 (d, *J* = 8.2 Hz, 2H), 7.18 – 7.00 (m, 4H), 4.32 (t, *J* = 6.4 Hz, 2H), 2.73 (t, *J* = 7.6 Hz, 2H), 2.31 – 1.88 (m, 2H).

^{13}C NMR (101 MHz, $CDCl_3$) δ 167.04, 165.58, 164.52, 140.13, 132.12, 132.03, 131.57, 130.18, 126.52, 126.49, 119.84, 115.63, 115.41, 64.28, 31.87, 30.09.

^{19}F NMR (376 MHz, $CDCl_3$) δ -105.56.

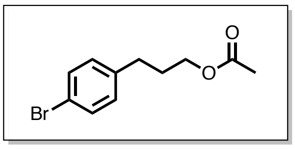

3-(4-bromophenyl)propyl acetate (38a): Following the above procedure. The product was purified by column chromatography on silica gel (10:1, petroleum ether/ethyl acetate), colorless oil, 1.2 g, 95% yield.

^{1}H NMR (400 MHz, $CDCl_3$) δ 7.52 – 7.33 (m, 2H), 7.14 – 6.88 (m, 2H), 4.07 (t, J = 6.5 Hz, 2H), 2.64 (dd, J = 8.7, 6.7 Hz, 2H), 2.05 (s, 3H), 2.01 – 1.83 (m, 2H).

^{13}C NMR (101 MHz, $CDCl_3$) δ 171.06, 140.16, 131.50, 130.15, 119.78, 63.58, 31.63, 30.03, 20.94.

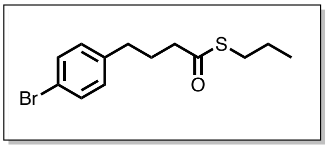

S-propyl 4-(4-bromophenyl)butanethioate (40a): Following the above procedure. The product was purified by column chromatography on silica gel (10:1, petroleum ether/ethyl acetate), colorless oil, 1.4 g, 90% yield.

^{1}H NMR (500 MHz, $CDCl_3$) δ 7.46 – 7.31 (m, 2H), 7.16 – 6.98 (m, 2H), 2.85 (t, J = 7.2 Hz, 2H), 2.60 (t, J = 7.6 Hz, 2H), 2.54 (t, J = 7.4 Hz, 2H), 1.96 (p, J = 7.5 Hz, 2H), 1.59 (h, J = 7.4 Hz, 2H), 0.96 (t, J = 7.3 Hz, 3H).

^{13}C NMR (101 MHz, $CDCl_3$) δ 199.23, 140.21, 131.48, 130.26, 119.82, 43.16, 34.31, 30.78, 26.99, 23.00, 13.35.

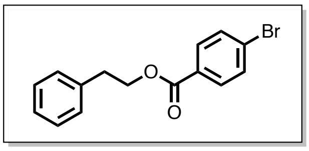

phenethyl 4-bromobenzoate (41a): Following the above procedure. The product was purified by column chromatography on silica gel (10:1, petroleum ether/ethyl acetate), colorless oil, 1.4 g, 90% yield.

^{1}H NMR (400 MHz, $CDCl_3$) δ 7.93 – 7.80 (m, 2H), 7.72 – 7.49 (m, 2H), 7.28 (tt, J = 14.1, 7.6 Hz, 5H), 4.52 (t, J = 6.9 Hz, 2H), 3.07 (t, J = 6.9 Hz, 2H).

^{13}C NMR (101 MHz, $CDCl_3$) δ 165.79, 137.75, 131.73, 131.11, 129.21, 128.95, 128.60, 128.05, 126.68, 65.71, 35.21.

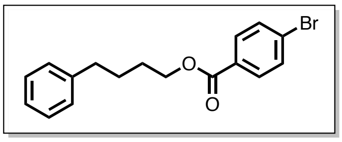

4-phenylbutyl 4-bromobenzoate (42a): Following the above procedure. The product was purified by column chromatography on silica gel (10:1, petroleum ether/ethyl acetate), colorless oil, 1.5 g, 92% yield.

^{1}H NMR (500 MHz, $CDCl_3$) δ 7.88 (d, *J* = 8.5 Hz, 2H), 7.65 – 7.49 (m, 2H), 7.28 (t, *J* = 7.5 Hz, 2H), 7.23 – 7.09 (m, 3H), 4.32 (t, *J* = 6.1 Hz, 2H), 2.68 (t, *J* = 7.0 Hz, 2H), 1.79 (qt, *J* = 6.0, 3.3 Hz, 4H).

^{13}C NMR (126 MHz, $CDCl_3$) δ 165.91, 141.96, 131.71, 131.13, 129.35, 128.44, 128.42, 127.98, 125.94, 65.14, 35.50, 28.30, 27.81.

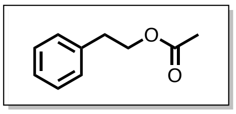

phenethyl acetate (52a): Following the above procedure. The product was purified by column chromatography on silica gel (10:1, petroleum ether/ethyl acetate), colorless oil, 0.8 g, 96% yield.

^{1}H NMR (400 MHz, $CDCl_3$) δ 7.36 – 7.27 (m, 2H), 7.27 – 7.13 (m, 3H), 4.28 (t, *J* = 7.1 Hz, 2H), 2.94 (t, *J* = 7.1 Hz, 2H), 2.03 (s, 3H).

^{13}C NMR (101 MHz, $CDCl_3$) δ 171.06, 137.84, 128.90, 128.51, 126.58, 64.95, 35.10, 20.99.

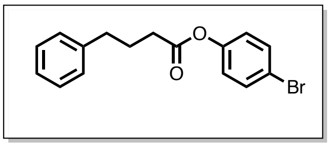

4-bromophenyl 4-phenylbutanoate (53a): Following the above procedure. The product was purified by column chromatography on silica gel (10:1, petroleum ether/ethyl acetate), colorless oil, 1.4 g, 90% yield.

^{1}H NMR (500 MHz, $CDCl_3$) δ 7.58 – 7.41 (m, 2H), 7.30 (dd, *J* = 8.3, 7.1 Hz, 2H), 7.21 (td, *J* = 6.4, 1.6 Hz, 3H), 6.99 – 6.92 (m, 2H), 2.73 (t, *J* = 7.6 Hz, 2H), 2.56 (t, *J* = 7.4 Hz, 2H), 2.07 (p, *J* = 7.5 Hz, 2H).

^{13}C NMR (126 MHz, $CDCl_3$) δ 171.68, 149.70, 141.07, 132.47, 128.53 (d, *J* = 2.8 Hz), 126.18, 123.38, 118.86, 35.05, 33.58, 26.38.

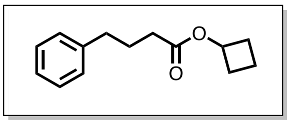

cyclobutyl 4-phenylbutanoate (55a): Following the above procedure. The product was purified by column chromatography on silica gel (10:1, petroleum ether/ethyl acetate), colorless oil, 0.9 g, 90% yield.

^{1}H NMR (500 MHz, $CDCl_3$) δ 7.28 (dd, J = 8.6, 6.5 Hz, 2H), 7.21 – 7.14 (m, 3H), 4.98 (p, J = 7.5 Hz, 1H), 2.64 (t, J = 7.6 Hz, 2H), 2.34 (dtt, J = 9.6, 4.4, 2.3 Hz, 2H), 2.29 (t, J = 7.5 Hz, 2H), 2.04 (dqt, J = 10.0, 7.9, 2.4 Hz, 2H), 1.94 (p, J = 7.5 Hz, 2H), 1.84 – 1.72 (m, 1H), 1.61 (ddt, J = 20.9, 10.4, 8.0 Hz, 1H).

^{13}C NMR (126 MHz, $CDCl_3$) δ 172.89, 141.47, 128.51, 128.40, 125.98, 68.65, 35.15, 33.65, 30.36, 26.54, 13.57.

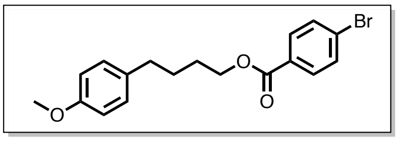

4-(4-methoxyphenyl)butyl 4-bromobenzoate (66a): Following the above procedure. The product was purified by column chromatography on silica gel (10:1, petroleum ether/ethyl acetate), colorless oil, 1.2 g, 65% yield.

^{1}H NMR (400 MHz, $CDCl_3$) δ 7.88 (d, J = 8.6 Hz, 2H), 7.57 (d, J = 8.5 Hz, 2H), 7.10 (d, J = 8.5 Hz, 2H), 6.83 (d, J = 8.6 Hz, 2H), 4.32 (t, J = 6.2 Hz, 2H), 3.79 (s, 3H), 2.63 (t, J = 7.1 Hz, 2H), 1.88 – 1.64 (m, 4H).

^{13}C NMR (126 MHz, $CDCl_3$) δ 165.92, 157.84, 134.02, 131.69, 131.09, 129.35, 129.27, 127.95, 113.81, 65.16, 55.27, 34.53, 28.21, 28.00.

The reduction of carbonyl[2]

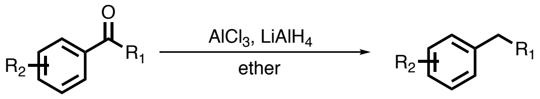

A flame-dried vial was charged with water free $AlCl_3$ (1180 mg, 8.84 mmol, 1.77 equiv.) and lithium aluminum hydride (210 mg, 5.00 mmol, 1.0 equiv.) under nitrogen atmosphere. The mixture was carefully suspended in dry ether (8 mL). Aromatic ketones (5.0 mmol, 1.0 equiv.) were carefully added as solid (violent reaction) to the suspension. The mixture was stirred for 3 h at room

temperature, diluted with ether (20 mL) and quenched by the addition of aqueous HCl (1 M). The phases where separated, and the aqueous phase was extracted with ether (3 x 10 mL). The organic phase was dried over sodium sulfate, filtered, and concentrated. The product was purified by column chromatography on silica gel.

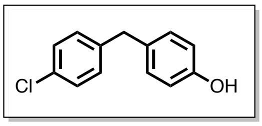

4-(4-chlorobenzyl)phenol (31b): Following the above procedure. The product was purified by column chromatography on silica gel (10:1, petroleum ether/ethyl acetate), yellow oil, 0.93 g, 85% yield.

^{1}H NMR (500 MHz, $CDCl_3$) δ 7.23 (d, J = 8.5 Hz, 2H), 7.08 (d, J = 8.2 Hz, 2H), 7.01 (d, J = 8.5 Hz, 2H), 6.75 (d, J = 8.5 Hz, 2H), 4.77 (s, 1H), 3.86 (s, 2H).

^{13}C NMR (101 MHz, $CDCl_3$) δ 153.95, 140.02, 132.90, 131.82, 130.17, 130.06, 128.56, 115.44, 40.35.

The synthesis of 32a[3]

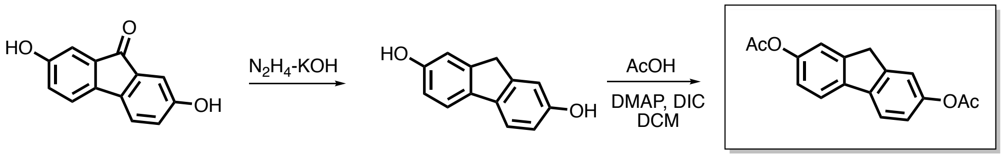

2,7-dihydroxy-9H-fluoren-9-one (2.5 mmol, 0.53 g) was dissolved in 10 mL of hot diethylene glycol. Hydrazine hydrate (85%, 3 mL) was added, and the mixture was refluxed at 120 °C for 1 hour. KOH (1.5 g) was then added, and the temperature was raised to 200-205 °C. Heating was continued without a condenser for 2 hours. The reaction mixture was then cooled, treated with 10 mL of cold water, and acidified with 3 mL concentrated HCl to precipitate crude product. The crude product did not require any other treatment and could be directly used for the next esterification reaction. To a solution of the crude product in CH_2Cl_2 (10 mL) was added AcOH (5 mmol, 2.0 equiv), followed by DIC (3 mmol, 1.2 equiv) and DMAP (0.5 mmol, 0.2 equiv). The resulting reaction mixture was stirred at room temperature for 12 h, then diluted with CH_2Cl_2, and quenched with 1N HCl. The layers were separated, and the aqueous layer was extracted twice with CH_2Cl_2. The combined organic solution was washed with brine, dried over anhydrous Na_2SO_4, and concentrated

under reduced pressure. The product was purified by column chromatography on silica gel.

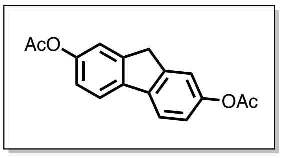

9H-fluorene-2,7-diyl diacetate (32a): Following the above procedure. The product was purified by column chromatography on silica gel (10:1-5:1, petroleum ether/ethyl acetate), white solid, 0.56 g, 80% yield.

^{1}H NMR (400 MHz, $CDCl_3$) δ 7.71 (d, J = 8.2 Hz, 2H), 7.26 (s, 2H), 7.08 (dd, J = 8.2, 2.2 Hz, 2H), 3.89 (s, 2H), 2.32 (s, 6H).

^{13}C NMR (101 MHz, $CDCl_3$) δ 169.81, 149.69, 144.61, 138.77, 120.31, 118.52, 36.95, 21.20.

The synthesis of 33a[4]

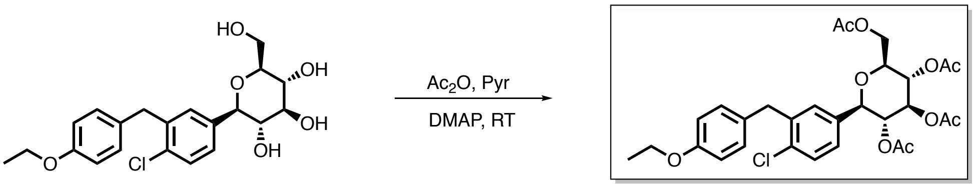

Dapaglifiozin (300 mg, 0.72 mmol) was dissolved in pyridine (4 mL), and then DMAP (15 mg) and Ac_2O (2.4 mL) were added slowly under an ice-water bath. After the completion of the addition, the mixture was stirred at room temperature for 2 h. A saturated aqueous $NaHCO_3$ solution was added to quench the reaction. The organic phase was separated. The aqueous phase was extracted with dichloromethane. The organic phases were combined, washed with water and a saturated aqueous NaCl solution, dried over with anhydrous Na_2SO_4, and concentrated. The residue was purified by a silica gel column chromatography to give desire product.

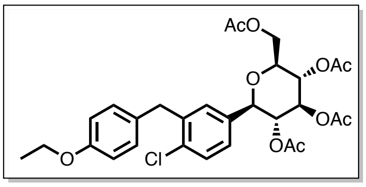

(2S,3S,4S,5R,6R)-2-(acetoxymethyl)-6-(4-chloro-3-(4-ethoxybenzyl)phenyl)tetrahydro-2H-pyran-3,4,5-triyl triacetate (33a): Following the above procedure. The product was purified by column chromatography on silica gel (10:1-5:1, petroleum ether/ethyl acetate), white solid, 0.34 g, 81% yield.

^{1}H NMR (400 MHz, $CDCl_3$) δ 7.35 (d, J = 8.2 Hz, 1H), 7.18 (dd, J = 8.3, 2.2 Hz, 1H), 7.11 – 6.99 (m, 3H), 6.82 (d, J = 8.6 Hz, 2H), 5.28 (t, J = 9.4 Hz, 1H), 5.20 (t, J = 9.6 Hz, 1H), 5.05 (t, J = 9.6 Hz, 1H), 4.36 – 4.22 (m, 2H), 4.14 (dd, J = 12.4, 2.3 Hz, 1H), 4.08 – 3.94 (m, 4H), 3.79 (ddd, J = 9.9, 4.8, 2.3 Hz, 1H), 2.05-2.07 (s, 3H), 1.99 (s, 3H), 1.71 (s, 3H), 1.40 (t, J = 7.0 Hz, 3H).

^{13}C NMR (101 MHz, $CDCl_3$) δ 170.68, 170.32, 169.47, 168.75, 157.54, 139.09, 135.15, 134.62, 131.07, 129.82, 125.99, 114.56, 79.53, 76.17, 74.17, 72.57, 68.55, 63.42, 62.31, 38.27, 20.74, 20.61, 20.28, 14.86.

The synthesis of 40a[5]

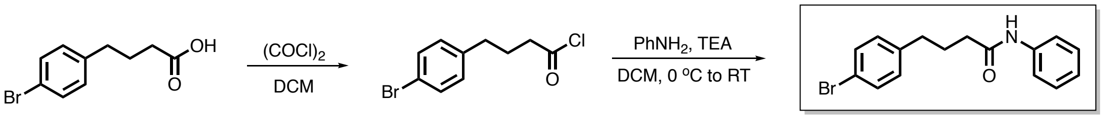

To a solution of 4-(4-bromophenyl)butanoic acid (5 mmol, 1.0 eq) and DMF (10 μL) in dry dichloromethane (20 mL) at 0 °C, oxalyl chloride (0.85 mL, 10 mmol) was slowly added to this solution and the reaction mixture was warmed up to room temperature and stirred for 2 hours. After concentration, the generated acid chloride 4-(4-bromophenyl)butanoyl chloride was directly used for next step without any further purification.

To a solution of the above synthesized 4-(4-bromophenyl)butanoyl chloride (5 mmol) in DCM (10.0 mL) at 0 °C, $PhNH_2$ (6 mmol, 1.2 eq) was added dropwise to the stirring reaction over 5 minutes. Then the reaction mixture was allowed to warm to room temperature and stirred for 2 hours. After addition of 15.0 mL DCM and 15.0 mL H_2O, the reaction mixture was extracted with DCM (3x15 mL). The combined organic layers were then dried over Na_2SO_4, and filtered. After concentration, the residue was purified by flash column chromatography on silica gel.

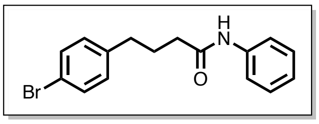

4-(4-bromophenyl)-N-phenylbutanamide (39a): Following the above procedure. The product was purified by column chromatography on silica gel (5:1, petroleum ether/ethyl acetate), yellow solid, 1.3 g, 84% yield.

^{1}H NMR (400 MHz, $CDCl_3$) δ 9.34 (s, 1H), 7.83 – 7.62 (m, 1H), 7.49 (d, J = 7.9 Hz, 2H), 7.42 – 7.38 (m, 2H), 7.32 (t, J = 7.9 Hz, 2H), 7.08 (d, J = 7.8 Hz, 2H), 2.67 (t, J = 7.5 Hz, 2H), 2.34 (t, J =

7.4 Hz, 2H), 2.05 (p, J = 7.4 Hz, 2H).

^{13}C NMR (126 MHz, $CDCl_3$) δ 170.61, 140.32, 137.78, 131.51, 130.28, 129.31, 129.04, 125.60, 124.32, 119.84, 119.75, 36.56, 34.44, 26.61.

The synthesis of 48a, 57a and 58a[6]

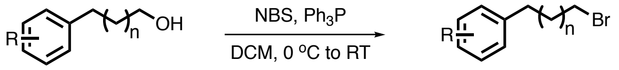

The corresponding alcohol (5 mmol, 1 eq.) was dissolved in anhydrous dichloromethane (75 mL) at argon atmosphere and the solution was chilled to 0 °C. While stirring, triphenylphosphine (6 mmol, 1.2 eq.) and N-bromosuccinimide (6 mmol, 1.2 eq.) were added and the mixture was stirred for additional 60 minutes. A saturated aqueous solution of $NaHCO_3$ (75 mL) was added and the mixture was extracted by dichloromethane (3 x 50 mL). The combined organic fractions were dried over $MgSO_4$ and the solvent was evaporated. The crude product was purified by flash column chromatography.

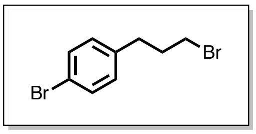

1-bromo-4-(3-bromopropyl)benzene (48a): Following the above procedure. The product was purified by column chromatography on silica gel (petroleum ether), colorless oil, 1.1 g, 80% yield.

^{1}H NMR (400 MHz, $CDCl_3$) δ 7.41 (d, J = 8.4 Hz, 2H), 7.07 (d, J = 8.4 Hz, 2H), 3.37 (t, J = 6.5 Hz, 2H), 2.74 (t, J = 7.4 Hz, 2H), 2.13 (dq, J = 8.0, 6.6 Hz, 2H).

^{13}C NMR (101 MHz, $CDCl_3$) δ 139.49, 131.59, 130.32, 119.98, 33.89, 33.35, 32.74.

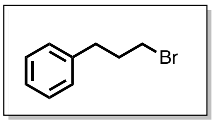

(3-bromopropyl)benzene (57a): Following the above procedure. The product was purified by column chromatography on silica gel (petroleum ether), colorless oil, 0.81 g, 82% yield.

^{1}H NMR (500 MHz, $CDCl_3$) δ 7.29 (dd, J = 8.5, 6.6 Hz, 2H), 7.23 – 7.12 (m, 3H), 3.38 (t, J = 6.6 Hz, 2H), 2.77 (t, J = 7.3 Hz, 2H), 2.25 – 2.08 (m, 2H).

^{13}C NMR (126 MHz, $CDCl_3$) δ 140.58, 128.59, 128.54, 126.20, 34.21, 34.02, 33.14.

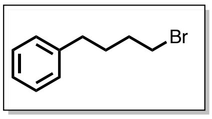

(4-bromobutyl)benzene (58a): Following the above procedure. The product was purified by column chromatography on silica gel (petroleum ether), colorless oil, 0.95 g, 90% yield.

^{1}H NMR (400 MHz, $CDCl_3$) δ 7.42 – 7.25 (m, 2H), 7.25 – 7.05 (m, 3H), 3.42 (td, *J* = 6.8, 2.8 Hz, 2H), 2.65 (td, *J* = 7.6, 3.1 Hz, 2H), 1.90 (qd, *J* = 6.9, 6.4, 3.3 Hz, 2H), 1.85 – 1.72 (m, 2H).

^{13}C NMR (126 MHz, $CDCl_3$) δ 141.81, 128.40, 125.92, 34.99, 33.66, 32.25, 29.86.

The synthesis of 47a[7]

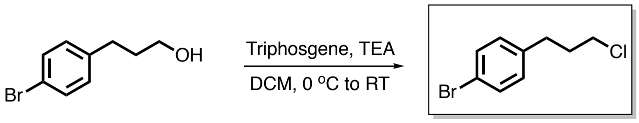

The corresponding alcohol (3 mmol, 1 eq.) was dissolved in anhydrous dichloromethane (30 mL) at argon atmosphere and the solution was chilled to 0 °C. While stirring, triphosgene (1.5 mmol, 0.5 eq.) and triethylamine (7.5 mmol, 2.5 eq.) were added and the mixture was stirred for additional 60 minutes. A saturated aqueous solution of $NaHCO_3$ (30 mL) was added and the mixture was extracted by dichloromethane (3 x 50 mL). The combined organic fractions were dried over Na_2SO_4 and the solvent was evaporated. The crude product was purified by flash column chromatography.

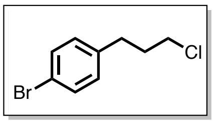

1-bromo-4-(3-chloropropyl)benzene (47a): Following the above procedure. The product was purified by column chromatography on silica gel (petroleum ether), colorless oil, 0.63 g, 90% yield.

^{1}H NMR (400 MHz, $CDCl_3$) δ 7.41 (d, *J* = 8.4 Hz, 2H), 7.21 – 6.94 (m, 2H), 3.51 (t, *J* = 6.4 Hz, 2H), 2.74 (t, *J* = 7.4 Hz, 2H), 2.33 – 1.94 (m, 2H).

^{13}C NMR (126 MHz, $CDCl_3$) δ 139.63, 131.57, 130.31, 119.93, 43.97, 33.77, 32.12.

The synthesis of 49a[8]

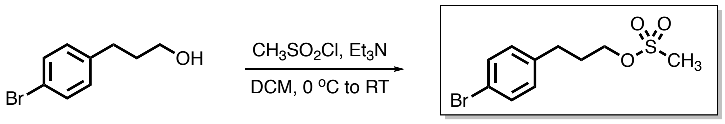

To a solution of alcohol (750 mg, 5 mmol) and triethylamine (1.38 mL, 10 mmol) in dry dichloromethane (15.0 mL) at 0 °C, methanesulfonyl chloride (464 μL, 6 mmol) was slowly added

to this solution and the reaction mixture was warmed up to room temperature and stirred for 2 hours. After the reaction was complete, water (15.0 mL) were added added to the reaction mixture. The organic layer was separated, and the aqueous layer was extracted with dichloromethane (3×15.0 mL) three times. The combined organic layers were washed with brine and dried over Na_2SO_4. After concentration, the residue was purified by flash column chromatography on silica gel.

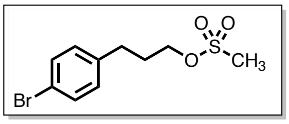

3-(4-bromophenyl)propyl methanesulfonate (49a): Following the above procedure. The product was purified by column chromatography on silica gel (petroleum ether), colorless oil, 1.3 g, 90% yield.

^{1}H NMR (500 MHz, $CDCl_3$) δ 7.42 (d, J = 8.3 Hz, 2H), 7.07 (d, J = 8.3 Hz, 2H), 4.21 (t, J = 6.3 Hz, 2H), 3.00 (s, 3H), 2.71 (t, J = 7.6 Hz, 2H), 2.14 – 1.98 (m, 2H).

^{13}C NMR (126 MHz, $CDCl_3$) δ 139.28, 131.67, 130.23, 120.10, 68.82, 37.41, 30.99, 30.53.

The synthesis of 44a[9,10]

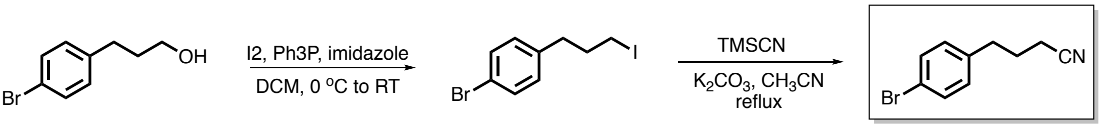

Alcohol (5 mmol, 1 equiv), triphenylphosphine (6 mmol, 1.2 equiv) and imidazole (6 mmol, 1.2 equiv) was added to an oven-dried flask and dichloromethane (50 mL) added under argon. The reaction mixture was stirred in an ice bath and iodine (6 mmol, 1.2 equiv) was added portionwise. The reaction was stirred, warming to room temperature. After the specified reaction time, the reaction mixture was filtered through a silica plug, washing with diethyl ether. The filtrate was concentrated in vacuo and purified by flash chromatography to afford the desired iodide.

The product iodide (2 mmol, 1.0 eq)was added a CH_3CN solution of Me_3SiCN (2.4 mmol, 1.2 eq) and K_2CO_3 (2.4 mmol, 1.2 eq).The resultant mixture was further heated at 80 ºC for 24 hours. The reaction was quenched with aq Na_2CO_3 (10 mL). The aqueous layer was extracted with EtOAc (3*15 mL), and the combined organic phases were dried by Na_2SO_4, then filtered, and evaporated under reduced pressure. The crude product was purified by flash chromatography to afford the

desired cyanide.

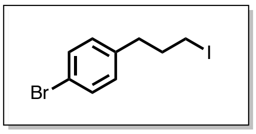

1-bromo-4-(3-iodopropyl)benzene (44b): Following the above procedure. The product was purified by column chromatography on silica gel (petroleum ether), colorless oil, 1.3 g, 84% yield.

^{1}H NMR (400 MHz, $CDCl_3$) δ 7.41 (d, J = 8.1 Hz, 2H), 7.07 (d, J = 8.1 Hz, 2H), 3.15 (t, J = 6.7 Hz, 2H), 2.69 (t, J = 7.3 Hz, 2H), 2.09 (p, J = 7.0 Hz, 2H).

^{13}C NMR (126 MHz, $CDCl_3$) δ 139.34, 131.58, 130.33, 119.98, 35.58, 34.57, 5.94.

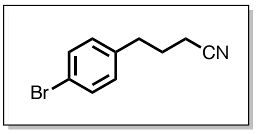

4-(4-bromophenyl)butanenitrile (44a): Following the above procedure. The product was purified by column chromatography on silica gel (10:1-5:1, petroleum ether/ethyl acetate), yellow oil, 0.16 g, 35% yield.

^{1}H NMR (400 MHz, $CDCl_3$) δ 7.59 – 7.31 (m, 2H), 7.06 (d, J = 8.2 Hz, 2H), 2.74 (t, J = 7.5 Hz, 2H), 2.32 (t, J = 7.0 Hz, 2H), 1.95 (p, J = 7.2 Hz, 2H).

^{13}C NMR (126 MHz, $CDCl_3$) δ 138.68, 131.77, 130.21, 120.35, 119.28, 33.77, 26.72, 16.39.

The synthesis of ether[11]

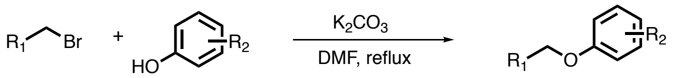

Potassium carbonate (3 mmol, 1.5 eq) was added to a solution of phenol (2.4 mmol, 1.2 eq) and bromide compounds (2 mmol, 1.0 eq) in DMF (10 ml). The resulting suspension was stirred at 80 ºC for 16 h. At this point, the reaction was quenched with water (10 mL) and extracted with EtOAc (3 x 15 mL). The combined organic phases were washed again with water (10 mL), brine solution (10 mL) and dried over anhydrous sodium sulfate, filtered and concentrated using rotary evaporator. The crude product was then purified by column chromatography to afford the desired product.

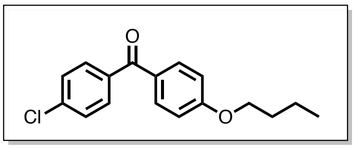

(4-butoxyphenyl)(4-chlorophenyl)methanone (30a): Following the above procedure. The product

was purified by column chromatography on silica gel (20:1, petroleum ether/ethyl acetate), white solid, 0.41 g, 71% yield.

^{1}H NMR (400 MHz, $CDCl_3$) δ 7.78 (d, J = 8.8 Hz, 2H), 7.71 (d, J = 8.5 Hz, 2H), 7.45 (d, J = 8.5 Hz, 2H), 6.95 (d, J = 8.8 Hz, 2H), 4.05 (t, J = 6.5 Hz, 2H), 1.81 (dq, J = 8.5, 6.6 Hz, 2H), 1.56 – 1.47 (m, 2H), 0.99 (t, J = 7.4 Hz, 3H).

^{13}C NMR (101 MHz, $CDCl_3$) δ 194.30, 163.08, 138.22, 136.68, 132.46, 131.14, 129.57, 128.51, 114.16, 68.04, 31.14, 19.19, 13.79.

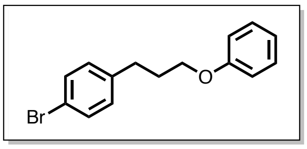

1-bromo-4-(3-phenoxypropyl)benzene (50a): Following the above procedure. The product was purified by column chromatography on silica gel (50:1, petroleum ether/ethyl acetate), colorless oil, 0.35 g, 60% yield.

^{1}H NMR (400 MHz, $CDCl_3$) δ 7.39 (d, J = 8.3 Hz, 2H), 7.27 (dd, J = 8.7, 7.3 Hz, 2H), 7.08 (d, J = 8.3 Hz, 2H), 6.99 – 6.80 (m, 3H), 3.93 (t, J = 6.2 Hz, 2H), 2.76 (dd, J = 8.4, 6.8 Hz, 2H), 2.34 – 1.97 (m, 2H).

^{13}C NMR (101 MHz, $CDCl_3$) δ 158.94, 140.52, 131.49, 130.34, 129.50, 120.72, 119.71, 114.53, 66.49, 31.63, 30.74.

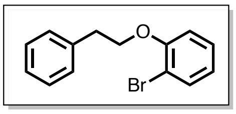

1-bromo-2-phenethoxybenzene (54a): Following the above procedure. The product was purified by column chromatography on silica gel (50:1, petroleum ether/ethyl acetate), colorless oil, 0.19 g, 35% yield.

^{1}H NMR (400 MHz, $CDCl_3$) δ 7.52 (dd, J = 7.8, 1.6 Hz, 1H), 7.43 – 7.27 (m, 4H), 7.27 – 7.14 (m, 2H), 6.93 – 6.74 (m, 2H), 4.20 (t, J = 6.9 Hz, 2H), 3.15 (t, J = 6.9 Hz, 2H)

^{13}C NMR (101 MHz, $CDCl_3$) δ 155.25, 138.11, 133.42, 129.30, 128.51, 128.43, 126.62, 121.87, 113.15, 112.26, 69.89, 35.80.

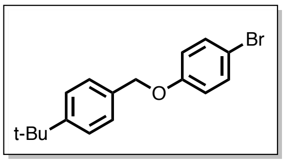

1-bromo-4-((4-(tert-butyl)benzyl)oxy)benzene (60a): Following the above procedure. The product was purified by column chromatography on silica gel (50:1, petroleum ether/ethyl acetate), white solid, 0.34 g, 54% yield.

^{1}H NMR (400 MHz, $CDCl_3$) δ 7.41 (d, J = 8.5 Hz, 2H), 7.39 – 7.33 (m, 4H), 6.85 (d, J = 9.0 Hz, 2H), 4.99 (s, 2H), 1.32 (s, 9H).

^{13}C NMR (126 MHz, $CDCl_3$) δ 167.29, 149.40, 134.43, 133.23 – 131.21 (m), 127.40 (d, J = 4.8 Hz), 124.24, 26.20, 22.37.

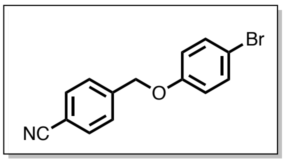

4-((4-bromophenoxy)methyl)benzonitrile (61a): Following the above procedure. The product was purified by column chromatography on silica gel (20:1, petroleum ether/ethyl acetate), white solid, 0.37 g, 65% yield.

1H NMR (400 MHz, $CDCl_3$) δ 7.67 (dd, J = 8.2, 1.6 Hz, 2H), 7.52 (d, J = 8.0 Hz, 2H), 7.47 – 7.34 (m, 2H), 6.98 – 6.70 (m, 2H), 5.09 (s, 2H).

^{13}C NMR (126 MHz, $CDCl_3$) δ 157.27, 142.00, 132.48 (d, J = 2.4 Hz), 127.55, 118.62, 116.61, 113.75, 111.90, 69.11.

The synthesis of 64a

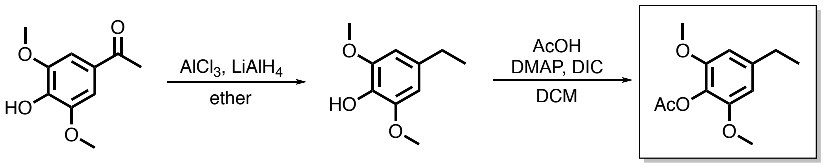

A flame-dried vial was charged with water free $AlCl_3$ (1180 mg, 8.84 mmol, 1.77 equiv.) and lithium aluminum hydride (210 mg, 5.00 mmol, 1.0 equiv.) under nitrogen atmosphere. The mixture was carefully suspended in dry ether (8 mL). Acetosyringone (5.0 mmol, 1.0 equiv.) were carefully added as solid (violent reaction) to the suspension. The mixture was stirred for 3 h at room temperature, diluted with ether (20 mL) and quenched by the addition of aqueous HCl (1 M). The phases where separated, and the aqueous phase was extracted with ether (3 x 10 mL). The organic phase was dried over sodium sulfate, filtered, and concentrated. After concentration, the reduction

product was directly used for next step without any further purification.

To a solution of AcOH (5 mmol, 1.0 equiv) in CH_2Cl_2 (20 mL) was added the previous crude product, followed by DIC (6 mmol, 1.2 equiv) and DMAP (1 mmol, 0.2 equiv). The resulting reaction mixture was stirred at room temperature for 12 h, then diluted with CH_2Cl_2, and quenched with 1N HCl. The layers were separated, and the aqueous layer was extracted twice with CH_2Cl_2. The combined organic solution was washed with brine, dried over anhydrous Na_2SO_4, and concentrated under reduced pressure. The product was purified by column chromatography on silica gel.

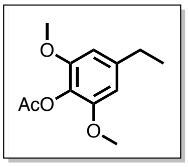

4-ethyl-2,6-dimethoxyphenyl acetate (64a): Following the above procedure. The product was purified by column chromatography on silica gel (10:1, petroleum ether/ethyl acetate), white solid, 0.51 g, 45% yield.

^{1}H NMR (500 MHz, $CDCl_3$) δ 6.45 (s, 2H), 3.81 (s, 6H), 2.62 (q, J = 7.6 Hz, 2H), 2.33 (s, 3H), 1.25 (t, J = 7.6 Hz, 3H).

^{13}C NMR (126 MHz, $CDCl_3$) δ 196.82, 168.22, 152.26, 135.16, 132.88, 105.15, 56.35, 26.55, 20.45.

5. The unsuccessful substrates

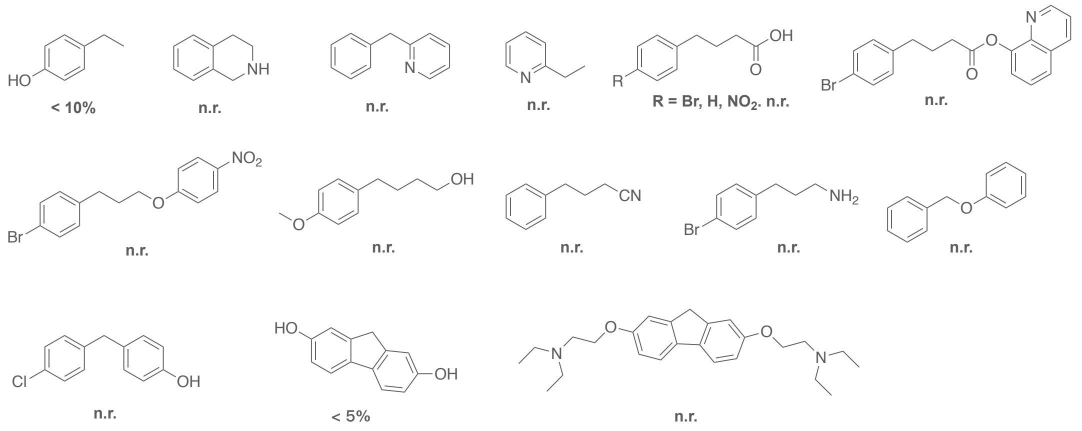

6. The benzylic C-H bonds dissociation enthalpies

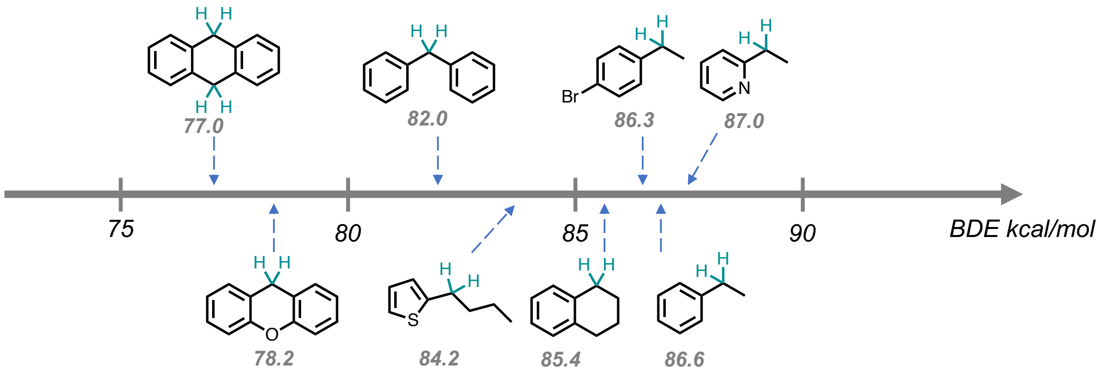

Figure S2. BDE energies (kcal/mol) of benzylic C-H bonds. The data were obtained using web-based computational tool ALFABET (https://bde.ml.nrel.gov/).[1]

7. The by-product of the oxidation reaction

The α-site oxidation of aromatic alkyl ether

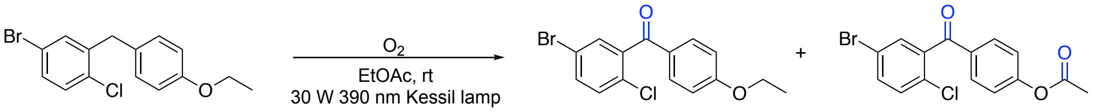

29a **29,** 54% **29b,** 21%

The peroxide in the oxidation reaction

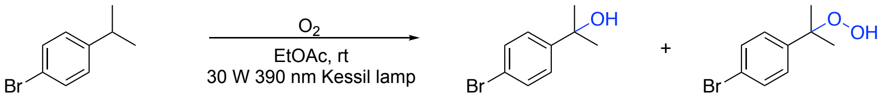

52a **52,** 50% **52b,** 25%

8. Gram-scale reaction

The gram-scale synthesis in solvent

The synthesis of 2

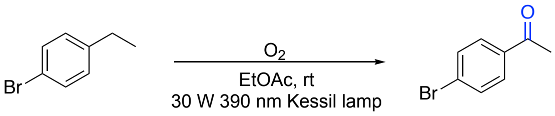

1, 5 mmol **2,** 72%

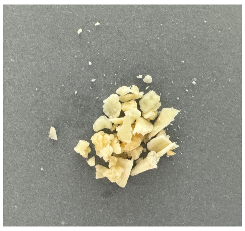

The oven-dried flask (25 mL) containing a stirring bar was charged with **1** (5 mmol) and EtOAc (15 mL). The flask was then evacuated and back-filled with O_2 for 3 times. The flask was stirring and irradiated with 30 W 390 nm Kessil lamp for 12 hours. The cooling fan was used for heat dissipation. The progress of the reaction was monitored by TLC. After completion of the reaction, the mixture was concentrated in *vacuo*. The residue was purified by silica gel flash column chromatography (10:1, petroleum ether/ethyl acetate) to give the pure desired product **2** (0.71g, 72%, white solid).

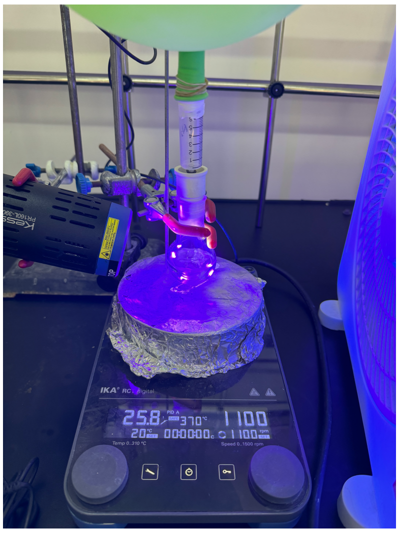

The synthesis of 43

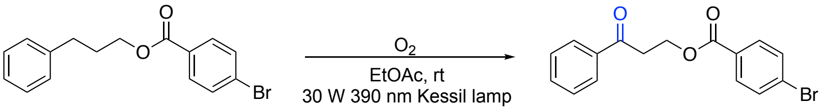

42a, 2 mmol **42,** 64%

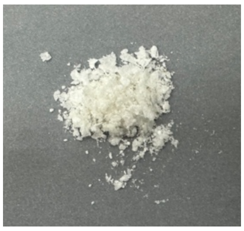

The oven-dried flask (25 mL) containing a stirring bar was charged with **42a** (2 mmol) and EtOAc (6 mL). The flask was then evacuated and back-filled with O_2 for 3 times. The flask was stirring and irradiated with 30 W 390 nm Kessil lamp for 12 hours. The cooling fan was used for heat

dissipation. The progress of the reaction was monitored by TLC. After completion of the reaction, the mixture was concentrated in *vacuo*. The residue was purified by silica gel flash column chromatography (10:1, petroleum ether/ethyl acetate) to give the pure desired product **42** (0.44g, 64%, white solid).

9. General procedure for continuous-flow oxidation

9.1 Continuous-flow setup

The photo reactions were performed in a homemade continuous-flow reactor as shown in Figure S3. The feed solution was conveyed to the photoreactor with a plunger pump (OU SHISHENG, DP-C10, 0.1-10 mL min^{-1}). The liquid was connected to an oxygen line with a Bronkhorst®F210CTM mass flow controller (MFC) via a T-mixer and the 4 mL flow reactor, including a mixing tube (0.9 mL, 40 cm PFA tubing, inner diameter 2.0 mm) filled with glass beads (diameter 0.8-1.0 mm) and a photoreaction tube (4 mL PFA capillary tubing, inner diameter 1.0 mm) coiled around 50 mL quartz cup. The mixing tube was designed to enhance the efficiency of gas-liquid mixing. Two kessil lamps were placed in different locations next to the photoreaction tube.

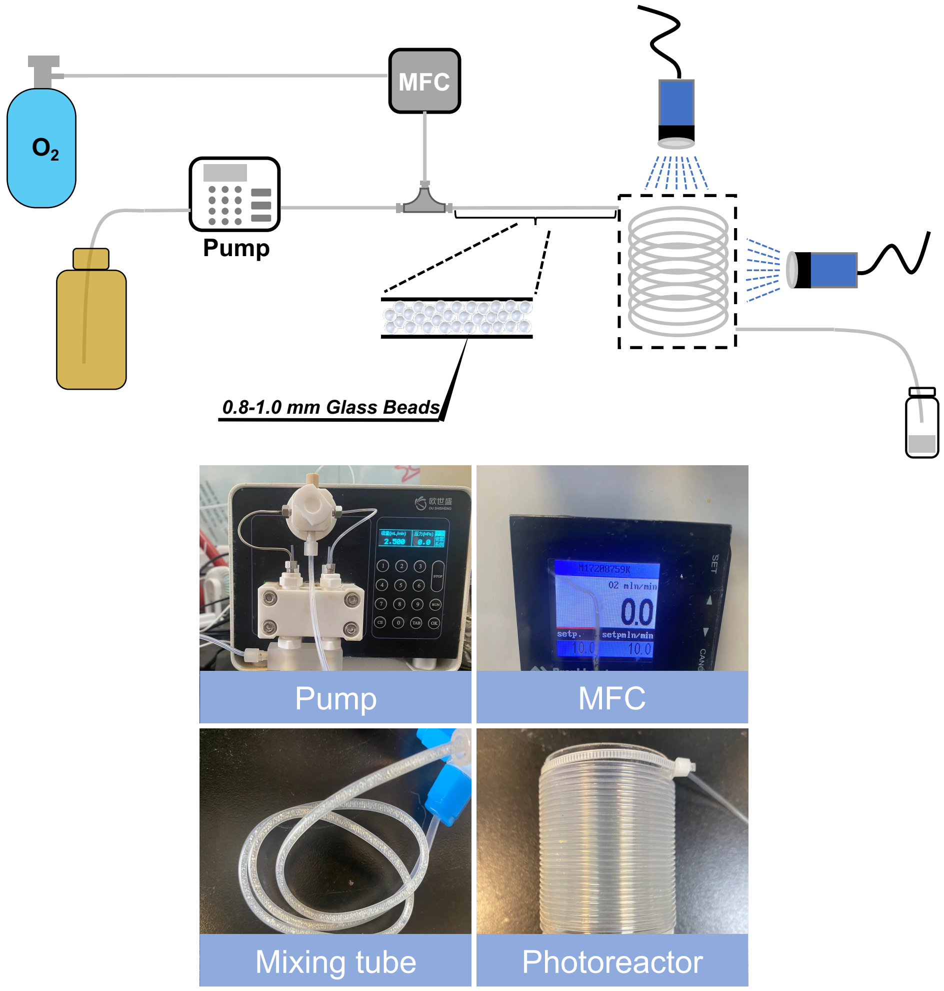

Figure S3. Continuous-flow setup.

9.2 Residence time calculation[13]

The residence time is calculated according to Equation S1:

$$Residence\ time\ (min) = \frac{Internal\ volume\ (mL)}{Flow\ rate\ (mL\ min^{-1})}\ \text{(Equation S1)}$$

The total flow rate combines the individual flow rates of all fluids fed into the reactor. The gas flow rate is calculated from the flow rate measured by the MFC according to Equation S2-3:

$$n_{O_2} = \frac{P_N(atm)V_N(L)}{R(L.atm.mol^{-1}.K^{-1})T_N(K)}\ \text{(Equation S2)}$$

$$V_{real} = \frac{n_{O_2}RT_{real}}{P_{real}}\ \text{(Equation S3)}$$

For example, the actual volume of O_2 delivered under 1 barg and 40 °C when the MFC is set at 10 mL min^{-1} is 78.75 min (see Equation S4-5):

$$n_{O_2} = \frac{P_N(atm)V_N(L)}{R(L.atm.mol^{-1}.K^{-1})T_N(K)} = \frac{1*0.01}{0.082*273.15} = 0.446mmol\ \text{(Equation S4)}$$

$$V_{real} = \frac{n_{O_2}RT_{real}}{P_{real}} = \frac{0.000446*0.082*313.15}{1} = 11.5\ mL\ \text{(Equation S5)}$$

Residence (irradiation) time within the reactor time was calculated according to Equation S1:

$$Residence\ time = \frac{Internal\ volume\ (mL)}{Flow\ rate\ liquid\ phase\ \left(\frac{mL}{min}\right) + Real\ flow\ rate\ gas\ phase\ \left(\frac{mL}{\min}\right)}$$

$$= \frac{4.9\ mL}{2.5\ mL\ min^{-1} + 11.5\ mL\ min^{-1}} = 0.35\ min$$

$$Total\ residence\ time = \frac{total\ time}{\frac{V_{mixture}}{flow}rate\ liquid\ phase} \times Residence\ time$$

$$= \frac{360\ min}{\frac{4\ mL}{2.5\ mL\ min^{-1}}} \times 0.35\ min = 78.75\ min$$

9.3 Optimization of continuous-flow conditions

Table S7. Condition screening of continuous-flow[a]

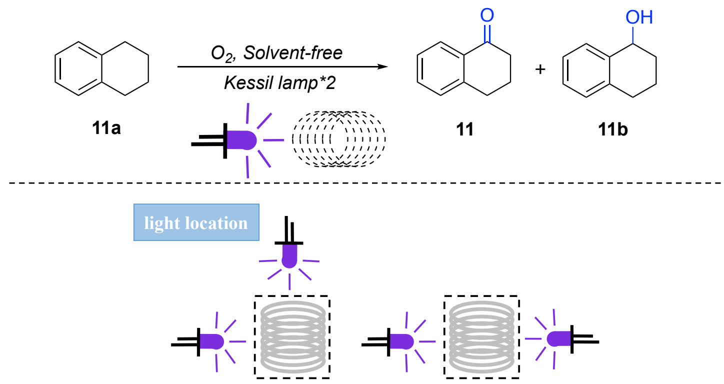

Entry	Solvent	V_l (mL min^{-1})	V_g	T (ºC)	light resource	light location	Total time	Residence time [min]	Yield of **11** [%][b]	Selectiity [%][b]
1	/	2.5	10	40	390 nm	a	6 h	78.8	50	82
2	/	2.5	20	40	390 nm	a	6 h	43.7	46	80
3	/	2.5	30	40	390 nm	a	6 h	30.4	43	79
4	/	5	40	40	390 nm	a	6 h	22.5	51	91
5	/	2.5	10	40	390 nm	a	8 h	105.0	56	84
6	/	2.5	10	40	370 nm	a	6 h	78.8	54	82
7	/	2.5	10	40	370 nm	b	6 h	78.8	62 (56[f])	86
8	H_2O	2.5	10	40	370 nm	a	6 h	63.0	44	85
9[c]	H_2O	2.5	10	40	370 nm	a	6 h	45.0	30	83
10[d]	H_2O	2.5	10	40	370 nm	a	6 h	229.5	31	86
11	CH_3OH	2.5	10	40	370 nm	a	6 h	63.0	51	80
12	DMSO	2.5	10	40	370 nm	a	4 h	63.0	33	98
13	Acetone	2.5	10	40	370 nm	a	6 h	63.0	43	94
14[e]	DMSO:H_2O	2.5	10	40	370 nm	a	6 h	63.0	43	97

[a]Reaction conditions: tetrahydronaphthalene (11, 4 mL, 30 mmol), Solvents (1.0 mL), the pump used to deliver the liquid phase and the rate of gas phase controlled by the MFC, without back pressure. [b]Yield and selectivity were determined by ^{1}H NMR with the internal standard CH_3NO_2. [c]3 mL H_2O. [d]5 bar back pressure. [e]DMSO:H_2O=2:1. [f]Isolated yield.

E-factor (E) calculation

According to the Table S8, entry 7, we calculated the E value as follow:

E = [3.96 g (**11a**) + 15.0 g (SiO_2 for purification) – 2.45 g (**11**)] / 2.45 g = 6.7

Note: In the flow synthesis of **11a**, only substrate **11a** was in the system and there were no any solvent. The eluents (petroleum ether and ethyl acetate) were recovered and reused.

10.Characteriation data of the synthesized compounds

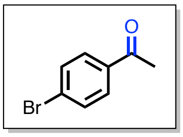

1-(4-bromophenyl)ethan-1-one (2)[20]: The product was purified by column chromatography on silica gel (10:1, petroleum ether/ethyl acetate), white solid, 79.6 mg, 80% yield.

^{1}H NMR (400 MHz, $CDCl_3$) δ 8.05 – 7.71 (m, 2H), 7.71 – 7.45 (m, 2H), 2.58 (s, 3H).

^{13}C NMR (101 MHz, $CDCl_3$) δ 196.98, 135.85, 131.90, 129.84, 128.30, 26.52.

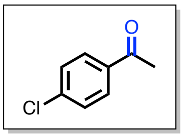

1-(4-chlorophenyl)ethan-1-one (3)[20]: The product was purified by column chromatography on silica gel (10:1, petroleum ether/ethyl acetate), white solid, 49.3 mg, 64% yield

^{1}H NMR (400 MHz, $CDCl_3$) δ 7.89 (dt, J = 8.4, 1.5 Hz, 2H), 7.42 (dt, J = 8.5, 1.6 Hz, 2H), 2.58 (s, 3H).

^{13}C NMR (126 MHz, $CDCl_3$) δ 196.73, 139.52, 135.44, 129.70, 128.85, 26.50, 26.48.

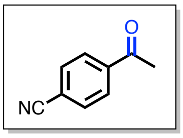

4-acetylbenzonitrile (4)[20]: The product was purified by column chromatography on silica gel (10:1, petroleum ether/ethyl acetate), white solid, 25.4 mg, 35% yield.

^{1}H NMR (400 MHz, $CDCl_3$) δ 8.16 – 7.97 (m, 2H), 7.86 – 7.63 (m, 2H), 2.65 (s, 3H).

^{13}C NMR (101 MHz, $CDCl_3$) δ 196.55, 139.94, 132.54, 128.72, 117.94, 116.44, 26.78.

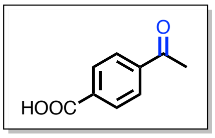

4-acetylbenzoic acid (5)[22]: The product was purified by column chromatography on silica gel (10:1, petroleum ether/ethyl acetate), white solid, 23.0 mg, 28% yield.

^{1}H NMR (400 MHz, DMSO-d_6) δ 13.31 (s, 1H), 8.06 (s, 4H), 2.63 (s, 3H).

^{13}C NMR (101 MHz, DMSO-d_6) δ 198.22, 167.12, 140.31, 134.98, 130.02, 128.80, 27.49.

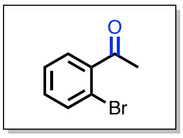

1-(2-bromophenyl)ethan-1-one (6)[20]:. The product was purified by column chromatography on silica gel (10:1, petroleum ether/ethyl acetate), pale yellow liquid, 59.7 mg, 60% yield.

^{1}H NMR (400 MHz, $CDCl_3$) δ 7.62 (dd, J = 7.9, 1.2 Hz, 1H), 7.47 (dd, J = 7.6, 1.8 Hz, 1H), 7.37 (td, J = 7.5, 1.2 Hz, 1H), 7.33 – 7.28 (m, 1H), 2.64 (s, 3H).

^{13}C NMR (101 MHz, $CDCl_3$) δ 201.44, 141.49, 133.87, 131.82, 128.94, 127.46, 118.93, 30.36.

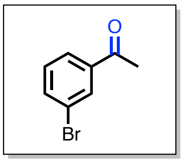

1-(2-bromophenyl)ethan-1-one (7)[20]: Following the general procedure A. The product was purified by column chromatography on silica gel (10:1, petroleum ether/ethyl acetate), pale yellow liquid, 59.7 mg, 60% yield.

^{1}H NMR (400 MHz, $CDCl_3$) δ 8.09 (t, J = 1.8 Hz, 1H), 7.93 – 7.83 (m, 1H), 7.69 (ddd, J = 8.0, 2.0, 1.0 Hz, 1H), 7.35 (t, J = 7.9 Hz, 1H), 2.60 (s, 3H).

^{13}C NMR (101 MHz, $CDCl_3$) δ 196.65, 138.81, 135.98, 131.40, 130.21, 126.86, 122.97, 26.63.

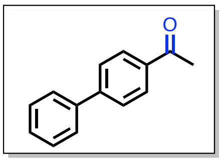

1-([1,1'-biphenyl]-4-yl)ethan-1-one (8)[20]: The product was purified by column chromatography on silica gel (10:1, petroleum ether/ethyl acetate), white solid, 77.4 mg, 79% yield.

^{1}H NMR (400 MHz, $CDCl_3$) δ 8.12 – 7.97 (m, 2H), 7.74 – 7.67 (m, 2H), 7.63 (dt, J = 6.3, 1.3 Hz, 2H), 7.54 – 7.45 (m, 2H), 7.44 – 7.33 (m, 1H), 2.64 (s, 3H).

^{13}C NMR (101 MHz, $CDCl_3$) δ 197.79, 145.81, 139.90, 135.89, 128.97, 128.93, 128.25, 127.29, 127.24, 26.66.

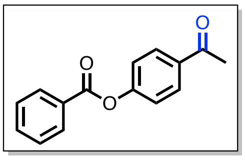

4-acetylphenyl benzoate (9)[23]: The product was purified by column chromatography on silica gel

(10:1, petroleum ether/ethyl acetate), white solid, 84.0 mg, 70% yield.

^{1}H NMR (400 MHz, $CDCl_3$) δ 8.21 (dd, *J* = 8.3, 1.4 Hz, 2H), 8.05 (d, *J* = 8.7 Hz, 2H), 7.72 – 7.59 (m, 1H), 7.53 (t, *J* = 7.8 Hz, 2H), 7.34 (d, *J* = 8.7 Hz, 2H), 2.63 (s, 3H).

^{13}C NMR (101 MHz, $CDCl_3$) δ 196.95, 164.67, 154.71, 134.81, 133.97, 130.29, 130.05, 129.05, 128.72, 121.97, 26.68.

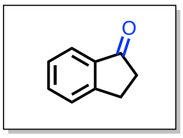

2,3-dihydro-1H-inden-1-one (10)[21]: The product was purified by column chromatography on silica gel (10:1, petroleum ether/ethyl acetate), yellow liquid, 40.9 mg, 62% yield.

^{1}H NMR (400 MHz, $CDCl_3$) δ 7.77 (dt, *J* = 7.7, 1.0 Hz, 1H), 7.59 (td, *J* = 7.5, 1.2 Hz, 1H), 7.48 (dp, *J* = 7.7, 1.0 Hz, 1H), 7.37 (ddt, *J* = 7.8, 7.1, 0.9 Hz, 1H), 3.22 – 3.12 (m, 2H), 2.75 – 2.66 (m, 2H).

^{13}C NMR (101 MHz, $CDCl_3$) δ 207.12, 155.18, 137.11, 134.61, 127.30, 126.71, 123.75, 36.24, 25.82.

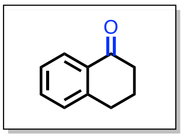

3,4-dihydronaphthalen-1(2H)-one (11)[21]: The product was purified by column chromatography on silica gel (10:1, petroleum ether/ethyl acetate), yellow liquid, 43.8 mg, 60% yield.

^{1}H NMR (400 MHz, $CDCl_3$) δ 8.04 (dd, *J* = 7.9, 1.5 Hz, 1H), 7.47 (td, *J* = 7.5, 1.5 Hz, 1H), 7.37 – 7.21 (m, 2H), 2.97 (t, *J* = 6.1 Hz, 2H), 2.66 (dd, *J* = 7.3, 5.8 Hz, 2H), 2.14 (p, *J* = 6.5 Hz, 2H).

^{13}C NMR (101 MHz, $CDCl_3$) δ 198.40, 144.50, 133.40, 132.65, 128.77, 127.19, 126.64, 39.18, 29.72, 23.30.

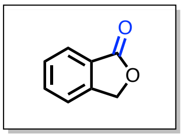

isobenzofuran-1(3H)-one (12)[24]: The product was purified by column chromatography on silica gel (10:1, petroleum ether/ethyl acetate), white solid, 52.3 mg, 78% yield.

^{1}H NMR (400 MHz, $CDCl_3$) δ 7.93 (dd, *J* = 7.7, 1.1 Hz, 1H), 7.70 (td, *J* = 7.5, 1.1 Hz, 1H), 7.64 – 7.47 (m, 2H), 5.33 (s, 2H).

^{13}C NMR (101 MHz, $CDCl_3$) δ 171.11, 146.54, 134.02, 129.06, 125.80, 125.78, 122.10, 69.66.

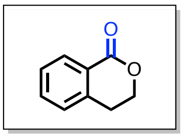

isochroman-1-one (13)[21]: The product was purified by column chromatography on silica gel (10:1, petroleum ether/ethyl acetate), yellow oil, 45.1 mg, 61% yield.

^{1}H NMR (400 MHz, $CDCl_3$) δ 8.10 (dd, J = 7.8, 1.4 Hz, 1H), 7.54 (td, J = 7.5, 1.4 Hz, 1H), 7.40 (td, J = 7.6, 1.2 Hz, 1H), 7.27 (d, J = 7.6 Hz, 1H), 4.54 (t, J = 6.0 Hz, 2H), 3.07 (t, J = 6.0 Hz, 2H).

^{13}C NMR (101 MHz, $CDCl_3$) δ 165.12, 139.55, 133.67, 130.45, 127.72, 127.23, 125.36, 67.31, 27.86.

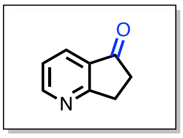

6,7-dihydro-5H-cyclopenta[b]pyridin-5-one (14)[25]: The product was purified by column chromatography on silica gel (10:1, petroleum ether/ethyl acetate), yellow oil, 22.6 mg, 34% yield.

^{1}H NMR (400 MHz, $CDCl_3$) δ 8.82 (dd, J = 4.8, 1.7 Hz, 1H), 8.03 (dd, J = 7.7, 1.8 Hz, 1H), 7.42 – 7.31 (m, 1H), 3.37 – 3.23 (m, 2H), 2.89 – 2.67 (m, 2H).

^{13}C NMR (101 MHz, $CDCl_3$) δ 204.95, 174.37, 155.73, 131.94, 130.33, 122.49, 35.78, 28.73.

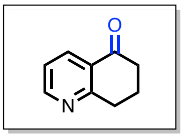

7,8-dihydroquinolin-5(6H)-one (15)[25]: The product was purified by column chromatography on silica gel (10:1, petroleum ether/ethyl acetate), yellow oil, 22.0 mg, 30% yield.

^{1}H NMR (400 MHz, $CDCl_3$) δ 8.68 (dd, J = 4.8, 1.9 Hz, 1H), 8.28 (dd, J = 7.8, 1.9 Hz, 1H), 7.38 – 7.23 (m, 1H), 3.17 (t, J = 6.2 Hz, 2H), 2.71 (dd, J = 7.4, 5.8 Hz, 2H), 2.21 (tt, J = 7.4, 5.8 Hz, 2H).

^{13}C NMR (101 MHz, $CDCl_3$) δ 197.97, 163.69, 153.50, 135.04, 128.18, 122.26, 38.56, 32.54, 21.86.

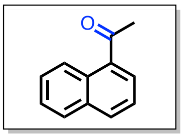

1-(naphthalen-1-yl)ethan-1-one (16)[20]: The product was purified by column chromatography on silica gel (10:1, petroleum ether/ethyl acetate), pale yellow oil, 40.8 mg, 48% yield.

^{1}H NMR (400 MHz, $CDCl_3$) δ 8.74 (dd, J = 8.7, 1.3 Hz, 1H), 7.99 (d, J = 8.2 Hz, 1H), 7.97 – 7.91

(m, 1H), 7.91 – 7.83 (m, 1H), 7.60 (ddd, J = 8.5, 6.7, 1.5 Hz, 1H), 7.58 – 7.40 (m, 1H), 2.74 (s, 3H).

^{13}C NMR (101 MHz, $CDCl_3$) δ 201.90, 135.49, 134.00, 133.06, 130.16, 128.69, 128.43, 128.09, 126.47, 126.03, 124.35, 30.01.

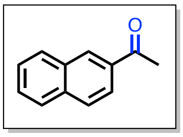

1-(naphthalen-2-yl)ethan-1-one (17) [20]**:** The product was purified by column chromatography on silica gel (10:1, petroleum ether/ethyl acetate), white solid, 54.3 mg, 64% yield.

^{1}H NMR (400 MHz, $CDCl_3$) δ 8.55 – 8.44 (m, 1H), 8.04 (dd, J = 8.6, 1.8 Hz, 1H), 8.00 – 7.93 (m, 1H), 7.89 (dd, J = 8.5, 6.2 Hz, 2H), 7.58 (dddd, J = 19.5, 8.1, 6.9, 1.4 Hz, 2H), 2.73 (s, 1H).

^{13}C NMR (101 MHz, $CDCl_3$) δ 198.15, 135.62, 134.52, 132.54, 130.21, 129.57, 128.46, 127.80, 126.79, 123.92, 53.43, 50.89, 26.70.

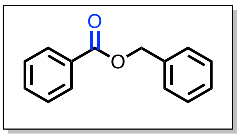

benzyl benzoate (18)[46]: The product was purified by column chromatography on silica gel (10:1, petroleum ether/ethyl acetate), colorless oil, 46.6 mg, 44% yield.

^{1}H NMR (400 MHz, $CDCl_3$) δ 8.17 – 7.97 (m, 2H), 7.62 – 7.50 (m, 1H), 7.50 – 7.28 (m, 7H), 5.36 (s, 2H).

^{13}C NMR (101 MHz, $CDCl_3$) δ 166.46, 136.12, 133.06, 130.20, 129.74, 128.64, 128.41, 128.27, 128.20, 66.72.

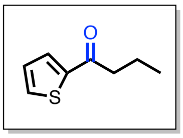

1-(thiophen-2-yl)butan-1-one (19)[13]: The product was purified by column chromatography on silica gel (10:1, petroleum ether/ethyl acetate), yellow oil, 43.1 mg, 56% yield .

^{1}H NMR (400 MHz, $CDCl_3$) δ 7.71 (dd, J = 3.8, 1.1 Hz, 1H), 7.62 (dd, J = 5.0, 1.1 Hz, 1H), 7.13 (dd, J = 4.9, 3.8 Hz, 1H), 2.88 (t, J = 7.3 Hz, 2H), 1.79 (h, J = 7.4 Hz, 2H), 1.01 (t, J = 7.4 Hz, 3H).

^{13}C NMR (101 MHz, $CDCl_3$) δ 193.39, 144.60, 133.29, 131.64, 128.01, 41.32, 29.70, 18.23, 13.87.

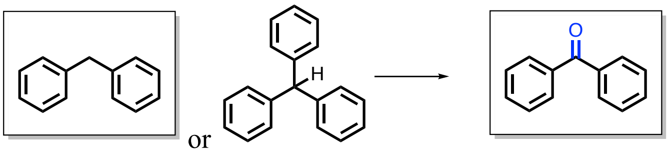

Benzophenone (20)[21]: For the diphenylmethane, the product was purified by column chromatography on silica gel (10:1, petroleum ether/ethyl acetate), white solid, 79.2 mg, 87% yield For the triphenylmethane, following the general procedure A, 68.3 mg, 75% yield.

^{1}H NMR (400 MHz, $CDCl_3$) δ 7.85 – 7.77 (m, 4H), 7.64 – 7.54 (m, 2H), 7.53 – 7.41 (m, 4H).

^{13}C NMR (101 MHz, $CDCl_3$) δ 196.80, 137.61, 132.45, 130.09, 128.30.

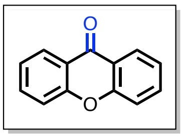

9H-xanthen-9-one (21)[21]: The product was purified by column chromatography on silica gel (10:1, petroleum ether/ethyl acetate), white solid, 64.7 mg, 66% yield.

^{1}H NMR (400 MHz, $CDCl_3$) δ 8.35 (dd, J = 8.0, 1.8 Hz, 2H), 7.74 (ddd, J = 8.6, 7.1, 1.8 Hz, 2H), 7.51 (dd, J = 8.5, 1.1 Hz, 2H), 7.39 (ddd, J = 8.1, 7.1, 1.1 Hz, 2H).

^{13}C NMR (101 MHz, $CDCl_3$) δ 156.22, 134.83, 126.77, 123.93, 121.90, 118.00.

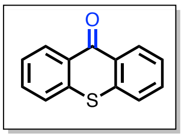

9H-thioxanthen-9-one (22)[24]: The product was purified by column chromatography on silica gel (10:1, petroleum ether/ethyl acetate), yellow solid, 85.9 mg, 81% yield.

^{1}H NMR (400 MHz, $CDCl_3$) δ 8.63 (dd, J = 8.2, 1.5 Hz, 2H), 7.61 (dtd, J = 14.3, 8.1, 7.4, 1.6 Hz, 4H), 7.49 (ddd, J = 8.3, 6.7, 1.6 Hz, 2H).

^{13}C NMR (101 MHz, $CDCl_3$) δ 180.00, 137.31, 132.29, 129.90, 129.27, 126.33, 126.01.

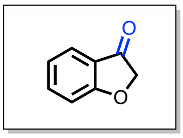

benzofuran-3(2H)-one (23)[47]: The product was purified by column chromatography on silica gel (10:1, petroleum ether/ethyl acetate), yellow solid, 42.8 mg, 64% yield.

^{1}H NMR (400 MHz, $CDCl_3$) δ 7.68 (ddd, J = 7.7, 1.5, 0.7 Hz, 1H), 7.62 (ddd, J = 8.6, 7.2, 1.5 Hz, 1H), 7.15 (dt, J = 8.4, 0.8 Hz, 1H), 7.12 – 7.07 (m, 1H), 4.63 (s, 2H).

^{13}C NMR (101 MHz, $CDCl_3$) δ 199.87, 174.03, 137.87, 124.10, 122.01, 113.68, 74.70.

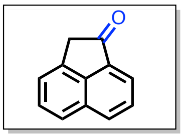

acenaphthylen-1(2H)-one (24)[21]: The product was purified by column chromatography on silica gel (10:1, petroleum ether/ethyl acetate), pale yellow solid, 85.9 mg, 85% yield.

^1H NMR (400 MHz, $CDCl_3$) δ 8.09 (dd, J = 8.1, 0.8 Hz, 1H), 7.96 (dd, J = 7.0, 0.7 Hz, 1H), 7.87 – 7.79 (m, 1H), 7.71 (dd, J = 8.1, 7.0 Hz, 1H), 7.60 (dd, J = 8.4, 6.9 Hz, 1H), 7.46 (dd, J = 6.8, 1.0 Hz, 1H), 3.82 (t, J = 0.9 Hz, 2H).

^{13}C NMR (101 MHz, $CDCl_3$) δ 202.97, 142.95, 135.02, 134.71, 131.49, 130.96, 128.38, 128.01, 123.96, 121.45, 121.04, 42.01.

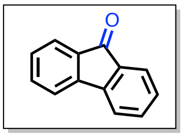

9H-fluoren-9-one (25)[21]: The product was purified by column chromatography on silica gel (10:1, petroleum ether/ethyl acetate), yellow solid, 75.6 mg, 84% yield.

^1H NMR (400 MHz, $CDCl_3$) δ 7.65 (dt, J = 7.4, 1.0 Hz, 2H), 7.56 – 7.40 (m, 4H), 7.29 (td, J = 7.2, 1.4 Hz, 2H).

^{13}C NMR (101 MHz, $CDCl_3$) δ 193.93, 144.45, 134.69, 134.18, 129.09, 124.33, 120.31.

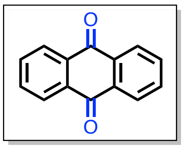

anthracene-9,10-dione (26)[21]: The product was purified by column chromatography on silica gel (10:1, petroleum ether/ethyl acetate), yellow solid, 98.8 mg, 95% yield.

^1H NMR (400 MHz, $CDCl_3$) δ 8.38 – 8.25 (m, 4H), 7.90 – 7.71 (m, 4H).

^{13}C NMR (101 MHz, $CDCl_3$) δ 183.18, 134.14, 133.55, 127.25.

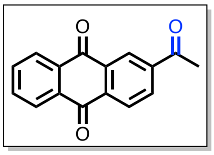

2-acetylanthracene-9,10-dione (27)[13]: The product was purified by column chromatography on silica gel (10:1, petroleum ether/ethyl acetate), yellow solid, 102.5 mg, 82% yield.

^{1}H NMR (400 MHz, $CDCl_3$) δ 8.94 – 8.72 (m, 1H), 8.47 – 8.28 (m, 4H), 7.95 – 7.80 (m, 2H), 2.76 (d, J = 0.9 Hz, 3H).

^{13}C NMR (101 MHz, $CDCl_3$) δ 196.79, 182.51, 182.45, 141.05, 136.05, 134.52, 133.75, 133.44, 133.40, 132.93, 127.86, 127.48, 127.46, 127.37, 27.07.

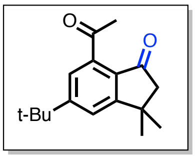

7-acetyl-5-(tert-butyl)-3,3-dimethyl-2,3-dihydro-1H-inden-1-one (28)[21]: The product was purified by column chromatography on silica gel (10:1, petroleum ether/ethyl acetate), white solid, 55.5 mg, 43% yield.

^{1}H NMR (500 MHz, $CDCl_3$) δ 7.55 (d, J = 1.6 Hz, 1H), 7.33 (d, J = 1.6 Hz, 1H), 2.64 (s, 2H), 2.61 (s, 2H), 1.44 (s, 4H), 1.37 (s, 6H).

^{13}C NMR (101 MHz, $CDCl_3$) δ 204.76, 204.10, 164.61, 159.42, 139.23, 129.37, 123.27, 121.77, 53.26, 38.71, 35.79, 31.44, 31.14, 30.97, 30.05.

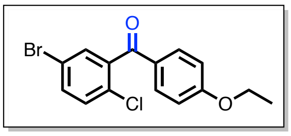

(5-bromo-2-chlorophenyl)(4-ethoxyphenyl)methanone (29)[26]: The product was purified by column chromatography on silica gel (10:1, petroleum ether/ethyl acetate), white solid, 91.2 mg, 54% yield..

^{1}H NMR (400 MHz, $CDCl_3$) δ 7.96 – 7.69 (m, 2H), 7.53 (dd, J = 8.5, 2.4 Hz, 1H), 7.48 (d, J = 2.4 Hz, 1H), 7.32 (d, J = 8.5 Hz, 1H), 6.93 (d, J = 8.9 Hz, 2H), 4.11 (q, 2H), 1.44 (t, 3H).

^{13}C NMR (101 MHz, $CDCl_3$) δ 192.03, 163.88, 140.75, 133.68, 132.58, 131.57, 131.44, 130.13, 128.66, 120.49, 114.49, 63.95, 14.63.

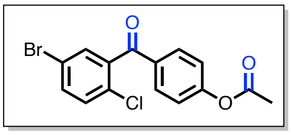

4-(5-bromo-2-chlorobenzoyl)phenyl acetate (29b): The by-product was purified by column chromatography on silica gel (10:1, petroleum ether/ethyl acetate), white solid, 36.5 mg, 21% yield.

^{1}H NMR (400 MHz, $CDCl_3$) δ 7.88 – 7.81 (m, 2H), 7.56 (dd, J = 8.5, 2.4 Hz, 1H), 7.50 (d, J = 2.4 Hz, 1H), 7.34 (d, J = 8.5 Hz, 1H), 7.25 – 7.18 (m, 2H), 2.33 (s, 3H).

^{13}C NMR (101 MHz, $CDCl_3$) δ 192.31, 168.70, 155.14, 139.98, 134.17, 133.36, 131.77, 131.67, 131.60, 130.24, 122.06, 120.61, 21.20.

HRMS $[M+H]^+$ calcd for $[C_{15}H_{11}BrClO_3]^+$: 352.9580; found: 352.9578.

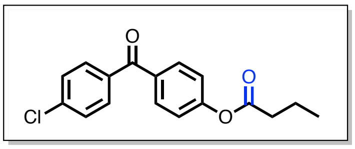

4-(4-chlorobenzoyl)phenyl butyrate (30): The product was purified by column chromatography on silica gel (10:1, petroleum ether/ethyl acetate), white solid, 27.1 mg, 18% yield.

^1H NMR (400 MHz, $CDCl_3$) δ 7.82 (d, J = 8.6 Hz, 2H), 7.75 (d, J = 8.5 Hz, 2H), 7.47 (d, J = 8.4 Hz, 2H), 7.22 (d, J = 8.6 Hz, 2H), 2.58 (t, J = 7.4 Hz, 2H), 1.81 (h, J = 7.4 Hz, 2H), 1.06 (t, J = 7.4 Hz, 3H).

^{13}C NMR (101 MHz, $CDCl_3$) δ 194.30, 171.58, 154.20, 138.97, 135.83, 134.63, 131.53, 131.35, 128.70, 121.69, 36.24, 18.38, 13.61.

HRMS $[M+H]^+$ calcd for $[C_{17}H_{16}ClO_3]^+$: 303.0788; found: 303.0787.

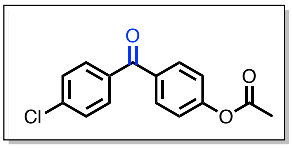

4-(4-chlorobenzoyl)phenyl acetate (31)[27]: The product was purified by column chromatography on silica gel (10:1, petroleum ether/ethyl acetate), white solid, 68.5 mg, 50% yield.

^1H NMR (400 MHz, $CDCl_3$) δ 7.75 (d, J = 8.2 Hz, 2H), 7.67 (d, J = 8.1 Hz, 2H), 7.39 (d, J = 8.2 Hz, 2H), 7.15 (d, J = 8.3 Hz, 2H), 2.27 (s, 3H).

^{13}C NMR (101 MHz, $CDCl_3$) δ 194.30, 168.91, 154.06, 138.98, 135.76, 134.71, 131.56, 131.37, 128.72, 121.70, 21.18.

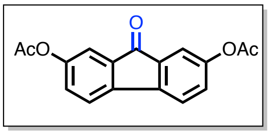

9-oxo-9H-fluorene-2,7-diyl diacetate (32): The product was purified by column chromatography on silica gel (10:1, petroleum ether/ethyl acetate), yellow solid, 51.8 mg, 35% yield.

^1H NMR (500 MHz, DMSO-d_6) δ 7.84 (d, J = 8.0 Hz, 2H), 7.41 (d, J = 2.2 Hz, 2H), 7.37 (dd, J = 8.0, 2.2 Hz, 2H), 2.29 (s, 6H).

^{13}C NMR (126 MHz, DMSO-d_6) δ 191.18, 169.09, 151.28, 140.71, 134.85, 128.35, 122.18, 118.14,

20.83.

HRMS $[M+H]^+$ calcd for $[C_{17}H_{13}O_5]^+$: 297.0763; found: 297.0762.

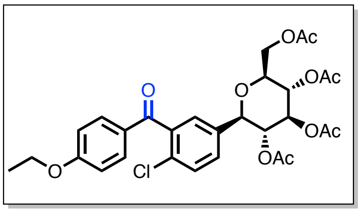

(2S,3S,4S,5R,6R)-2-(acetoxymethyl)-6-(4-chloro-3-(4-ethoxybenzoyl)phenyl)tetrahydro-2H-pyran-3,4,5-triyl triacetate (33)[28]: The product was purified by column chromatography on silica gel (10:1, petroleum ether/ethyl acetate), white solid, 64.5 mg, 55% yield.

^{1}H NMR (400 MHz, $CDCl_3$) δ 7.74 (d, J = 8.9 Hz, 2H), 7.43 (d, J = 1.7 Hz, 2H), 7.30 (d, J = 1.5 Hz, 1H), 6.93 (d, J = 8.9 Hz, 2H), 5.32 (t, J = 9.4 Hz, 1H), 5.20 (t, J = 9.7 Hz, 1H), 5.06 (t, J = 9.6 Hz, 1H), 4.41 (d, J = 9.9 Hz, 1H), 4.28 (dd, J = 12.4, 4.9 Hz, 1H), 4.20 – 4.07 (m, 3H), 3.88 – 3.80 (m, 1H), 2.06 (d, J = 10.4 Hz, 6H), 2.00 (s, 3H), 1.86 (s, 3H), 1.44 (t, 3H).

^{13}C NMR (101 MHz, $CDCl_3$) δ 193.32, 170.75, 170.32, 169.52, 168.90, 163.73, 139.04, 135.36, 132.55, 131.50, 130.17, 129.03, 128.90, 127.66, 114.42, 79.11, 76.24, 73.93, 72.65, 68.48, 63.91, 62.26, 20.78, 20.63, 20.50.

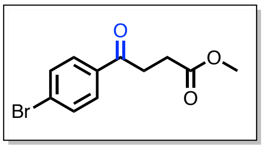

methyl 4-(4-bromophenyl)-4-oxobutanoate (34)[29]: The product was purified by column chromatography on silica gel (10:1, petroleum ether/ethyl acetate), white solid, 90.0 mg, 67% yield.

^{1}H NMR (400 MHz, $CDCl_3$) δ 7.78 (d, J = 8.4 Hz, 2H), 7.54 (d, J = 8.3 Hz, 2H), 3.64 (s, 3H), 3.21 (t, J = 6.6 Hz, 2H), 2.70 (t, J = 6.6 Hz, 2H).

^{13}C NMR (101 MHz, $CDCl_3$) δ 197.07, 173.23, 135.28, 131.96, 129.57, 128.44, 51.88, 33.35, 27.94.

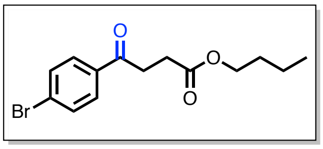

butyl 4-(4-bromophenyl)-4-oxobutanoate (35)[30]: The product was purified by column chromatography on silica gel (10:1, petroleum ether/ethyl acetate), white solid, 79.5 mg, 51% yield.

^{1}H NMR (400 MHz, $CDCl_3$) δ 7.77 (d, J = 8.2 Hz, 2H), 7.54 (d, J = 8.3 Hz, 2H), 4.03 (t, J = 6.7 Hz,

2H), 3.19 (t, J = 6.6 Hz, 2H), 2.69 (t, J = 6.6 Hz, 2H), 1.54 (p, J = 7.0 Hz, 2H), 1.30 (h, J = 7.4 Hz, 2H), 0.85 (t, J = 7.4 Hz, 3H).

^{13}C NMR (101 MHz, $CDCl_3$) δ 197.16, 172.85, 135.36, 131.94, 129.57, 128.38, 64.67, 33.35, 30.64, 28.21, 19.11, 13.68.

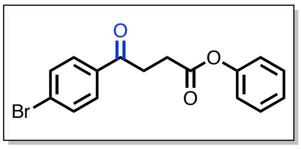

phenyl 4-(4-bromophenyl)-4-oxobutanoate (36)[31]: The product was purified by column chromatography on silica gel (10:1, petroleum ether/ethyl acetate), white solid, 79.6 mg, 48% yield.

^{1}H NMR (400 MHz, $CDCl_3$) δ 7.79 (d, J = 8.2 Hz, 2H), 7.54 (d, J = 8.2 Hz, 2H), 7.30 (t, J = 7.8 Hz, 2H), 7.15 (q, J = 7.8 Hz, 1H), 7.03 (d, J = 8.0 Hz, 2H), 3.30 (t, J = 6.5 Hz, 2H), 2.94 (t, J = 6.5 Hz, 2H).

^{13}C NMR (101 MHz, $CDCl_3$) δ 196.88, 171.43, 150.73, 135.23, 132.01, 129.59, 129.42, 128.54, 125.85, 121.53, 33.40, 28.40.

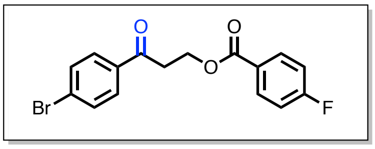

3-(4-bromophenyl)-3-oxopropyl 4-fluorobenzoate (37): The product was purified by column chromatography on silica gel (10:1, petroleum ether/ethyl acetate), white solid, 91.4 mg, 52% yield.

^{1}H NMR (400 MHz, $CDCl_3$) δ 8.00 (dd, J = 8.7, 5.6 Hz, 2H), 7.85 (d, J = 8.5 Hz, 2H), 7.63 (d, J = 8.5 Hz, 2H), 7.08 (t, J = 8.6 Hz, 2H), 4.75 (t, J = 6.4 Hz, 2H), 3.41 (t, J = 6.4 Hz, 2H).

^{13}C NMR (101 MHz, $CDCl_3$) δ 195.97, 165.53, 135.29, 132.23, 132.13, 132.09, 129.62, 128.75, 115.65, 115.43, 60.22, 37.47.

^{19}F NMR (376 MHz, $CDCl_3$) δ -105.40.

HRMS $[M+H]^+$ calcd for $[C_{16}H_{13}BrFO_3]^+$: 351.0032; found: 351.0029.

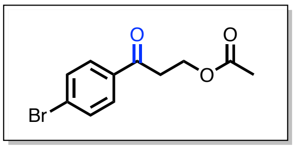

3-(4-bromophenyl)-3-oxopropyl acetate (38): The product was purified by column chromatography on silica gel (10:1, petroleum ether/ethyl acetate), white solid, 87.4 mg, 65% yield.

^{1}H NMR (400 MHz, $CDCl_3$) δ 7.83 (d, J = 8.5 Hz, 2H), 7.63 (d, J = 8.5 Hz, 2H), 4.51 (t, J = 6.4 Hz,

2H), 3.28 (t, J = 6.3 Hz, 2H), 2.04 (s, 3H).

^{13}C NMR (101 MHz, $CDCl_3$) δ 196.02, 171.02, 135.31, 132.06, 129.60, 128.69, 59.51, 37.34, 20.92.

HRMS $[M+H]^+$ calcd for $[C_{11}H_{12}BrO_3]^+$: 270.9970; found: 270.9967.

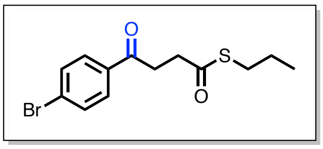

S-propyl 4-(4-bromophenyl)-4-oxobutanethioate (40): The product was purified by column chromatography on silica gel (10:1, petroleum ether/ethyl acetate), white solid, 78.4 mg, 50% yield.

^{1}H NMR (400 MHz, $CDCl_3$) δ 7.92 – 7.77 (m, 2H), 7.73 – 7.51 (m, 2H), 3.30 (t, J = 6.7 Hz, 2H), 3.02 (t, J = 6.7 Hz, 2H), 2.97 – 2.79 (m, 2H), 1.61 (h, J = 7.4, 6.8 Hz, 2H), 0.97 (td, J = 7.4, 1.2 Hz, 3H).

^{13}C NMR (101 MHz, $CDCl_3$) δ 198.53, 196.74, 135.18, 131.97, 129.60, 128.48, 37.68, 33.49, 30.89, 22.94, 13.35.

HRMS $[M+H]^+$ calcd for $[C_{13}H_{16}BrO_2S]^+$: 315.0054; found: 315.0052.

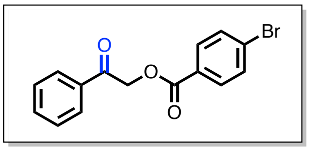

2-oxo-2-phenylethyl 4-bromobenzoate (41)[32]: The product was purified by column chromatography on silica gel (10:1, petroleum ether/ethyl acetate), white solid, 98.6 mg, 62% yield.

^{1}H NMR (500 MHz, $CDCl_3$) δ 8.03 – 7.98 (m, 2H), 7.98 – 7.94 (m, 2H), 7.66 – 7.60 (m, 3H), 7.52 (t, J = 7.8 Hz, 2H), 5.58 (s, 2H).

^{13}C NMR (101 MHz, $CDCl_3$) δ 191.84, 165.37, 134.24, 134.00, 131.85, 131.50, 128.95, 128.58, 128.37, 127.83, 66.59.

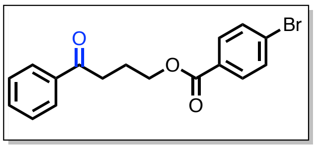

4-oxo-4-phenylbutyl 4-bromobenzoate (42): The product was purified by column chromatography on silica gel (10:1, petroleum ether/ethyl acetate), white solid, 106.2 mg, 64% yield.

^{1}H NMR (400 MHz, $CDCl_3$) δ 8.07 – 7.92 (m, 2H), 7.87 (d, J = 8.5 Hz, 2H), 7.56 (dd, J = 7.9, 3.1 Hz, 3H), 7.46 (t, J = 7.6 Hz, 2H), 4.43 (t, J = 6.4 Hz, 2H), 3.15 (t, J = 7.1 Hz, 2H), 2.25 (p, J = 6.8 Hz, 2H).

^{13}C NMR (126 MHz, CDCl$_3$) δ 198.96, 165.84, 136.75, 133.22, 131.72, 131.11, 129.57, 129.11, 128.66, 128.37, 128.07, 128.02, 64.62, 34.91, 23.28.

HRMS [M+H]$^+$ calcd for [$C_{17}H_{16}BrO_3$]$^+$: 347.0283; found: 347.0280.

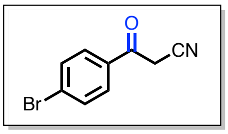

3-(4-bromophenyl)-3-oxopropanenitrile (43)[33]: The product was purified by column chromatography on silica gel (10:1, petroleum ether/ethyl acetate), white solid, 56.9 mg, 51% yield.

^{1}H NMR (400 MHz, CDCl$_3$) δ 7.79 (d, *J* = 8.6 Hz, 2H), 7.68 (d, *J* = 8.6 Hz, 2H), 4.06 (s, 2H).

^{13}C NMR (101 MHz, CDCl$_3$) δ 186.23, 132.96, 132.59, 130.33, 129.89, 113.45, 29.41

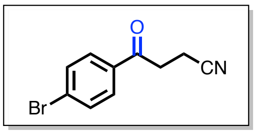

4-(4-bromophenyl)-4-oxobutanenitrile (44)[34]: The product was purified by column chromatography on silica gel (10:1, petroleum ether/ethyl acetate), white solid, 65.1 mg, 55% yield.

^{1}H NMR (400 MHz, CDCl$_3$) δ 8.08 – 7.73 (m, 2H), 7.73 – 7.51 (m, 2H), 3.35 (t, *J* = 7.2 Hz, 2H), 2.78 (t, *J* = 7.1 Hz, 2H).

^{13}C NMR (126 MHz, CDCl$_3$) δ 194.34, 134.30, 132.27, 129.49, 129.27, 118.99, 34.26, 11.77.

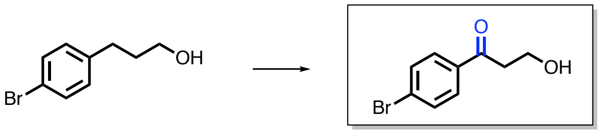

1-(4-bromophenyl)-3-hydroxypropan-1-one (45)[35]: The product was purified by column chromatography on silica gel (5:1, petroleum ether/ethyl acetate), white solid, 41.1 mg, 36% yield.

^{1}H NMR (400 MHz, CDCl$_3$) δ 7.83 (d, *J* = 8.6 Hz, 2H), 7.62 (d, *J* = 8.6 Hz, 2H), 4.04 (t, *J* = 5.3 Hz, 2H), 3.20 (t, *J* = 5.3 Hz, 2H).

^{13}C NMR (101 MHz, CDCl$_3$) δ 199.40, 135.37, 132.06, 129.58, 128.81, 57.93, 40.39.

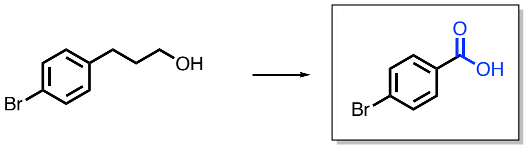

4-bromobenzoic acid (46)[49]: The product was purified by column chromatography on silica gel (10:1-5:1, petroleum ether/ethyl acetate), white solid, 67.2 mg, 67% yield.

^{1}H NMR (400 MHz, DMSO-d$_6$) δ 13.42 – 12.56 (m, 1H), 7.96 – 7.79 (m, 2H), 7.72 (d, J = 8.3 Hz, 2H).

^{13}C NMR (101 MHz, DMSO-d$_6$) δ 167.07, 132.17, 131.76, 130.48, 127.35.

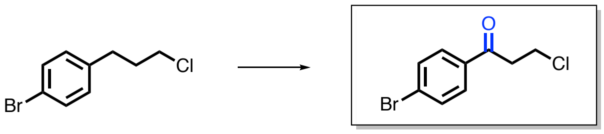

1-(4-bromophenyl)-3-chloropropan-1-one (47)[36]: The product was purified by column chromatography on silica gel (100:1, petroleum ether/ethyl acetate), yellow oil, 55.2 mg, 45% yield.

^{1}H NMR (600 MHz, $CDCl_3$) δ 8.08 – 7.73 (m, 2H), 7.73 – 7.52 (m, 2H), 3.92 (t, J = 6.8 Hz, 2H), 3.43 (t, J = 6.8 Hz, 2H).

^{13}C NMR (101 MHz, $CDCl_3$) δ 195.75, 135.08, 132.12, 129.68, 128.87, 41.23, 38.47.

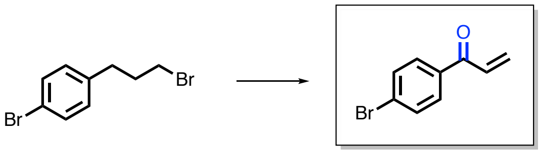

1-(4-bromophenyl)prop-2-en-1-one (48)[37]: The product was purified by column chromatography on silica gel (petroleum ether), white solid, 84.2 mg, 80% yield.

^{1}H NMR (600 MHz, $CDCl_3$) δ 7.81 (dd, J = 8.5, 1.6 Hz, 2H), 7.62 (dd, J = 8.5, 1.6 Hz, 2H), 7.11 (ddd, J = 17.2, 10.6, 1.4 Hz, 1H), 6.44 (dd, J = 17.2, 1.7 Hz, 1H), 5.95 (d, J = 10.6 Hz, 1H).

^{13}C NMR (101 MHz, $CDCl_3$) δ 189.96, 135.98, 131.98, 131.92, 130.75, 130.23, 128.20.

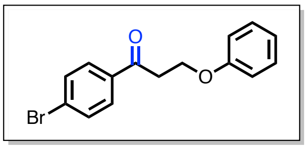

1-(4-bromophenyl)-3-phenoxypropan-1-one (50): The product was purified by column chromatography on silica gel (10:1, petroleum ether/ethyl acetate), white solid, 83.5 mg, 55% yield.

^{1}H NMR (400 MHz, $CDCl_3$) δ 7.87 (d, J = 8.2 Hz, 2H), 7.63 (d, J = 8.2 Hz, 2H), 7.36 – 7.18 (m, 2H), 6.93 (dd, J = 12.1, 7.5 Hz, 3H), 4.42 (t, J = 6.6 Hz, 2H), 3.43 (t, J = 6.5 Hz, 2H).

^{13}C NMR (101 MHz, $CDCl_3$) δ 196.74, 158.54, 135.56, 132.02, 129.71, 129.52, 128.62, 121.04, 114.57, 63.07, 38.17.

HRMS $[M+H]^+$ calcd for $[C_{15}H_{14}BrO_2]^+$: 305.0177; found: 305.0175.

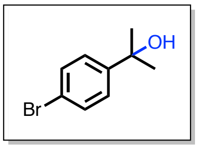

2-(4-bromophenyl)propan-2-ol (51)[38]: The product was purified by column chromatography on silica gel (10:1, petroleum ether/ethyl acetate), light yellow oil, 53.4 mg, 50% yield.

^{1}H NMR (500 MHz, $CDCl_3$) δ 7.44 (d, J = 8.5 Hz, 2H), 7.35 (d, J = 8.6 Hz, 2H), 1.55 (s, 6H).

^{13}C NMR (126 MHz, $CDCl_3$) δ 148.17, 131.23, 126.38, 120.56, 72.30, 31.71.

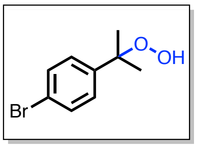

1-bromo-4-(2-hydroperoxypropan-2-yl)benzene (51b)[39]: The peroxide product was purified by column chromatography on silica gel (10:1, petroleum ether/ethyl acetate), light yellow oil, 28.5 mg, 25% yield.

^{1}H NMR (500 MHz, $CDCl_3$) δ 7.48 (d, J = 8.1 Hz, 2H), 7.33 (d, J = 8.3 Hz, 2H), 1.57 (s, 6H).

^{13}C NMR (126 MHz, $CDCl_3$) δ 143.84, 131.59, 127.33, 121.43, 83.64, 26.03.

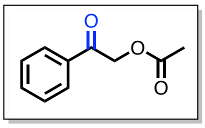

2-oxo-2-phenylethyl acetate (52)[40]: The product was purified by column chromatography on silica gel (10:1, petroleum ether/ethyl acetate), white solid, 28.5 mg, 32% yield.

^{1}H NMR (400 MHz, $CDCl_3$) δ 7.92 (dd, J = 8.3, 1.4 Hz, 2H), 7.68 – 7.57 (m, 1H), 7.49 (t, J = 7.7 Hz, 2H), 5.35 (s, 2H), 2.24 (s, 3H).

^{13}C NMR (126 MHz, $CDCl_3$) δ 192.17, 170.46, 133.92, 127.77, 66.03, 20.60.

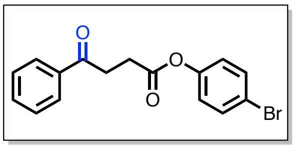

4-bromophenyl 4-oxo-4-phenylbutanoate (53): The product was purified by column chromatography on silica gel (10:1, petroleum ether/ethyl acetate), white solid, 66.3 mg, 40% yield.

^{1}H NMR (400 MHz, $CDCl_3$) δ 8.31 – 7.85 (m, 2H), 7.59 (t, J = 7.4 Hz, 1H), 7.48 (dt, J = 7.9, 3.4 Hz, 3H), 7.02 (d, J = 8.7 Hz, 2H), 3.43 (t, J = 6.4 Hz, 2H), 3.00 (t, J = 6.5 Hz, 2H).

^{13}C NMR (126 MHz, $CDCl_3$) δ 197.81, 171.31, 133.44, 132.44, 128.71, 128.07, 123.40, 33.41,

28.42.

HRMS $[M+H]^+$ calcd for $[C_{16}H_{14}BrO_3]^+$: 333.0126; found: 333.0122.

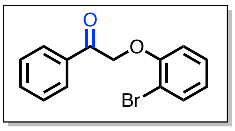

2-(2-bromophenoxy)-1-phenylethan-1-one (54)[41]: The product was purified by column chromatography on silica gel (10:1, petroleum ether/ethyl acetate), white solid, 65.3 mg, 45% yield.

^{1}H NMR (400 MHz, $CDCl_3$) δ 8.16 – 7.83 (m, 2H), 7.65 – 7.59 (m, 1H), 7.56 (dd, *J* = 7.9, 1.7 Hz, 1H), 7.51 (dd, *J* = 8.4, 7.0 Hz, 2H), 7.22 (td, *J* = 7.8, 1.6 Hz, 1H), 6.93 – 6.80 (m, 2H), 5.34 (s, 2H).

^{13}C NMR (126 MHz, $CDCl_3$) δ 194.10, 154.57, 134.39, 134.00, 133.71, 128.85, 128.46, 128.33, 122.94, 113.87, 112.42, 71.94.

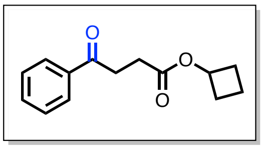

cyclobutyl 4-oxo-4-phenylbutanoate (55): The product was purified by column chromatography on silica gel (10:1, petroleum ether/ethyl acetate), white solid, 57.8 mg, 50% yield.

^{1}H NMR (400 MHz, $CDCl_3$) δ 8.22 – 7.80 (m, 2H), 7.67 – 7.52 (m, 1H), 7.47 (t, *J* = 7.7 Hz, 2H), 5.00 (p, *J* = 7.5 Hz, 1H), 3.30 (t, *J* = 6.7 Hz, 2H), 2.74 (t, *J* = 6.7 Hz, 2H), 2.34 (dtd, *J* = 12.5, 7.3, 6.1, 3.9 Hz, 2H), 2.08 (qdd, *J* = 10.0, 7.9, 2.8 Hz, 2H), 1.91 – 1.72 (m, 1H), 1.62 (ddt, *J* = 12.9, 10.3, 5.2 Hz, 2H).

^{13}C NMR (126 MHz, $CDCl_3$) δ 198.13, 172.30, 136.62, 133.20, 128.62, 128.05, 69.05, 33.36, 30.28, 28.29, 13.55.

HRMS $[M+H]^+$ calcd for $[C_{14}H_{17}O_3]^+$: 233.1178; found: 233.1175.

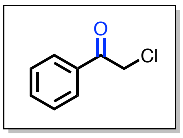

2-chloro-1-phenylethan-1-one (56)[13]: The product was purified by column chromatography on silica gel (10:1, petroleum ether/ethyl acetate), yellow oil, 50.1 mg, 65% yield.

^{1}H NMR (400 MHz, $CDCl_3$) δ 8.00 – 7.93 (m, 2H), 7.69 – 7.58 (m, 1H), 7.56 – 7.47 (m, 2H), 4.72 (s, 2H).

^{13}C NMR (101 MHz, $CDCl_3$) δ 191.10, 134.29, 134.01, 128.92, 128.55, 45.96.

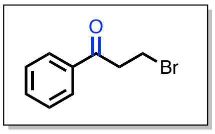

3-bromo-1-phenylpropan-1-one (57)[38]: The product was purified by column chromatography on silica gel (petroleum ether), yellow oil, 74.1 mg, 70% yield.

^{1}H NMR (400 MHz, $CDCl_3$) δ 7.96 – 7.83 (m, 2H), 7.61 – 7.49 (m, 1H), 7.42 (t, J = 7.7 Hz, 2H), 3.68 (t, J = 6.9 Hz, 2H), 3.51 (t, J = 6.9 Hz, 2H).

^{13}C NMR (101 MHz, $CDCl_3$) δ 196.99, 136.27, 133.59, 128.78, 128.07, 41.56, 25.74.

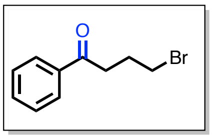

4-bromo-1-phenylbutan-1-one (58) [21]: The product was purified by column chromatography on silica gel (petroleum ether), yellow oil, 84.6 mg, 75% yield.

^{1}H NMR (400 MHz, $CDCl_3$) δ 8.07 – 7.94 (m, 2H), 7.58 (t, J = 7.7 Hz, 1H), 7.48 (t, J = 7.6 Hz, 2H), 3.56 (t, J = 6.3 Hz, 2H), 3.19 (t, J = 6.9 Hz, 2H), 2.32 (p, J = 6.6 Hz, 2H).

^{13}C NMR (126 MHz, $CDCl_3$) δ 198.84, 136.74, 133.25, 128.67, 128.03, 36.57, 33.64, 26.87.

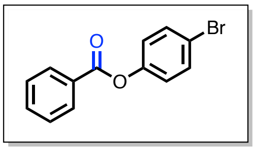

4-bromophenyl benzoate (59)[42]: The product was purified by column chromatography on silica gel (10:1, petroleum ether/ethyl acetate), white solid, 93.8 mg, 68% yield.

^{1}H NMR (400 MHz, $CDCl_3$) δ 8.24 – 8.12 (m, 2H), 7.73 – 7.61 (m, 1H), 7.53 (q, J = 8.1 Hz, 4H), 7.12 (d, J = 8.8 Hz, 2H).

^{13}C NMR (101 MHz, $CDCl_3$) δ 164.89, 150.00, 133.83, 132.56, 130.23, 129.19, 128.66, 123.56, 119.01.

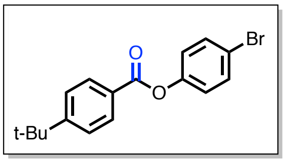

4-bromophenyl 4-(tert-butyl)benzoate (60): The product was purified by column chromatography on silica gel (10:1, petroleum ether/ethyl acetate), white solid, 116.2 mg, 70% yield.

^{1}H NMR (400 MHz, $CDCl_3$) δ 8.30 – 8.02 (m, 2H), 7.53 (dd, J = 8.7, 2.9 Hz, 4H), 7.10 (d, J = 8.8 Hz, 2H), 1.37 (s, 9H).

^{13}C NMR (126 MHz, $CDCl_3$) δ 164.88, 157.69, 150.08, 132.50, 130.13, 126.36, 125.65, 123.61, 118.87, 31.11.

HRMS $[M+H]^+$ calcd for $[C_{17}H_{18}BrO_2]^+$: 333.0490; found: 333.0487.

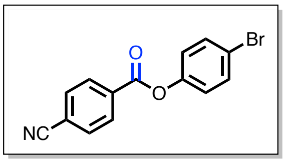

4-bromophenyl 4-cyanobenzoate (61): The product was purified by column chromatography on silica gel (10:1, petroleum ether/ethyl acetate), white solid, 75.2 mg, 50% yield.

^{1}H NMR (400 MHz, $CDCl_3$) δ 8.29 (d, J = 8.5 Hz, 2H), 7.98 – 7.74 (m, 2H), 7.67 – 7.48 (m, 2H), 7.20 – 6.98 (m, 2H).

^{13}C NMR (126 MHz, $CDCl_3$) δ 163.28, 149.54, 133.01, 132.73, 132.46, 130.68, 123.27, 119.52, 117.78, 117.23.

HRMS $[M+H]^+$ calcd for $[C_{14}H_9BrNO_2]^+$: 301.9817; found: 301.9815.

11.Studies on reaction mechanism

11.1 Spectroscopic characterization

11.1.1 Absorption spectra of 1 and 2

UV-vis absorption spectra of the substrates and products in EtOAc was collected using a UV-vis absorption spectrophotometer (UV8453). The different concentrations of 4-bromoethylbenzene (**1**) and 4-bromoacetophenones (**2**) were analyzed to record the absorption spectra (Figure S4 and S5). Similarly, the absorption spectra of 4-bromoethylbenzene (**1**, 0.1M) in different solvents was recorded (Figure S6).

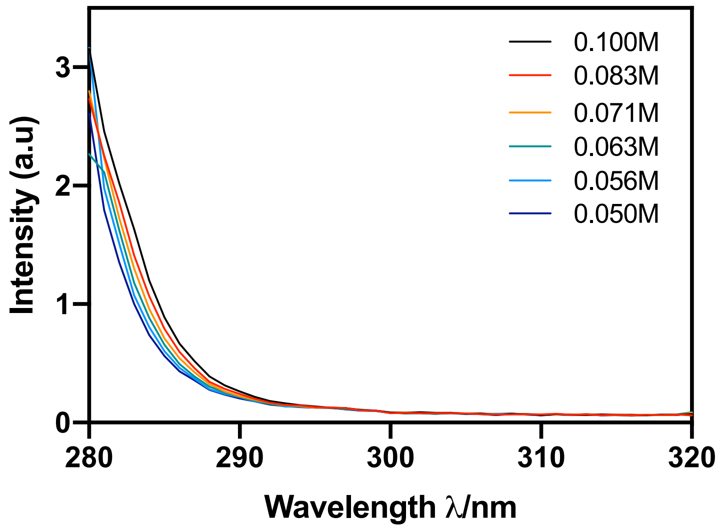

Figure S4. UV-visible spectra of 4-bromoethylbenzene (**1**) in EtOAc

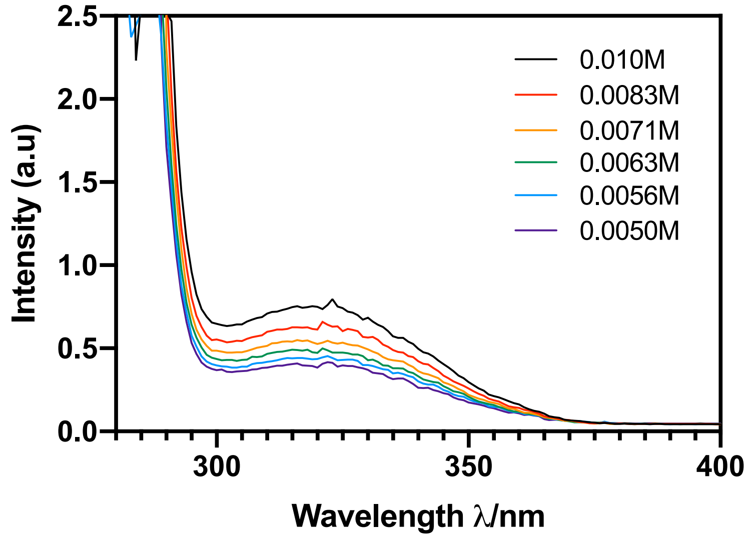

Figure S5. UV-visible spectra of 4-bromoacetophenones (**2**) in EtOAc

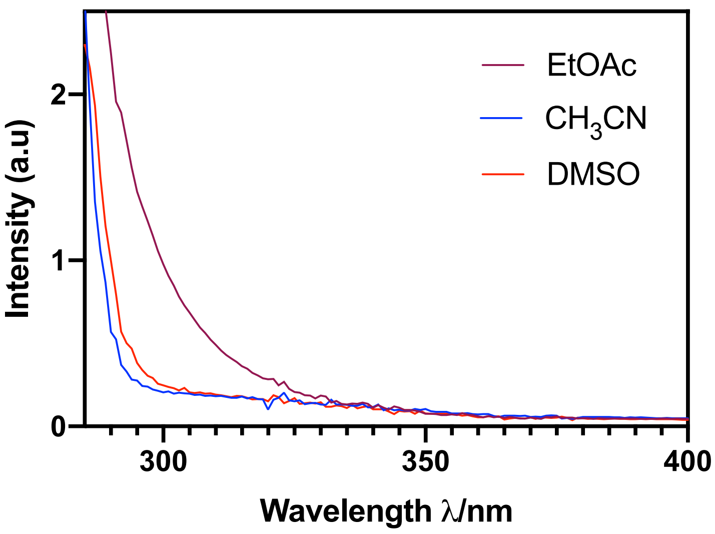

Figure S6. UV-visible spectra of 4-bromoethylbenzene (**1**) in different solvents (0.1 M)

11.1.2 Fluorescence spectra of 1 and 2

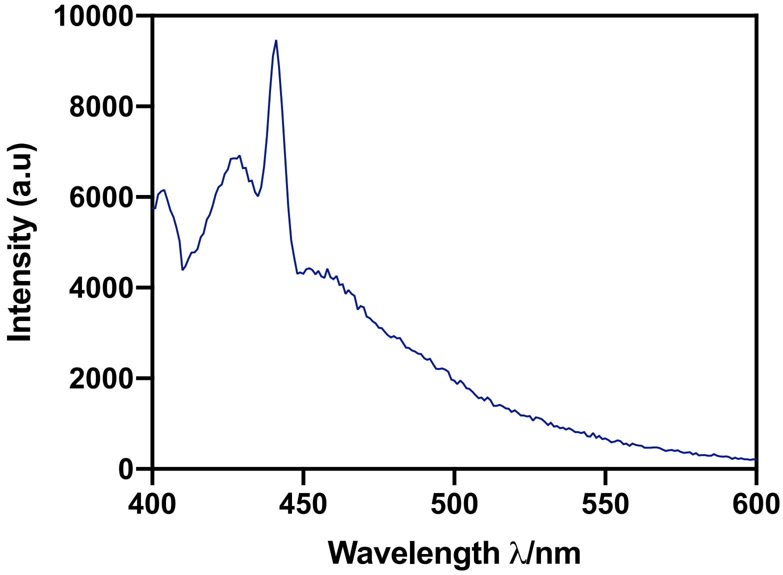

Figure S7. Fluorescence spectra of 4-bromoethylbenzene (**1**) in EtOAc under 390 nm excitation.

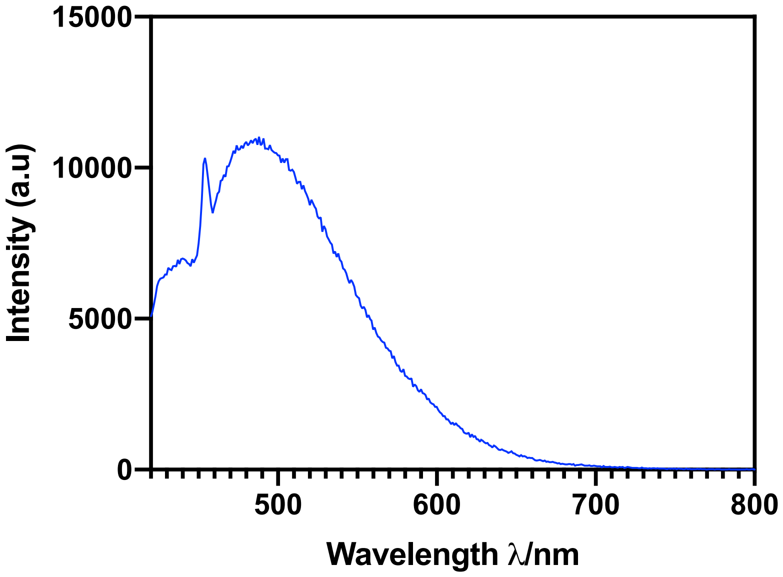

Figure S8. Fluorescence spectra of 4-bromoacetophenones (**2**) in EtOAc under 390 nm excitation.

11.1.3 Absorption spectra of different substrates

The absorption spectrums of different substrates (54a, 55a, 60a, 80a) were also measured in EtOAc (Figure S9). The substrates with high absorption intensity exhibit good reactivity. 68% yield of 60a was obtained when EtOAc as solvent, however almost no reaction occurred when 80a as a reactant, whether under 365 nm irradiation or the extension of reaction time.

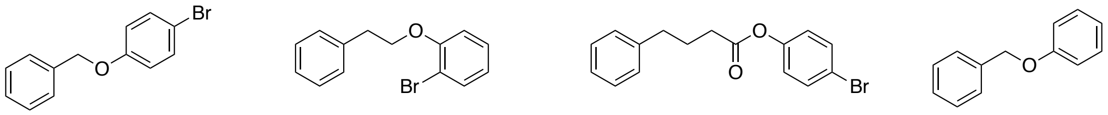

59a, 6 h, 68% (390 nm) **54a**, 24 h, 45% (365 nm) **53a**, 24 h, 40% (365 nm) **73a**, trace

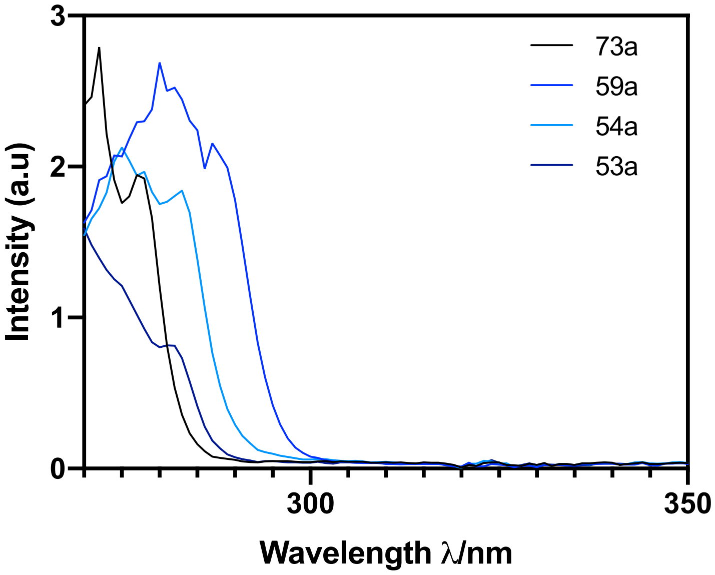

Figure S9. Absorption spectrums of different substrates in EtOAc (0.05 M).

11.2 Controlled experiments and radical inhibited experiments

11.2.1 Controlled experiments

A 10 mL quartz tube equipped with magnetic stir bar was charged with 4-bromoethylbenzene (**1**, 0.5 mmol) and EtOAc (1.5 mL). The reaction tube was sealed and pumped vacuum, then feeding nitrogen to keep pure nitrogen atmosphere with N_2 balloon, then was performed under irradiation of light (30 W 390 nm Kessil lamps) at room temperature for 12 h. Or the reaction tube was sealed and pumped vacuum, then feeding oxygen to keep pure oxygen atmosphere with O_2 balloon, then was performed under darkness at room temperature for 12 h. After the reaction completed, TLC (PE/EA = 10:1) and ^{1}H NMR showed that no desired product **2** was detected.

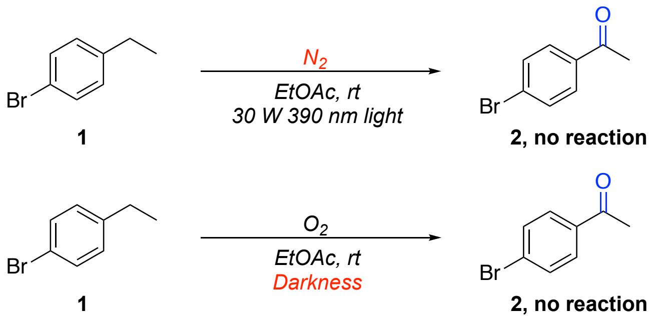

$^{18}O_2$ experiment was carried out to understand the source of oxygen atoms in the product. The mixture of reaction was analyzed by GC-MS to detect the ^{18}O-labeled product 2 (Figure S10).

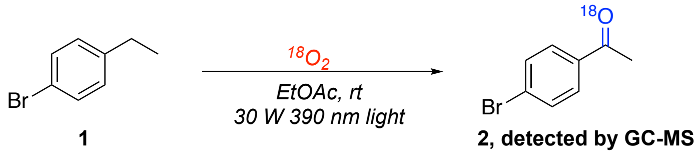

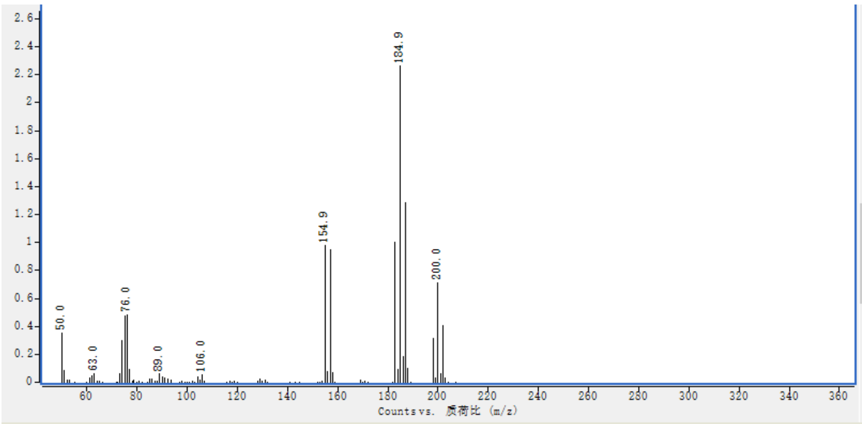

Figure S10. MS of 2-^{18}O.

11.2.2 Radical inhibited experiments

A 10 mL quartz tube equipped with magnetic stir bar was charged with 4-bromoethylbenzene (1, 0.5 mmol) and EtOAc (1.5 mL). A series of inhibitors were added to the model reaction of 1. The reaction tube was sealed and pumped vacuum, then feeding oxygen to keep pure oxygen atmosphere with O_2 balloon, then was performed under darkness at room temperature for 12 h. After the reaction completed, the yield was analyzed by ^{1}H NMR. As shown in table S8, the reactions were suppressed obviously.

Table S8. The inhibitor experiments[a]

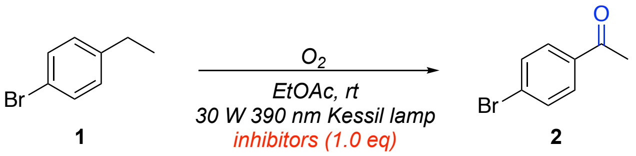

Entry	Inhibitor	Species	Yield [%][b]
1	TEMPO	radical	NR
2	BHT	radical	18
3	Anthracene	$^1O_2$	27
4	DABCO	$^1O_2$	NR
5	Benzoquinone	$O_2^{\cdot-}$	NR
6	DDQ	electron	24
7	$AgNO_3$	electron	30
8	/	/	82

[a]Reaction conditions: 4-bromoethylbenzene (1, 0.50 mmol), EtOAc (1.5 mL), and inhibitor (1.0 eq) with the oxygen atmosphere by O_2 balloon under the irradiation of 30 W 390 nm Kessil lamp for 12 h at the room temperature. [b]Yield was determined by ^{1}H NMR with the internal standard CH_3NO_2.

11.2.3 Radical trapping experiment

To determine the mechanism of the reaction, 2,2,6,6-tetramethylpiperidine-1-oxyl (TEMPO) was added in the standard conditions to capture the free radical species involved in the reaction, and the desired product 2 and 11 were not obtained but the benzylic radical with TEMPO was observed by mass spectrometry (HRMS, Figure S11 and S12).

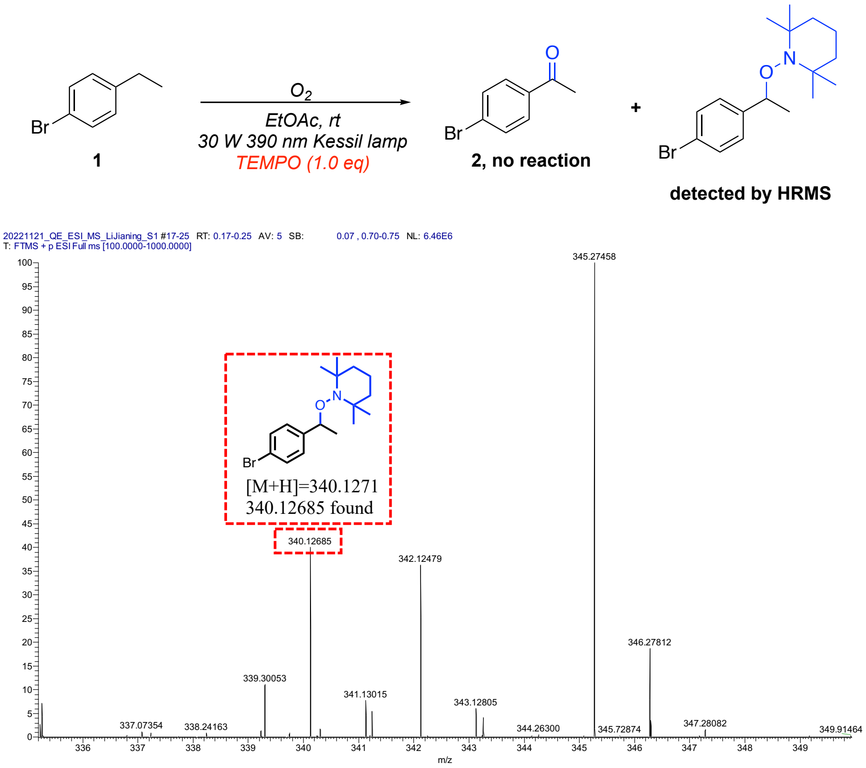

Figure S11. The radical of 4-bromoethylbenzene trapped by TEMPO

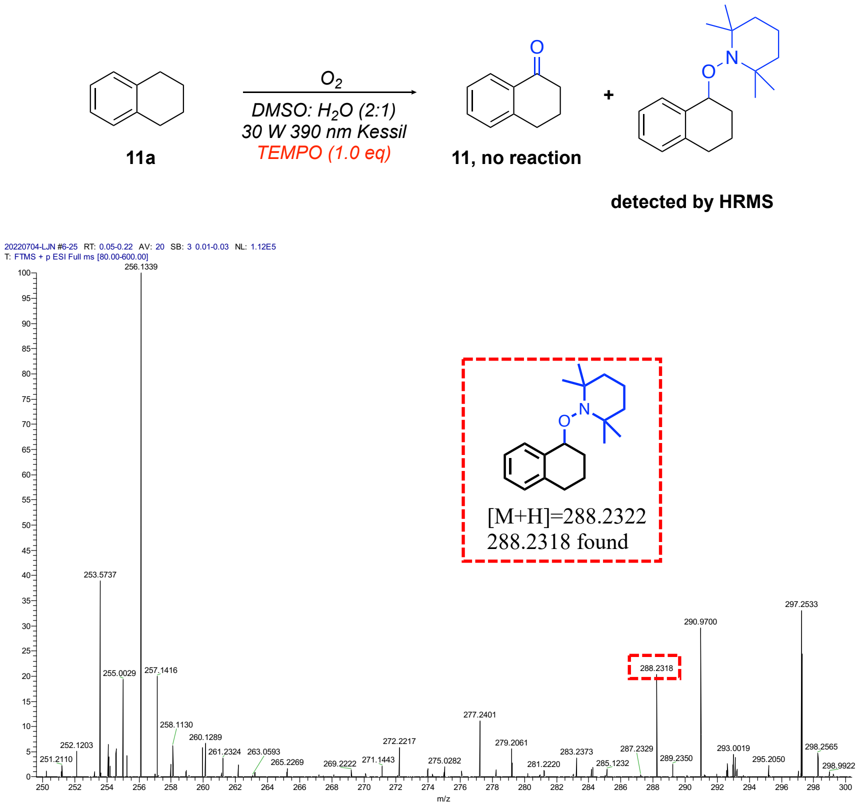

Figure S12. The radical of tetrahydronaphthalene trapped by TEMPO

11.2.4 Generation of benzylic bromide

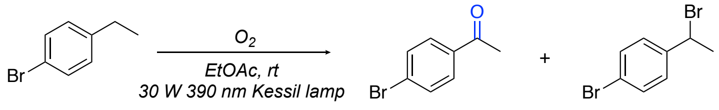

1 **2** 82% yield **2b** 11% yield

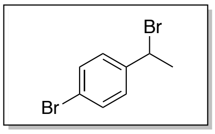

1-bromo-4-(1-bromoethyl)benzene (2b)[48]: The product was purified by column chromatography on silica gel (10:1, petroleum ether/ethyl acetate), colorless oil, 14.4 mg, 11% yield.

^{1}H NMR (500 MHz, Chloroform-*d*) δ 7.47 (d, *J* = 8.5 Hz, 2H), 7.31 (d, *J* = 8.5 Hz, 2H), 5.15 (q, *J* = 6.9 Hz, 1H), 2.02 (d, *J* = 6.9 Hz, 3H).

^{13}C NMR (101 MHz, Chloroform-*d*) δ 142.28, 131.84, 128.52, 122.19, 48.22, 26.70.

In our previous study[49], we found that the dehalogenation of aryl halides under the excitation of light, where the generated bromine radical could couple with the benzylic radical to form benzyl bromide **2b** (Scheme S1).

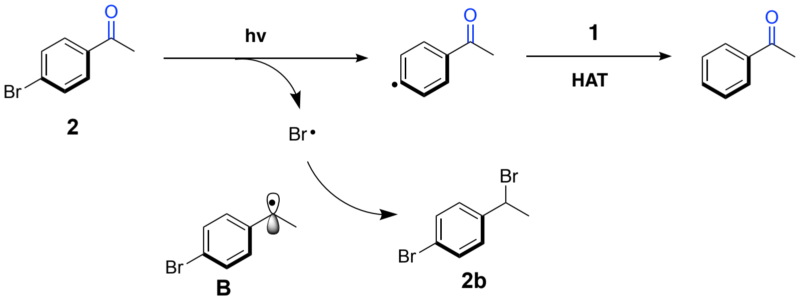

Scheme S1. The possible pathway of benzyl bromide generation

11.3 Oxygen type determination by EPR

In order to determine the active species of oxygen involved in the oxidative reaction, 5,5-dimethyl-pyrroline-N-oxide (DMPO) and 2,2,6,6-tetramethylpiperidine (TEMP) were used to capture superoxide radicals ($O_2 \cdot^-$) and singlet oxygen ($^1O_2$).

11.3.1 Determination of singlet oxygen

When TEMP (1.0 eq) was added into a solution of 4-bromoethylbenzene **1** in EtOAc in the absence of light irradiation (Figure S13, a), there was no signal, indicating that the light source was the key role in the reaction. Under the irradiation of white LED, there was a strong characteristic signal of $^1O_2$ adduct with TEMP (Figure S13, b). When the irradiation time was prolonged, a stronger characteristic signal of $^1O_2$ was detected (Figure S13, c), indicating the formation of $^1O_2$ in the reaction.

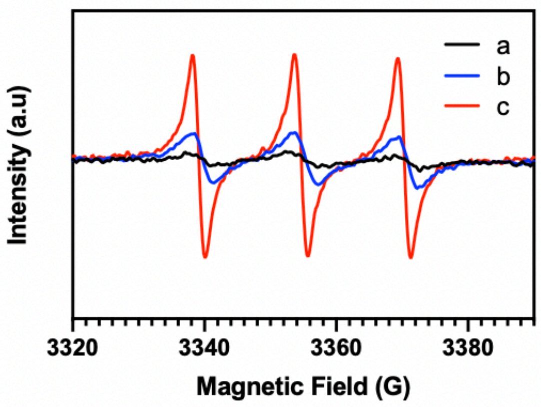

Figure S13. Singlet oxygen signal of EPR spectra. (a) a solution of 1.0 eq TEMP with 4-bromoethylbenzene **1** in EtOAc without light irradiation. (b) a solution of 1.0 eq TEMP with 4-bromoethylbenzene **1** in EtOAc under white LED irradiation for 1 min. (c) a solution of 1.0 eq TEMP with 4-bromoethylbenzene **1** in EtOAc under white LED irradiation for 2 min.

Interestingly, there was also an obvious signal when only TEMP was added into EtOAc without 4-bromoethylbenzene **1** (Figure S14), which indicated O_2 could be converted to $^1O_2$ under the irradiation.

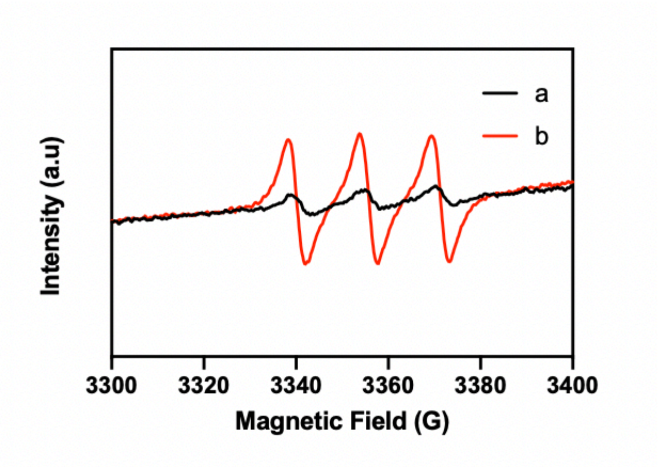

Figure S14. Singlet oxygen signal of EPR spectra without 4-bromoethylbenzene **1**. (a) a solution of 1.0 eq TEMP in EtOAc without light irradiation. (b) a solution of 1.0 eq TEMP in EtOAc under white LED irradiation for 1 min.

11.3.1 Determination of superoxide radical

Similarly, there was no signal when DMPO (1.0 eq) was added into a solution of 4-bromoethylbenzene 1 in EtOAc in the absence of light irradiation (Figure S15, a). Irradiation of EtOAc solution of DMPO and bromoethylbenzene 1 in air under white LED for 1 min resulted in the observation of a strong characteristic signal of $O_2{\cdot}^-$ adduct with DMPO. When the irradiation time was prolonged, a stronger characteristic signal of $O_2{\cdot}^-$ was observed (Figure 14, c), indicating the formation of $O_2{\cdot}^-$ in the reaction.

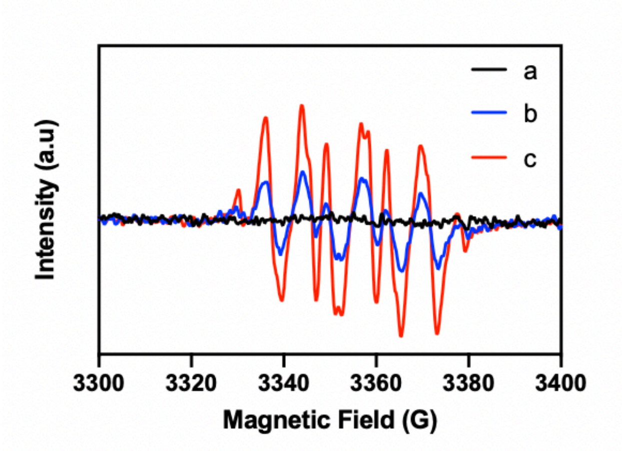

Figure S15. Superoxide radical signal of EPR spectra. (a) a solution of 1.0 eq DMPO with 4-bromoethylbenzene **1** in EtOAc without light irradiation. (b) a solution of 1.0 eq DMPO with 4-bromoethylbenzene **1** in EtOAc under white LED irradiation for 1 min. (c) a solution of 1.0 eq

DMPO with 4-bromoethylbenzene **1** in EtOAc under white LED irradiation for 2 min.

11.4 Switch on-off experiment

A 10 mL quartz tube equipped with magnetic stir bar was charged with 4-bromoethylbenzene **2a** (0.5 mmol) and EtOAc (1.5 mL). The reaction tube was sealed and pumped vacuum, then feeding oxygen to keep pure oxygen atmosphere with O_2 balloon. The resulting mixture was performed under irradiation of light (30 W 390 nm Kessil lamps) at room temperature. After 3 h of light irradiation, an aliquot portion was taken from the reaction mixture and further analyzed by ^{1}H NMR to obtain the conversion of product 2 using CH_3NO_2 as the internal standard. Then, the reaction mixture was sealed and pumped vacuum again, feeding oxygen to keep pure oxygen atmosphere with O_2 balloon. Thereafter, the light was switched off with continuous stirring for 2 h. After that, an analytical sample solution was prepared and analysed similarly, and the light was switched on for 3 h. This cycle was repeated and the conversion of 2 with respect to reaction time was plotted (Figure S16). The result of light on-off experiment indicated the reaction was induced by the light irradiation.

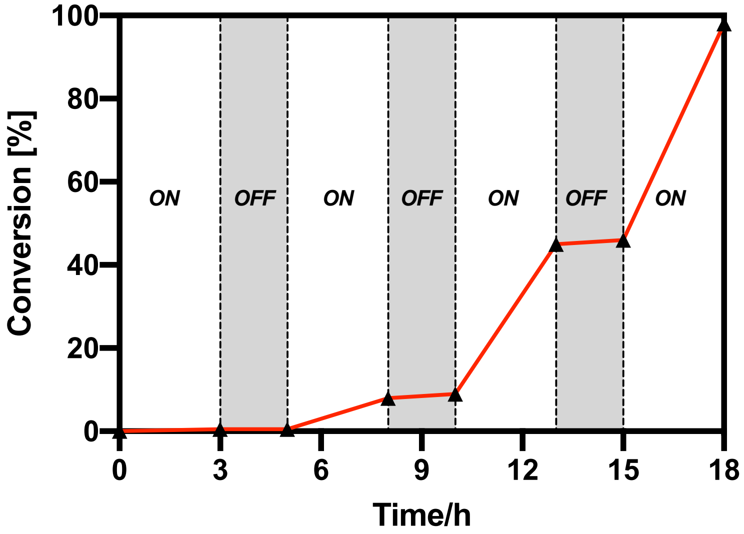

Figure S16. Switch on-off experiment

11.5 Hydrogen peroxide detection experiment

Following the general procedure A, taking 4-bromoethylbenzene **1** for example. After the reaction finished, the mixture of reaction was obtained. When 0.5 mL of NaI (aq, 0.5 M) was added into the mixture of reaction, the color of the reaction was deeper, due to the oxidation of I^- by H_2O_2 (Table S17). In addition, ^1H NMR was also used to demonstrate the formation of hydrogen peroxide (H_2O_2) in the reaction (Table S18). A peak for hydrogen peroxide was detected, which was in agreement with literature value[14]. H_2O_2: ^1H NMR (400 MHz, CD_3CN) δ 8.62 (s, 2H).

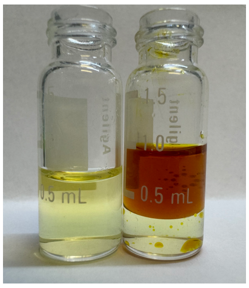

Figure S17. The initial mixture of reaction (left) and the addition of NaI aq. (ringht)

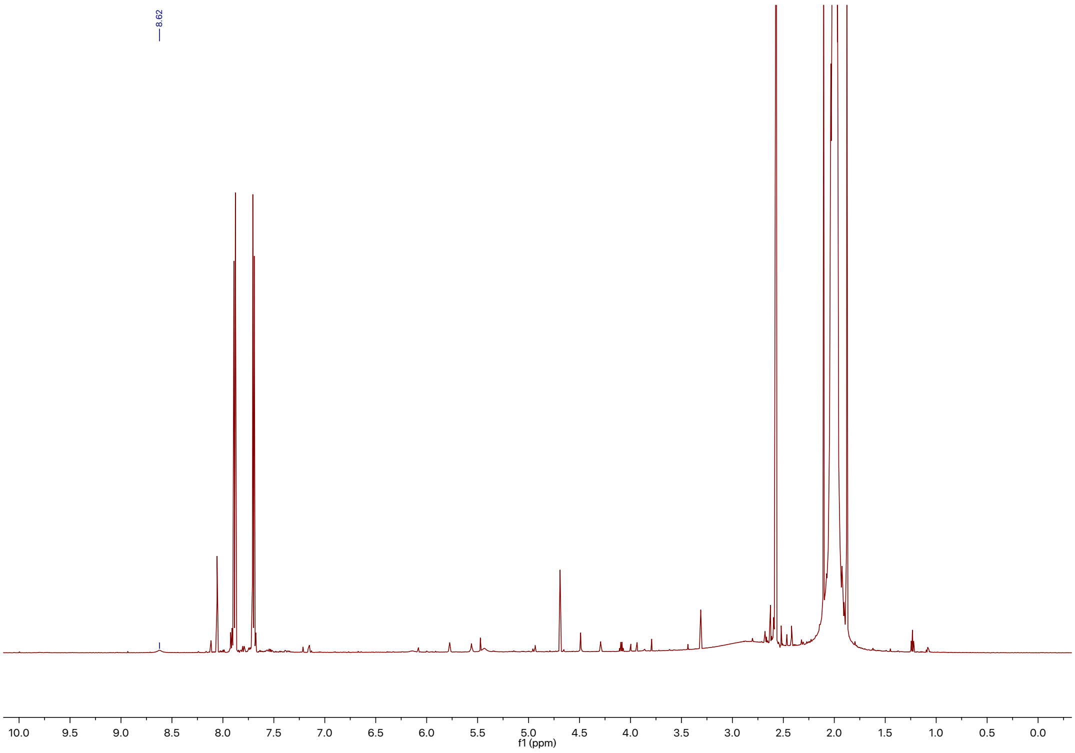

Figure S18. ^1H NMR of hydrogen peroxide in the reaction

11.6 Benzylic oxidation catalyzed by aromatic ketones as photocatalyst

A 10 mL quartz tube was charged with ethylbenzene (0.5 mmol), aromatic ketones (0.5 mmol, 1.0 eq) and a magnetic stirring bead. EtOAc (1.5 mL) was added as the solvent. The tube was sealed and pumped vacuum, then feeding oxygen to keep pure oxygen atmosphere with O_2 balloon. The reaction tube was placed on the magnetic stirrer under the irradiation of a Kessil lamp (390 nm) equipped with a compact fan kit for maintaining the room temperature. After 18 h, an aliquot from the mixture was analyzed in GC with cyclohexanone as internal standard (Table S9).

When the product acetophenone was added to the reaction, only 8% yield of extra product was obtained, indicating that the aromatic ketone could indeed induce the reaction (entry 13). Thereafter, the reaction was proceeded smoothly when the substituent group R_2 on aromatic rings as the electron-withdrawing group, such as 4-NO_2, 4-CN and 4-SO_2Me (entry 4-6). However, no desired product was acquired when the substituent group R_2 as the electron-donating group, such as 4-OMe (entry 1). Among the acetophenone substituted by halogen atom, p-bromoacephenone had a good performance on the benzylic oxidation (entry 2-3, 7-8, 12). Furthermore, the substituent R_1 was also investigated. The yield of the product was 24-51% when R_1 was substituted by -CF_3 and cyclohexyl group, or 1-tetralone and 4-bromobenzaldehyde as photocatalyst (entry 9-10, 14, 16). Compared to acetophenone as photocatalyst, the yield was increased to 42% when aromatic ring was pyridine (entry 15), probably because of the weak density of electron on pyridine. Based on the above results, we suggested that the electron-withdrawing effect of the aromatic ring was critical in this reaction, presumably the interaction between aromatic ketone and ethylbenzene, thereby promoting the formation of CT complexes. Moreover, when the equivalent of p-bromoacephenone was decreased to 0.2 eq, the reaction performed with 71% yield (entry 17), which was similar with the common ketone photocatalysts, such as benzophenone, anthraquinone and thioxanthen-9-one (entry 11, 18-19).

Table S9. The aromatic ketones screening[a]

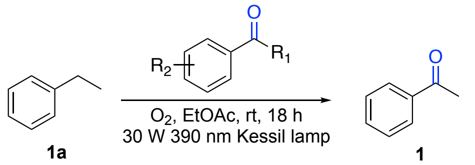

Entry	Ar-R_2	R_1	Conv. [%]	Yield [%][b]
1	4-OMe-Ph	Me	trace	/

2	4-F-Ph	Me	36	17
3	4-Br-Ph	Me	99	73, 71[d]
4	4-NO_2-Ph	Me	78	46
5	4-CN-Ph	Me	86	53
6	4-SO_2Me-Ph	Me	30	16
7	2-Br-Ph	Me	99	48
8	3-Br-Ph	Me	99	53
9	Ph	CF_3	53	29
10	Ph	Cy	50	24
11	Ph	Ph	85	65
12	3,4,5-F-Ph	Me	59	34
13	Ph	Me	29	8
14	4-Br-Ph	H	65	51
15	Py	Me	71	42
16	1-Tetralone		89	46
17[c]	4-Br	Me	99	71
18[c]	Anthraquinone		99	75
19[c]	Thioxanthen-9-one		99	69

[a]Reaction conditions: ethylbenzene (0.5 mmol), aromatic ketones (0.5 mmol, 1.0 eq), EtOAc (1.5 mL) with the oxygen atmosphere by O_2 balloon under the irradiation of 30 W 390 nm Kessil for 18 h at the room temperature. [b]Yield was determined by GC-FID with the internal standard cyclohexanone. [c]20 mol% aromatic ketone. [d]isolated yield.

11.7 KIE experiment

The cleavage of benzylic C-H bonds is involved in the benzylic carbonylation reaction, thereby generating the key benzylic radical. Thus, the KIE experiment was performed in order to investigate whether the cleavage of benzylic C-H bonds is the rate-determining step.

11.7.1 Synthesis of the deuterated substrates[15]

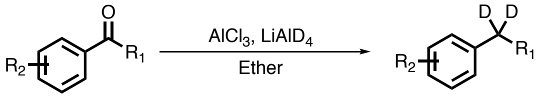

Following a reported procedure: a flame-dried vial was charged with water free $AlCl_3$ (1180 mg, 8.84 mmol, 1.77 equiv.) and lithium aluminum deuteride (210 mg, 5.00 mmol, 1.0 equiv.) under nitrogen atmosphere. The mixture was carefully suspended in dry ether (8 mL). Aromatic ketones (5.0 mmol, 1.0 equiv.) were carefully added as solid (violent reaction) to the suspension. The mixture was stirred for 3 h at room temperature, diluted with ether (20 mL) and quenched by the addition of aqueous HCl (1 M). The phases where separated, and the aqueous phase was extracted with ether (3 x 10 mL). The organic phase was dried over sodium sulfate, filtered, and concentrated. The product was purified by column chromatography on silica gel.

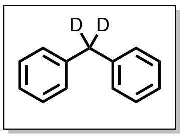

Diphenylmethane-d2: Following the above procedure. The product was purified by column chromatography on silica gel (10:1, petroleum ether/ethyl acetate), colorless oil, 0.72 g, 85% yield.

^{1}H NMR (400 MHz, $CDCl_3$) δ 7.31 – 7.24 (m, 5H), 7.19 – 7.17 (m, 5H).

^{13}C NMR (101 MHz, $CDCl_3$) δ 141.11, 128.98, 128.52, 126.14.

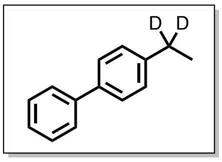

4-(ethyl-1,1-d2)-1,1'-biphenyl: Following the above procedure. The product was purified by column chromatography on silica gel (10:1, petroleum ether/ethyl acetate), white solid, 0.65 g, 71% yield.

^{1}H NMR (400 MHz, $CDCl_3$) δ 7.58 (d, J = 7.7 Hz, 2H), 7.52 (d, J = 7.9 Hz, 2H), 7.42 (t, J = 7.5 Hz, 2H), 7.30 (dd, J = 21.4, 7.6 Hz, 3H), 1.26 (s, 3H).

^{13}C NMR (101 MHz, $CDCl_3$) δ 143.36, 141.22, 138.64, 128.74, 128.32, 127.11, 127.05, 127.00, 15.48.

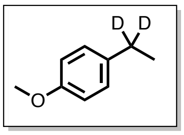

1-(ethyl-1,1-d2)-4-methoxybenzene: Following the above procedure. The product was purified by column chromatography on silica gel (10:1, petroleum ether/ethyl acetate), colorless oil, 0.58 g, 85% yield.

^{1}H NMR (400 MHz, $CDCl_3$) δ 7.34 – 7.02 (m, 2H), 6.92 – 6.69 (m, 2H), 3.78 (s, 3H), 1.19 (s, 3H).

^{13}C NMR (101 MHz, $CDCl_3$) δ 157.63, 136.36, 128.71, 113.75, 55.29, 27.29, 15.74.

11.7.2 KIE study

The procedure is the same as A. Initially, diphenylmethane-d2 as reactive substrate was oxidated under the irradiation of 390 nm Kessil lamp with oxygen in the absence of catalysts. However, the reaction did not conduct at all, because of the better stability of C-D bond than C-H bond. Thus, the subsequent KIE study would use p-bromoacephenone as the initiator.

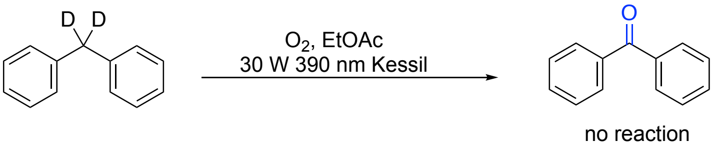

Similarly, the parallel KIE study of diphenylmethane-d2 was proceeded following the procedure as the same as A, except for the following changes: diphenylmethane (0.5 mmol, 1.0 eq), diphenylmethane-d2 (0.5 mmol, 1.0 eq) were used as substrates in two reaction tubes, respectively, then adding p-bromoacephenone (0.1 mmol, 20 mol%). These mixtures were reacted for 1 hours. After 20 min, 0.1 mL of aliquot was taken per 10 min and diluted with 0.5 mL CH_2Cl_2. The conversion of substrate was detected with GC-MS.

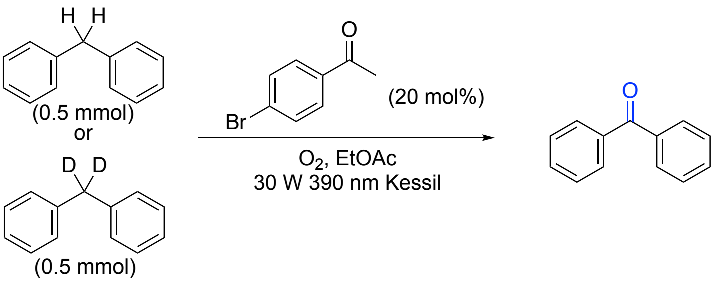

Parallel KIE = k_H/k_D= 0.52/0.295 =1.8

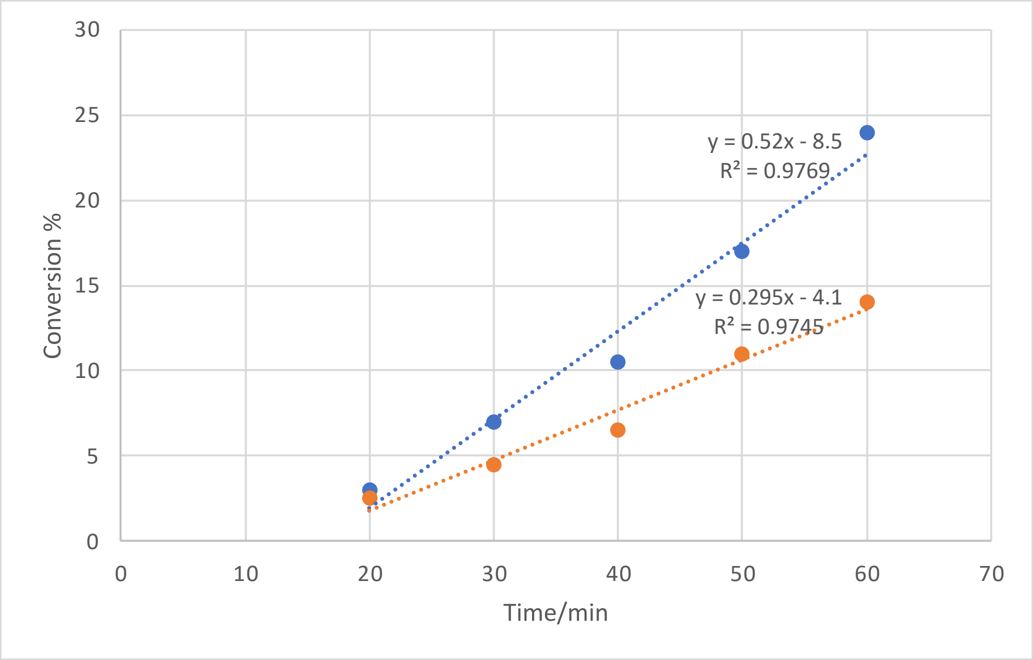

Figure S19. Parallel KIE study of diphenylmethane.

Next, we also used 4-(ethyl-1,1-d2)-1,1'-biphenyl as substrate to study the parallel KIE of 4-ethyl-1,1'-biphenyl. The reaction procedure was consistent with the previous steps. These mixtures were reacted for 24 hours. After 1 h, 50 μL of aliquot was taken per 1 h and diluted with 0.5 mL CH_2Cl_2. The conversion of substrate was detected with GC-MS.

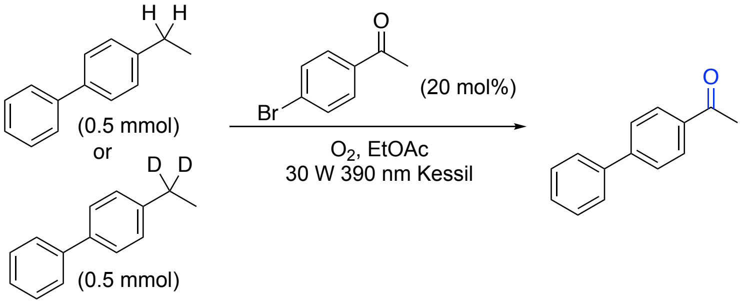

Parallel KIE = k_H/k_D= 9.6/2.3 =4.2

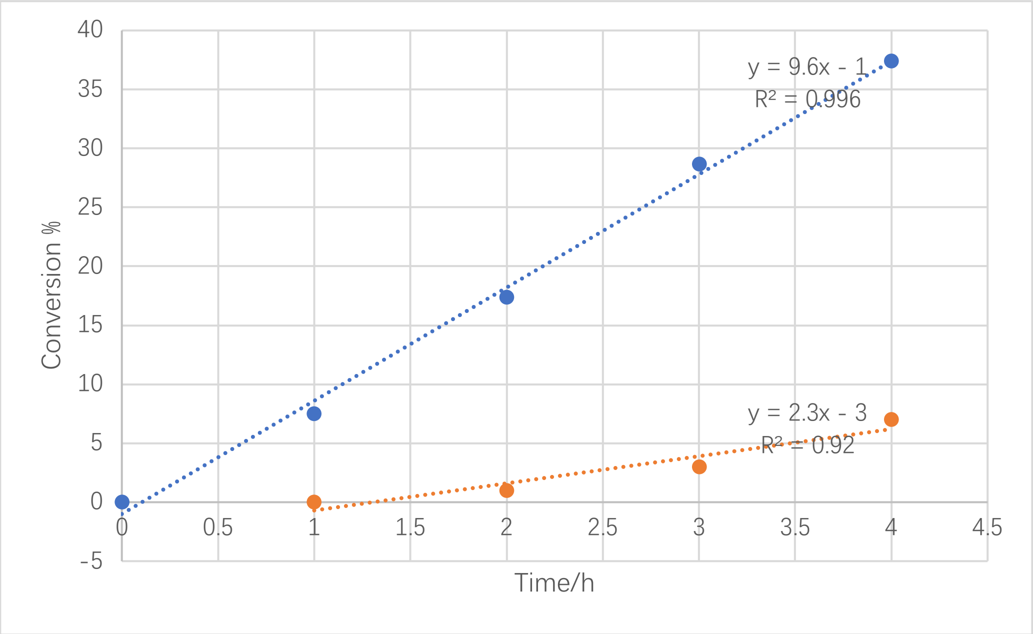

Figure S20. Parallel KIE study of 4-ethyl-1,1'-biphenyl.

For electron-donating group substituted, 1-ethyl-4-methoxybenzene was also explored. The procedure is the same as A, except for the following changes: 1-ethyl-4-methoxybenzene (0.25 mmol, 0.5 eq), 1-(ethyl-1,1-d2)-4-methoxybenzene (0.25 mmol, 0.5 eq), and p-bromoacephenone (0.1 mmol, 20 mol%) were added. After reaction for 1 h, 50 μL of aliquot was taken per 1 h or 2 h and diluted with 0.5 mL CH_2Cl_2. The conversion of substrate was detected with GC-MS..

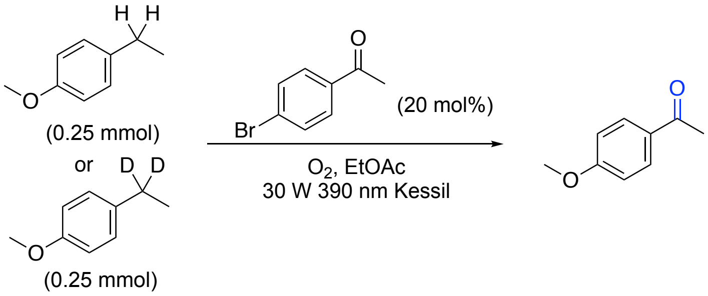

Parallel KIE = k_H/k_D= 9.2912/6.2719 =1.5

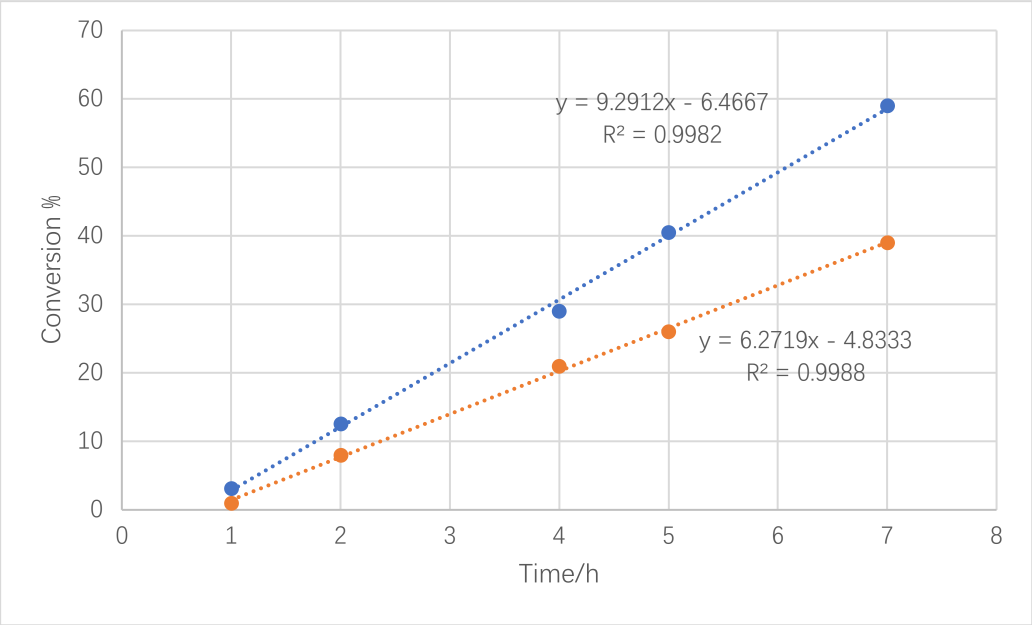

Figure S21. Parallel KIE study of 1-ethyl-4-methoxybenzene.

On the basis of the above KIE study, the parallel KIE (4.2) observed in parallel reaction of 4-ethyl-1,1'-biphenyl and 4-(ethyl-1,1-d2)-1,1'-biphenyl revealed that the cleavage of benzylic C-H bond was supposed to be a rate-determining step, causing the pathway of HAT. When diphenylmethane and diphenylmethane-d2 as substrates, the parallel KIE of 1.8 was measured, reflecting that the kinetic isotope effect was weakened. However, the KIE value observed in parallel (1.5) reaction using 1-ethyl-4-methoxybenzene and 1-(ethyl-1,1-d2)-4-methoxybenzene indicated the cleavage of benzylic C-H bond might not be a rate-determining step, but rather there might be other possible decisive steps, such as the formation of the charge-transfer complexes. Thus, due to the differences in electron-withdrawing effect of substrates, the different pathways would exist in the reaction.

To prove our conjecture, 4-nitroacetophenone was selected as the initiator and we also measured the KIE value of 1-ethyl-4-methoxybenzene and 1-(ethyl-1,1-d2)-4-methoxybenzene in parallel reaction. The procedure was same with the above steps. Obviously, the KIE of 1.3 revealed that isotope effect was weakened and the cleavage of C-H bond was not the rate-determining step, which is that 1-ethyl-4-methoxybenzene as electron donor and 4-nitroacetophenone as electron acceptor form charge-transfer complexes. Moreover, the reaction rate of 4-nitroacetophenone become faster than 4-bromoacephenone’s due to the stronger interaction between 1-ethyl-4-methoxybenzene and

4-nitroacetophenone (Figure S and S).

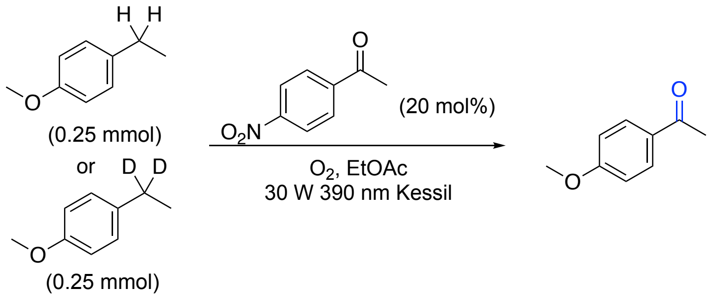

Parallel KIE = k_H/k_D= 44/33.1 =1.3

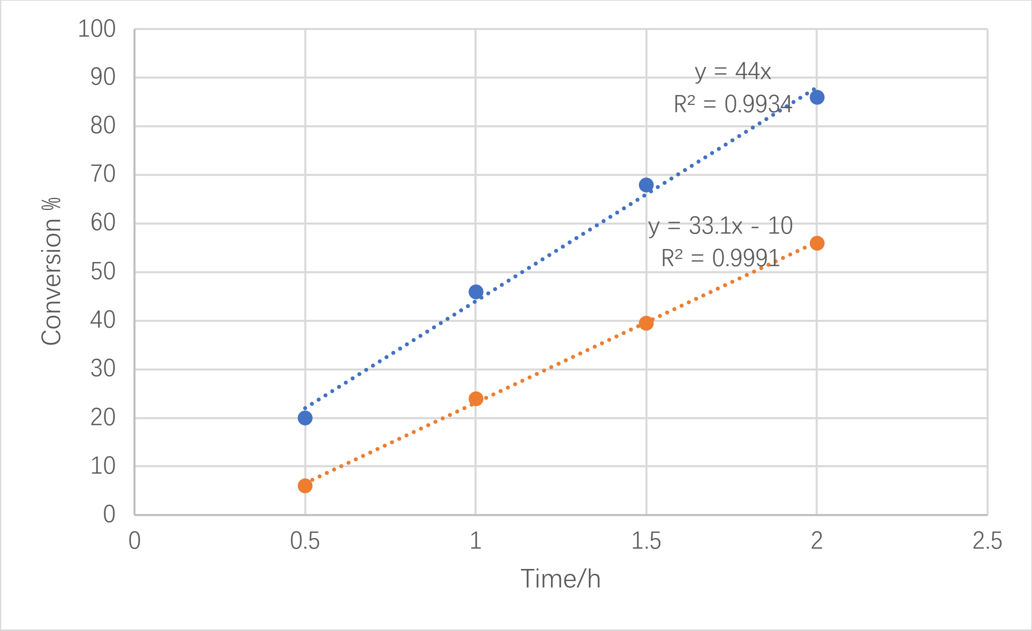

Figure S22. Parallel KIE study of 1-ethyl-4-methoxybenzene.

11.8 Competitive study

A 10 mL quartz tube was charged with 1-ethyl-4-methoxybenzene (0.25 mmol) and 4-acetylbenzonitrile (0.25 mmol), 4-bromoacephenone or 4-nitroacetophenone (0.1 mmol, 20 mol%) and a magnetic stirring bead. EtOAc (1.5 mL) was added as the solvent. The tube was sealed and pumped vacuum, then feeding oxygen to keep pure oxygen atmosphere with O_2 balloon. The reaction tube was placed on the magnetic stirrer under the irradiation of a Kessil lamp (390 nm) equipped with a compact fan kit for maintaining the room temperature. After 4 h, the reaction mixture was analyzed by ^{1}H NMR with nitromethane as the internal standard.

4-bromoacephenone as the initiator, the product ratio was found to be 16.7. While using 4-nitroacetophenone, the product ratio was 28.0 (Figure S23).

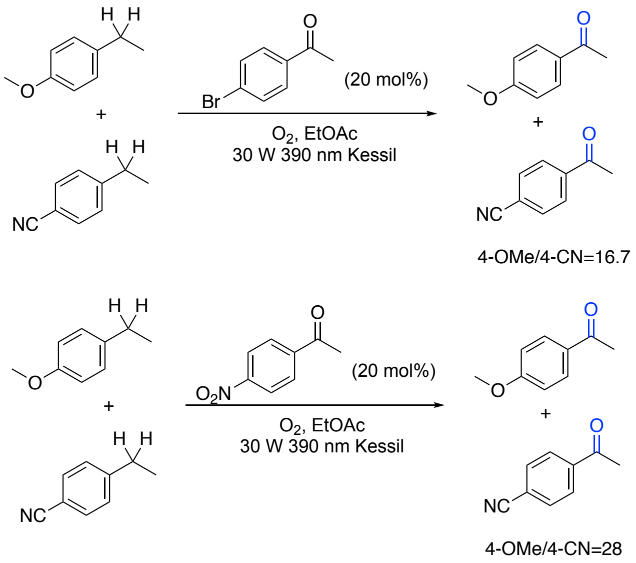

Figure S23. Competition experiments

11.9 The capture of peroxide intermediate by ^{1}H NMR

The mixture of 1-ethyl-4-methoxybenzene (0.5 mmol) and 4-nitroacetophenone (0.5 mmol) was irradiated under the 390 nm Kessil lamp for 1 h with oxygen atmosphere. Then, the mixture was analyzed by ^{1}H NMR and the characteristic hydrogen of peroxide intermediate was found, which was consistent with the reported literature (Figure S24).[16, 17]

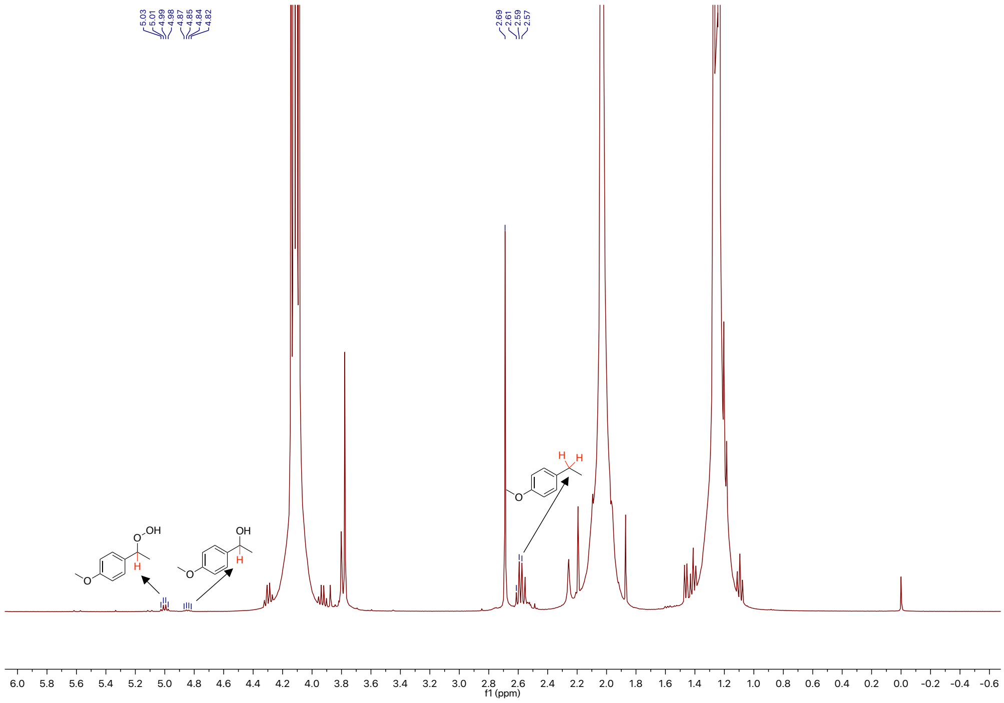

Figure S24. The ^{1}H NMR of peroxide intermediate.

11.10 Spectroscopic investigation of CT complexes

UV-vis absorption spectra of CT complexes

The UV-vis absorption spectra of **1** under different atmosphere (O_2/N_2) were measured using a UV-vis absorption spectrophotometer (Figure S25). Due to the operation of gas bubbling, the volatilization of solvents could lead to changes in **1** concentration, resulting in inaccurate spectra. Thus, we measured the absorption spectra of pure **1** under different atmosphere. The **1** was degassed by N_2 bubbling for 30 minutes before measuring, then, the absorption spectra of **1** in N_2 was recorded. Similarly, the absorption spectras of **1** by O_2 bubbling for 10/20/30 minutes were also recorded. The red shift was observed after O_2 bubbling, and the absorption spectra after O_2 bubbling for 10 minutes had no change due to oxygen saturation.

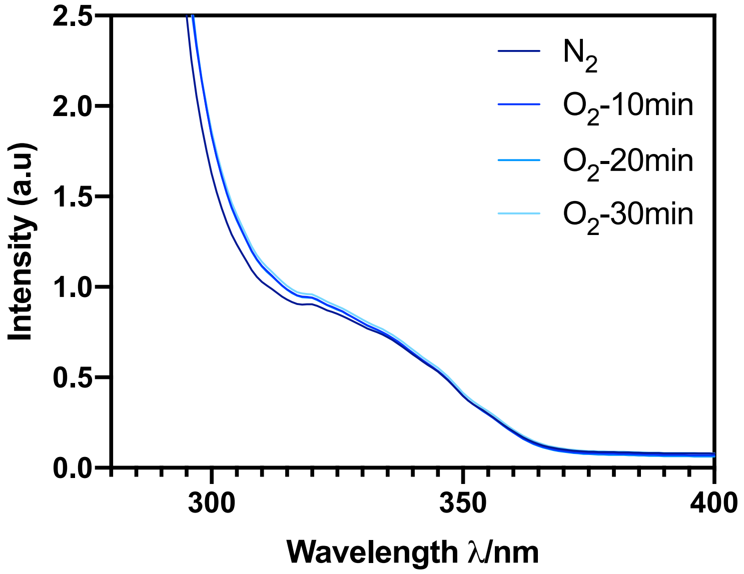

Figure S25. The UV-vis absorption spectra of **1** under O_2/N_2 atmosphere

Transient absorption (TA) spectra

Femtosecond transient absorption (fs-TA) spectra were collected using commercial femtosecond transient absorption spectrometer (Helios, Ultrafast System). A 310 nm pulse was used as the actinic pump for the TAS experiment. The samples were prepared with 4-bromoethylbenzene 1 without any solvents with N_2/O_2 atmosphere before being into 1 mm pathlength cuvettes for the experiment. In N_2 atmosphere, the two-dimensional transient absorption spectrum shown a broad excited state absorption which was assigned to the quenched excited state absorption of 1 (Figure S26).

Compared to the TA spectrum of 1 with N_2, a new peak (~410 nm) was displayed in TA spectrum of 1 with O_2 (Figure S27, right), which might be assigned to the interaction of 1 and oxygen. Then, the lifetime was analyzed through kinetic curves of 1 at ~ 410 nm after fitting. A lifetime in the oxygen atmosphere (542.1 fs, Figure S29) was shorter than in the N_2 atmosphere (650.4 fs, Figure S28) because of the quenching behavior of ground state oxygen[18].

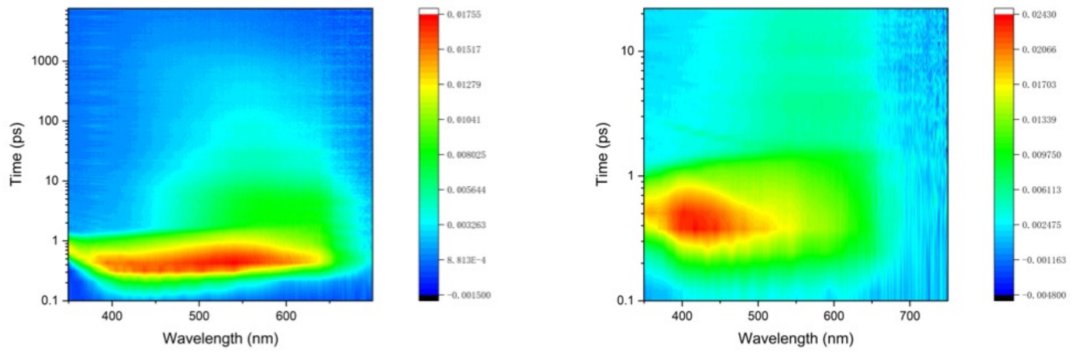

Figure S26. Two-dimensional transient absorption spectrum of **1** in N_2 (left) and O_2 (right) atmosphere.

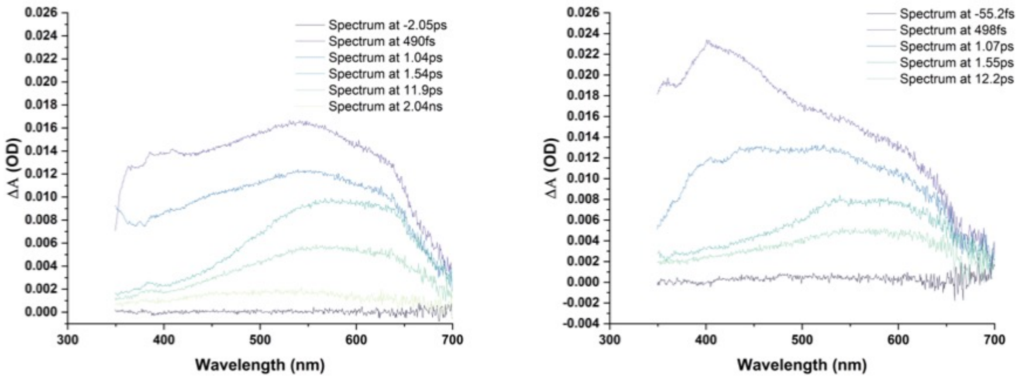

Figure S27. TA spectrum of **1** in N_2 (left) and O_2 (right) atmosphere at selected time delays.

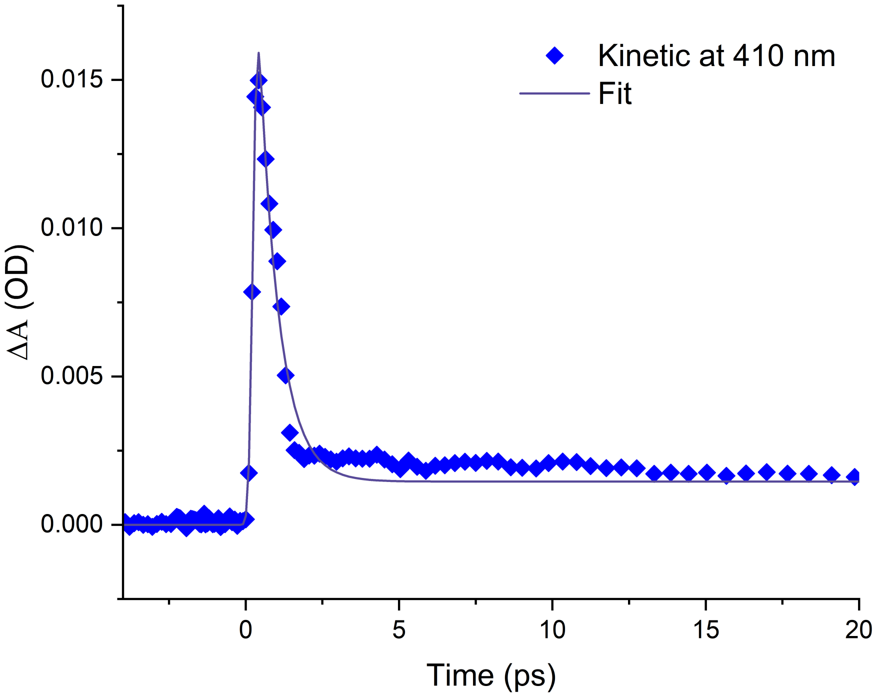

Figure S28. The lifetime (650.4 fs) of **1** in N_2 at 410 nm.

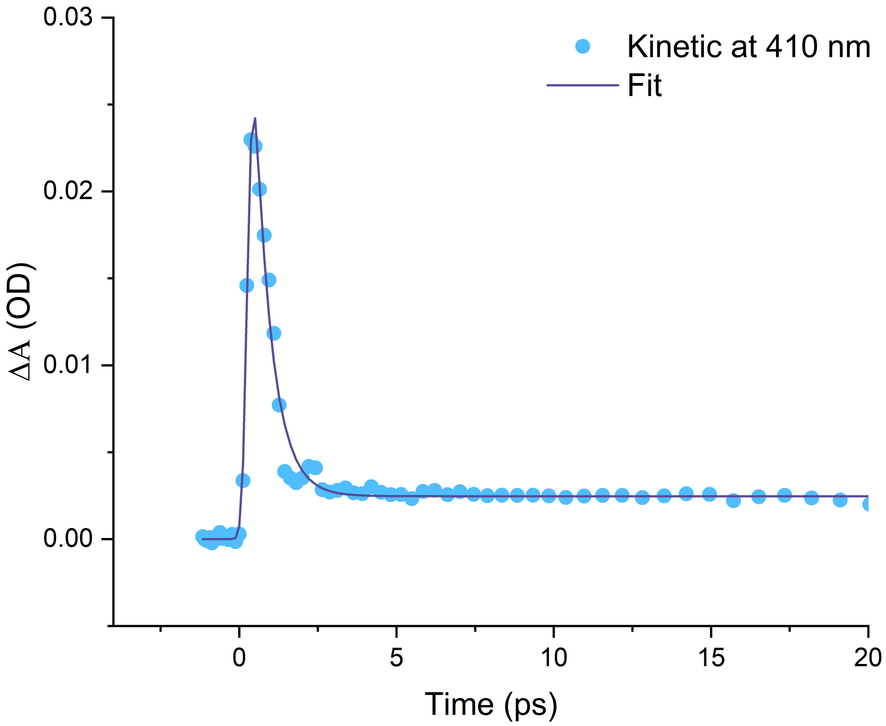

Figure S29. The lifetime (542.1 fs) of **1** in O_2 at 410 nm.

12. The synthetic application

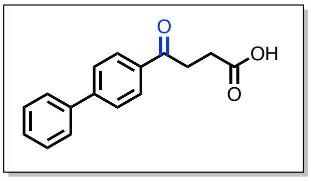

Fenbufen (62)[21]: The product was purified by column chromatography on silica gel (3:1, petroleum ether/ethyl acetate), white solid, 30.4 mg, 60% yield.

^{1}H NMR (400 MHz, $CDCl_3$) δ 8.09 – 8.02 (m, 2H), 7.73 – 7.66 (m, 2H), 7.63 (dt, *J* = 6.0, 1.3 Hz, 2H), 7.47 (dd, *J* = 8.2, 6.6 Hz, 2H), 7.43 – 7.38 (m, 1H), 3.36 (t, *J* = 6.5 Hz, 2H), 2.85 (t, *J* = 6.5 Hz, 2H).

^{13}C NMR (101 MHz, $CDCl_3$) δ 197.51, 177.05, 146.07, 139.84, 135.09, 128.98, 128.68, 128.30, 127.32, 127.30, 33.26, 27.86.

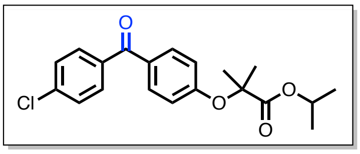

Fenofibrate (63)[29]: The product was purified by column chromatography on silica gel (3:1, petroleum ether/ethyl acetate), white solid, 46.8 mg, 65% yield.

^{1}H NMR (400 MHz, $CDCl_3$) δ 7.75 – 7.72 (m, 2H), 7.72 – 7.68 (m, 2H), 7.45 (d, *J* = 8.5 Hz, 2H), 6.87 (d, *J* = 8.8 Hz, 2H), 5.10 (h, *J* = 6.3 Hz, 1H), 1.66 (s, 6H), 1.21 (d, *J* = 6.2 Hz, 6H).

^{13}C NMR (101 MHz, $CDCl_3$) δ 194.28, 173.11, 159.75, 138.36, 136.44, 131.96, 131.17, 130.23, 128.55, 117.26, 79.43, 69.36, 25.38, 21.53.

Synthesis of acetosyringone

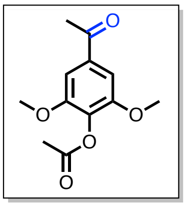

4-acetyl-2,6-dimethoxyphenyl acetate (64): The product was purified by column chromatography on silica gel (3:1, petroleum ether/ethyl acetate), white solid, 47.5 mg, 40% yield.

^{1}H NMR (500 MHz, $CDCl_3$) δ 7.26 (s, 2H), 3.89 (s, 6H), 2.61 (s, 3H), 2.36 (s, 3H).

^{13}C NMR (126 MHz, $CDCl_3$) δ 196.82, 168.22, 152.26, 135.16, 132.88, 105.15, 56.35, 26.55, 20.45.

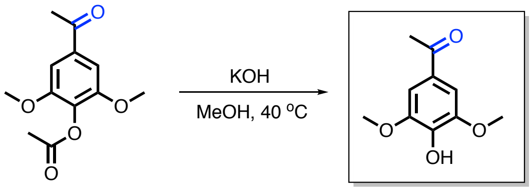

A mixture of 64 (0.2 mmol, 1.0 eq), KOH (0.24 mmol, 1.2 eq) and MeOH (2.0 mL) was heated to 40°C for 2 h. The solution was cooled to room temperature, and neutralized with conc. HCl(aq). The acidified mixture was poured into water (10 mL) and extracted with EtOAc (15 mL *3). The combined organic layers were dried over Na_2SO_4, and the solvent evaporated under reduced pressure. The resulting residue was purified by flash silica gel column chromatography to yield the desired products acetosyringone.

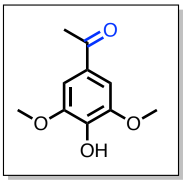

Acetosyringone (65)[43]: Following the above procedure. The product was purified by column chromatography on silica gel (3:1, petroleum ether/ethyl acetate), white solid, 36.0 mg, 92% yield.

^1H NMR (500 MHz, $CDCl_3$) δ 6.45 (s, 2H), 3.81 (s, 6H), 2.62 (q, J = 7.6 Hz, 2H), 2.33 (s, 3H), 1.25 (t, J = 7.6 Hz, 3H).

^{13}C NMR (101 MHz, $CDCl_3$) δ 196.67, 146.75, 139.75, 128.81, 105.75, 56.48, 26.27.

Synthesis of 69[19]

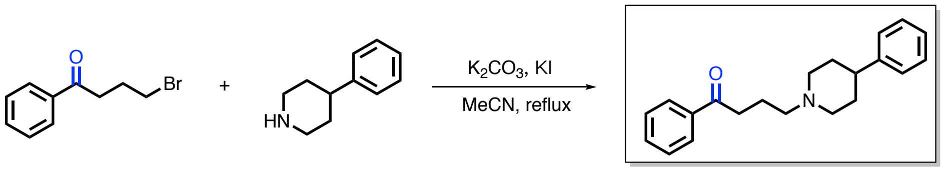

A 5 mL round bottom flask was charged with a stir bar, **58** (0.2 mmol, 1.0 eq), K_2CO_3 (0.26 mmol, 1.3 eq), KI (0.02 mmol, 0.1 eq), 1-aryl -4-bromobutane (0.24 mmol, 1.2 eq), and MeCN (2.0 mL). The reaction mixture was stirred rapidly and heated at 80 °C overnight. The reaction mixture was allowed to cool to room temperature, Then, the reaction mixture was poured into water (10 mL) and extracted with EtOAc (15 mL *3). The solvent was removed to give the crude product, which was purified by flash column chromatography on silica gel to give the compound.

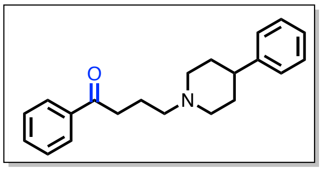

1-phenyl-4-(4-phenylpiperidin-1-yl)butan-1-one (69): Following the above procedure. The product was purified by column chromatography on silica gel (3:1, petroleum ether/ethyl acetate), white solid, 46.0 mg, 75% yield.

^{1}H NMR (400 MHz, $CDCl_3$) δ 7.99 (d, J = 7.5 Hz, 2H), 7.55 (t, J = 7.3 Hz, 1H), 7.46 (t, J = 7.6 Hz, 2H), 7.28 (q, J = 6.2, 4.8 Hz, 2H), 7.19 (d, J = 7.7 Hz, 3H), 3.03 (q, J = 7.1, 6.0 Hz, 4H), 2.46 (q, J = 7.3, 5.7 Hz, 3H), 2.16 – 1.93 (m, 4H), 1.88 – 1.61 (m, 4H).

^{13}C NMR (126 MHz, $CDCl_3$) δ 200.09, 146.44, 137.20, 132.90, 128.57, 128.40, 128.13, 126.88, 126.12, 58.17, 54.29, 42.70, 36.44, 33.36, 21.87.

HRMS $[M+H]^+$ calcd for $[C_{21}H_{26}NO]^+$: 308.2014; found: 308. 2012.

Synthesis of 70

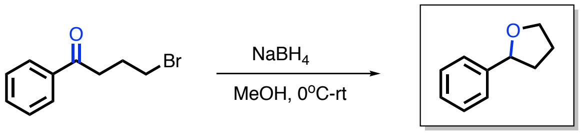

To a 25 mL bottom flask, **58** (0.2 mmol, 1.0 eq) was dissolved in MeOH (4 mL) and cooled to 0-5 ºC. $NaBH_4$ (0.2 mmol, 1.0 eq) was added in fractions to the above solution under stirring in such a manner that the temperature of the reaction mixture did not rise above 10 ºC. The reaction mixture was stirred 3 h at room temperature. MeOH was removed from the reaction mixture and cold water (10 mL) was added to it. After quenching with water, then reaction mixture was extracted with EtOAc for 3 times. The combined organic phase was concentrated in vacuo and the residue was purified by by flash column chromatography on silica gel to give the desired product.

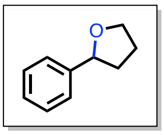

2-phenyltetrahydrofuran (70)[44]: Following the above procedure. The product was purified by column chromatography on silica gel (50:1, petroleum ether/ethyl acetate), colorless oil, 19.3 mg, 85% yield.

^{1}H NMR (400 MHz, $CDCl_3$) δ 7.29 (dd, J = 32.5, 4.2 Hz, 5H), 4.89 (t, J = 7.2 Hz, 1H), 4.10 (dt, J = 8.2, 6.8 Hz, 1H), 3.94 (td, J = 7.9, 6.4 Hz, 1H), 2.32 (dtd, J = 12.3, 7.1, 5.4 Hz, 1H), 2.09 – 1.93 (m, 2H), 1.90 – 1.75 (m, 1H).

^{13}C NMR (126 MHz, $CDCl_3$) δ 143.46, 128.29, 127.12, 125.64, 80.68, 68.67, 34.61, 26.04.

Synthesis of 71

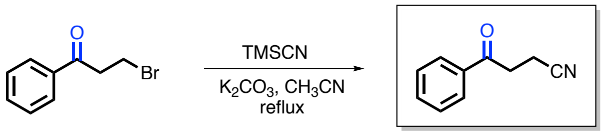

57 (0. 2 mmol, 1.0 eq) was added a CH_3CN solution of Me_3SiCN (0.24 mmol, 1.2 eq) and K_2CO_3 (0.24 mmol, 1.2 eq). The resultant mixture was further heated at 80 °C for 24 hours. The reaction was quenched with aq Na_2CO_3 (10 mL). The aqueous layer was extracted with EtOAc (3*15 mL), and the combined organic phases were dried by Na_2SO_4, then filtered, and evaporated under reduced pressure. The crude product was purified by flash chromatography to afford the desired cyanide.

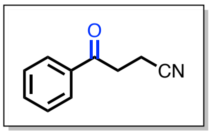

4-oxo-4-phenylbutanenitrile (71) [21]: Following the above procedure. The product was purified by column chromatography on silica gel (10:1, petroleum ether/ethyl acetate), white solid, 15.9 mg, 50% yield.

^{1}H NMR (400 MHz, $CDCl_3$) δ 8.09 – 7.88 (m, 2H), 7.70 – 7.57 (m, 1H), 7.50 (td, J = 7.6, 1.9 Hz, 2H), 3.39 (ddd, J = 9.4, 7.0, 2.6 Hz, 2H), 2.78 (ddd, J = 9.2, 5.4, 2.4 Hz, 2H).

^{13}C NMR (101 MHz, $CDCl_3$) δ 195.31, 135.62, 133.93, 128.90, 128.04, 34.29, 11.82.

Synthesis of 72

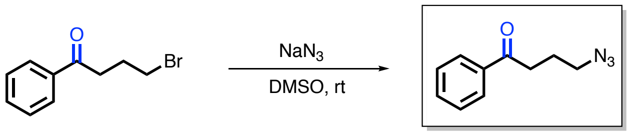

To a solution of **58** (0.2 mmol, 1.0 eq) in DMSO (1.0 mL), NaN_3 (0.2 mmol, 1.0 eq) was added to this solution and the reaction mixture was stirred for 18 hours at room temperature. After the reaction was complete, water (10.0 mL) and EtOAc (10.0 mL) were added. The organic layer was

separated, and the aqueous layer was extracted with EtOAc (30.0 mL) three times. The combined organic layers were dried over Na_2SO_4. After concentration, the residue was purified by flash column chromatography on silica gel to afford the desired cyanide.

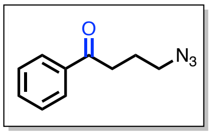

4-azido-1-phenylbutan-1-one (72)[19]: Following the above procedure. The product was purified by column chromatography on silica gel (10:1, petroleum ether/ethyl acetate), colorless oil, 30.2 mg, 80% yield.

^{1}H NMR (400 MHz, $CDCl_3$) δ 7.96 (dd, *J* = 7.3, 1.6 Hz, 2H), 7.71 – 7.53 (m, 1H), 7.47 (dd, *J* = 8.5, 6.9 Hz, 2H), 3.42 (t, *J* = 6.6 Hz, 2H), 3.08 (t, *J* = 7.0 Hz, 2H), 2.04 (p, *J* = 6.8 Hz, 2H).

^{13}C NMR (126 MHz, $CDCl_3$) δ 198.95, 136.73, 133.24, 128.67, 128.00, 50.88, 35.17, 23.34.

Synthesis of 67 and 68

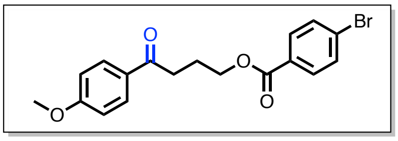

4-(4-methoxyphenyl)-4-oxobutyl 4-bromobenzoate (66): The product was purified by column chromatography on silica gel (10:1, petroleum ether/ethyl acetate), white solid, 47.5 mg, 85% yield.

^{1}H NMR (400 MHz, $CDCl_3$) δ 7.94 (d, *J* = 8.9 Hz, 2H), 7.87 (d, *J* = 8.6 Hz, 2H), 7.56 (d, *J* = 8.6 Hz, 2H), 6.92 (d, *J* = 8.9 Hz, 2H), 4.42 (t, *J* = 6.4 Hz, 2H), 3.86 (s, 3H), 3.09 (t, *J* = 7.2 Hz, 2H), 2.23 (p, *J* = 6.8 Hz, 2H).

^{13}C NMR (126 MHz, $CDCl_3$) δ 197.51, 165.82, 163.55, 131.69, 131.10, 130.28, 129.88, 129.15, 128.02, 113.77, 64.72, 55.48, 34.51, 23.45.

HRMS $[M+H]^+$ calcd for $[C_{18}H_{18}BrO_4]^+$: 377.0388; found: 377.0385.

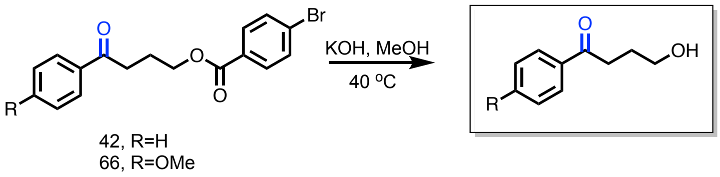

A mixture of **42** or **66** (0.2 mmol, 1.0 eq), KOH (0.24 mmol, 1.2 eq) and MeOH (2.0 mL) was heated to 40°C for 2 h. The solution was cooled to room temperature, and neutralized with conc. HCl (aq).

The acidified mixture was poured into water (10 mL) and extracted with EtOAc (15 mL *3). The combined organic layers were dried over Na_2SO_4, and the solvent evaporated under reduced pressure. The resulting residue was purified by flash silica gel column chromatography to yield the desired products acetosyringone.

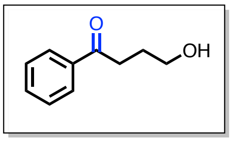

4-hydroxy-1-phenylbutan-1-one (67)[45]: Following the above procedure. The product was purified by column chromatography on silica gel (5:1-2:1, petroleum ether/ethyl acetate), colorless oil, 29.5 mg, 90% yield.

^{1}H NMR (400 MHz, $CDCl_3$) δ 7.98 (dd, *J* = 8.2, 1.4 Hz, 1H), 7.62 – 7.52 (m, 1H), 7.54 – 7.41 (m, 2H), 3.75 (t, *J* = 6.1 Hz, 1H), 3.14 (t, *J* = 6.9 Hz, 1H), 2.03 (p, *J* = 6.5 Hz, 1H).

^{13}C NMR (126 MHz, $CDCl_3$) δ 200.54, 136.86, 133.15, 128.62, 128.11, 62.35, 35.31, 26.91.

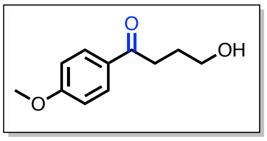

4-hydroxy-1-(4-methoxyphenyl)butan-1-one (68)[29]: Following the above procedure. The product was purified by column chromatography on silica gel (5:1-2:1, petroleum ether/ethyl acetate), colorless oil, 34.2 mg, 88% yield.

^{1}H NMR (400 MHz, $CDCl_3$) δ 7.97 (d, *J* = 8.9 Hz, 2H), 6.93 (d, *J* = 8.9 Hz, 2H), 3.87 (s, 3H), 3.74 (t, *J* = 6.1 Hz, 2H), 3.09 (t, *J* = 6.9 Hz, 2H), 2.10 – 1.91 (m, 3H).

^{13}C NMR (101 MHz, $CDCl_3$) δ 199.27, 163.55, 130.42, 129.93, 113.75, 62.44, 55.49, 35.04, 27.09.

13. NMR Spectra

^{1}H NMR spectrum of **2** (400 MHz, $CDCl_3$)

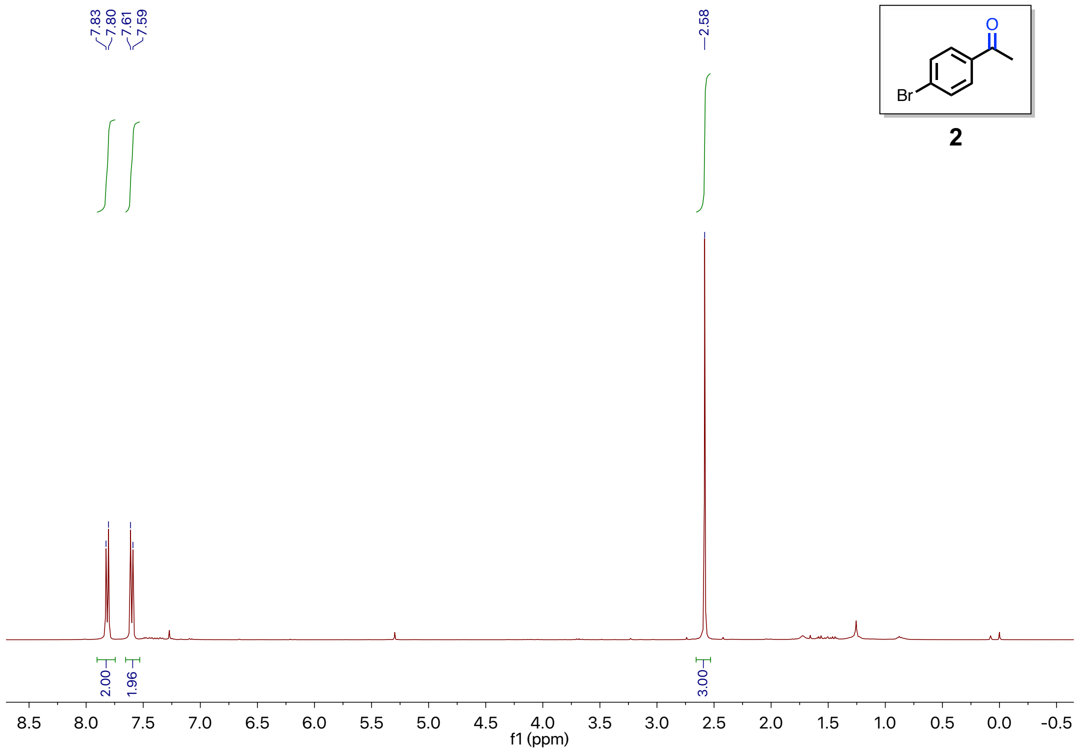

^{13}C NMR spectrum of **2** (101 MHz, $CDCl_3$)

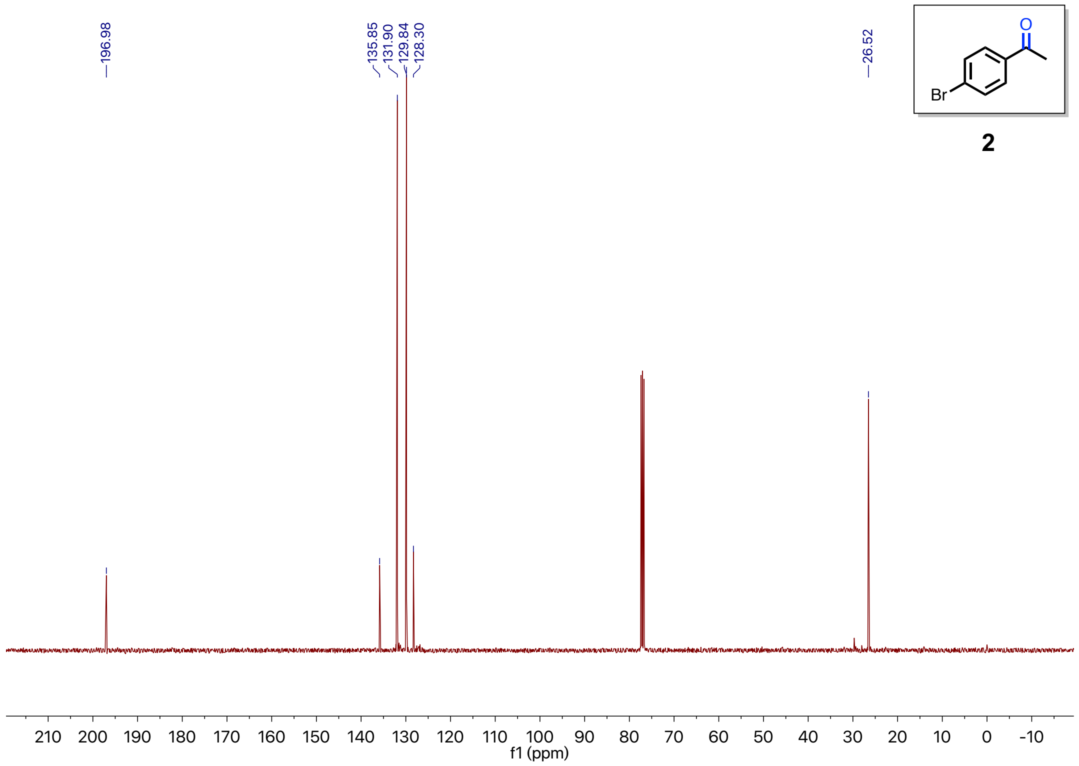

^{1}H NMR spectrum of **3** (400 MHz, $CDCl_3$)

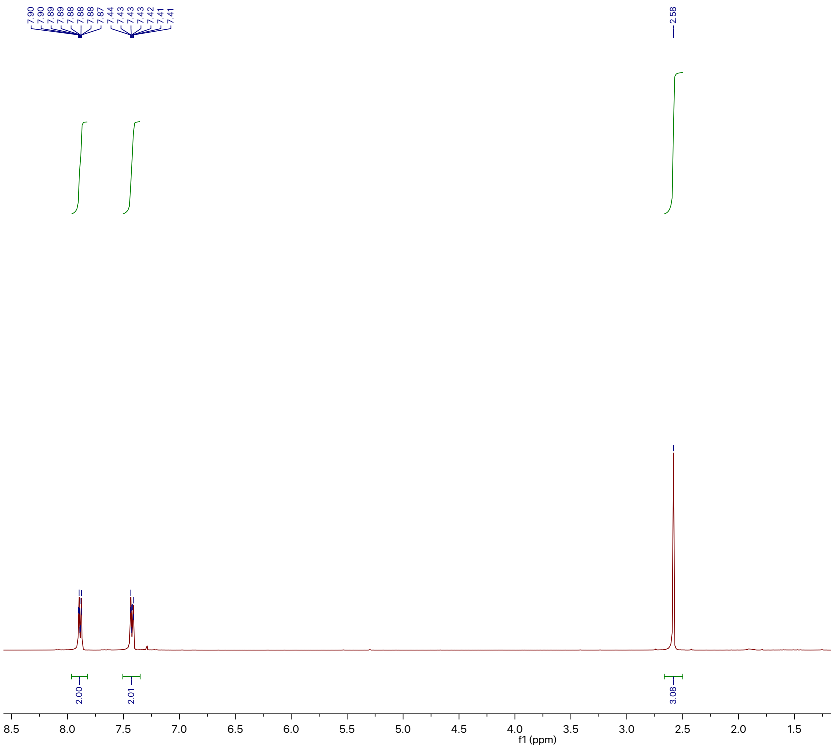

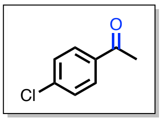

3

^{13}C NMR spectrum of **3** (126 MHz, $CDCl_3$)

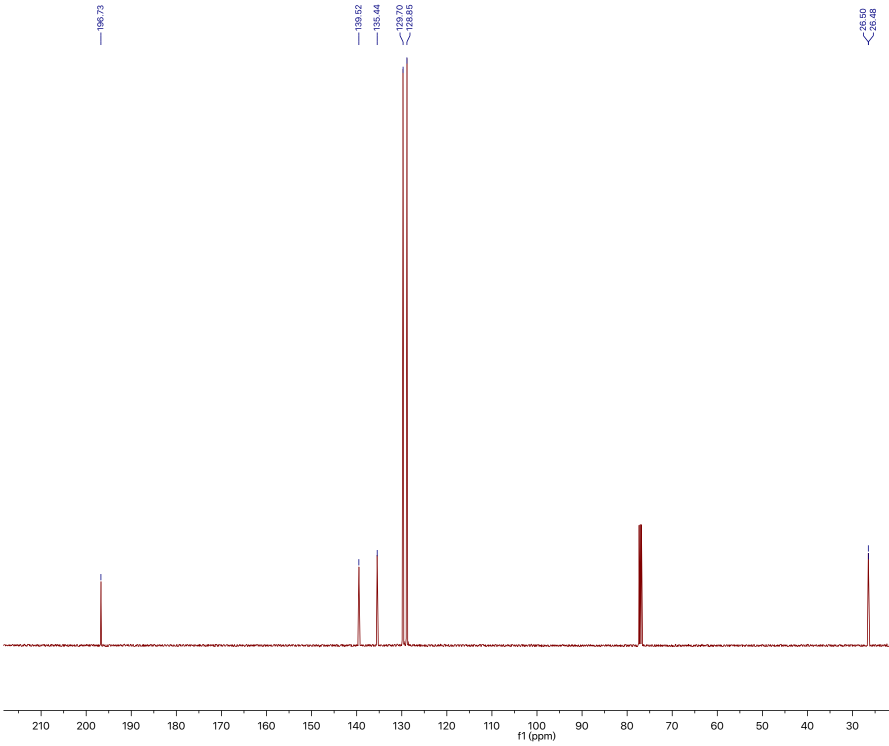

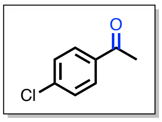

3

^{1}H NMR spectrum of **4** (400 MHz, $CDCl_3$)

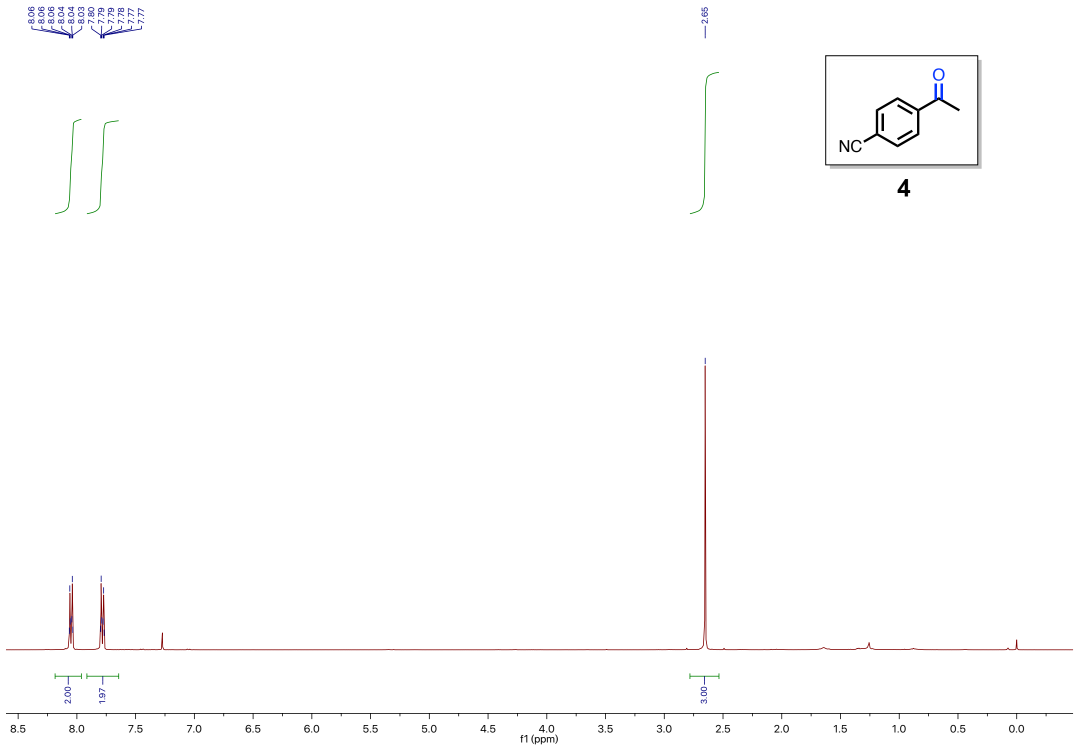

^{13}C NMR spectrum of **4** (101 MHz, $CDCl_3$)

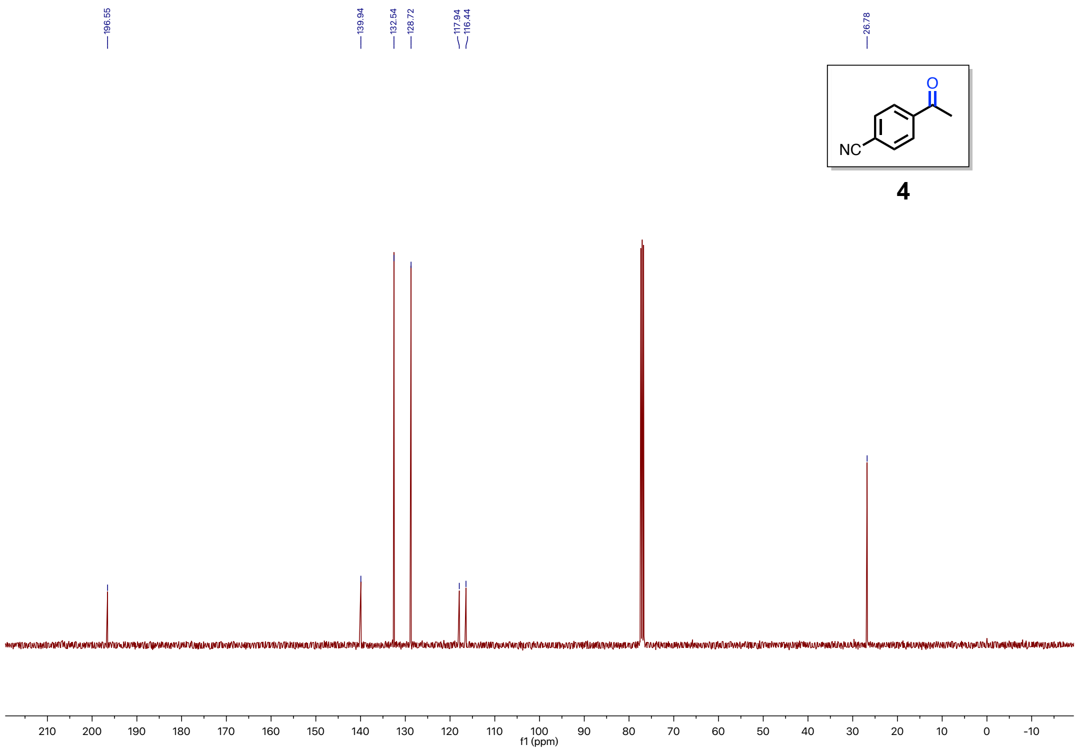

^{1}H NMR spectrum of **5** (400 MHz, DMSO-d$_6$)

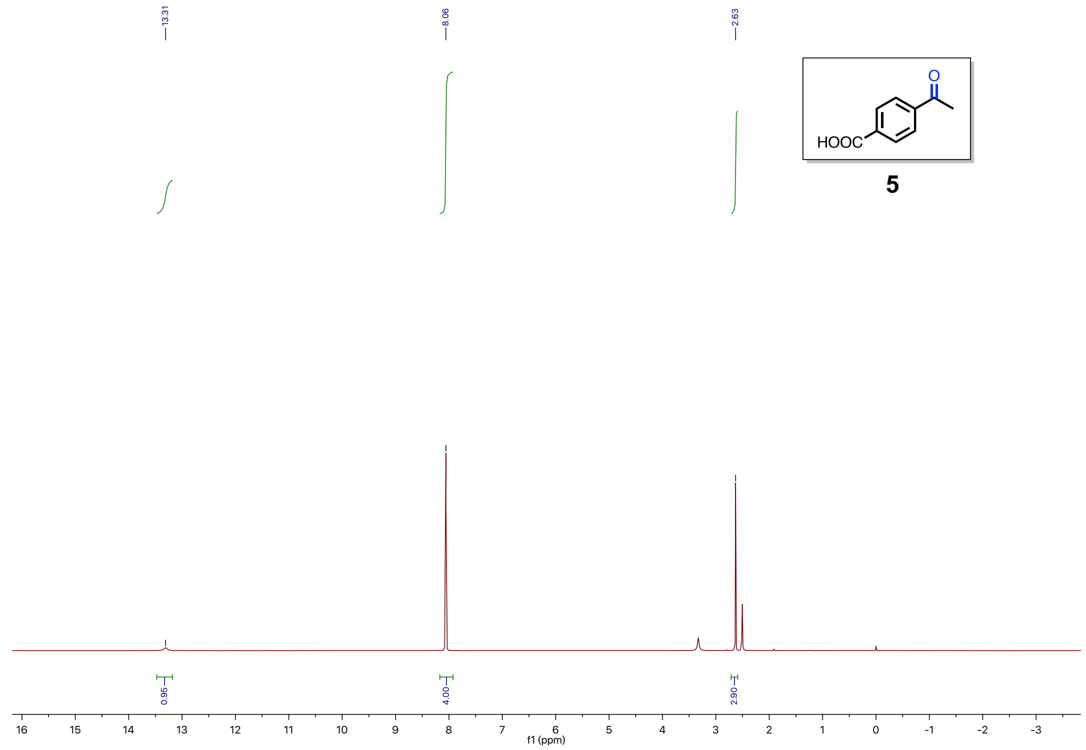

^{13}C NMR spectrum of **5** (101 MHz, DMSO-d$_6$)

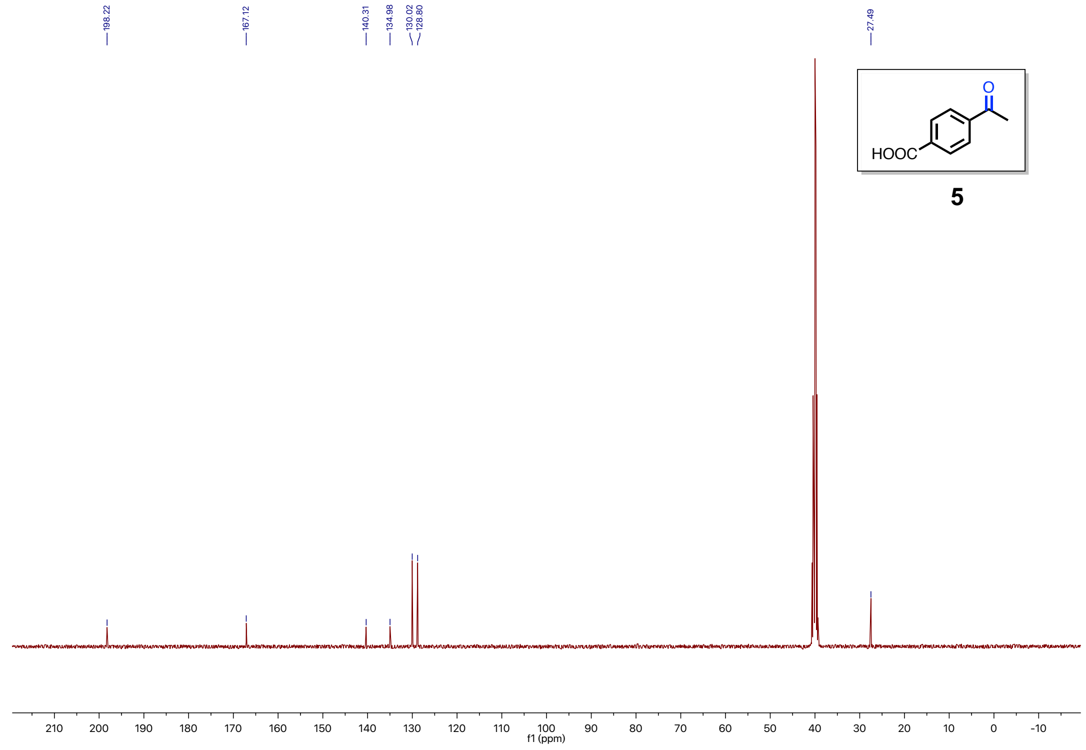

^{1}H NMR spectrum of **6** (400 MHz, $CDCl_3$)

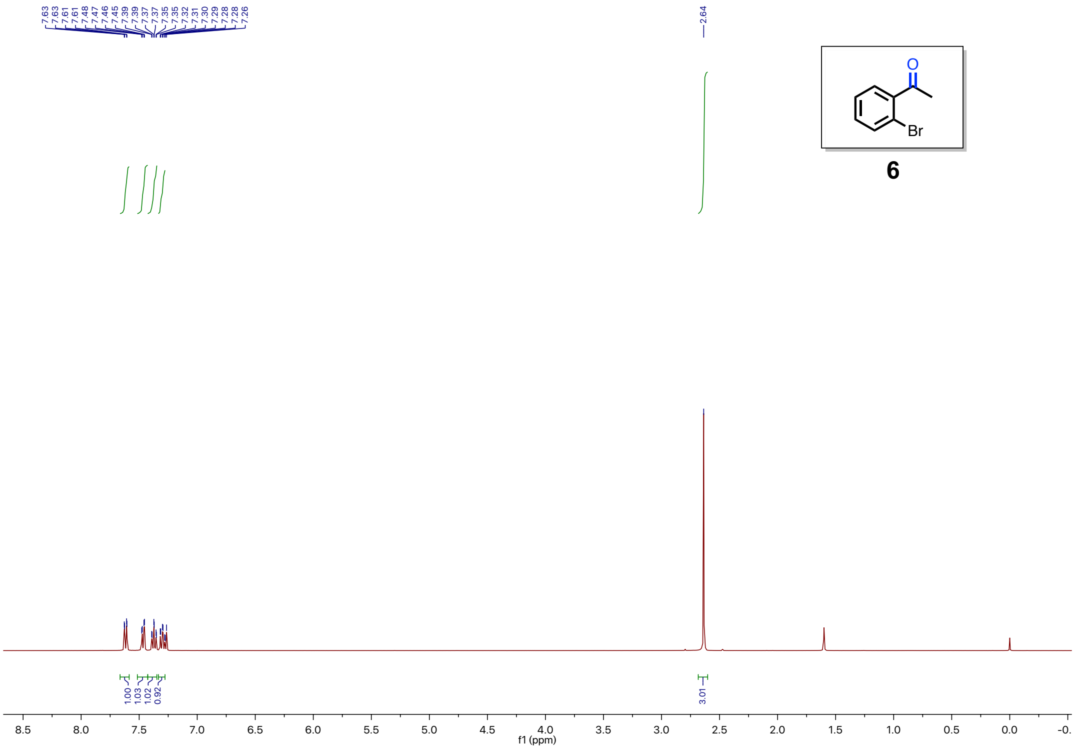

^{13}C NMR spectrum of **6** (101 MHz, $CDCl_3$)

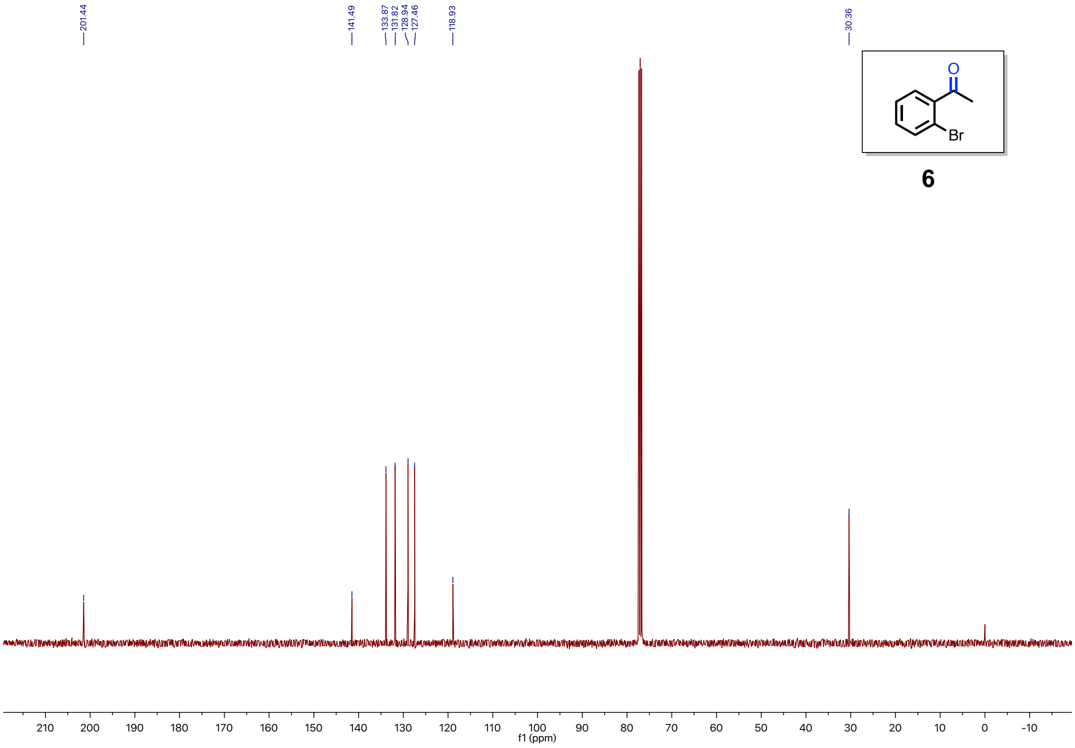

^{1}H NMR spectrum of **7** (400 MHz, $CDCl_3$)

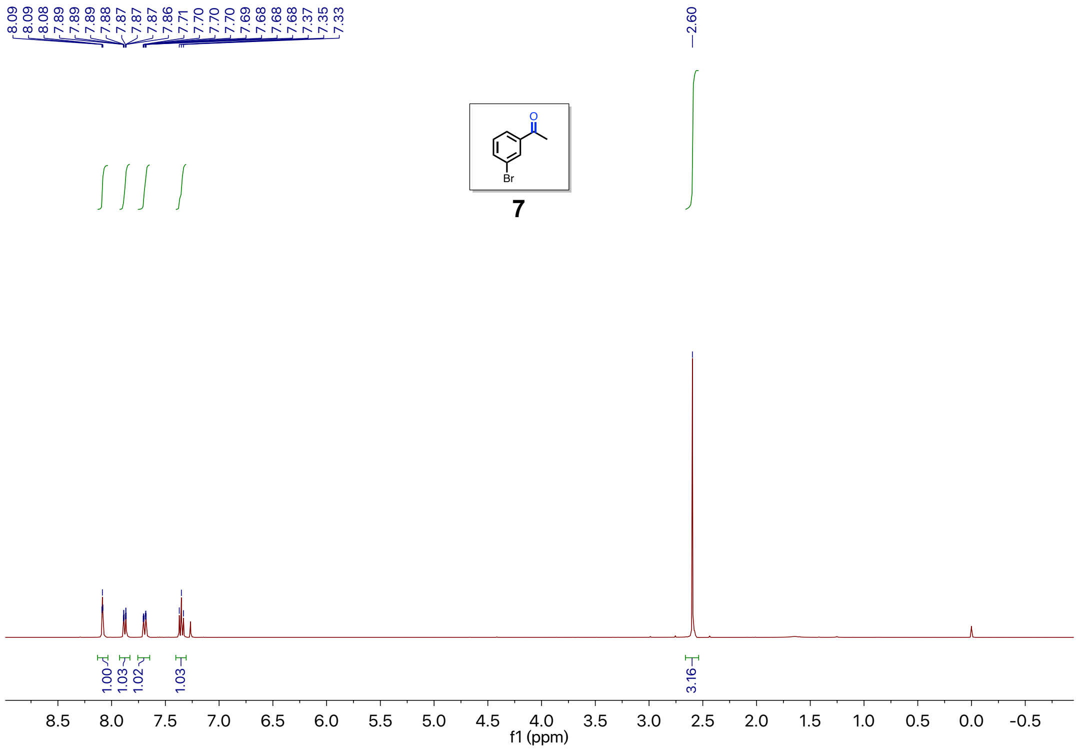

^{13}C NMR spectrum of **7** (101 MHz, $CDCl_3$)

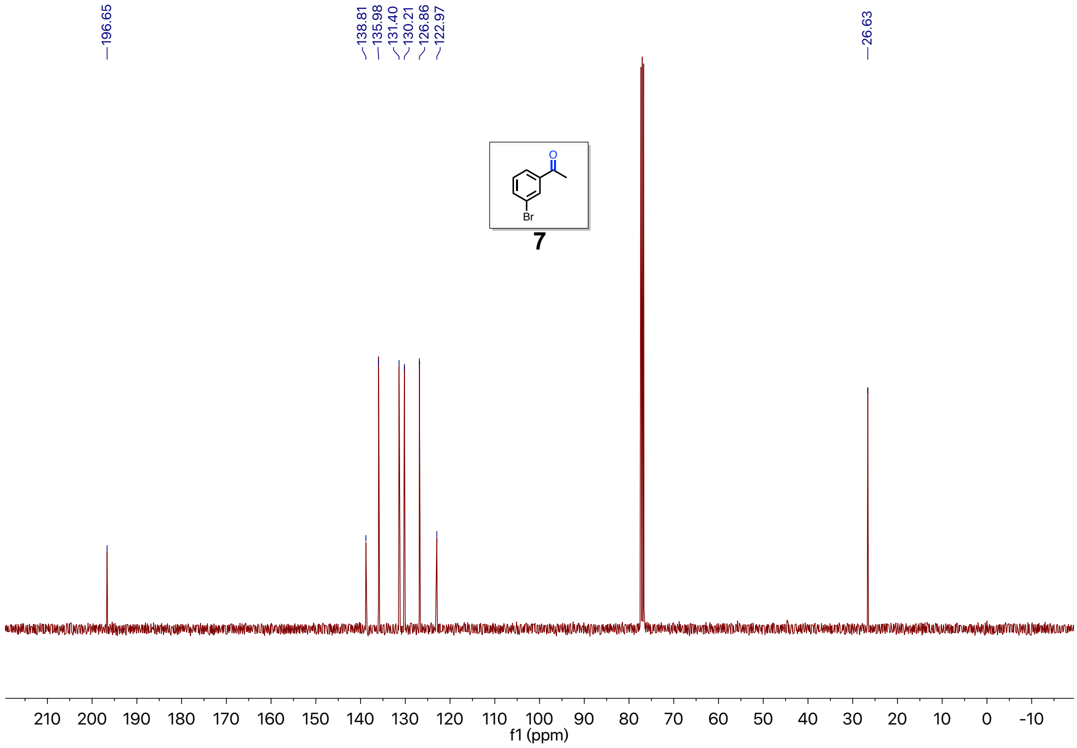

^{1}H NMR spectrum of **8** (400 MHz, $CDCl_3$)

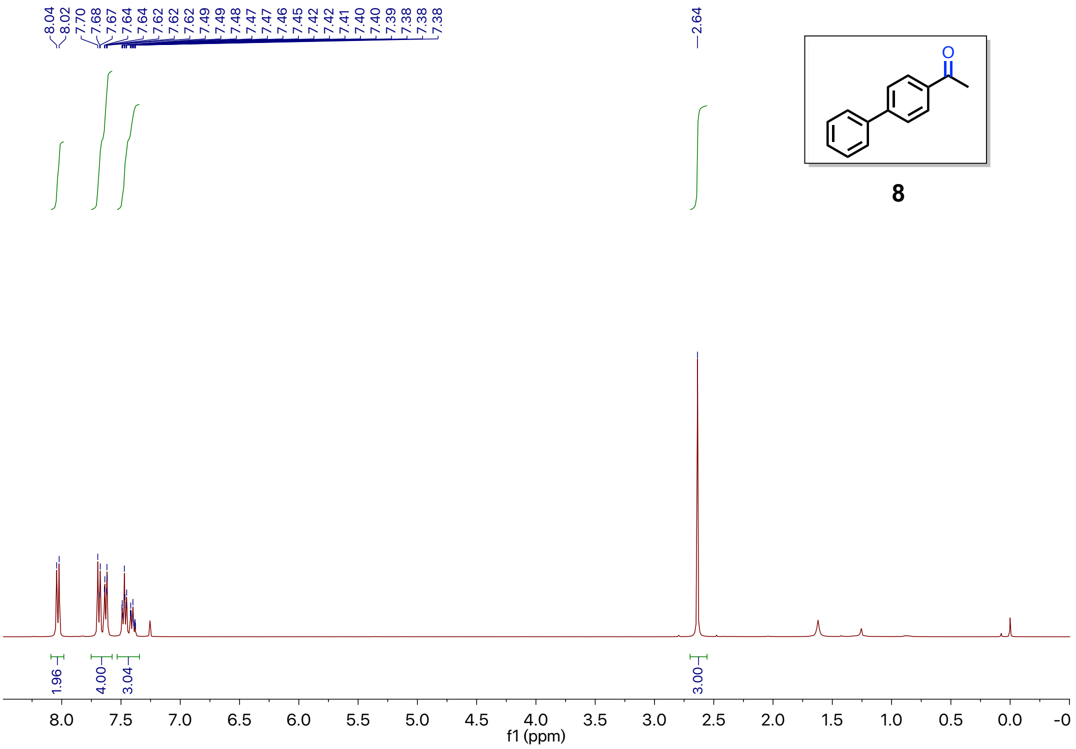

^{13}C NMR spectrum of **8** (101 MHz, $CDCl_3$)

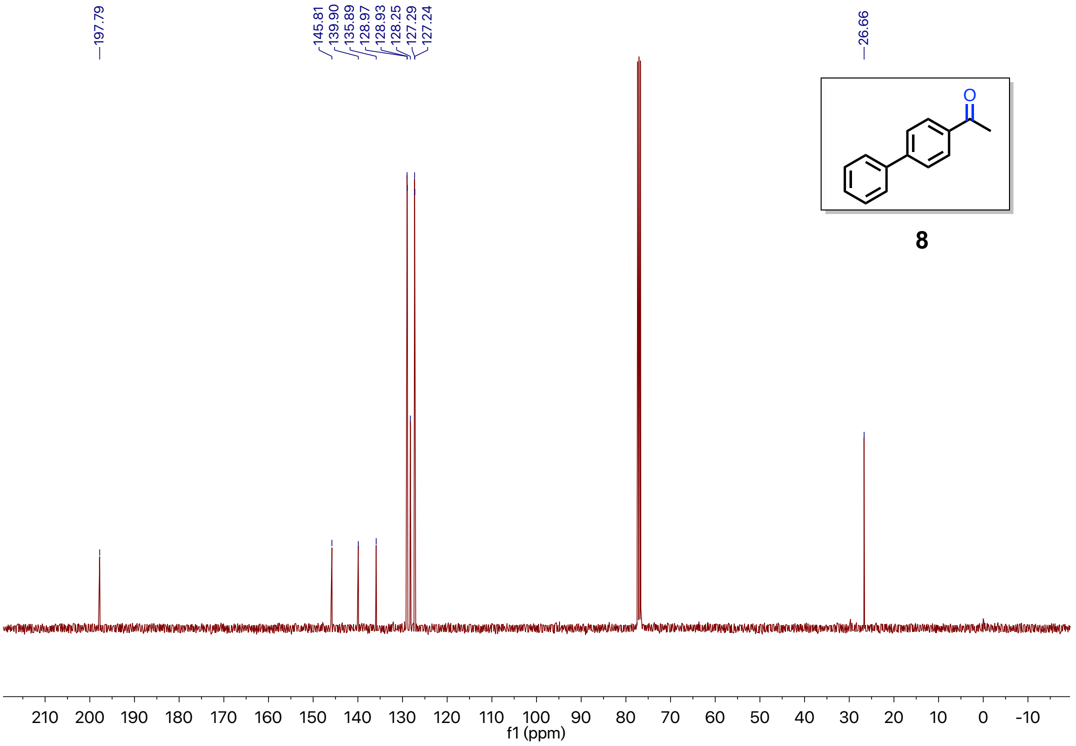

^{1}H NMR spectrum of **9** (400 MHz, $CDCl_3$)

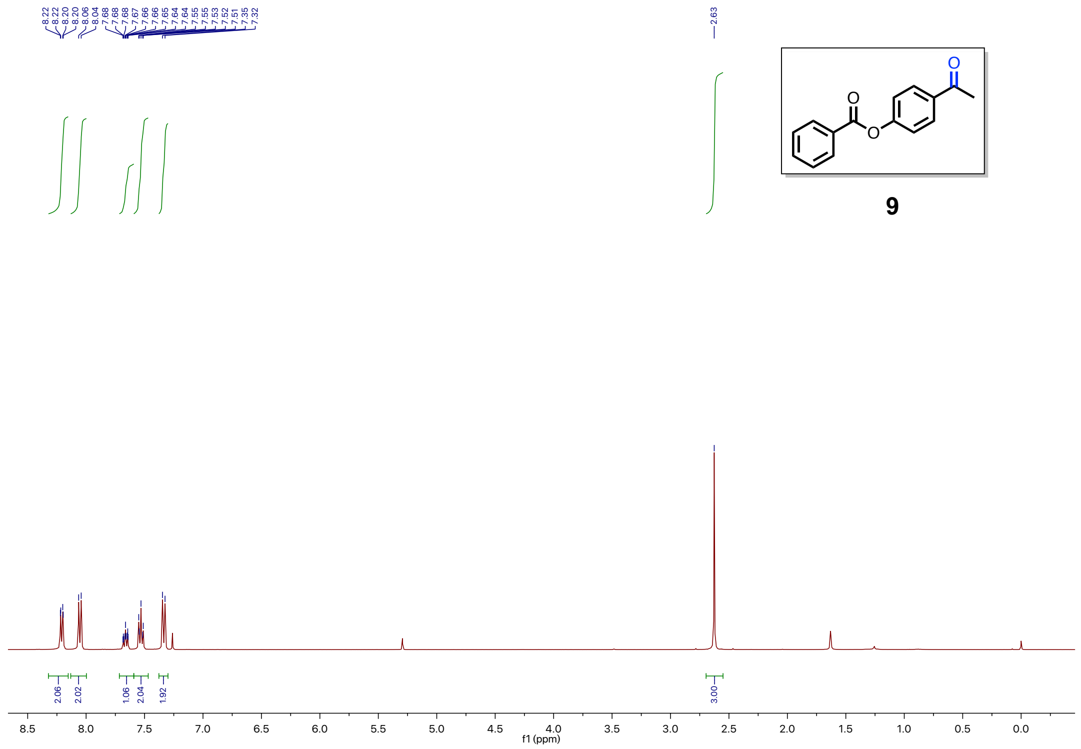

^{13}C NMR spectrum of **9** (101 MHz, $CDCl_3$)

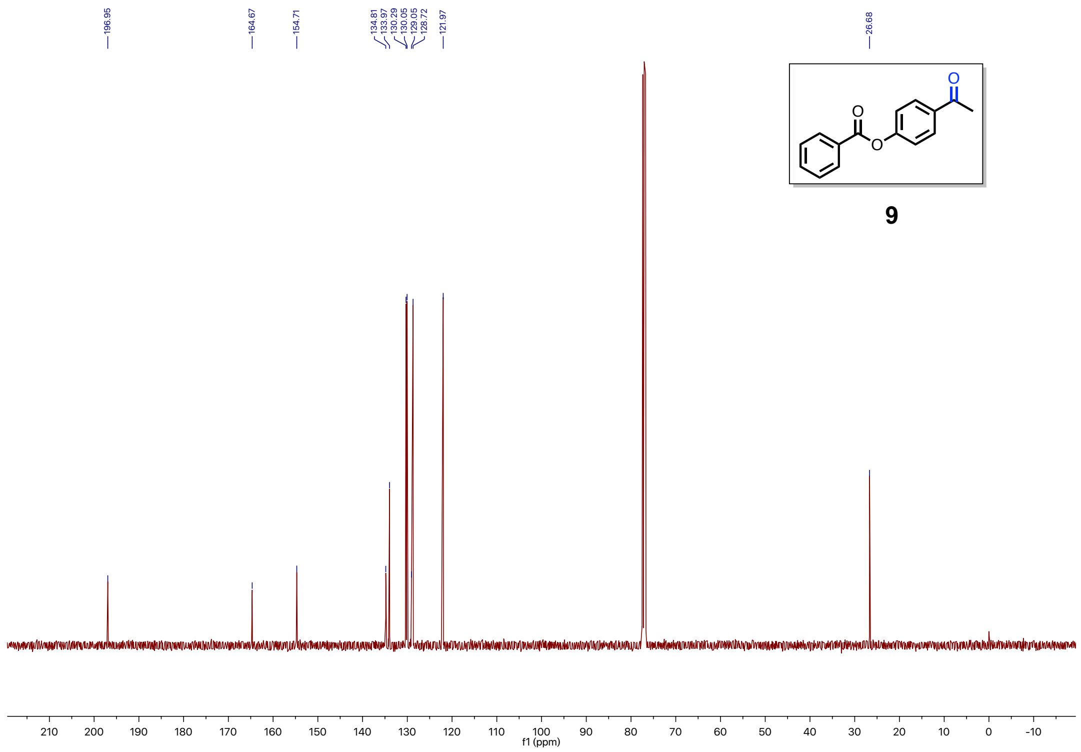

^{1}H NMR spectrum of **10** (400 MHz, $CDCl_3$)

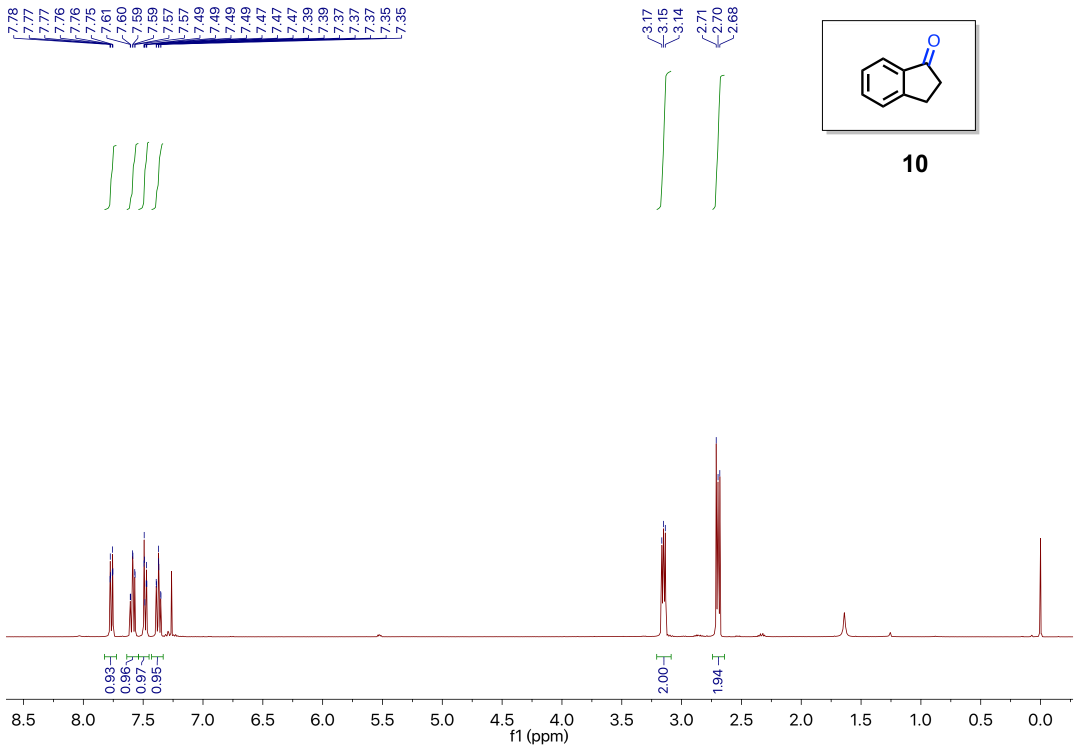

^{13}C NMR spectrum of **10** (101 MHz, $CDCl_3$)

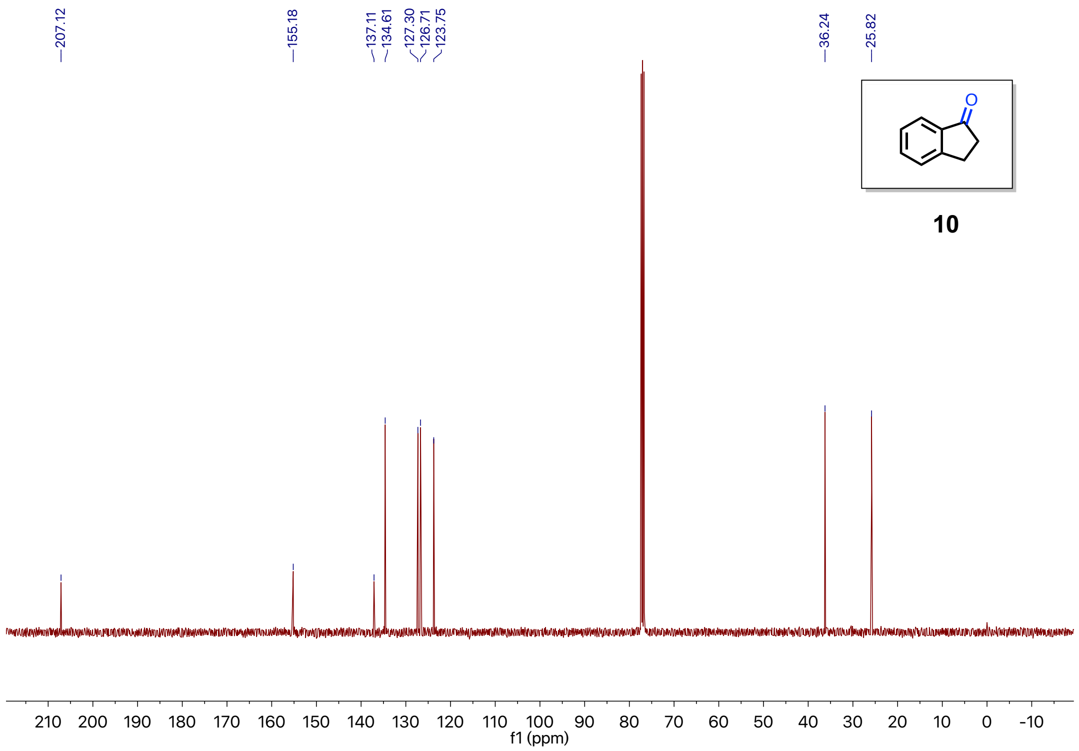

^{1}H NMR spectrum of **11** (400 MHz, $CDCl_3$)

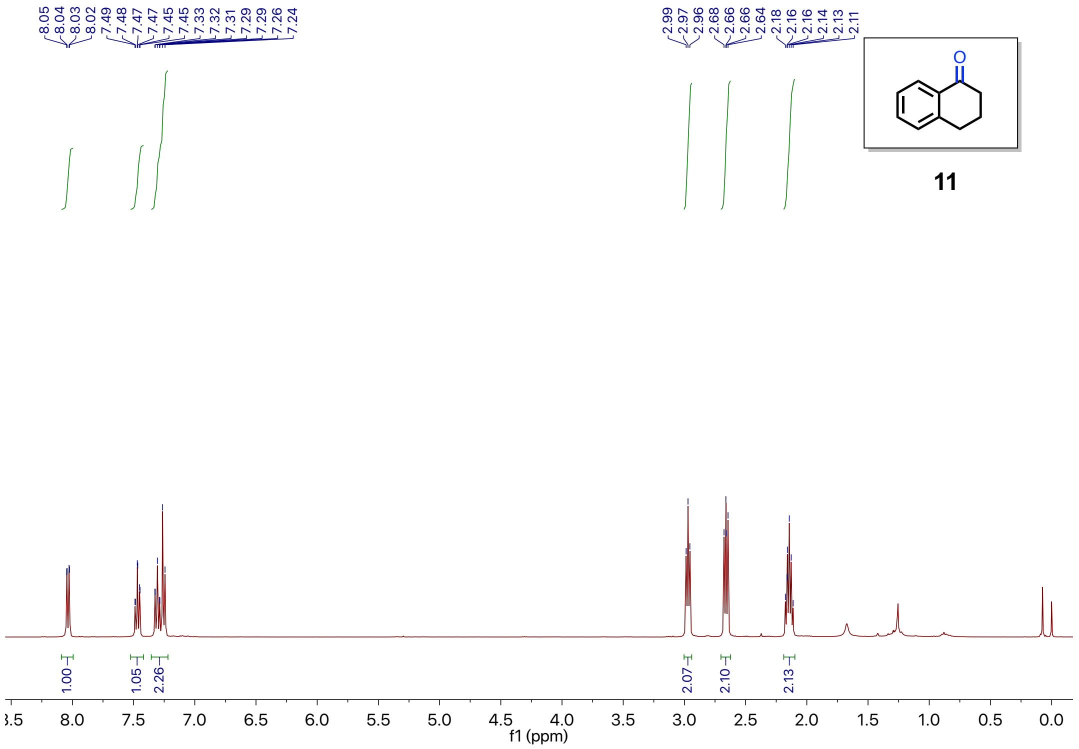

^{13}C NMR spectrum of **11** (101 MHz, $CDCl_3$)

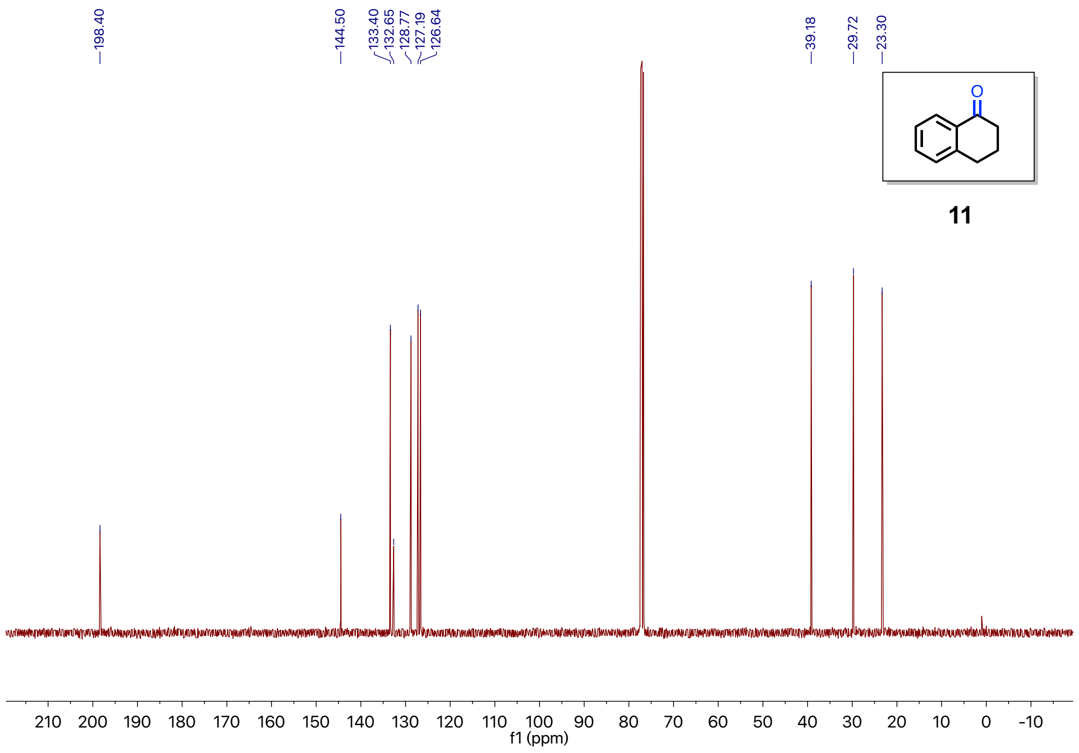

^{1}H NMR spectrum of **12** (400 MHz, $CDCl_3$)

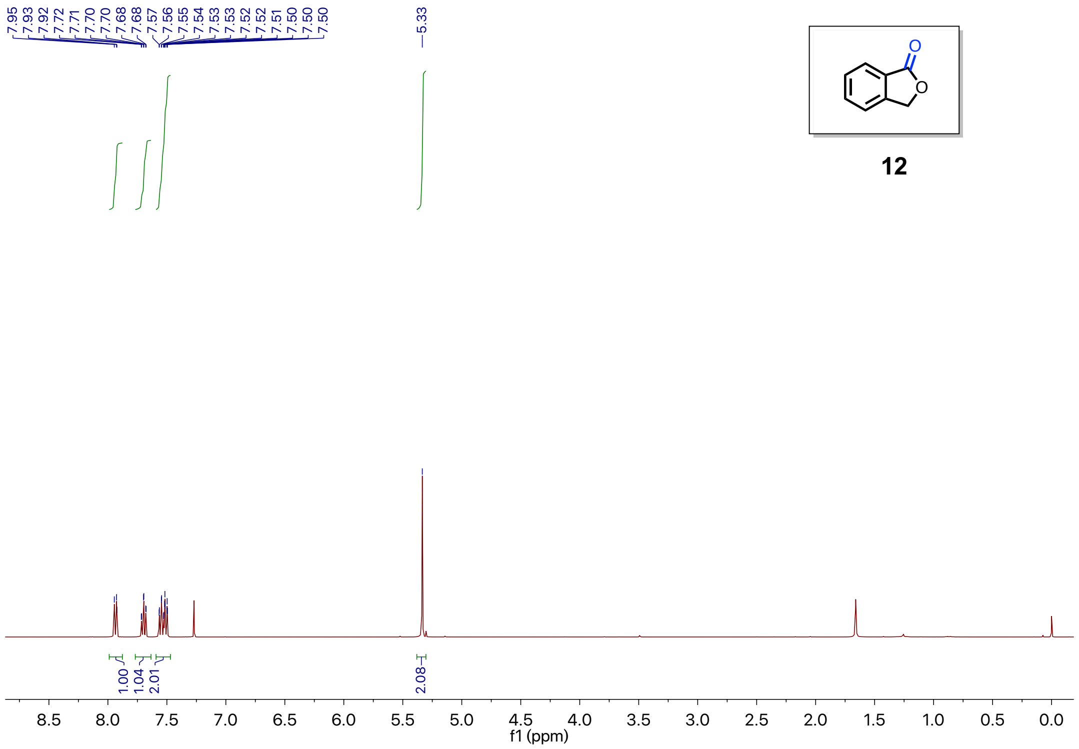

^{13}C NMR spectrum of **12** (101 MHz, $CDCl_3$)

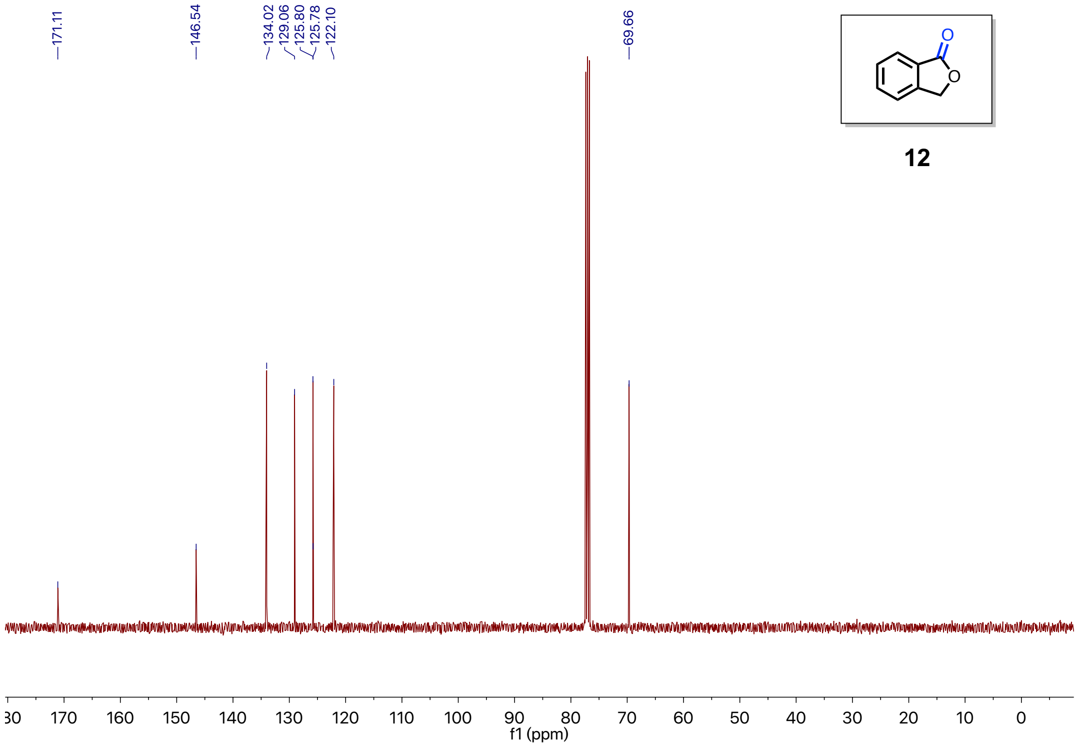

^{1}H NMR spectrum of **13** (400 MHz, $CDCl_3$)

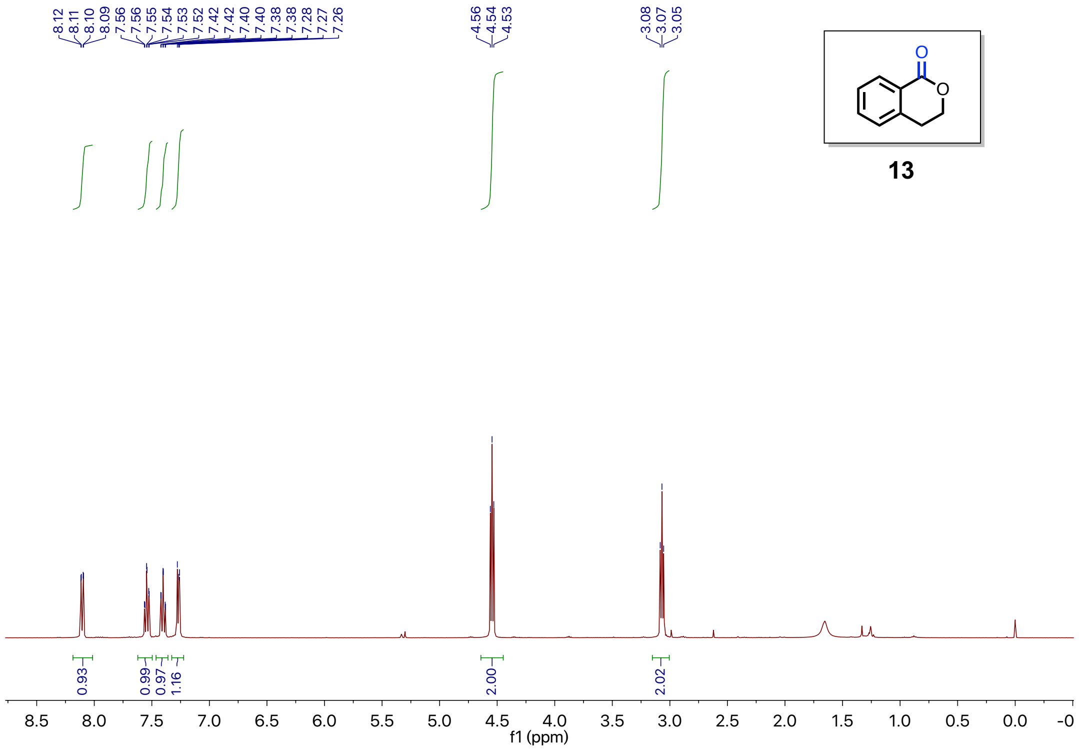

13

^{13}C NMR spectrum of **13** (101 MHz, $CDCl_3$)

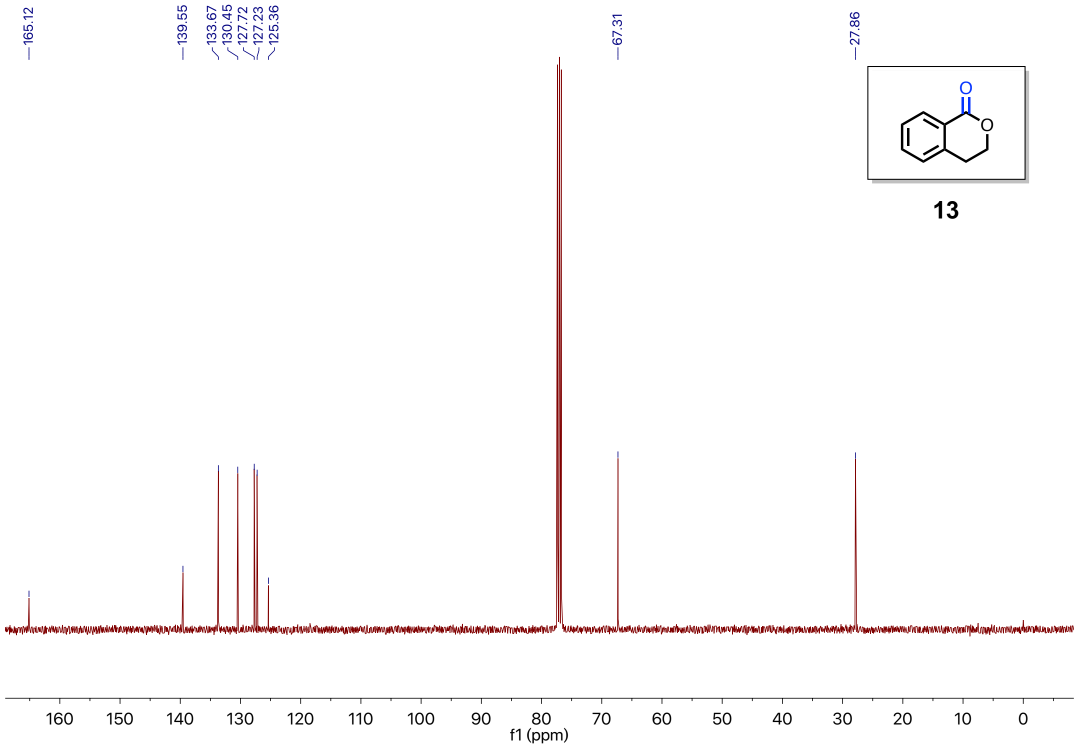

13

^{1}H NMR spectrum of **14** (400 MHz, $CDCl_3$)

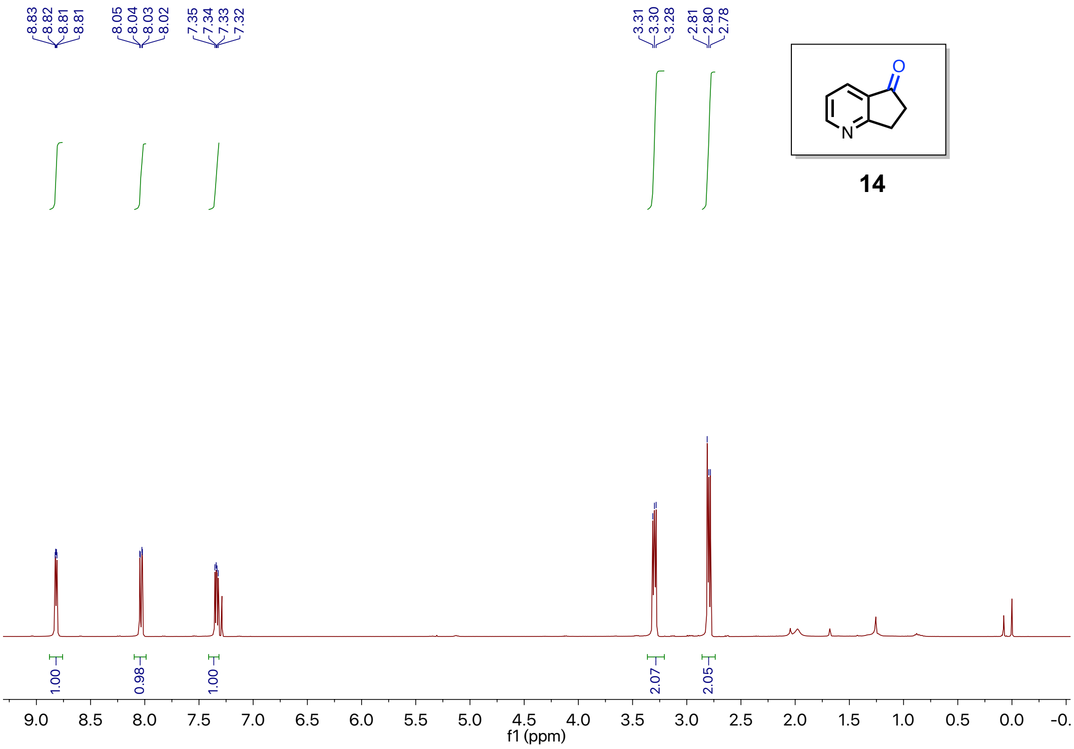

^{13}C NMR spectrum of **14** (101 MHz, $CDCl_3$)

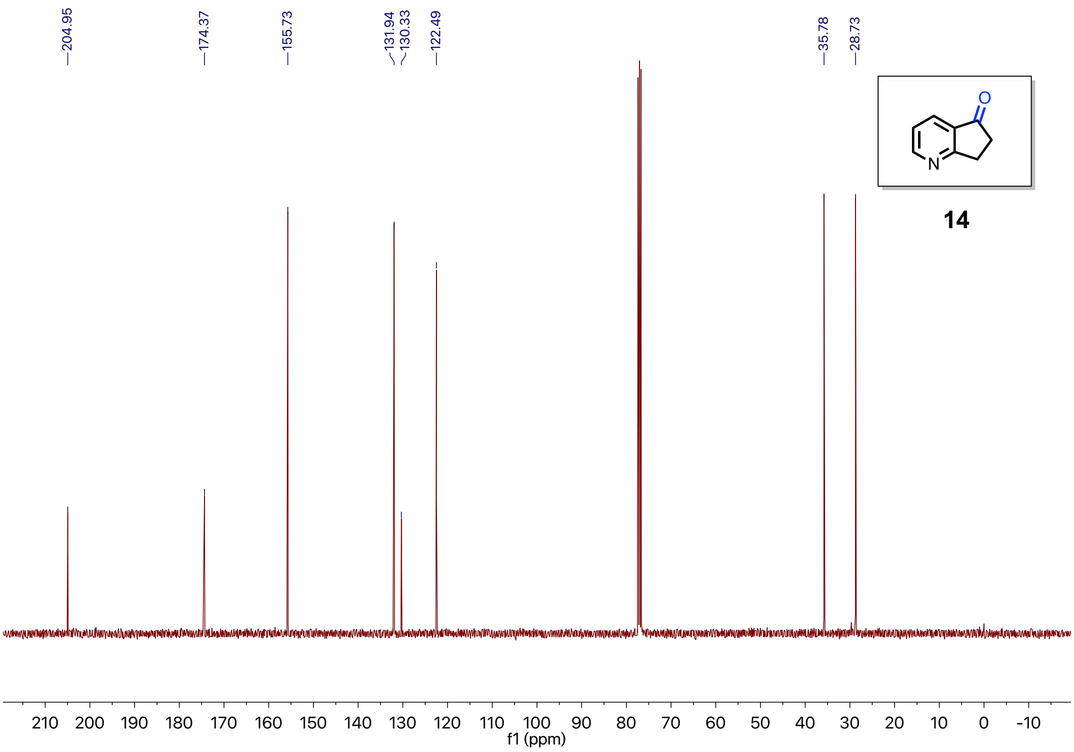

^{1}H NMR spectrum of **15** (400 MHz, $CDCl_3$)

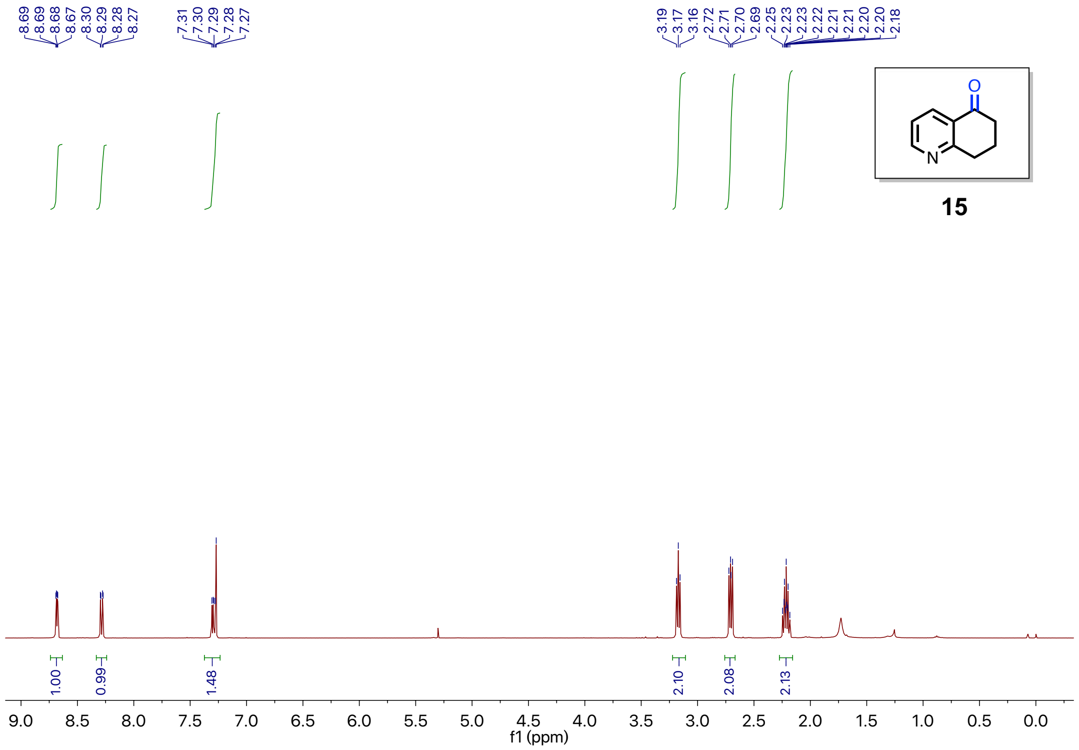

^{13}C NMR spectrum of **15** (101 MHz, $CDCl_3$)

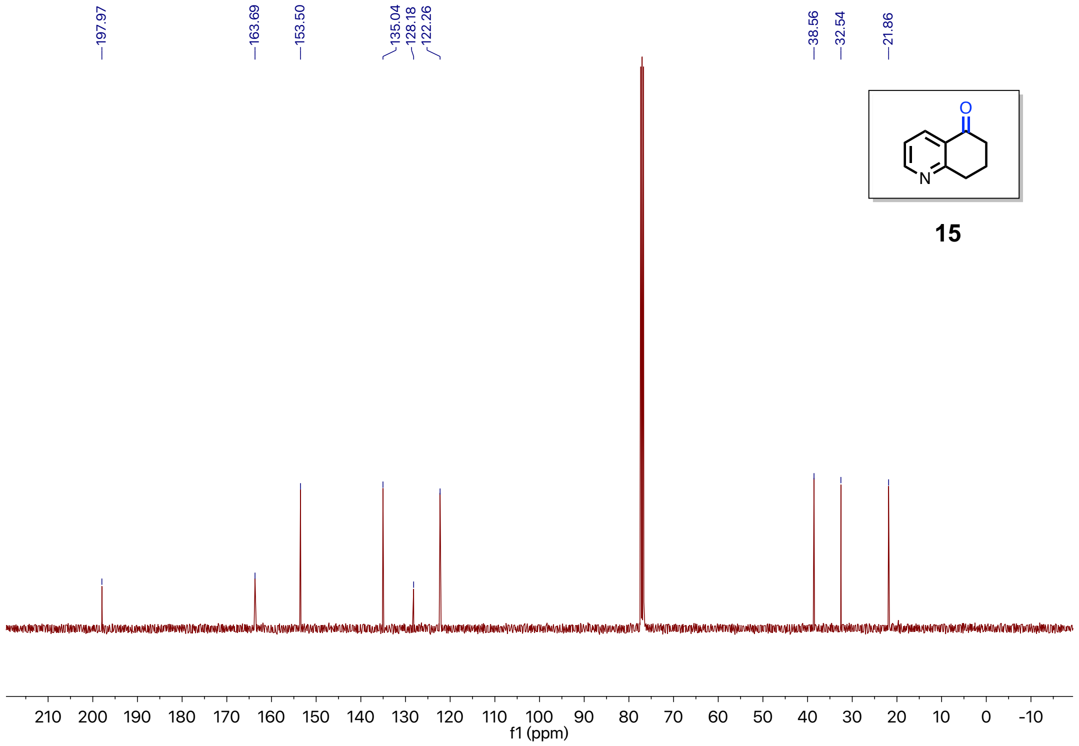

^{1}H NMR spectrum of **16** (400 MHz, $CDCl_3$)

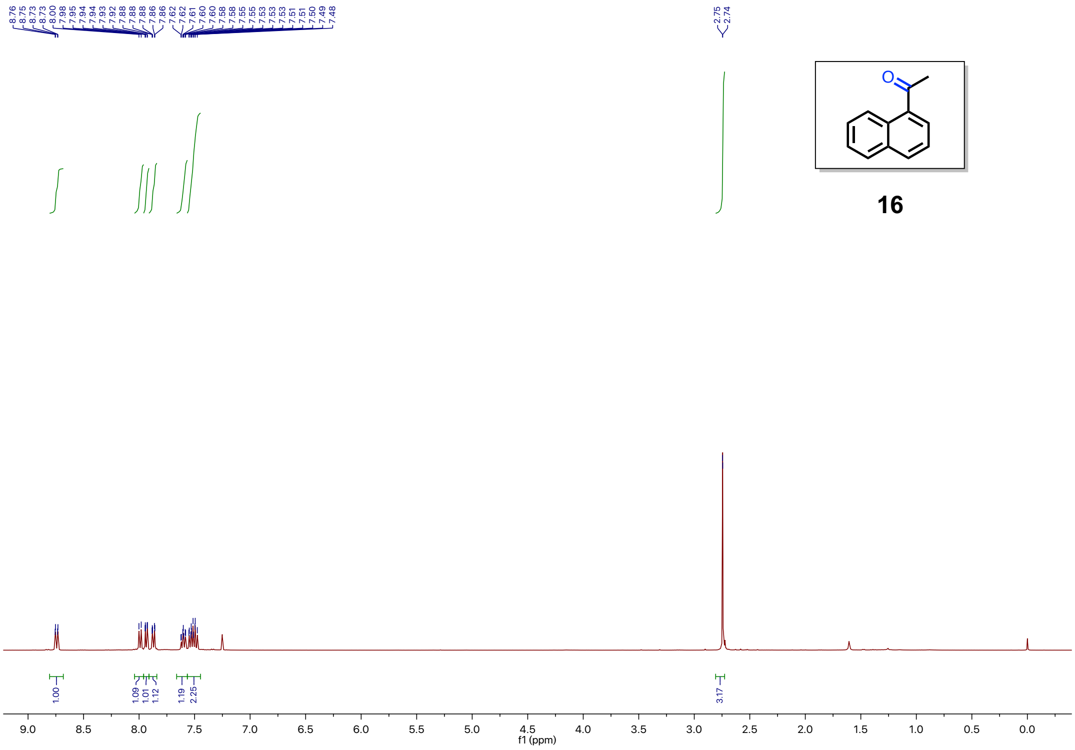

^{13}C NMR spectrum of **16** (101 MHz, $CDCl_3$)

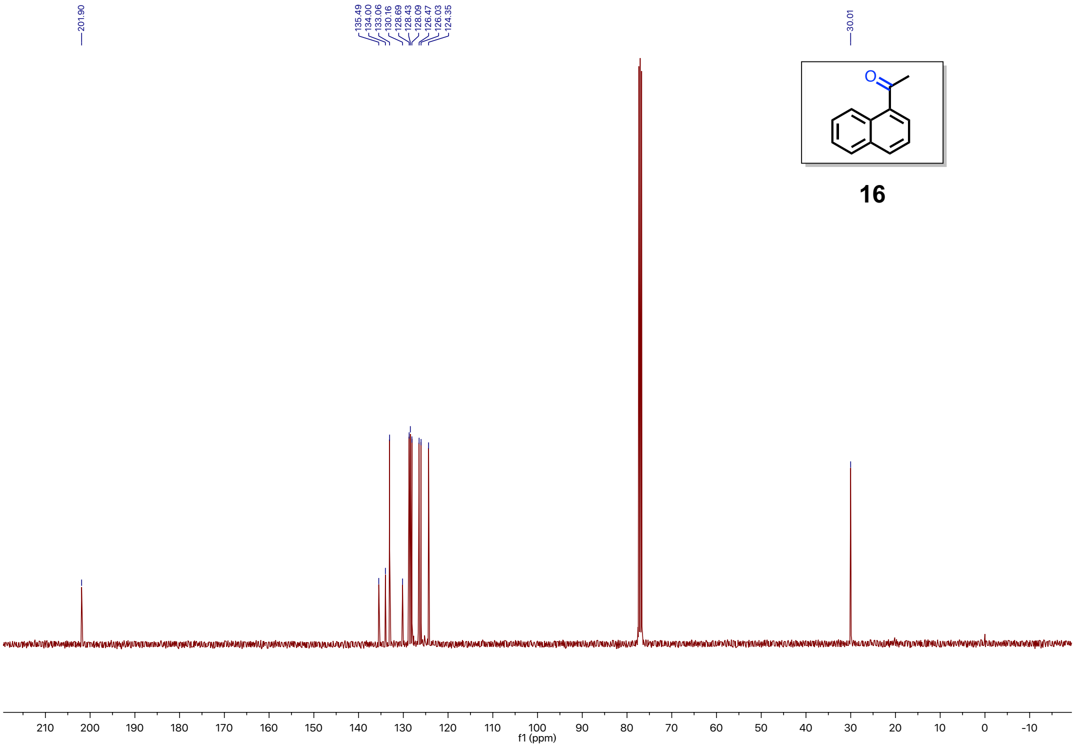

^{1}H NMR spectrum of **17** (400 MHz, $CDCl_3$)

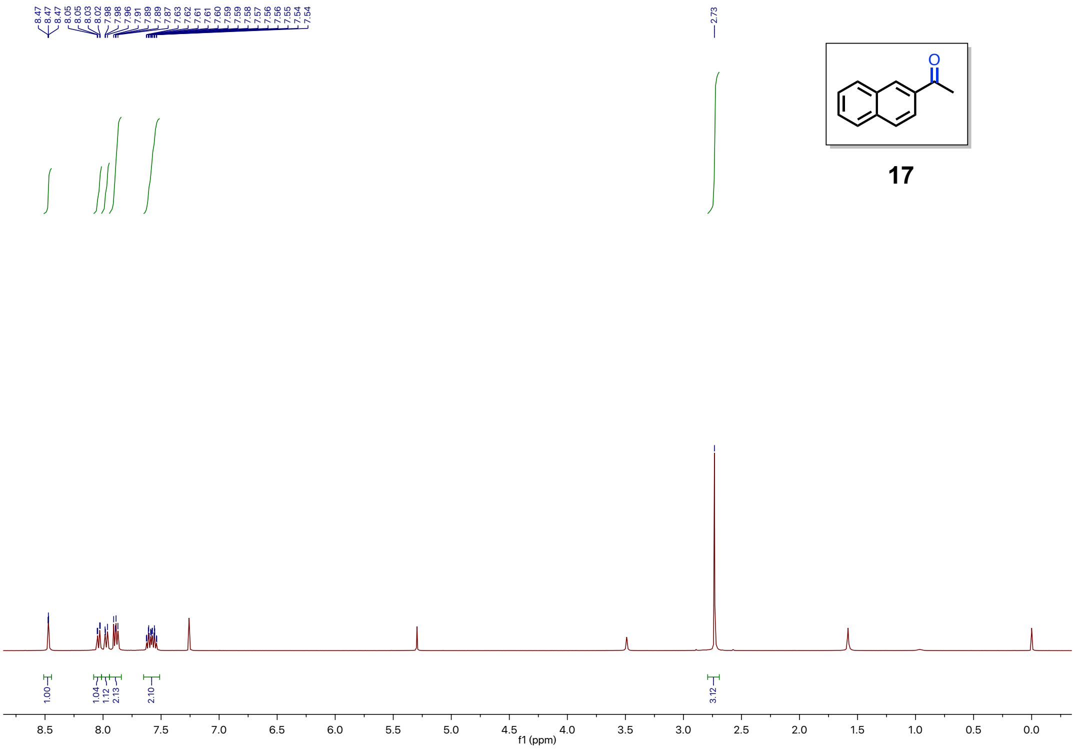

^{13}C NMR spectrum of **17** (101 MHz, $CDCl_3$)

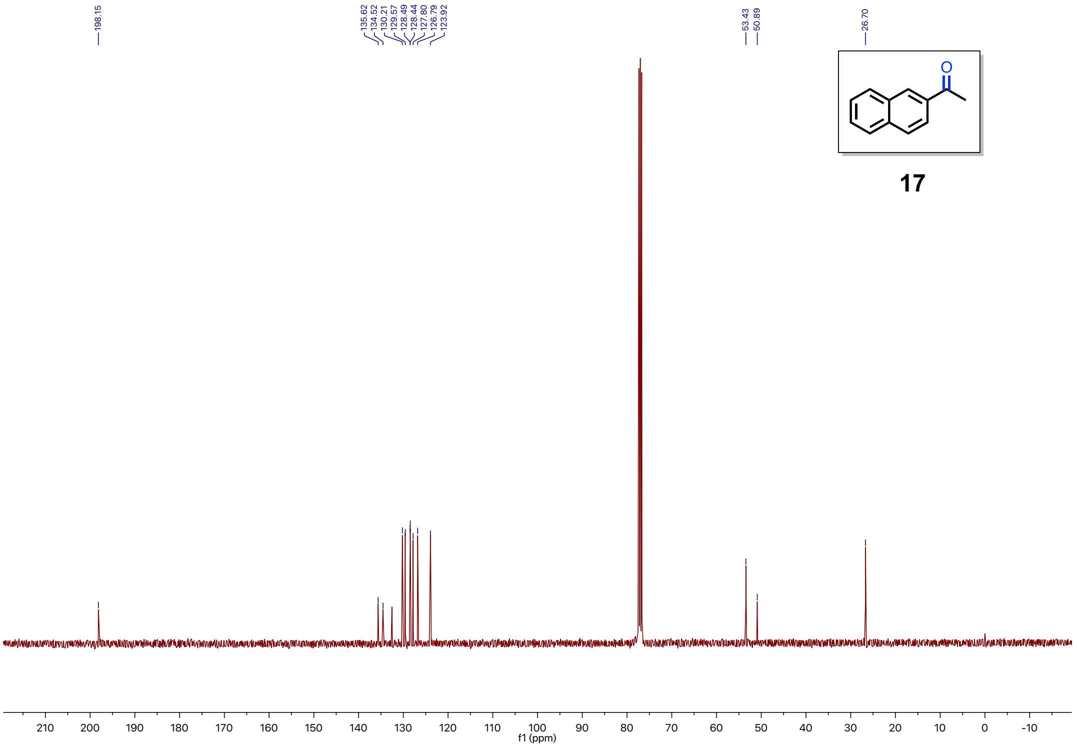

^{1}H NMR spectrum of **18** (400 MHz, $CDCl_3$)

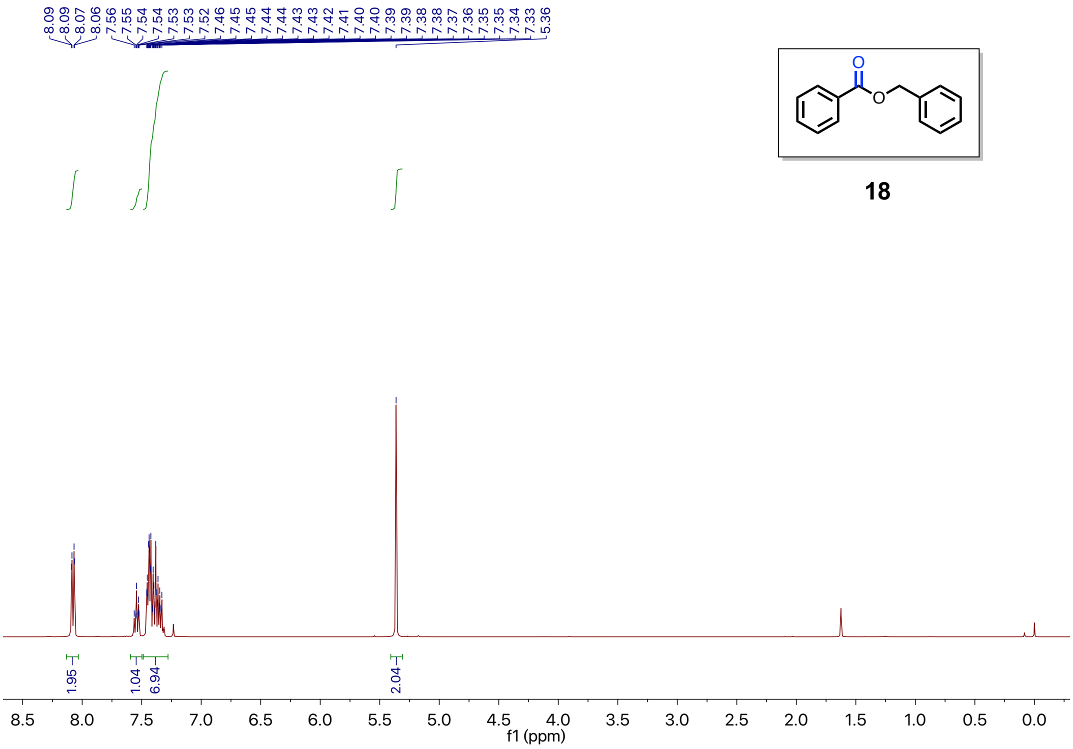

^{13}C NMR spectrum of **18** (101 MHz, $CDCl_3$)

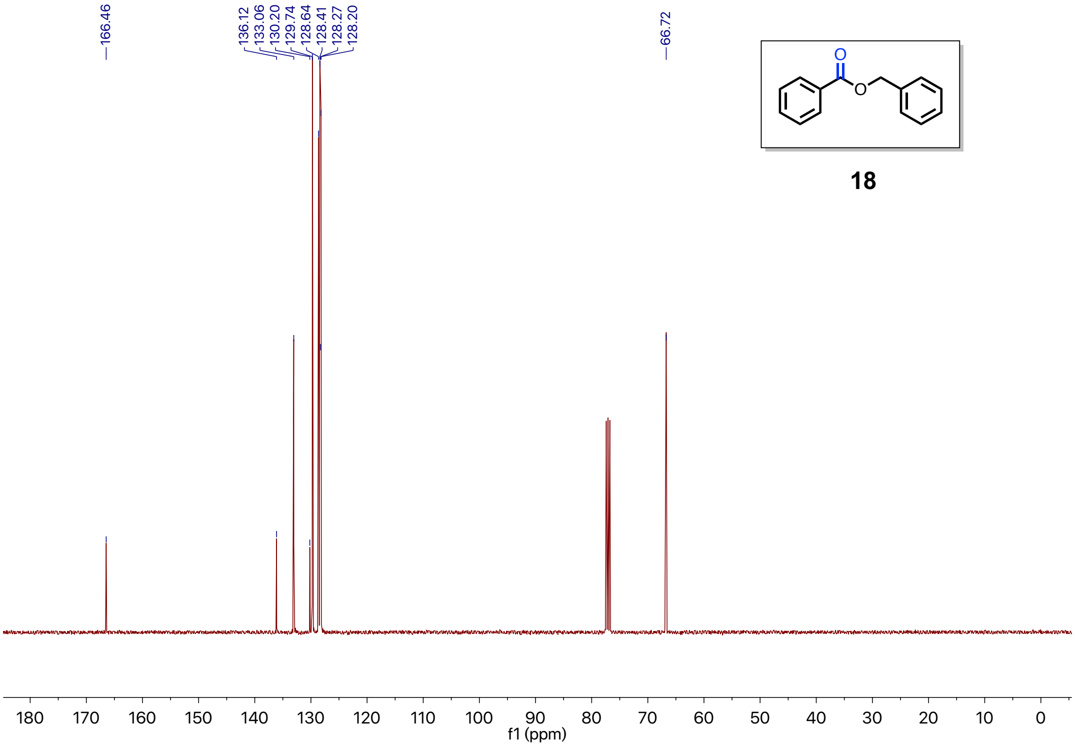

^{1}H NMR spectrum of **19** (400 MHz, $CDCl_3$)

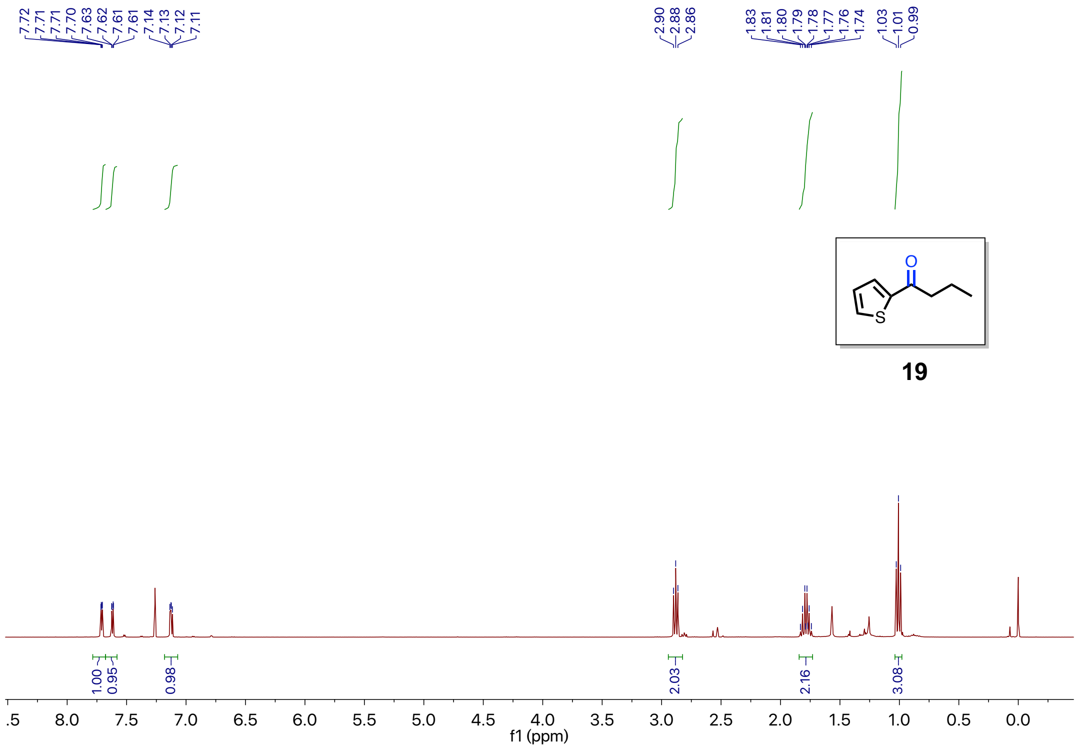

^{13}C NMR spectrum of **19** (101 MHz, $CDCl_3$)

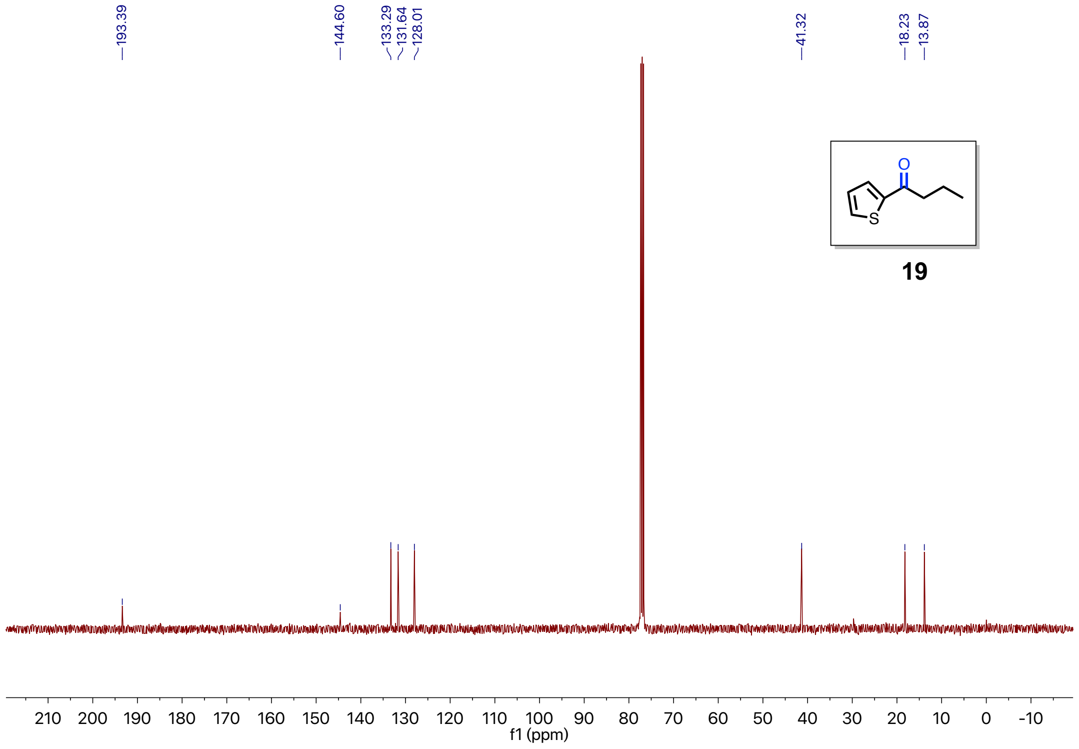

^{1}H NMR spectrum of **20** (400 MHz, $CDCl_3$)

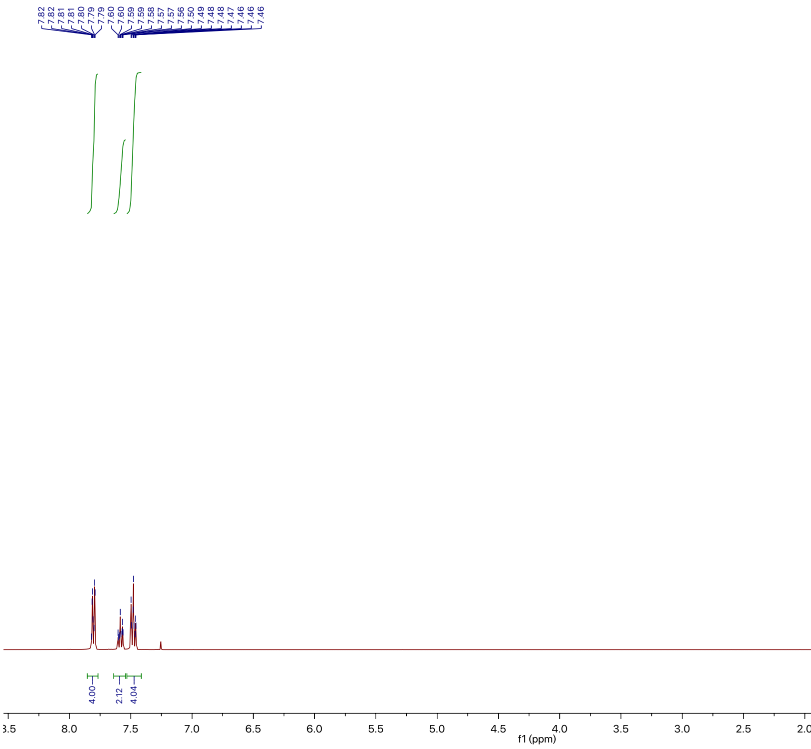

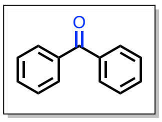

20

^{13}C NMR spectrum of **20** (101 MHz, $CDCl_3$)

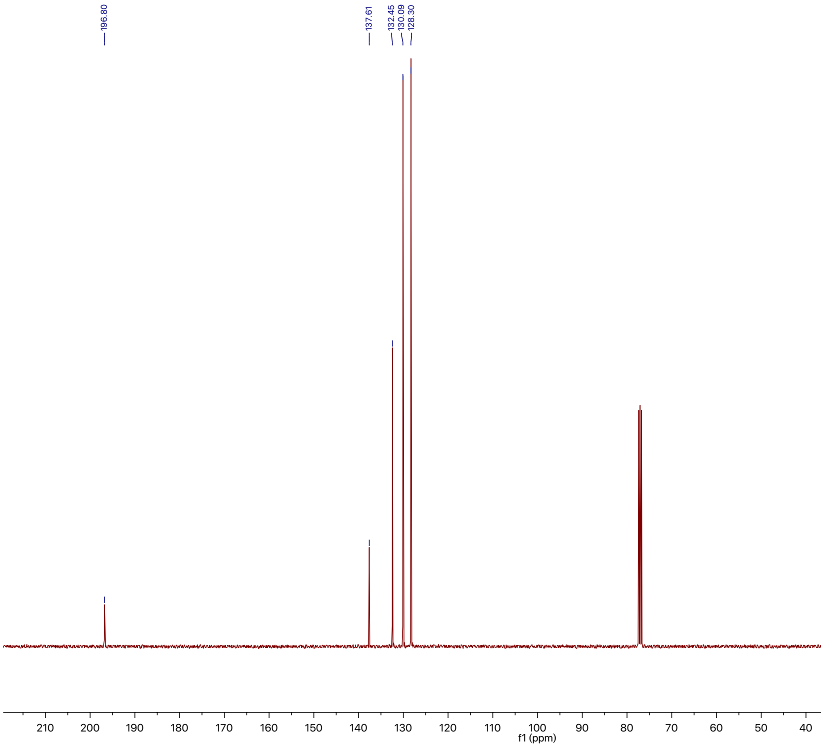

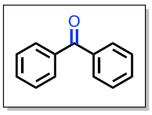

20

^{1}H NMR spectrum of **21** (400 MHz, $CDCl_3$)

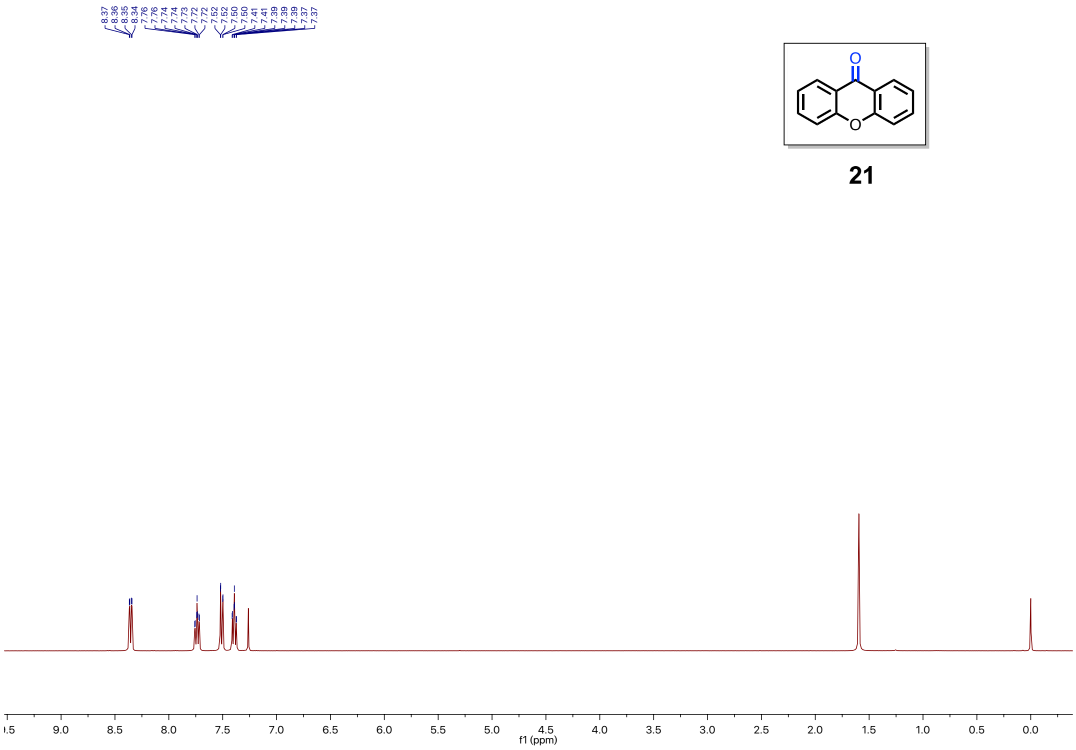

^{13}C NMR spectrum of **21** (101 MHz, $CDCl_3$)

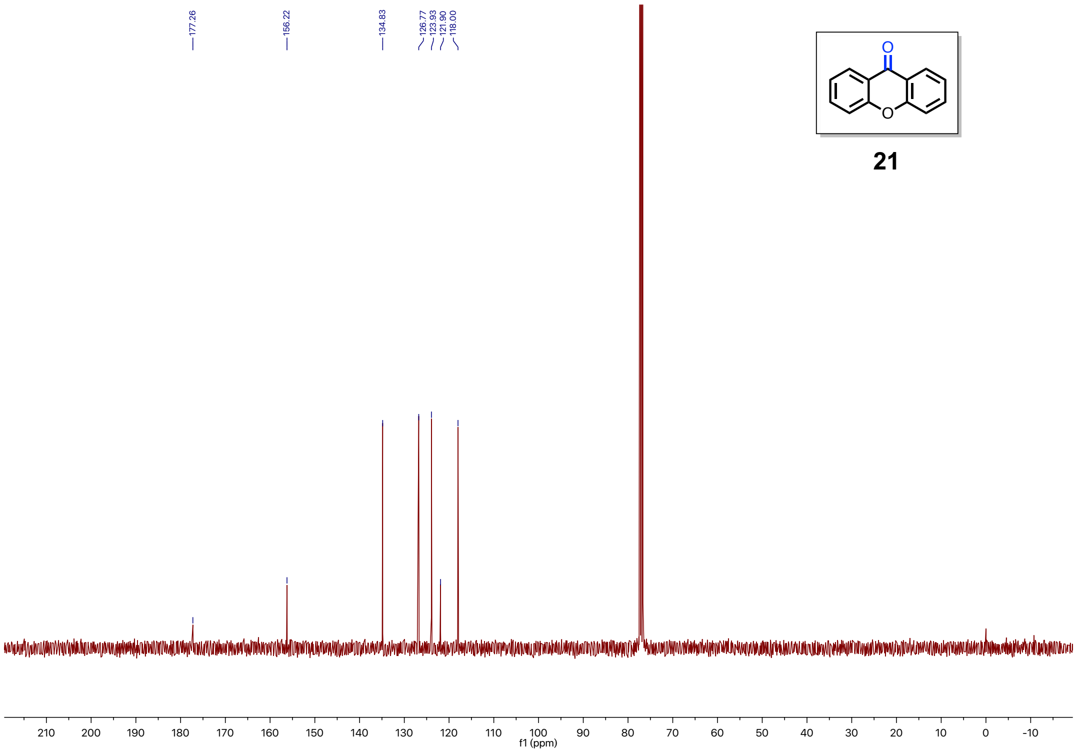

^{1}H NMR spectrum of **22** (400 MHz, $CDCl_3$)

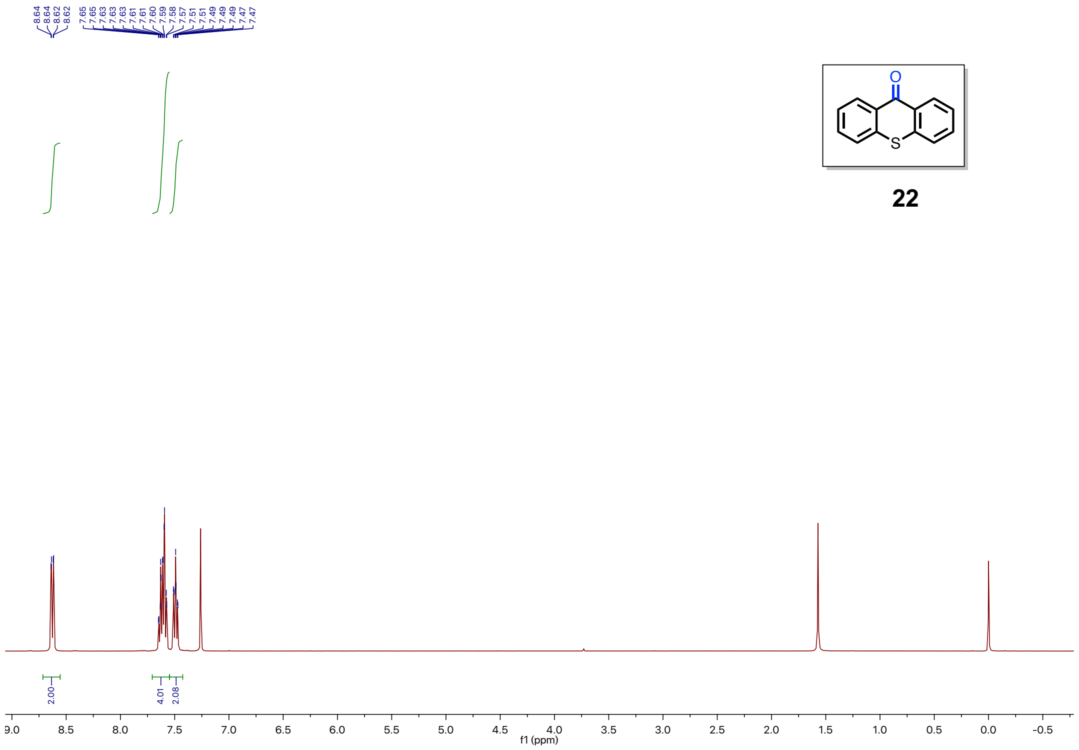

^{13}C NMR spectrum of **22** (101 MHz, $CDCl_3$)

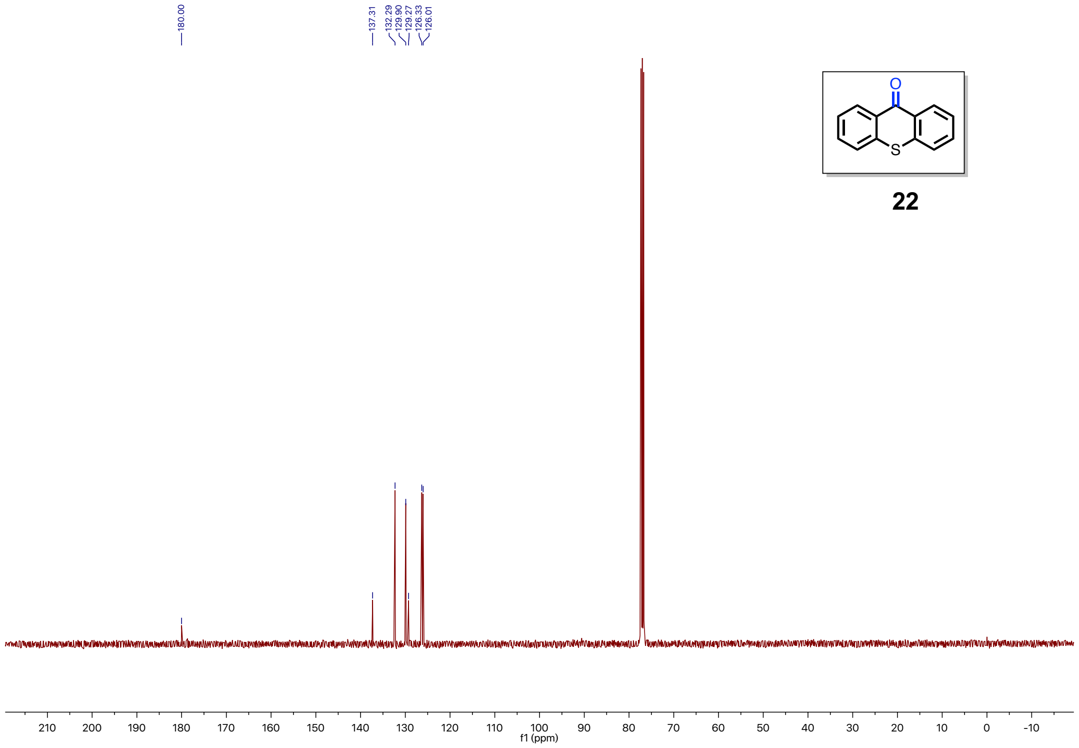

^{1}H NMR spectrum of **23** (400 MHz, $CDCl_3$)

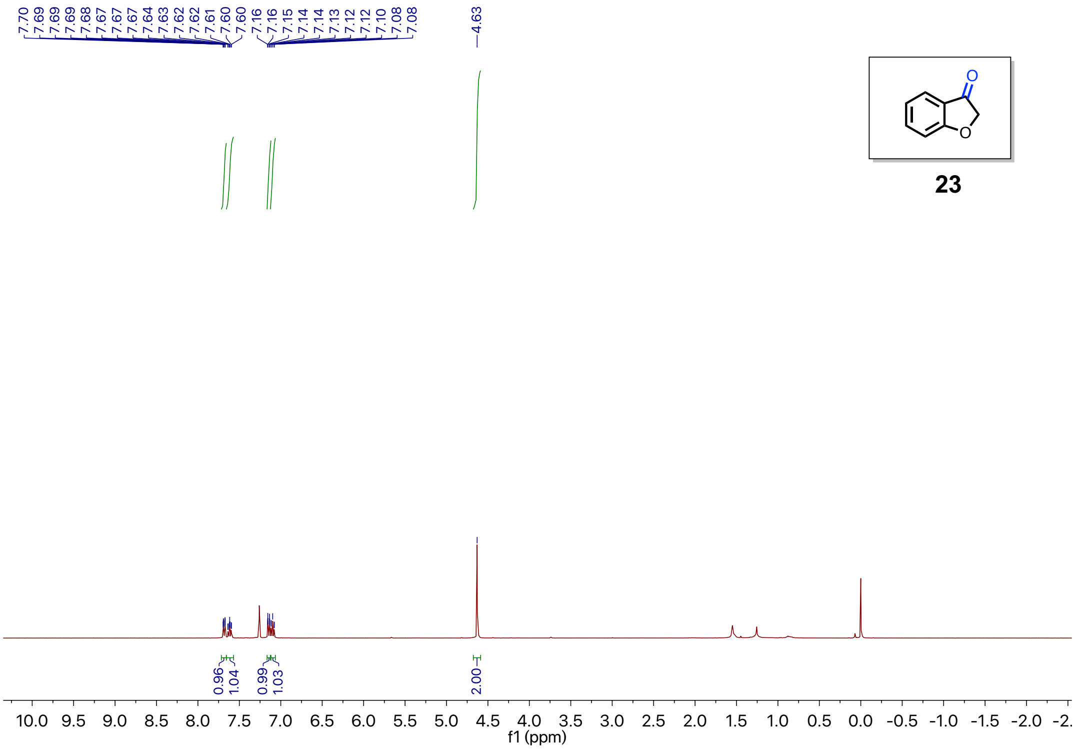

23

^{13}C NMR spectrum of **23** (101 MHz, $CDCl_3$)

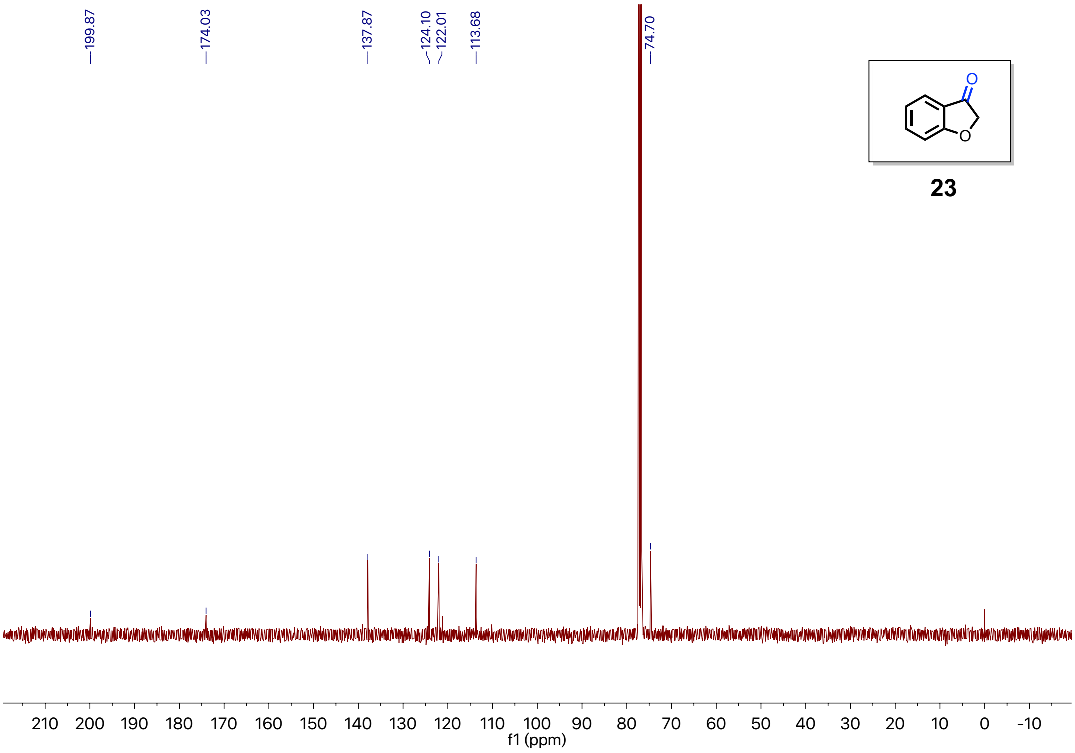

23

^{1}H NMR spectrum of **24** (400 MHz, $CDCl_3$)

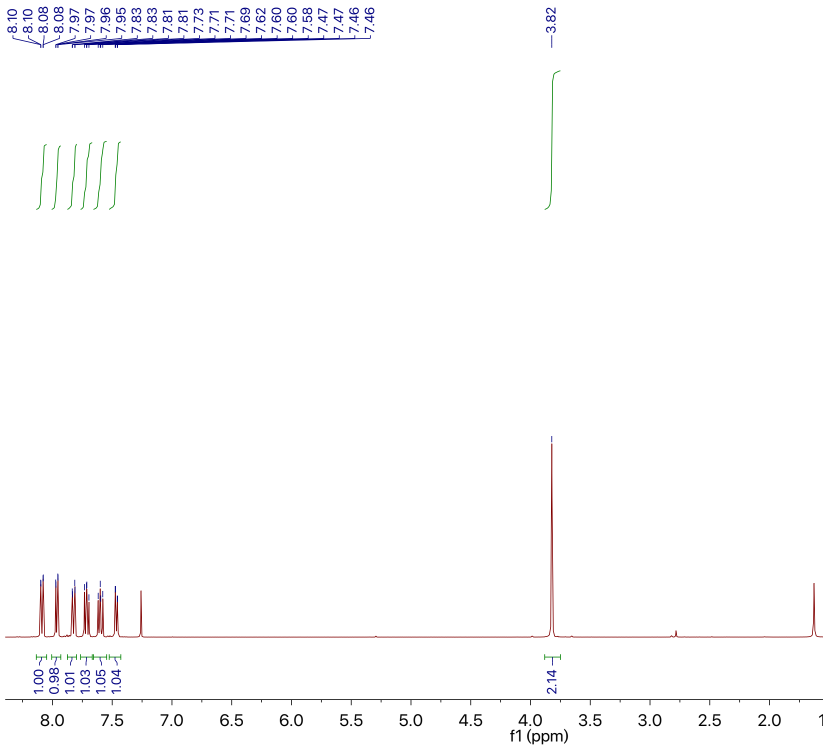

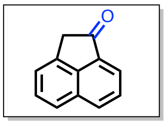

24

^{13}C NMR spectrum of **24** (101 MHz, $CDCl_3$)

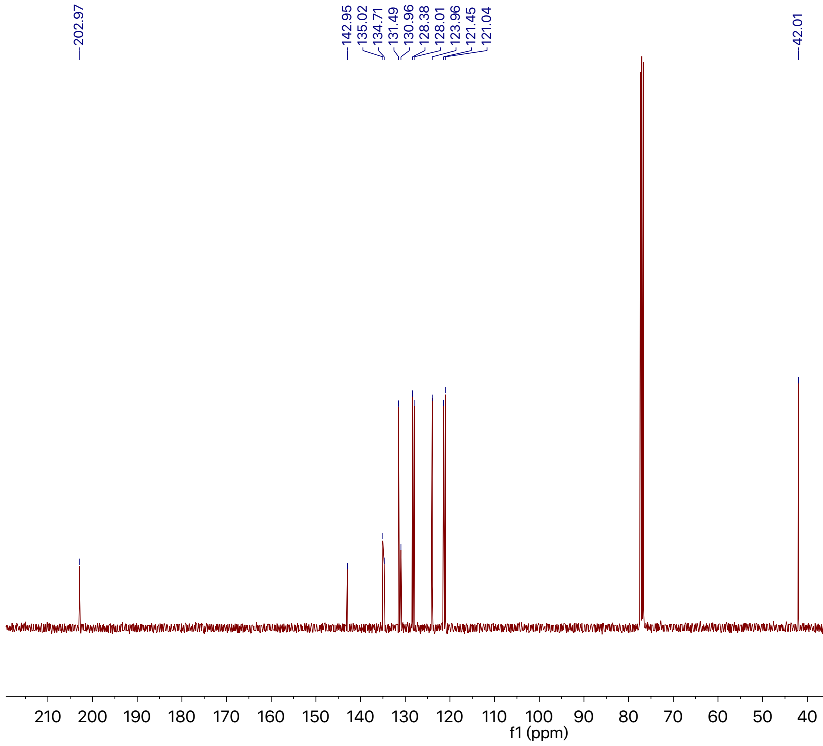

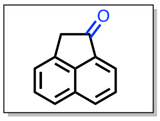

24

^{1}H NMR spectrum of **25** (400 MHz, $CDCl_3$)

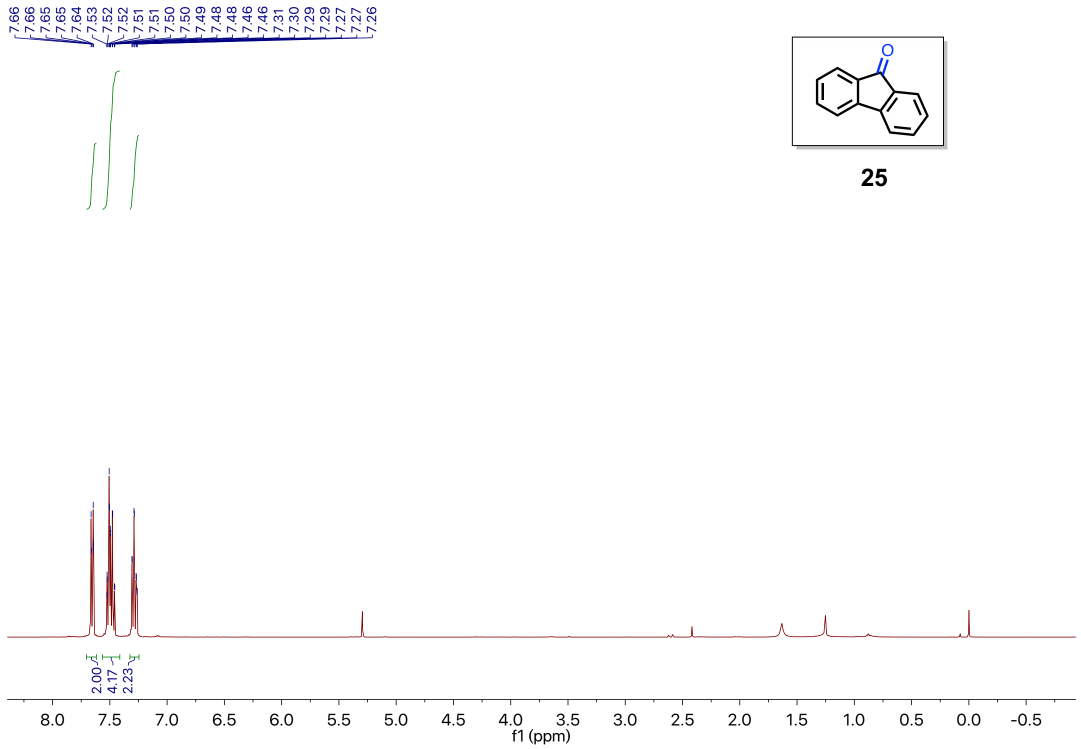

^{13}C NMR spectrum of **25** (101 MHz, $CDCl_3$)

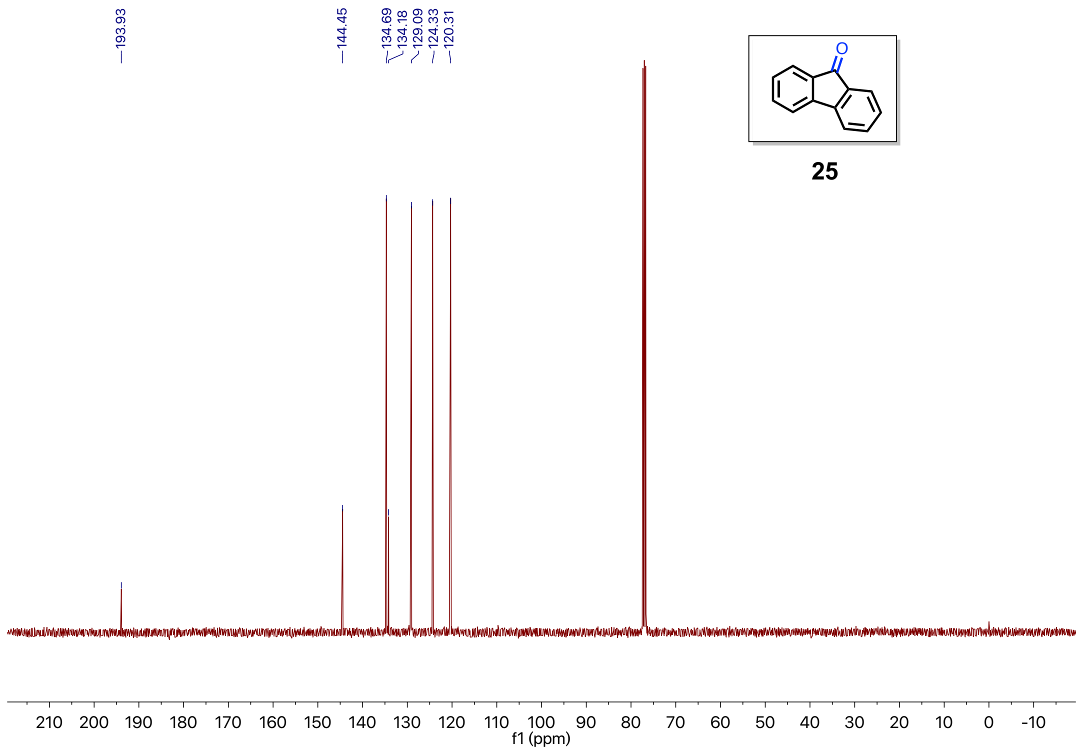

^{1}H NMR spectrum of **26** (400 MHz, $CDCl_3$)

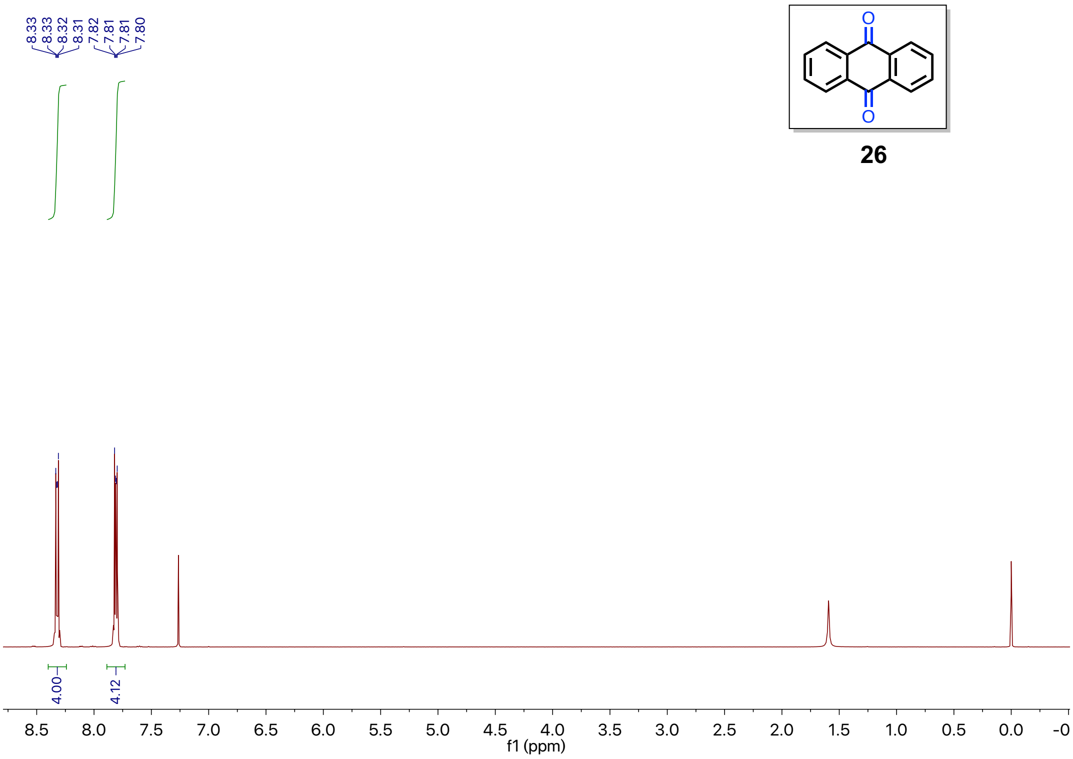

^{13}C NMR spectrum of **26** (101 MHz, $CDCl_3$)

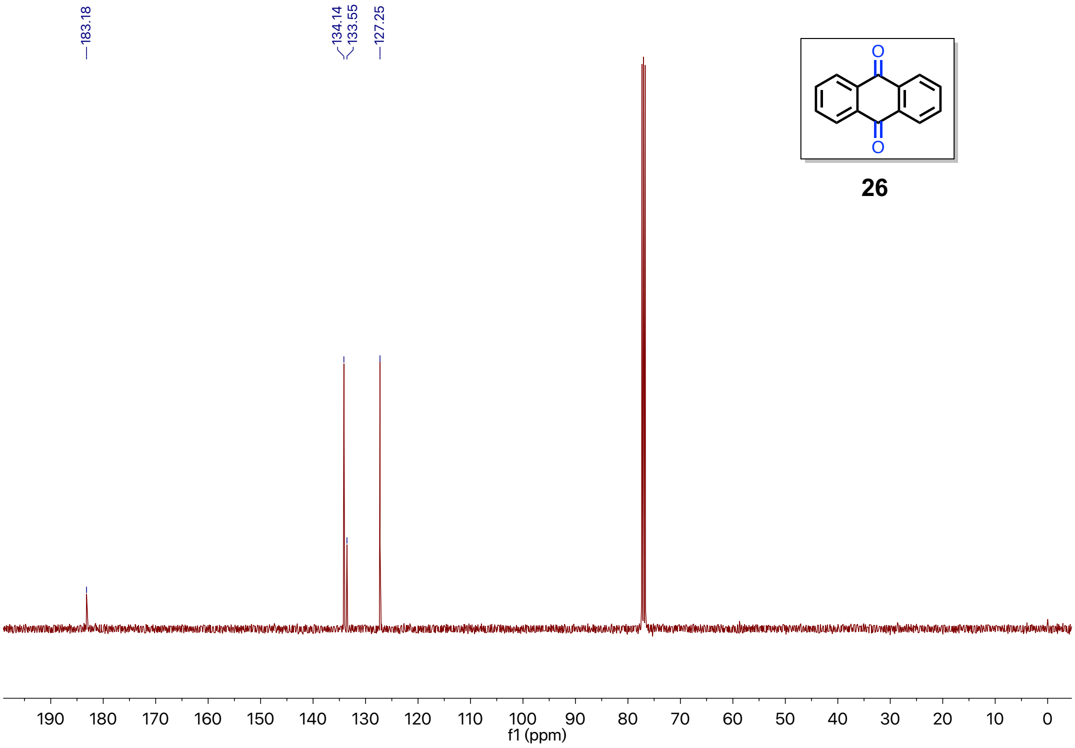

^{1}H NMR spectrum of **27** (400 MHz, $CDCl_3$)

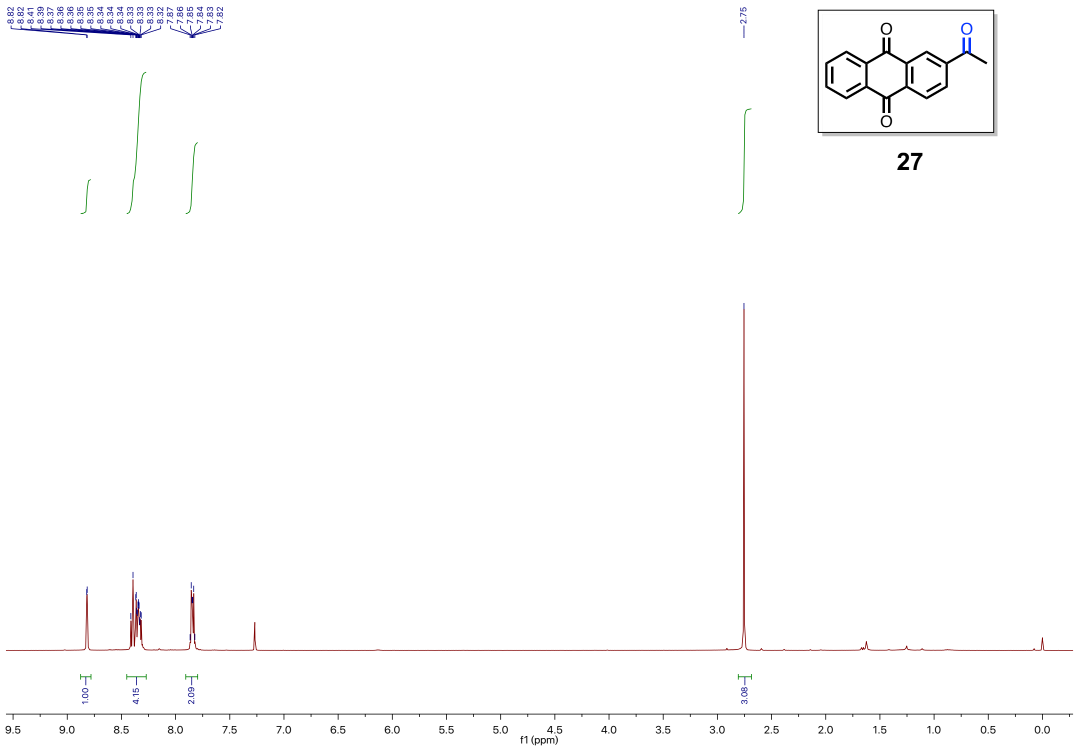

^{13}C NMR spectrum of **27** (101 MHz, $CDCl_3$)

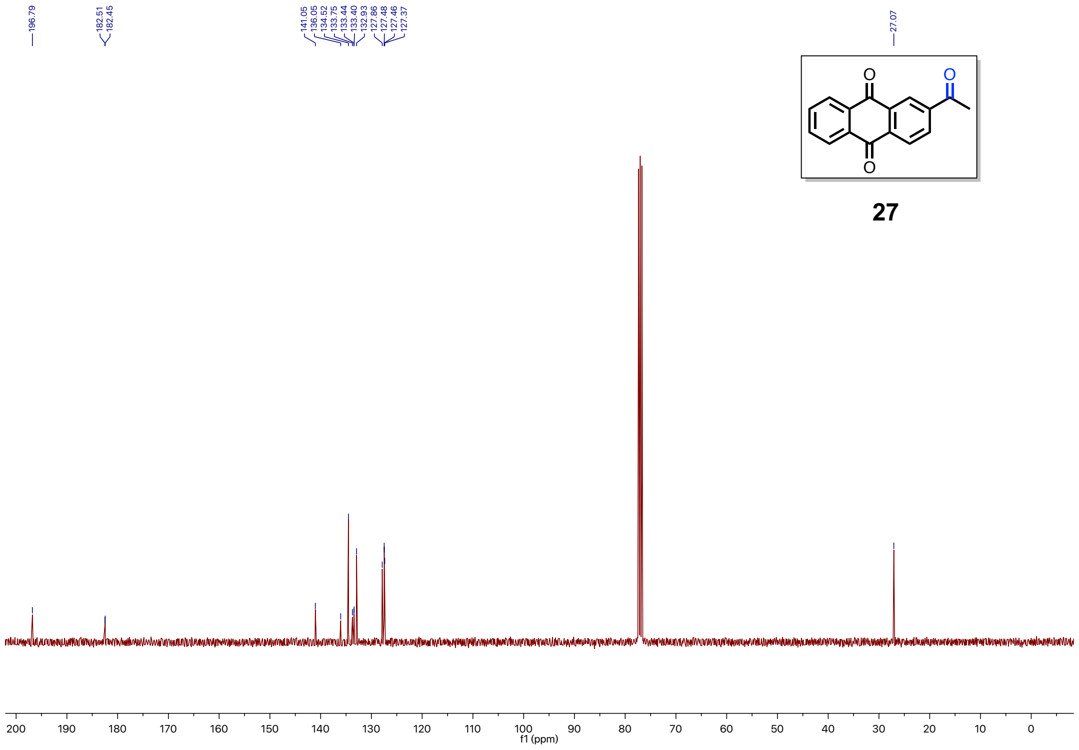

^{1}H NMR spectrum of **28** (500 MHz, CDCl$_3$)

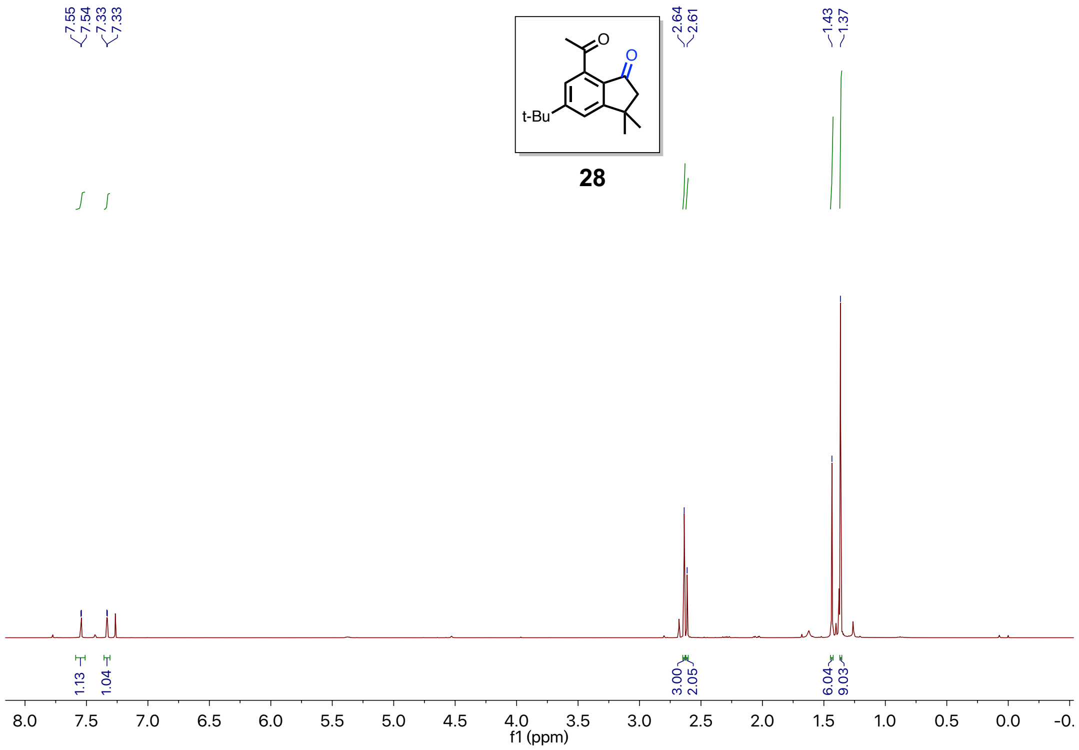

^{13}C NMR spectrum of **28** (101 MHz, CDCl$_3$)

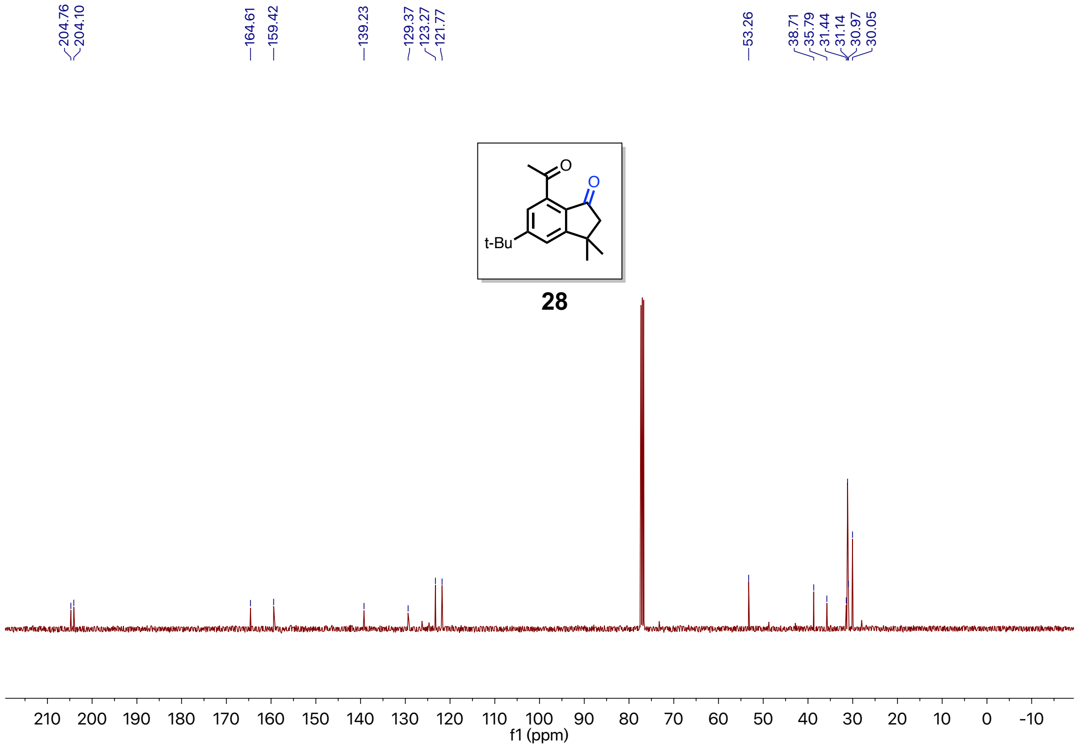

^{1}H NMR spectrum of **29** (400 MHz, $CDCl_3$)

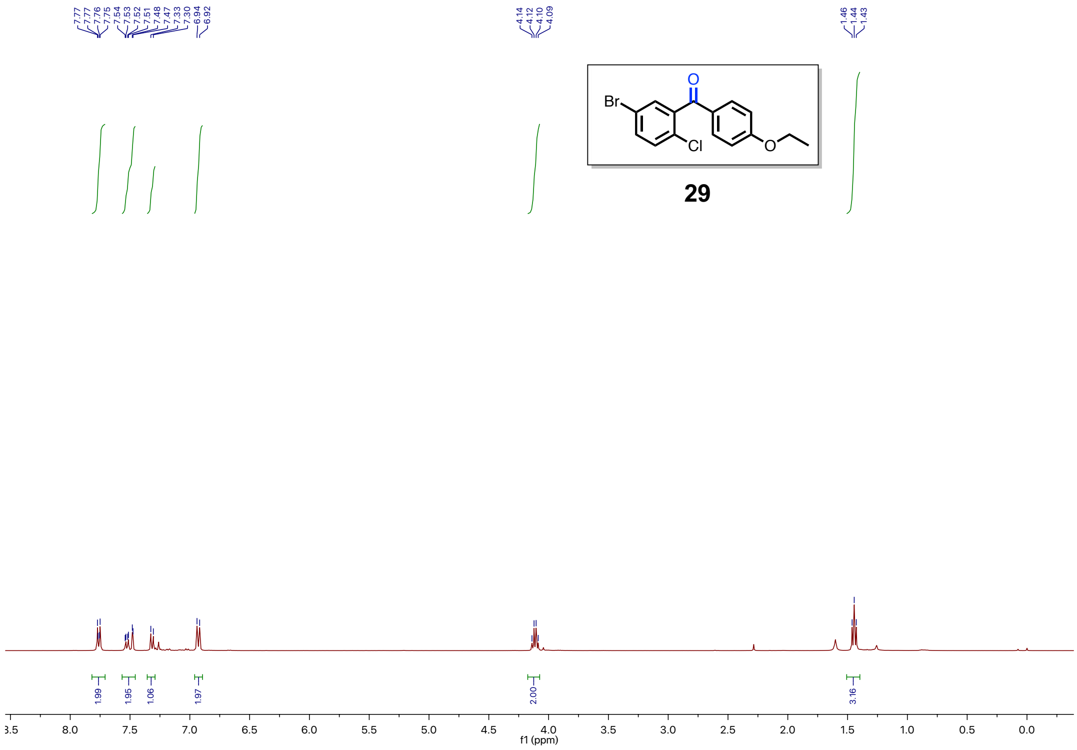

^{13}C NMR spectrum of **29** (101 MHz, $CDCl_3$)

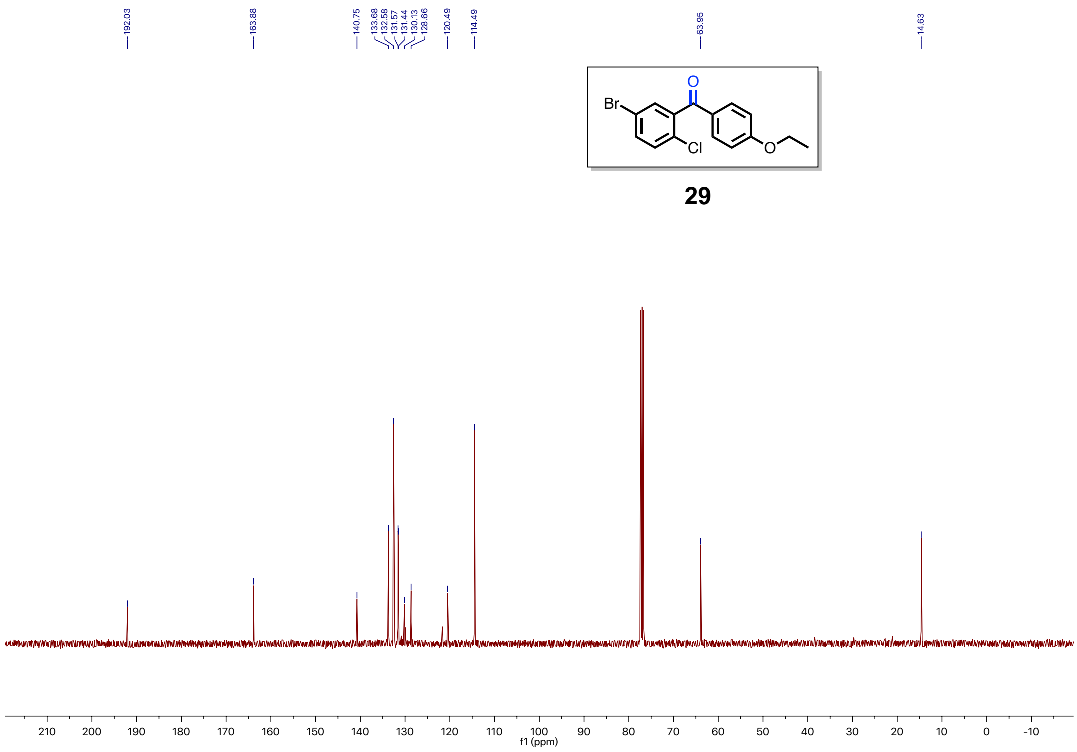

^{1}H NMR spectrum of **29b** (400 MHz, $CDCl_3$)

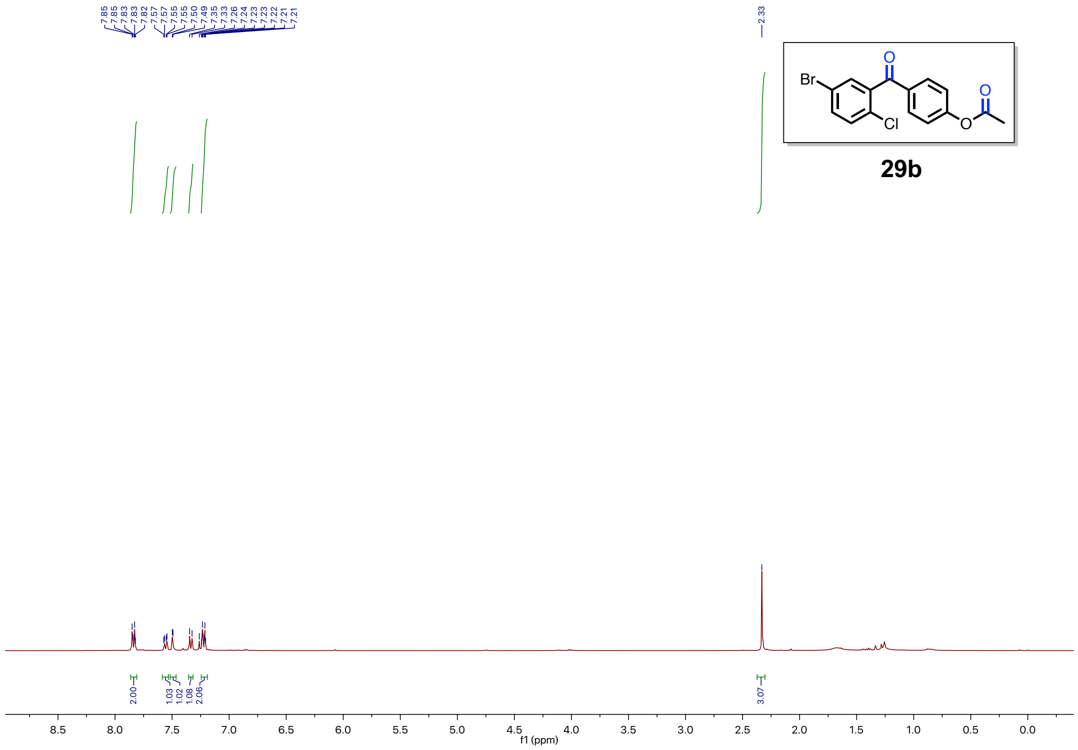

^{13}C NMR spectrum of **29b** (101 MHz, $CDCl_3$)

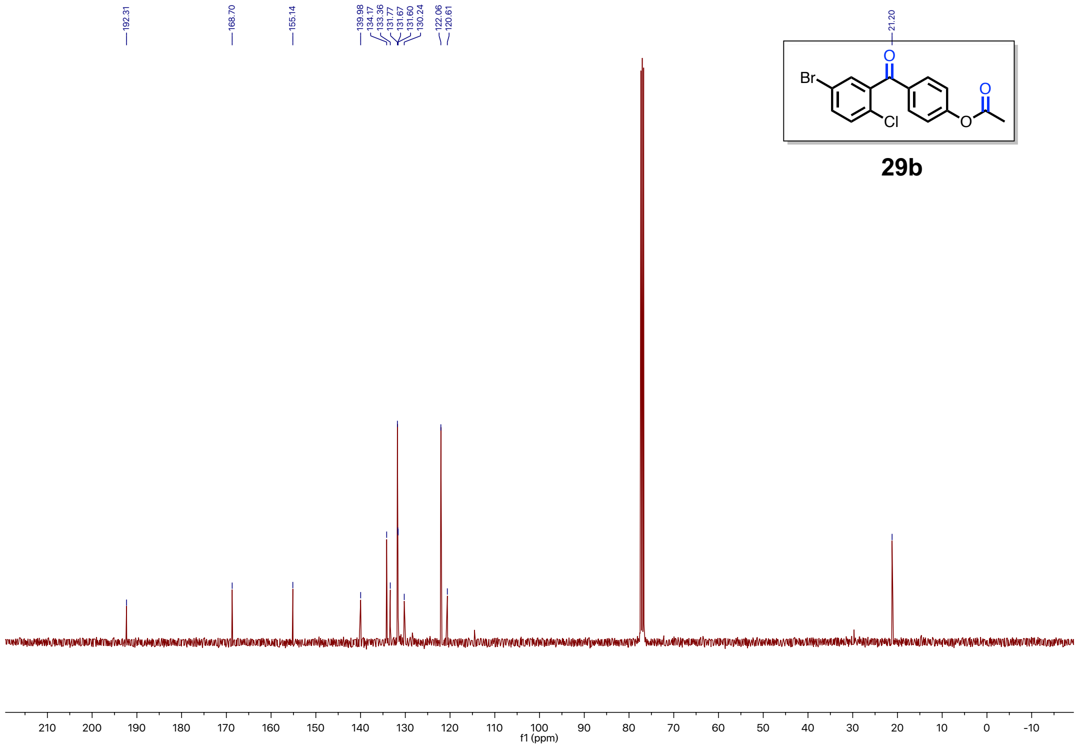

^{1}H NMR spectrum of **30** (400 MHz, $CDCl_3$)

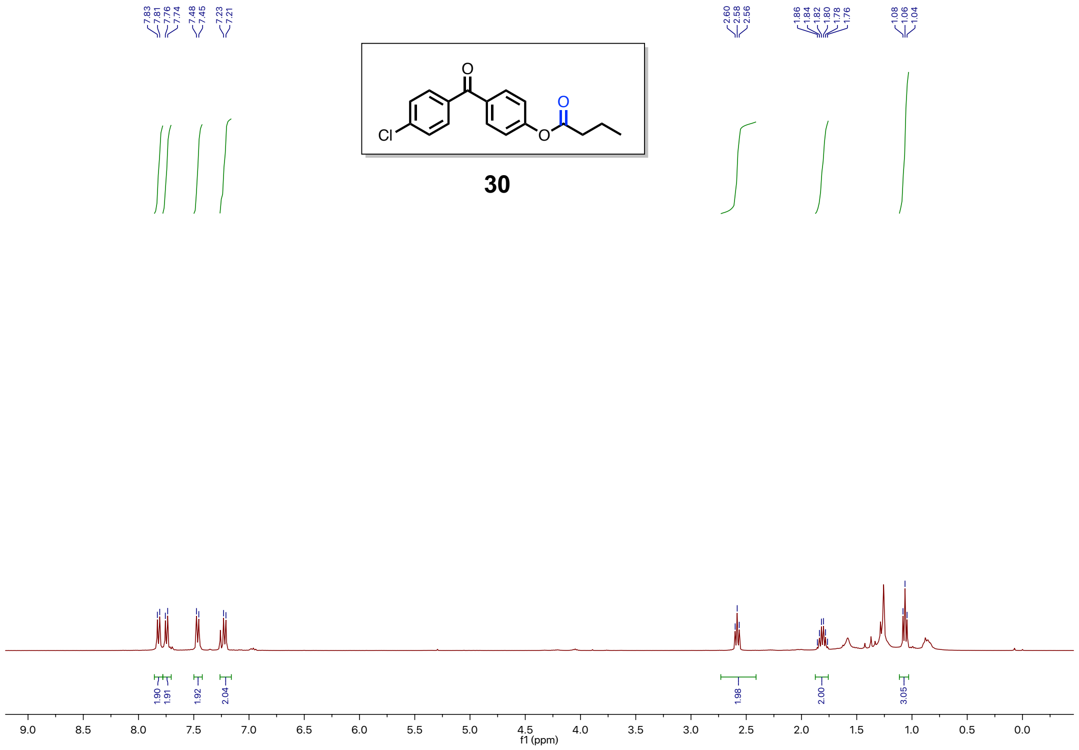

^{13}C NMR spectrum of **30** (101 MHz, $CDCl_3$)

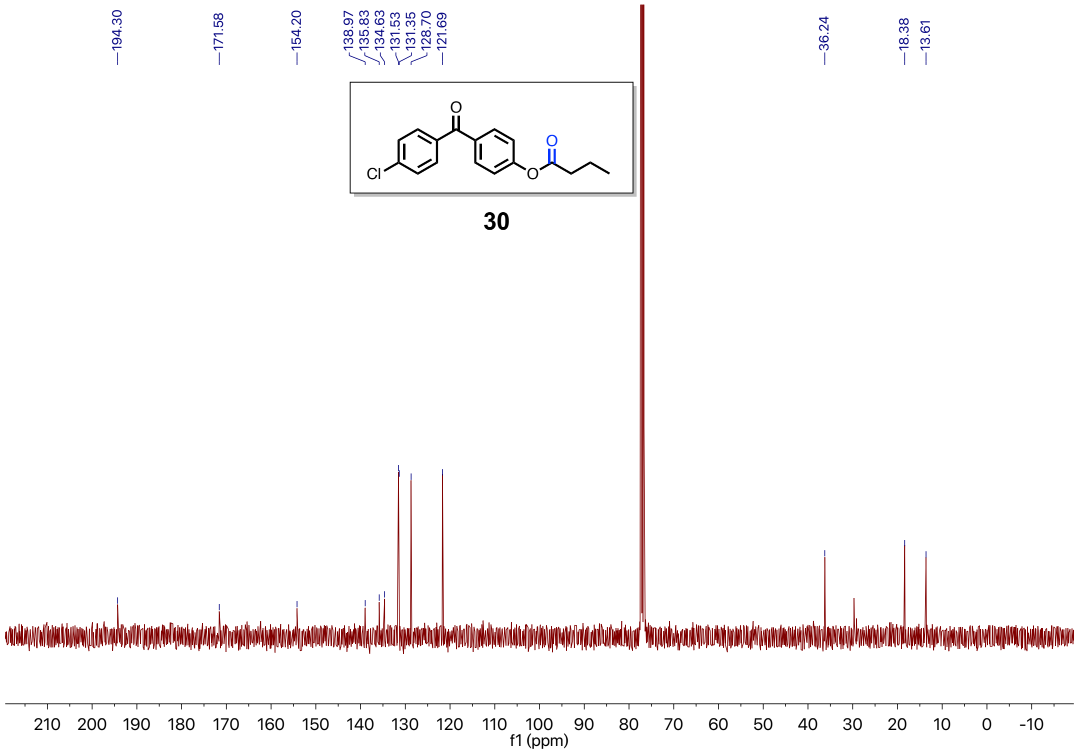

^{1}H NMR spectrum of **31** (400 MHz, $CDCl_3$)

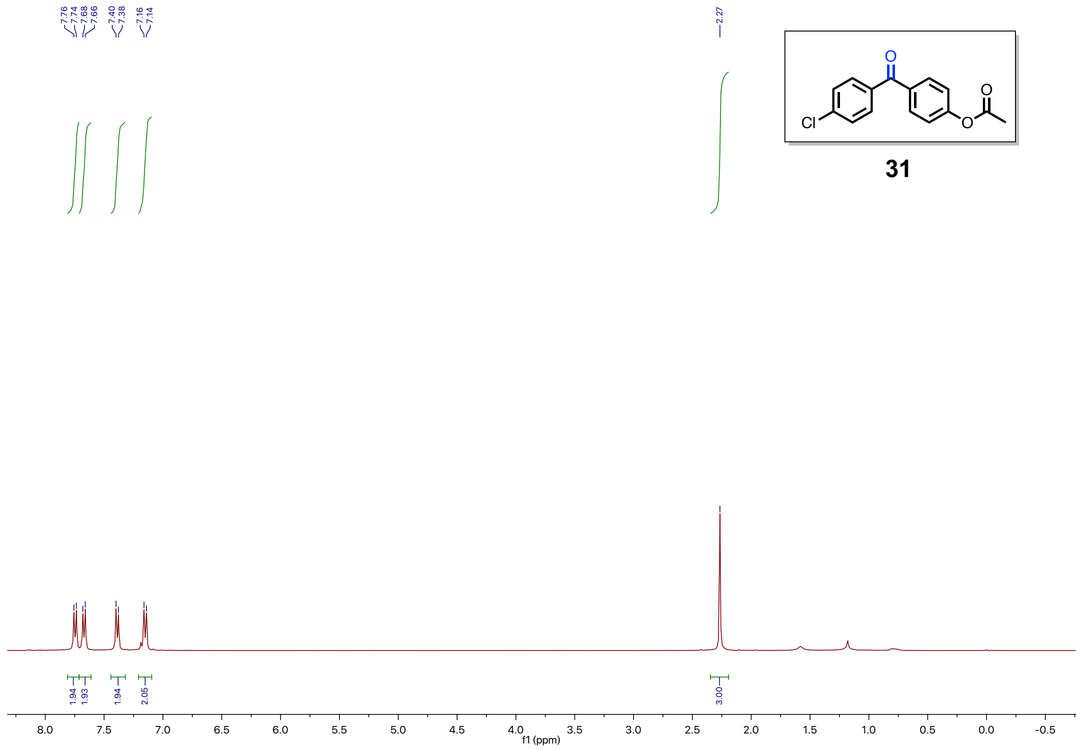

^{13}C NMR spectrum of **31** (101 MHz, $CDCl_3$)

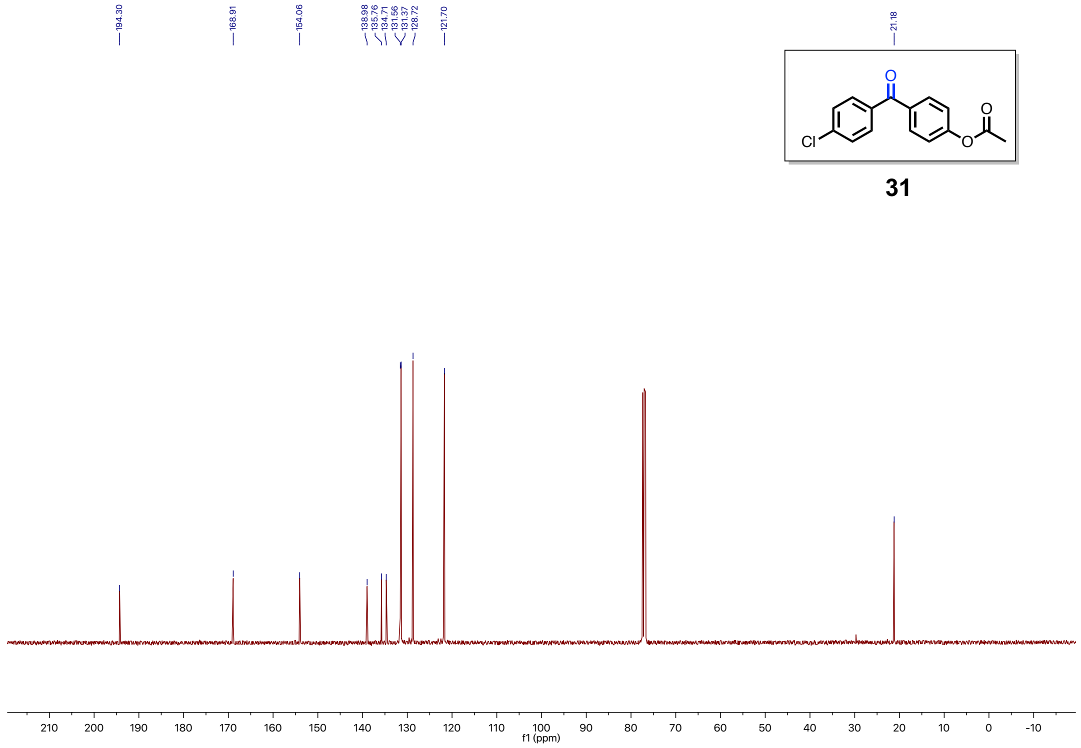

^{1}H NMR spectrum of **32** (500 MHz, DMSO-d$_6$)

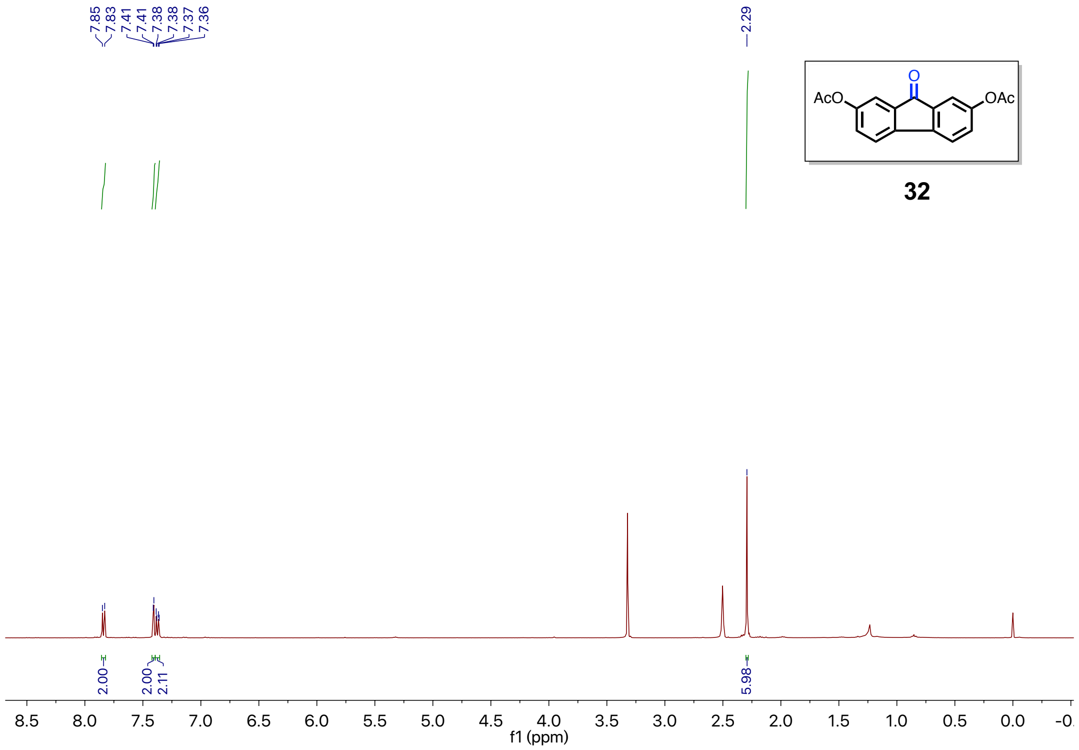

^{13}C NMR spectrum of **32** (126 MHz, DMSO-d$_6$)

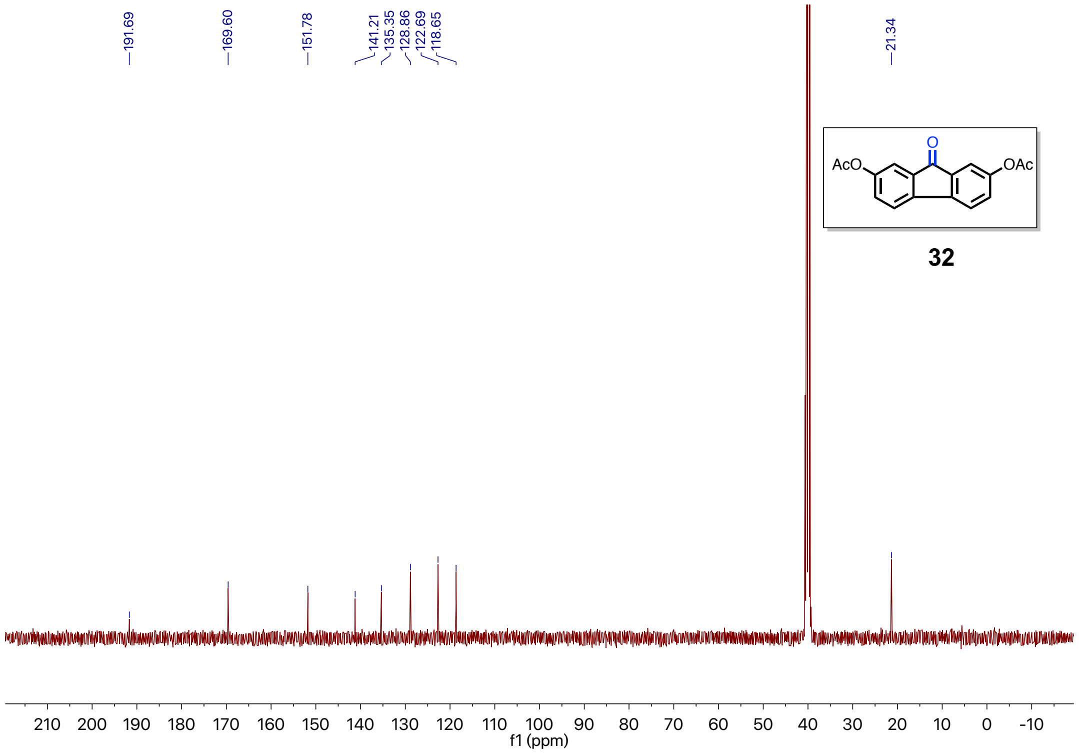

^{1}H NMR spectrum of **33** (400 MHz, $CDCl_3$)

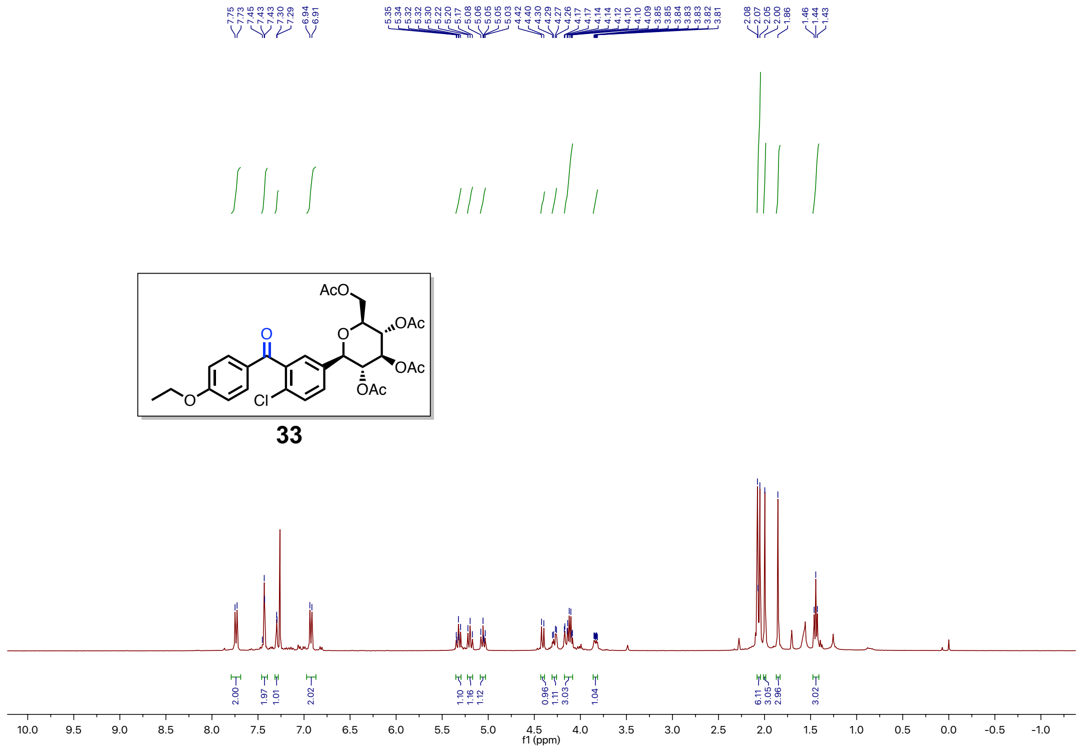

^{13}C NMR spectrum of **33** (101 MHz, $CDCl_3$)

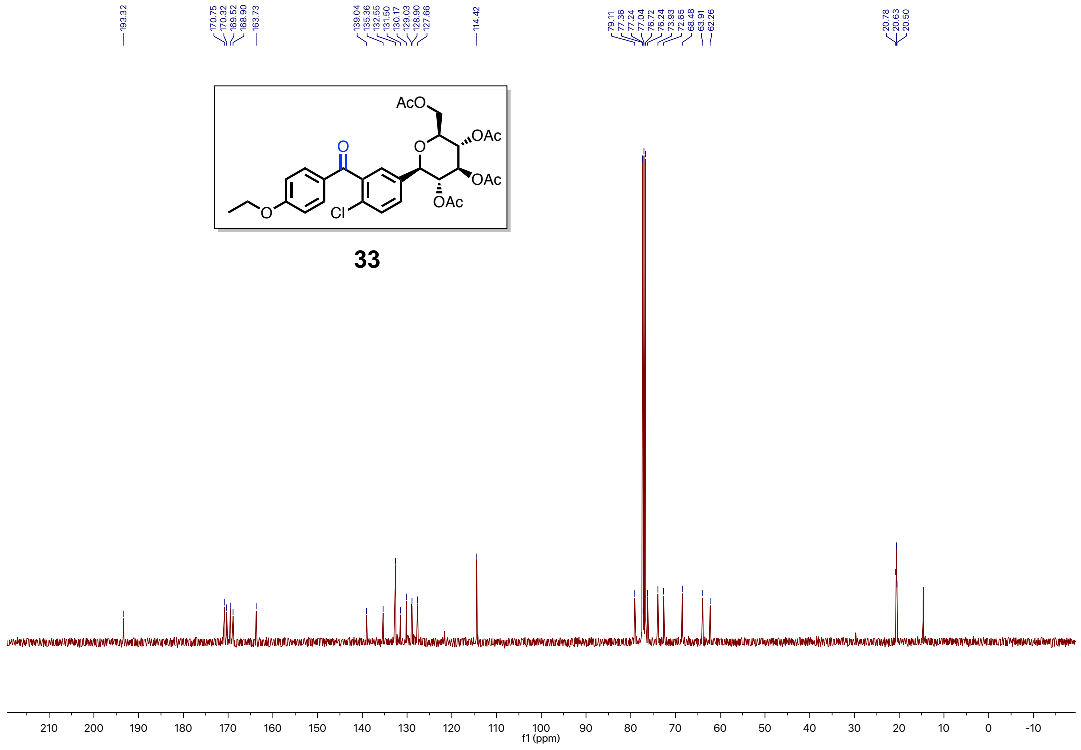

^{1}H NMR spectrum of **34** (400 MHz, $CDCl_3$)

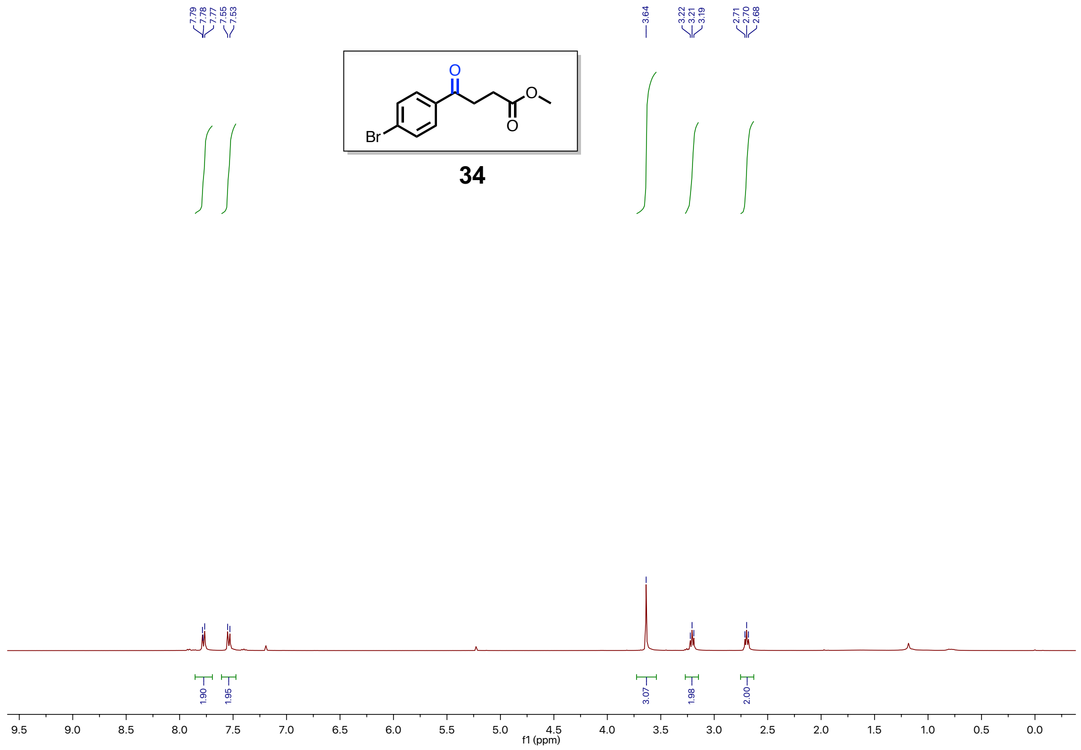

^{13}C NMR spectrum of **34** (101 MHz, $CDCl_3$)

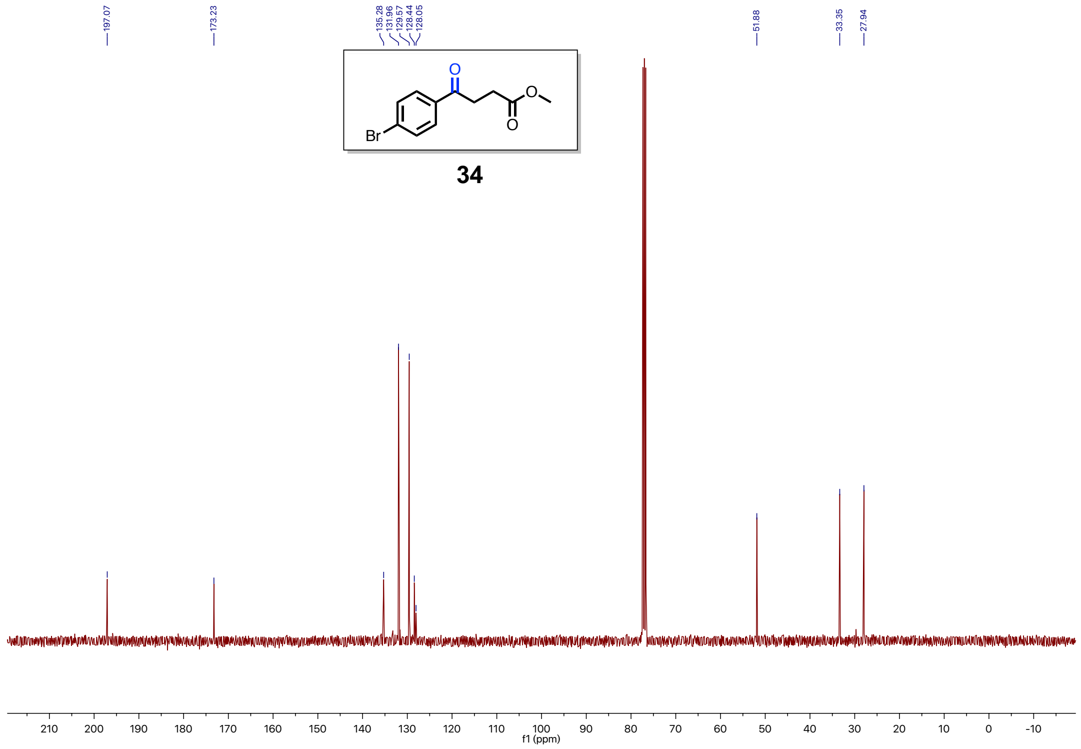

^{1}H NMR spectrum of **35** (400 MHz, $CDCl_3$)

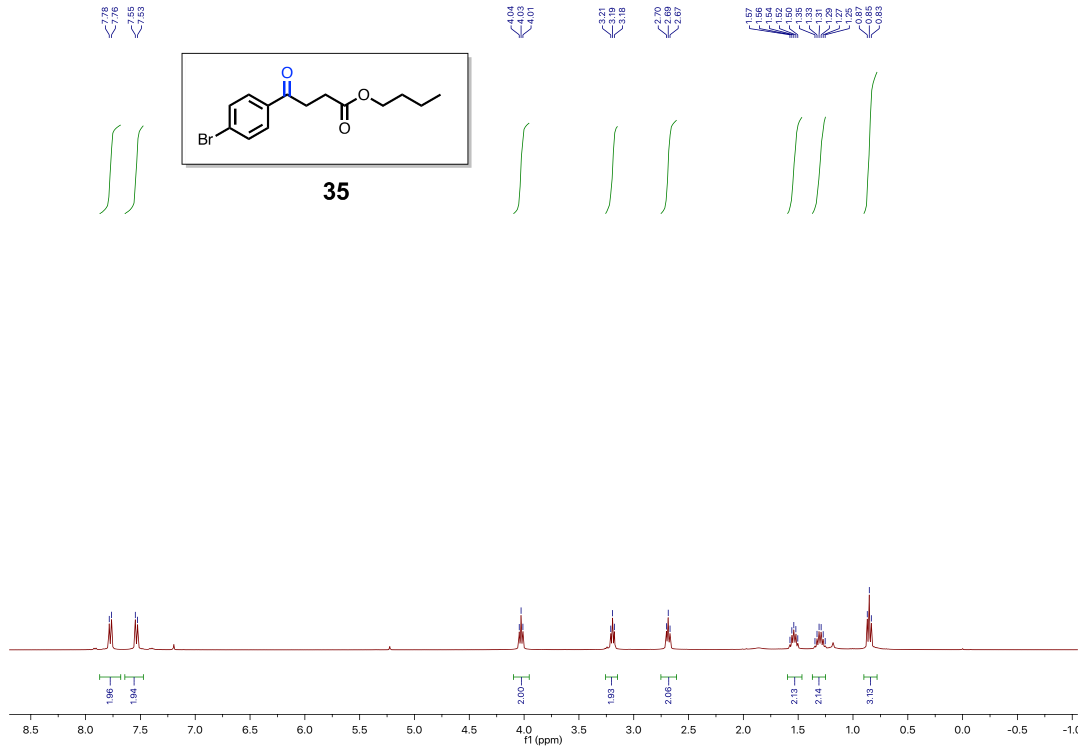

^{13}C NMR spectrum of **35** (101 MHz, $CDCl_3$)

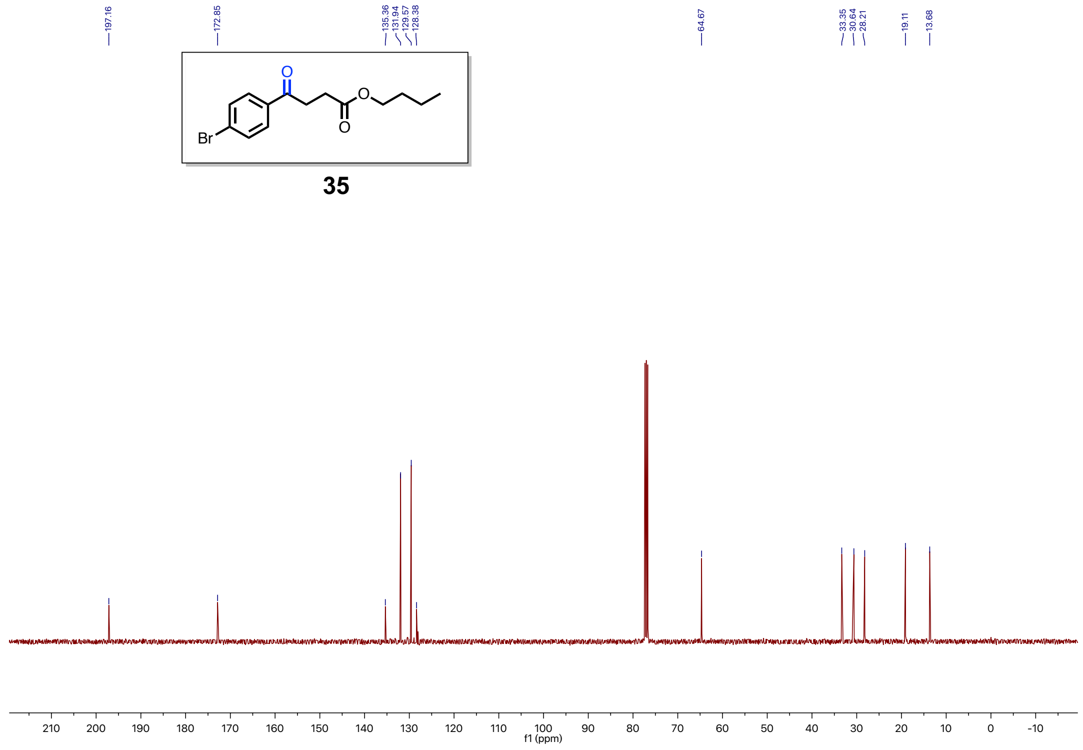

^{1}H NMR spectrum of **36** (400 MHz, $CDCl_3$)

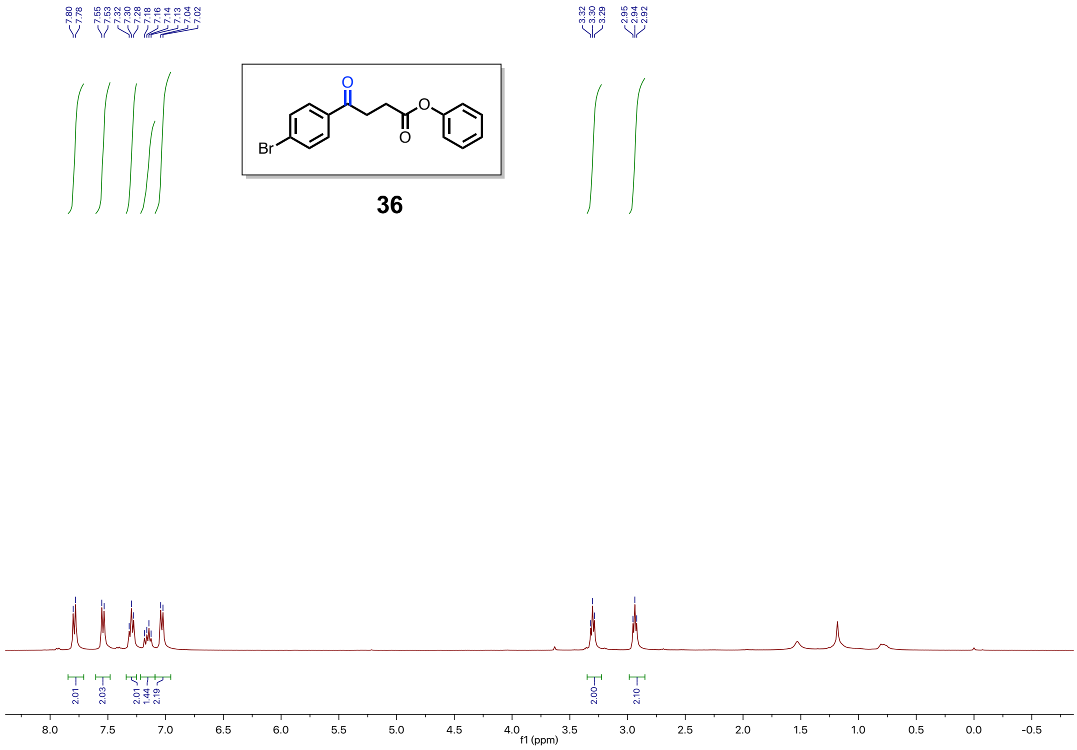

^{13}C NMR spectrum of **36** (101 MHz, $CDCl_3$)

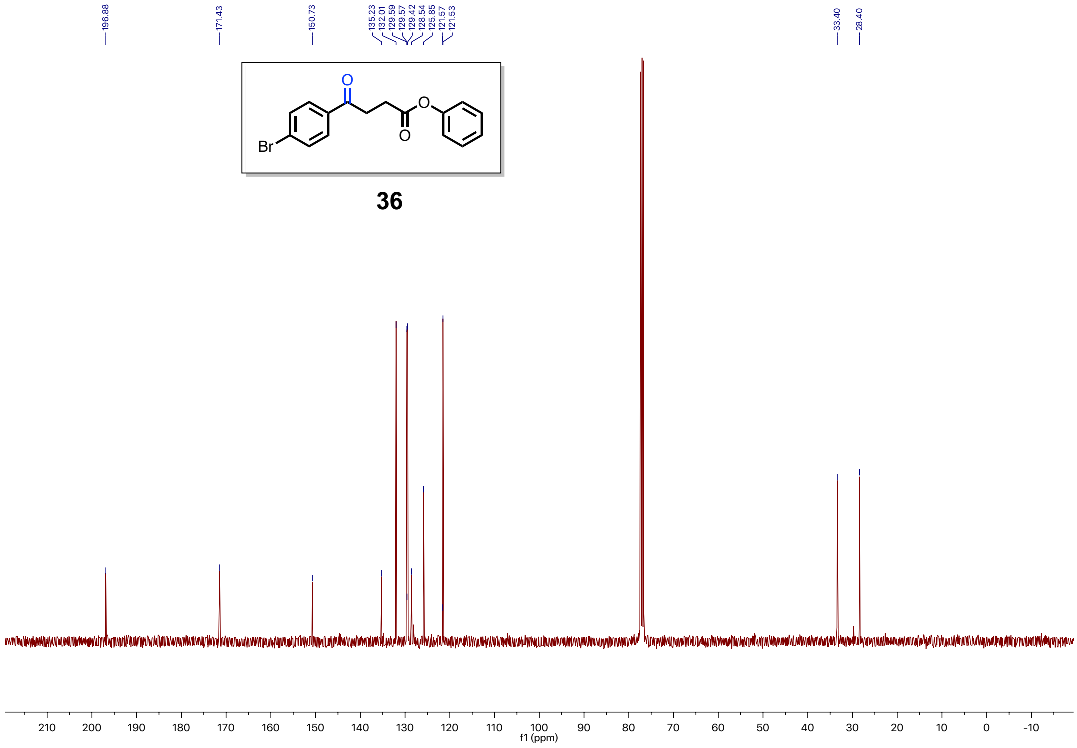

^{1}H NMR spectrum of **37** (400 MHz, $CDCl_3$)

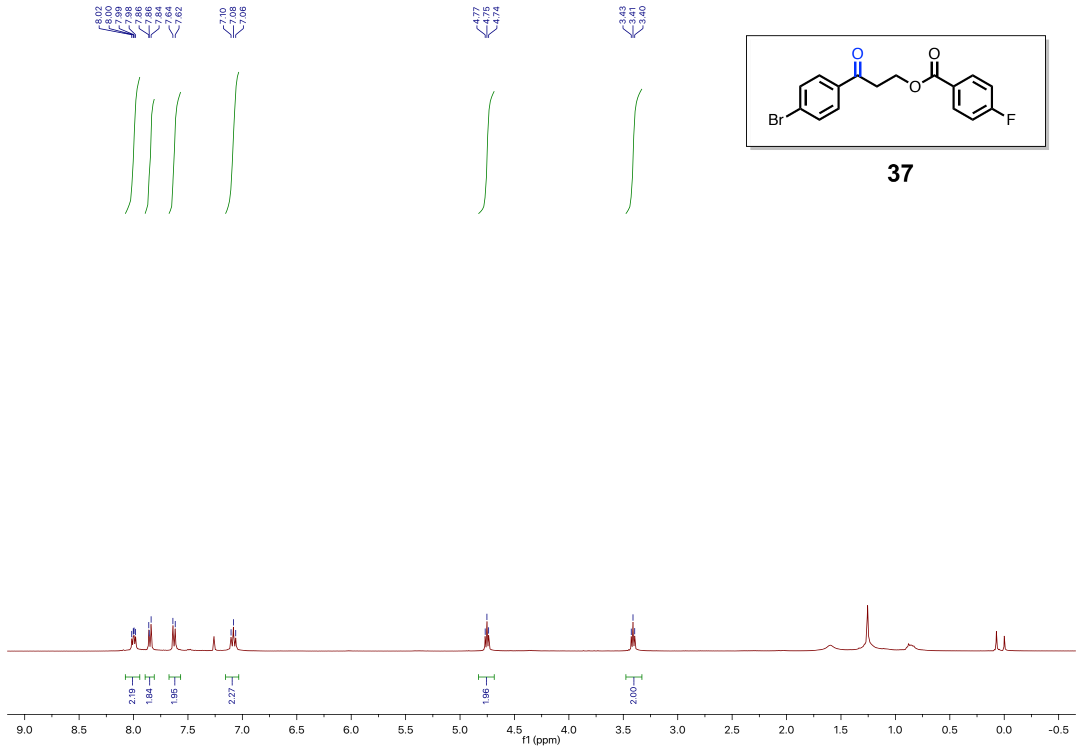

^{13}C NMR spectrum of **37** (101 MHz, $CDCl_3$)

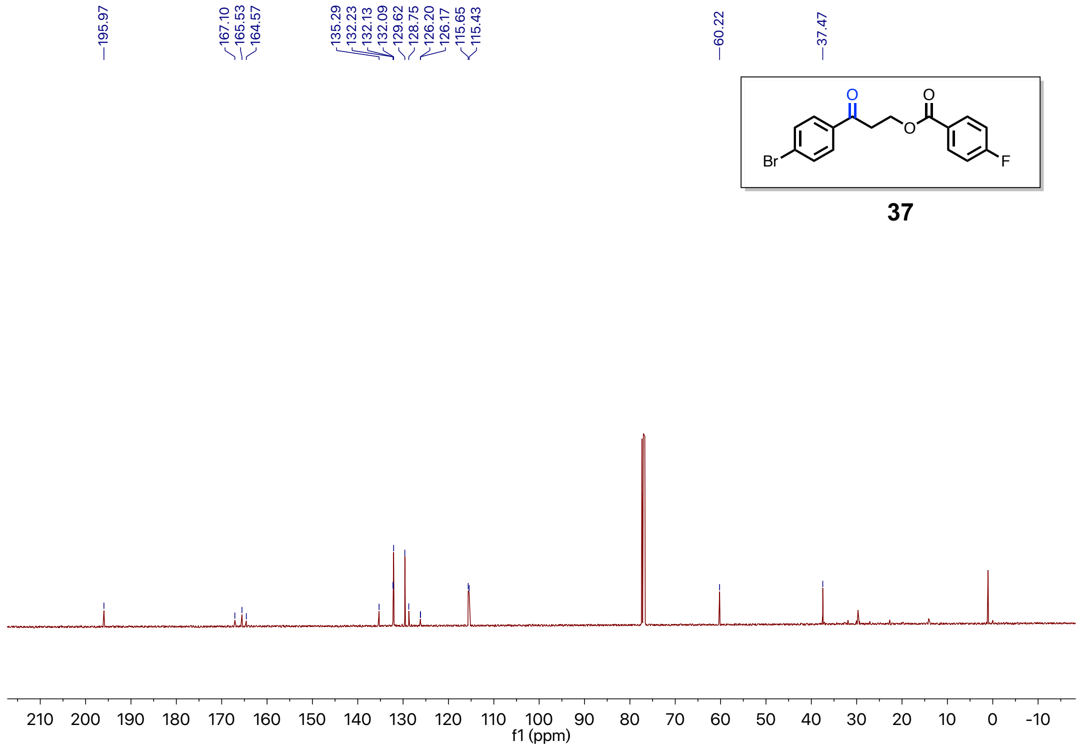

^{19}F NMR spectrum of **37** (376 MHz, $CDCl_3$)

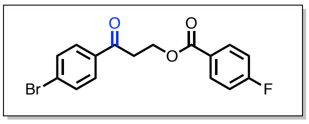

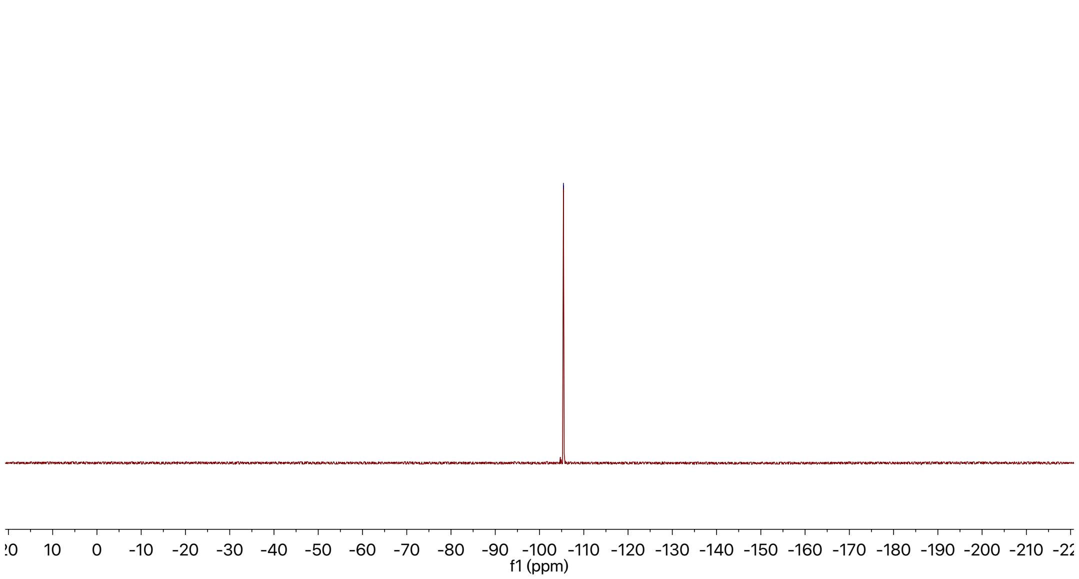

^{1}H NMR spectrum of **38** (400 MHz, $CDCl_3$)

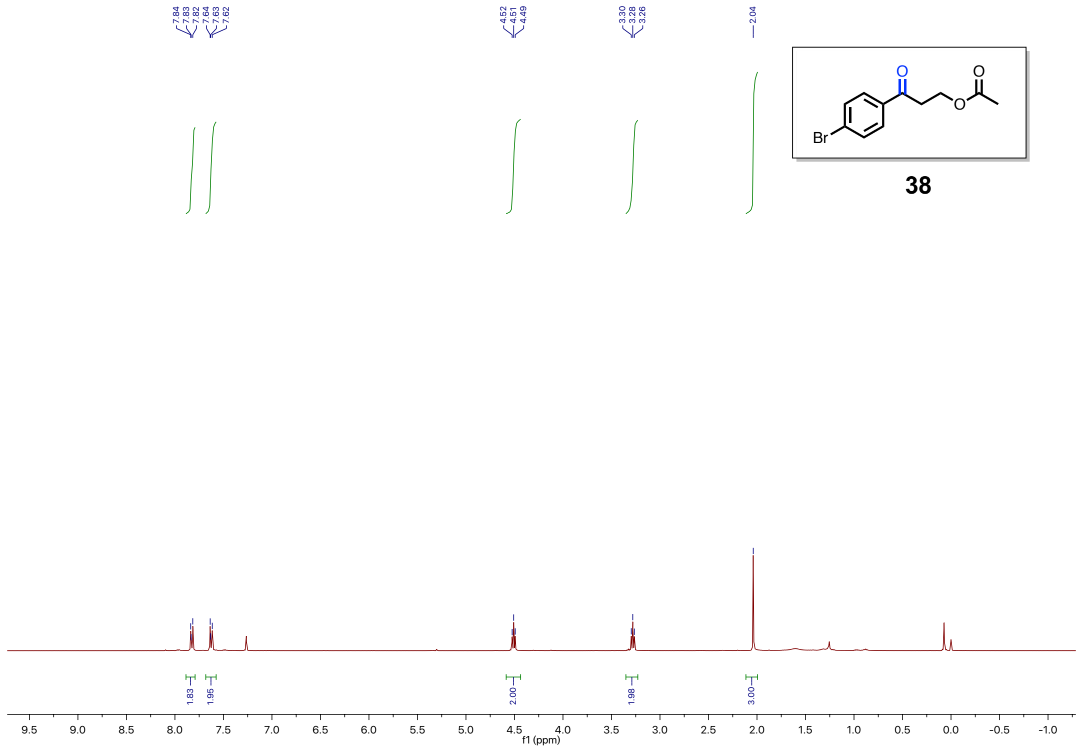

^{13}C NMR spectrum of **38** (101 MHz, $CDCl_3$)

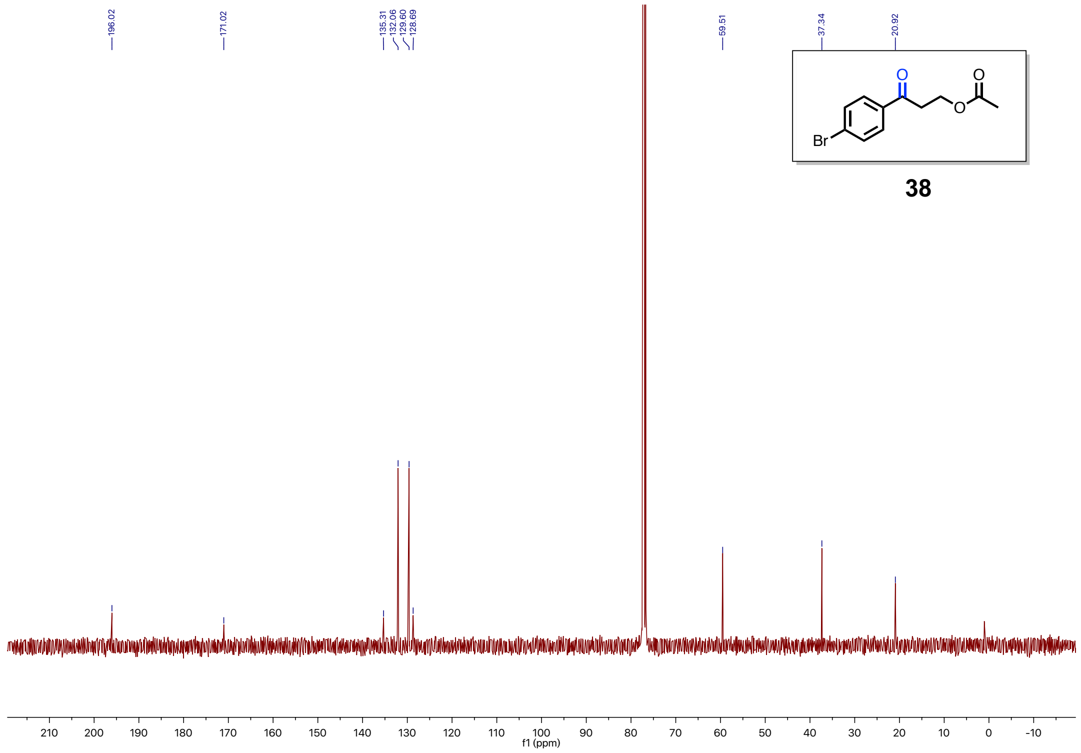

^{1}H NMR spectrum of **40** (400 MHz, $CDCl_3$)

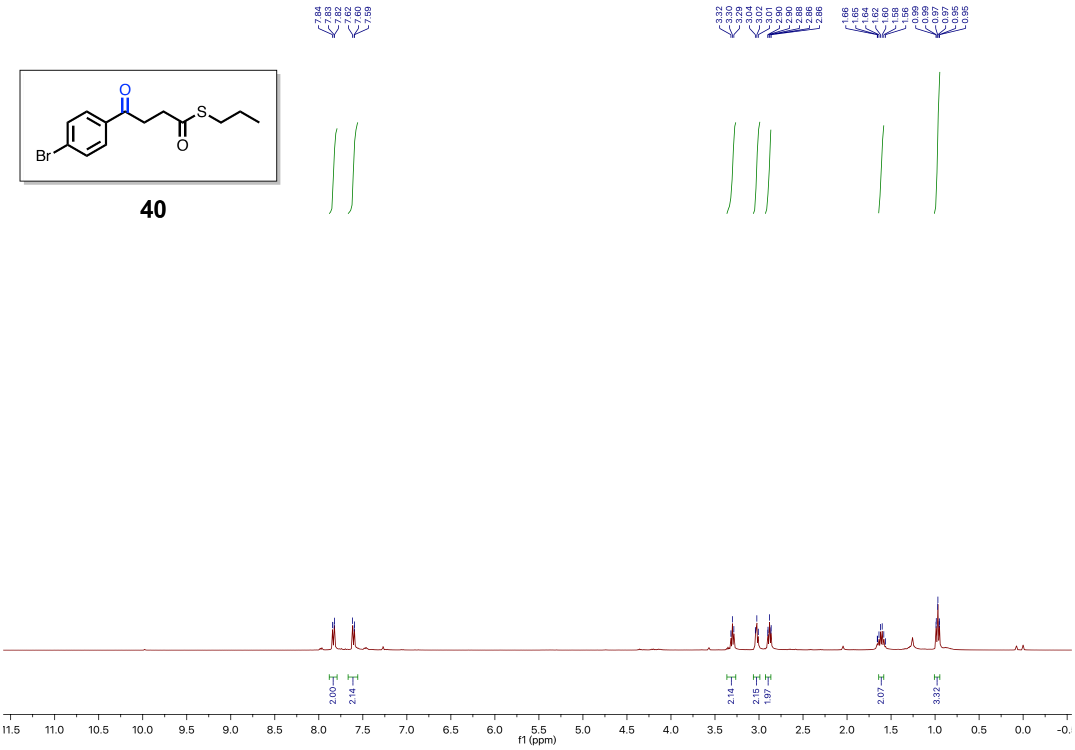

^{13}C NMR spectrum of **40** (101 MHz, $CDCl_3$)

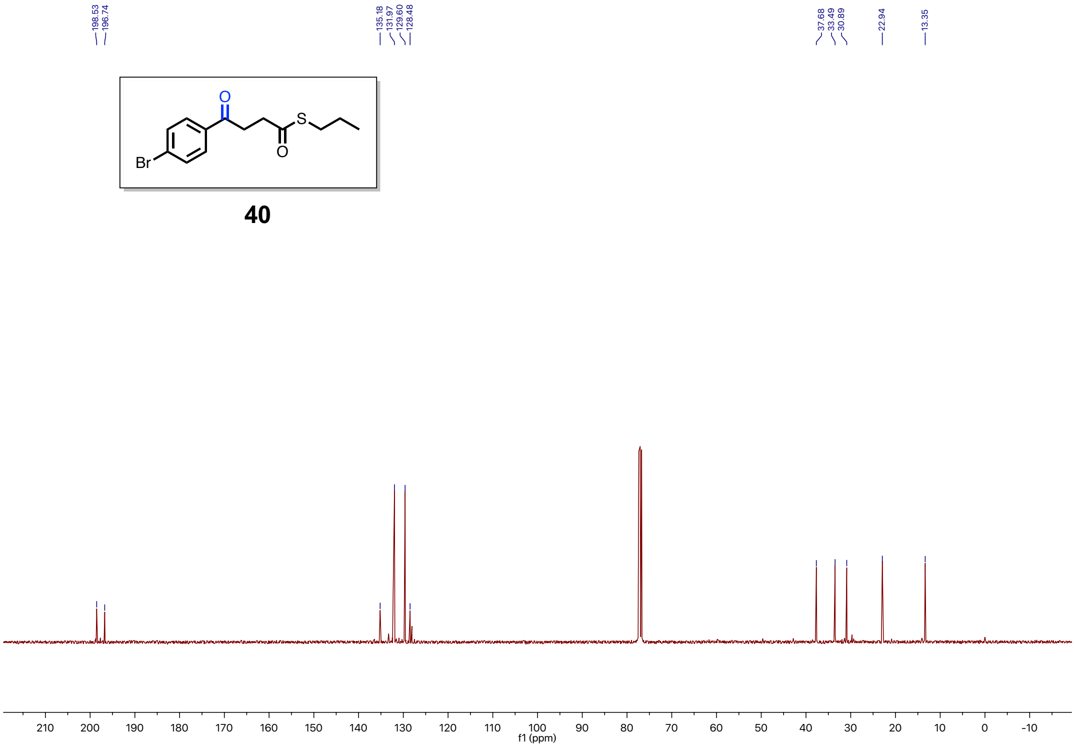

^{1}H NMR spectrum of **41** (500 MHz, $CDCl_3$)

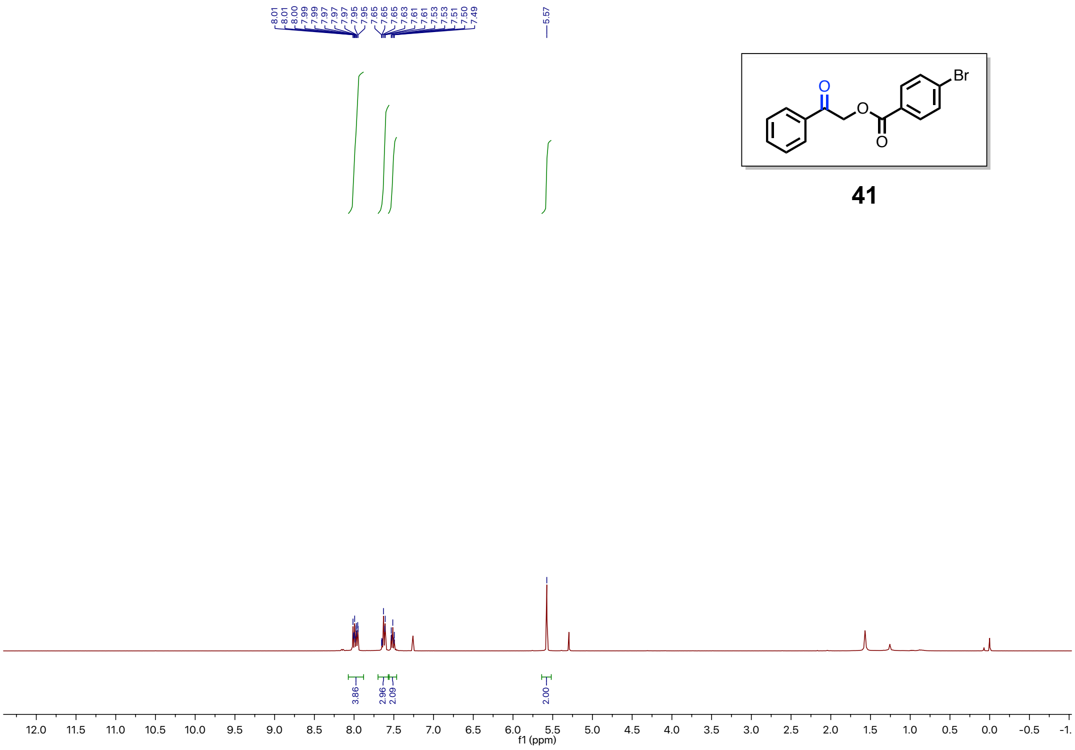

^{13}C NMR spectrum of **41** (101 MHz, $CDCl_3$)

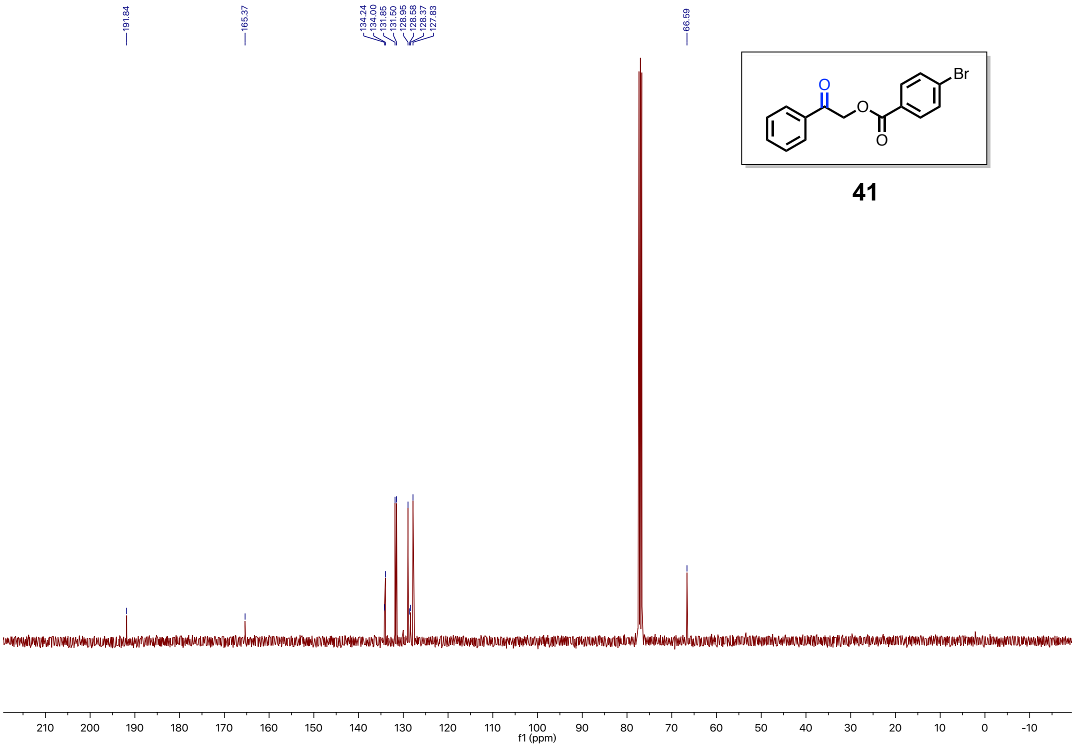

^{1}H NMR spectrum of **42** (400 MHz, $CDCl_3$)

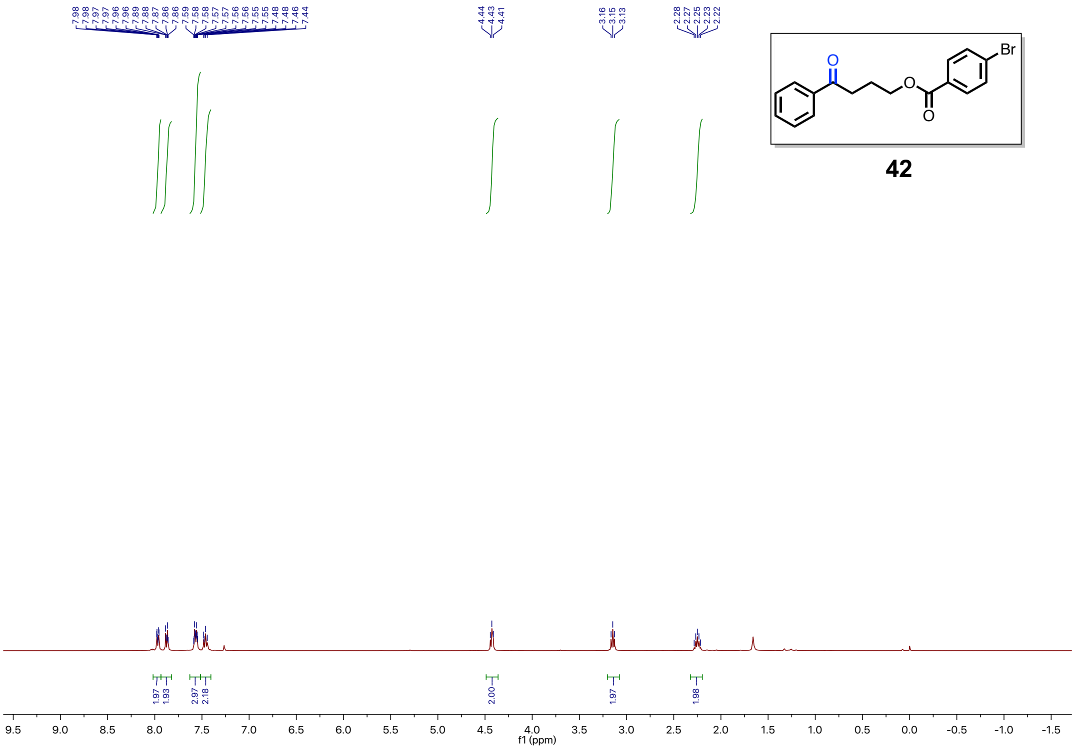

42

^{13}C NMR spectrum of **42** (126 MHz, $CDCl_3$)

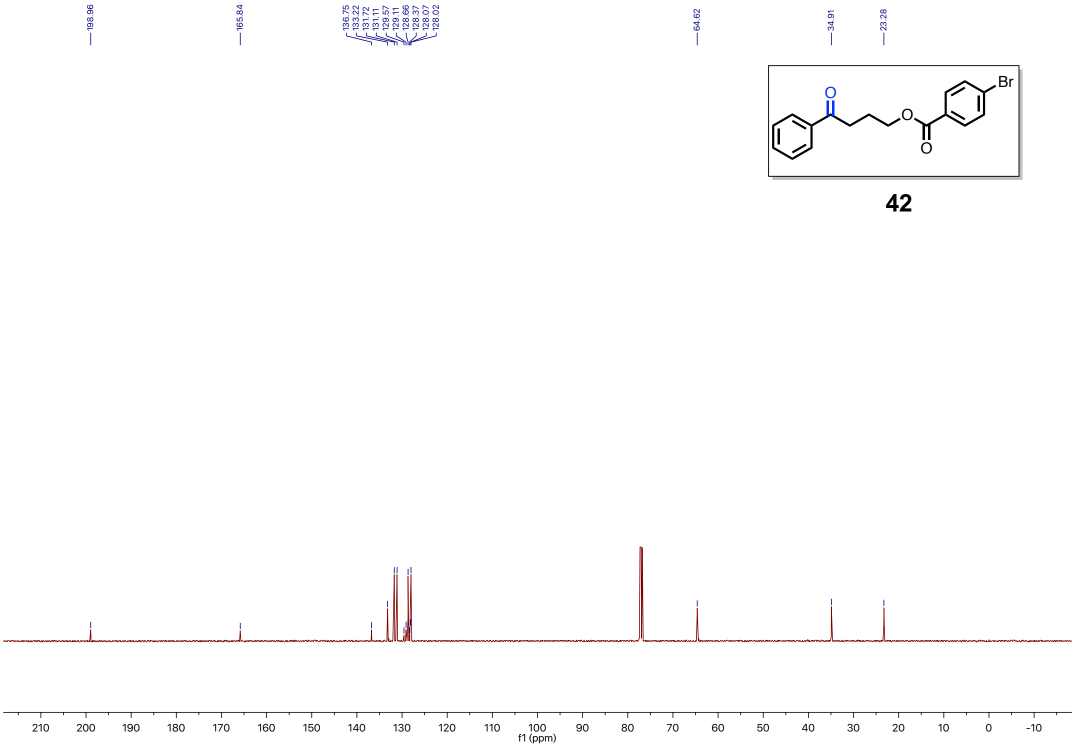

42

^{1}H NMR spectrum of **43** (400 MHz, $CDCl_3$)

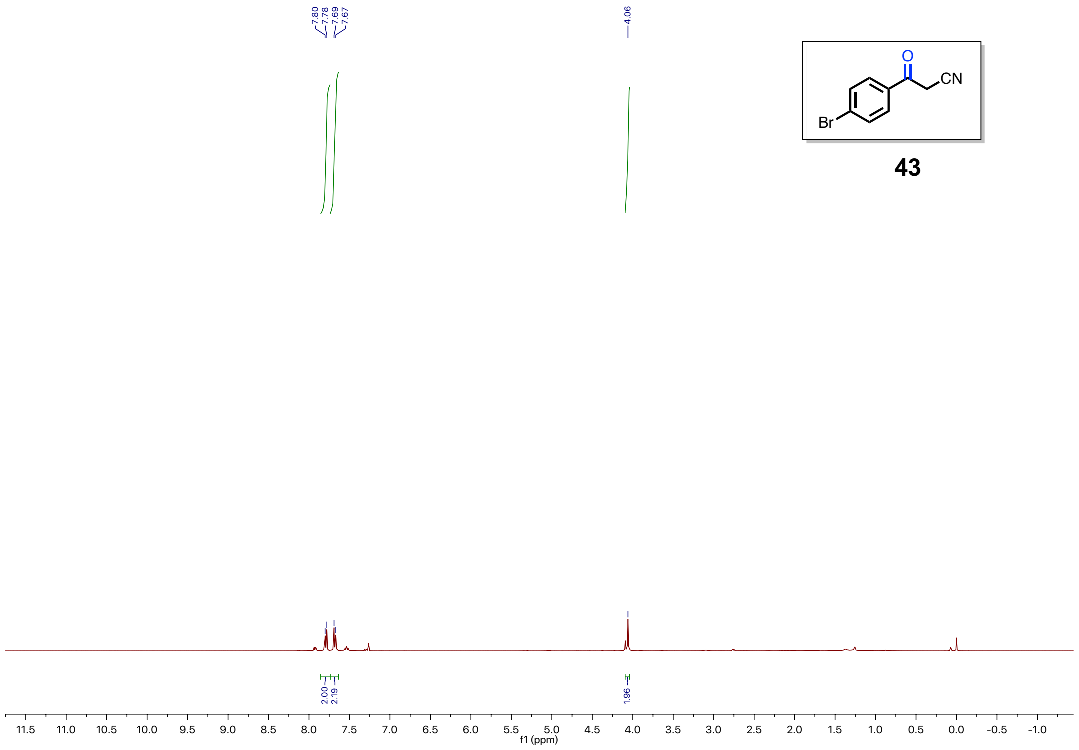

^{13}C NMR spectrum of **43** (101 MHz, $CDCl_3$)

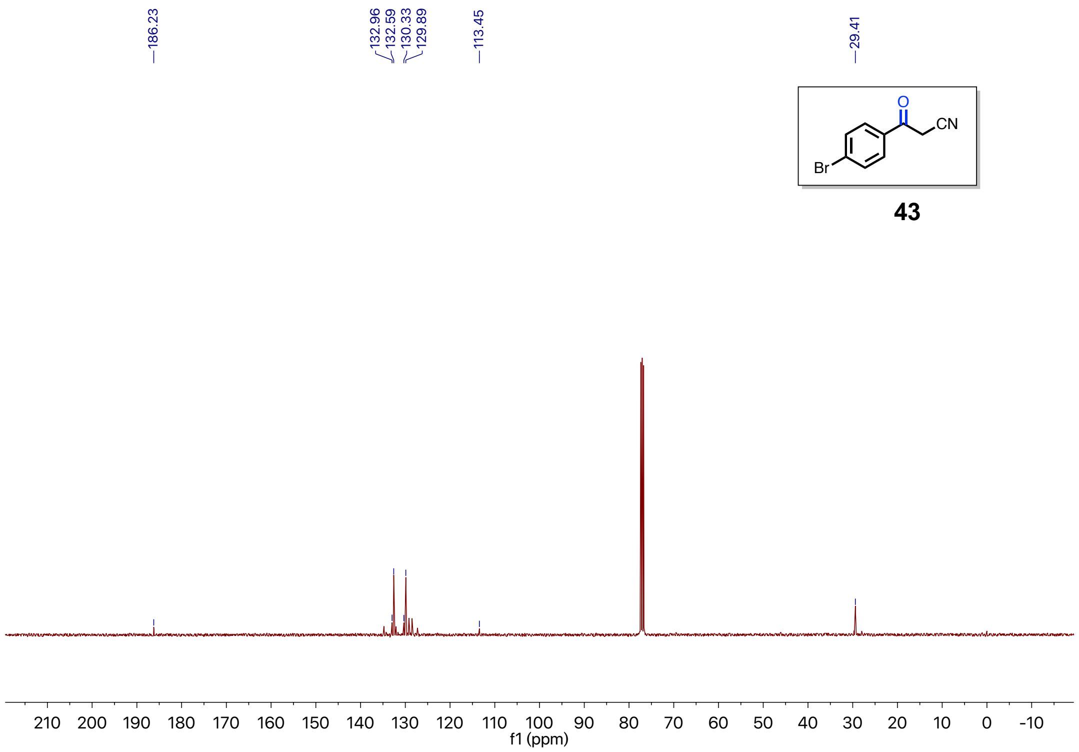

^{1}H NMR spectrum of **44** (400 MHz, $CDCl_3$)

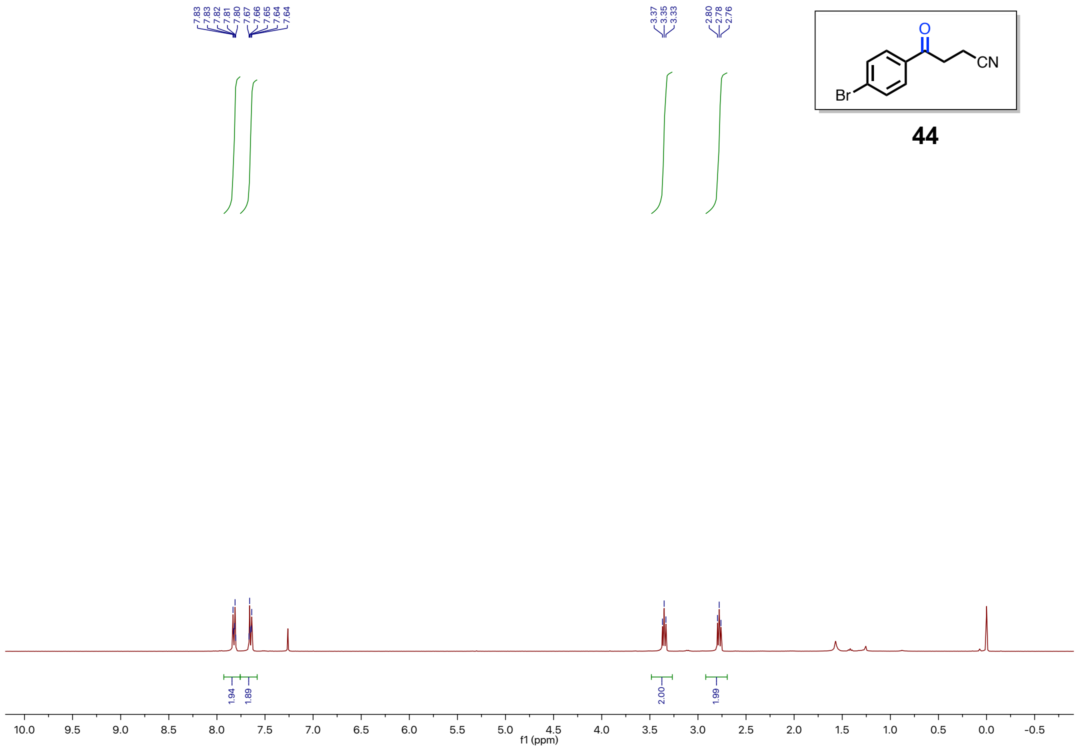

^{13}C NMR spectrum of **44** (126 MHz, $CDCl_3$)

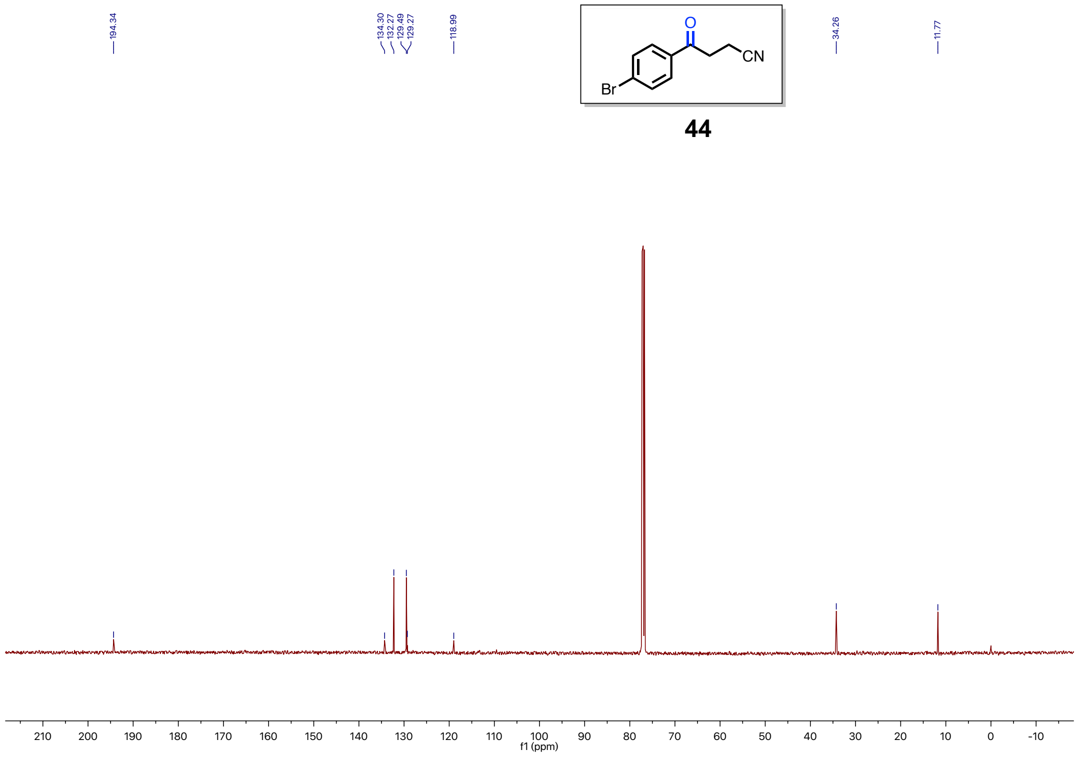

^{1}H NMR spectrum of **45** (400 MHz, $CDCl_3$)

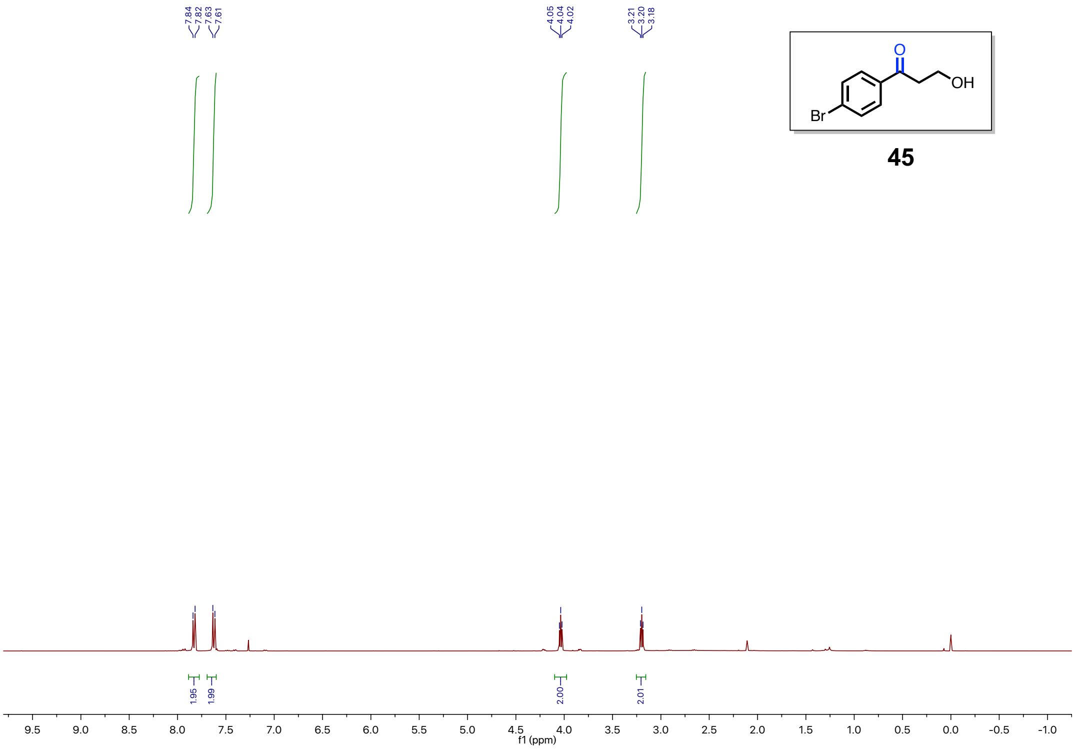

45

^{13}C NMR spectrum of **45** (101 MHz, $CDCl_3$)

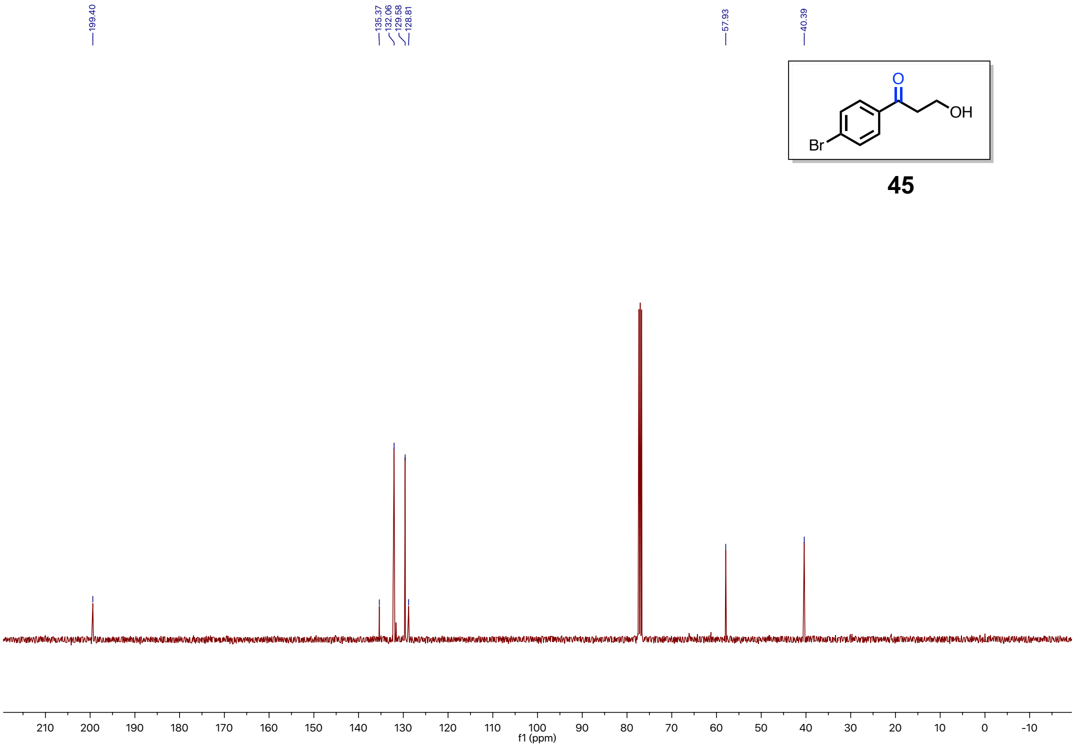

45

^{1}H NMR spectrum of **46** (400 MHz, DMSO-d$_6$)

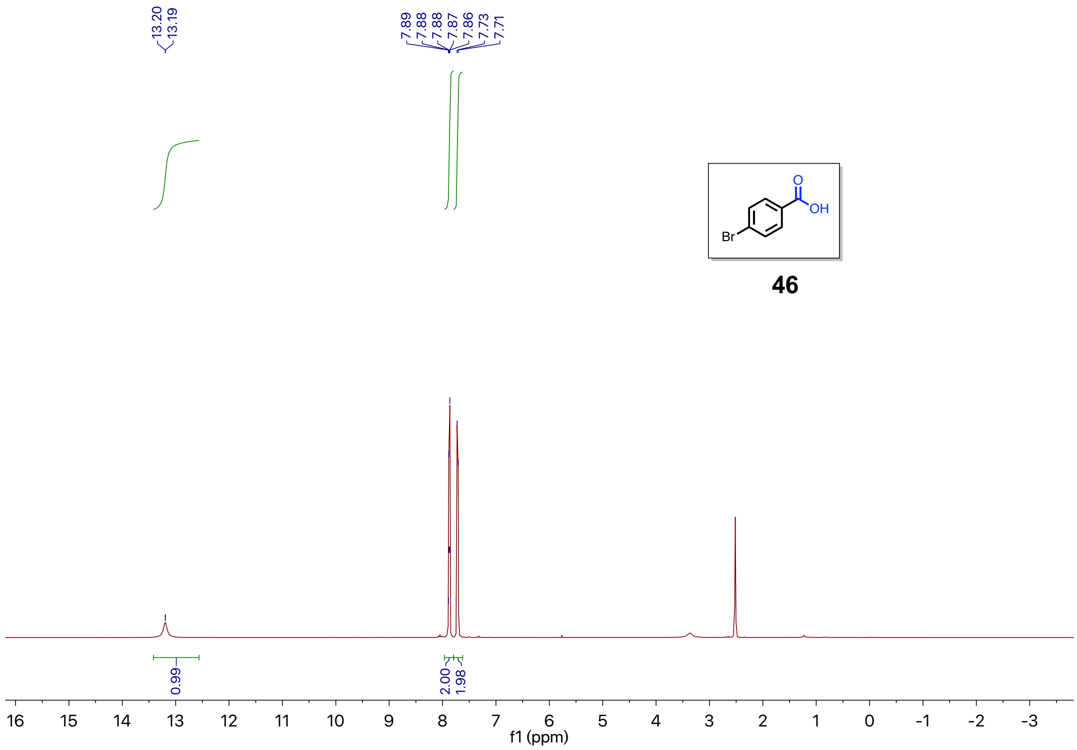

^{13}C NMR spectrum of **46** (101 MHz, CDCl$_3$)

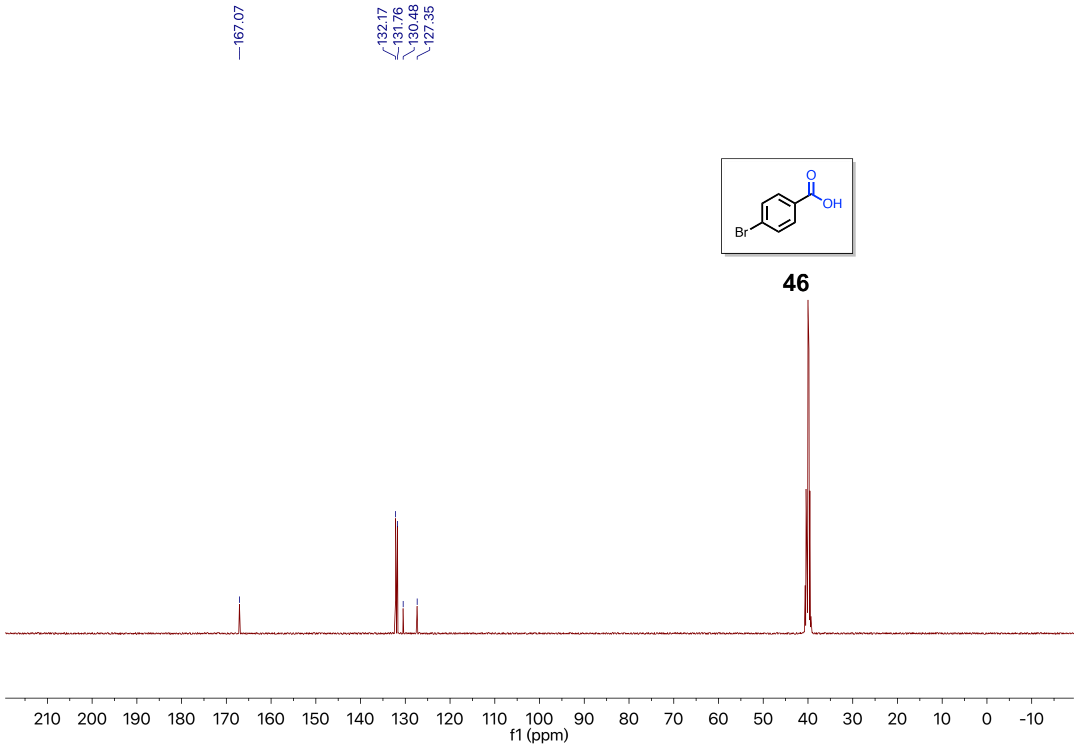

^{1}H NMR spectrum of **47** (600 MHz, $CDCl_3$)

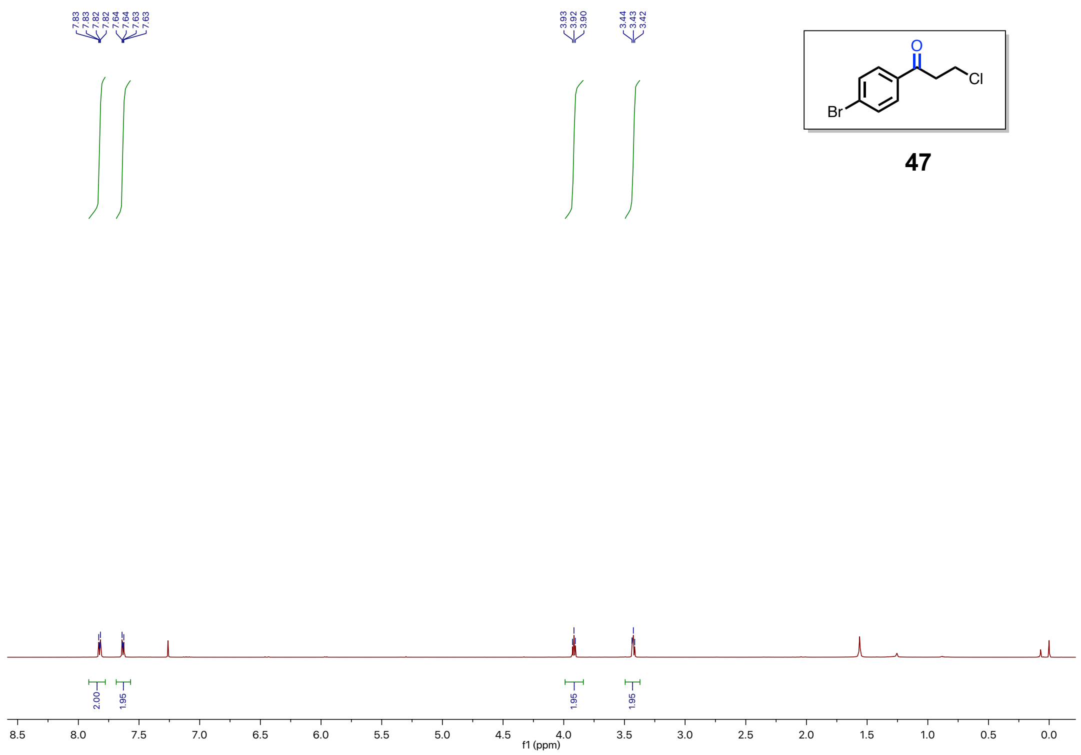

^{13}C NMR spectrum of **47** (101 MHz, $CDCl_3$)

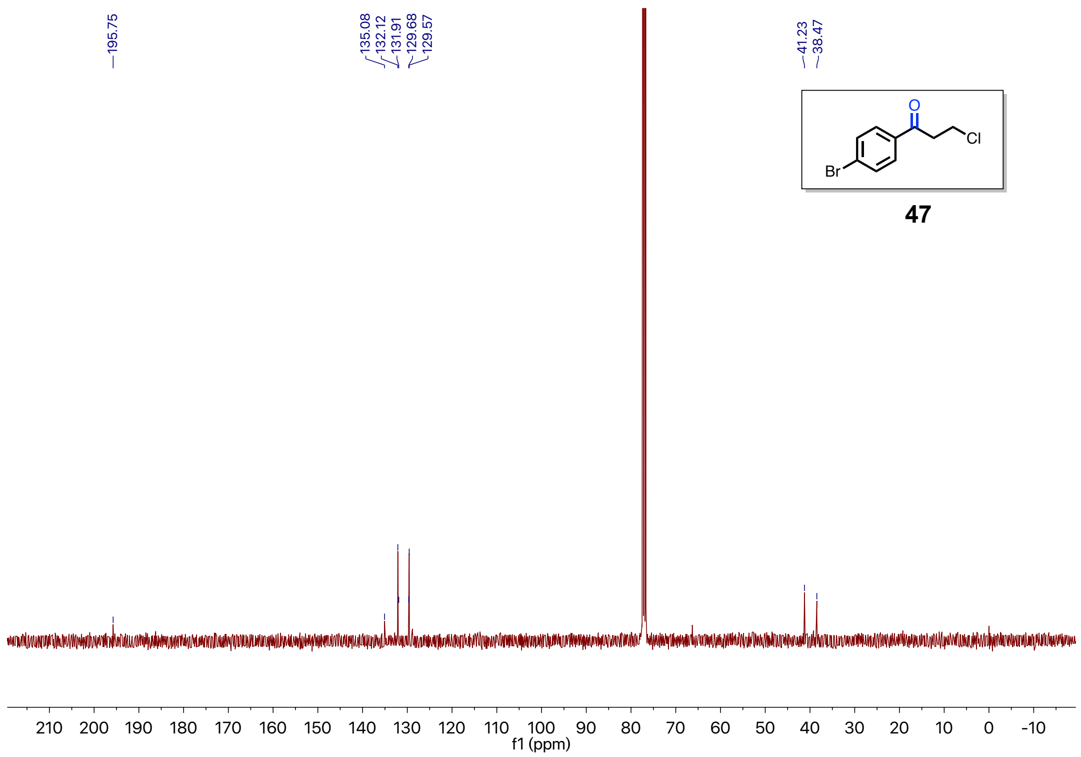

^{1}H NMR spectrum of **48** (600 MHz, $CDCl_3$)

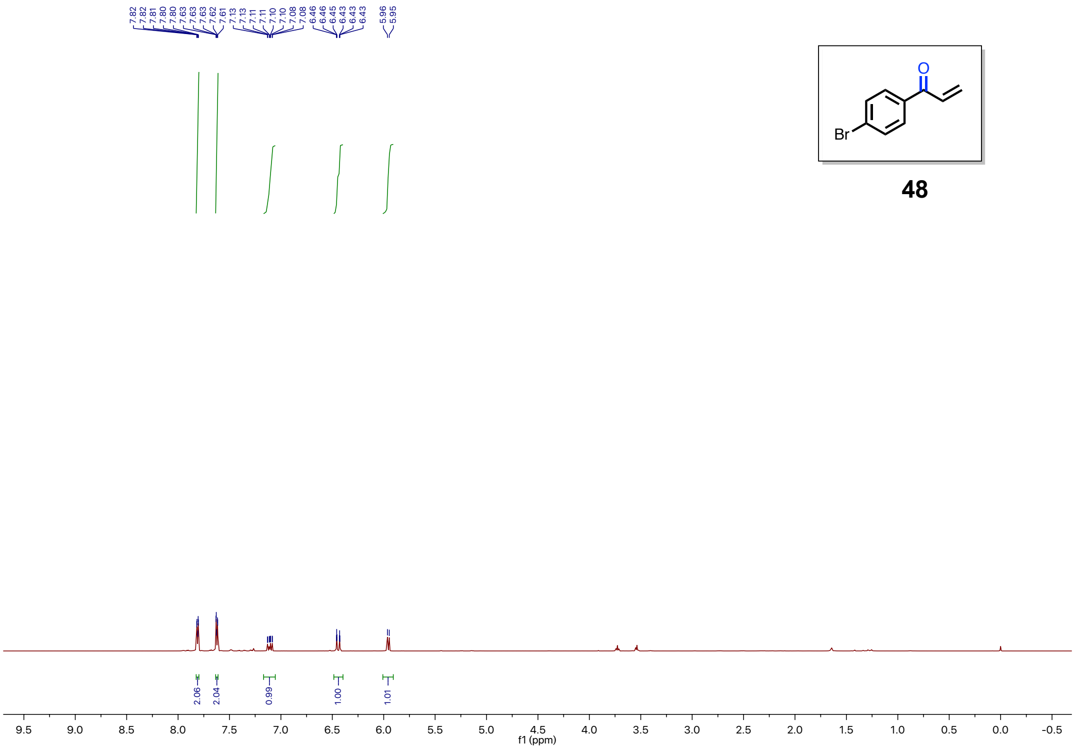

48

^{13}C NMR spectrum of **48** (101 MHz, $CDCl_3$)

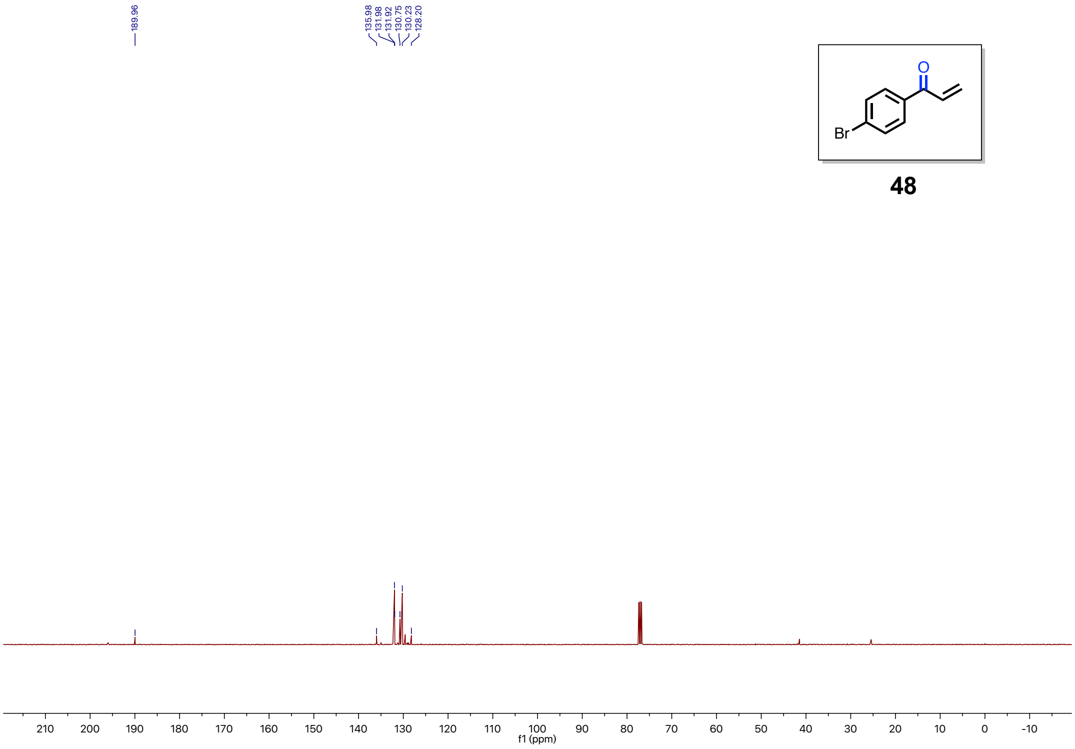

48

^{1}H NMR spectrum of **50** (400 MHz, $CDCl_3$)

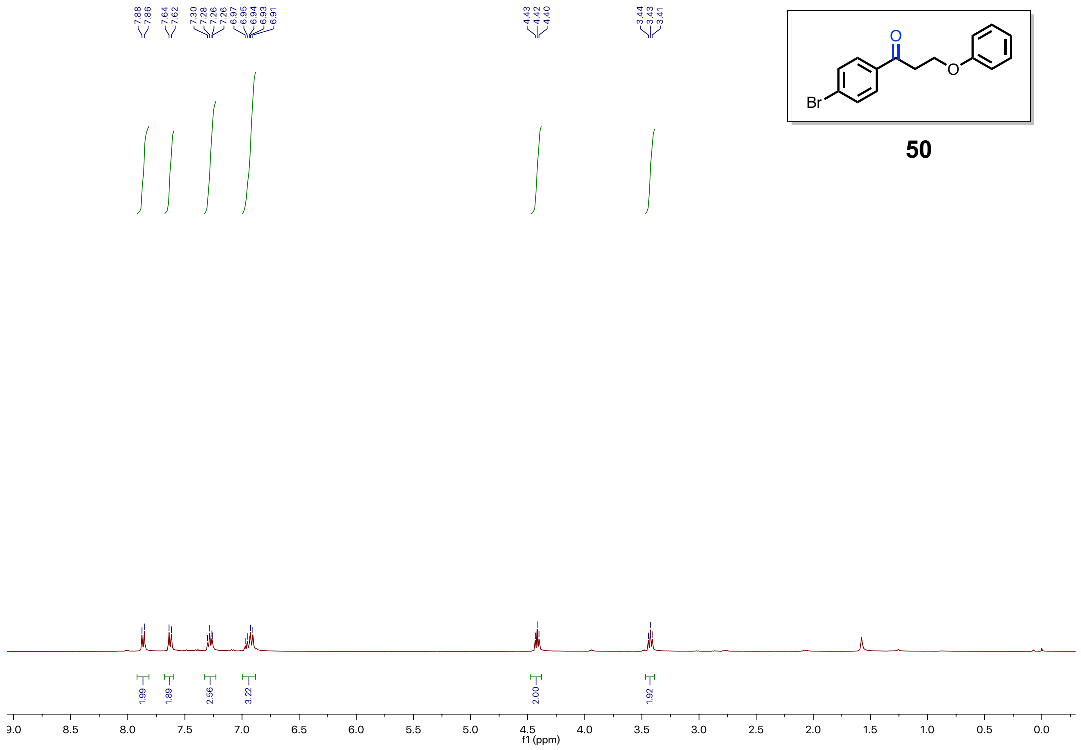

^{13}C NMR spectrum of **50** (101 MHz, $CDCl_3$)

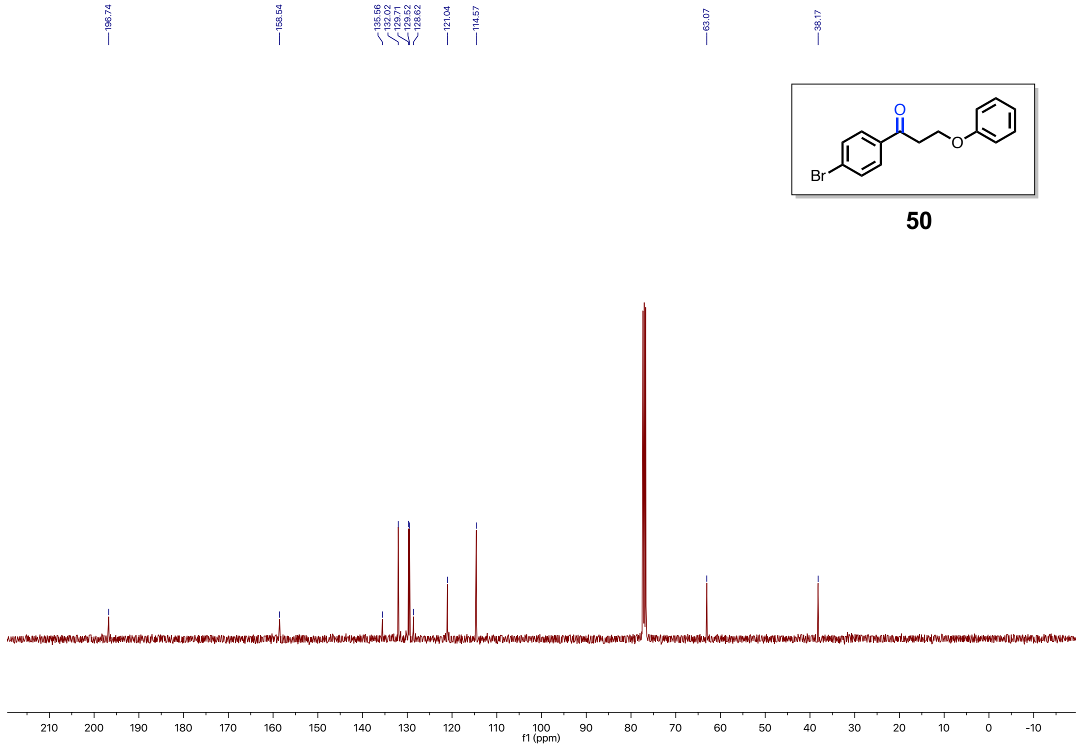

^{1}H NMR spectrum of **51** (500 MHz, $CDCl_3$)

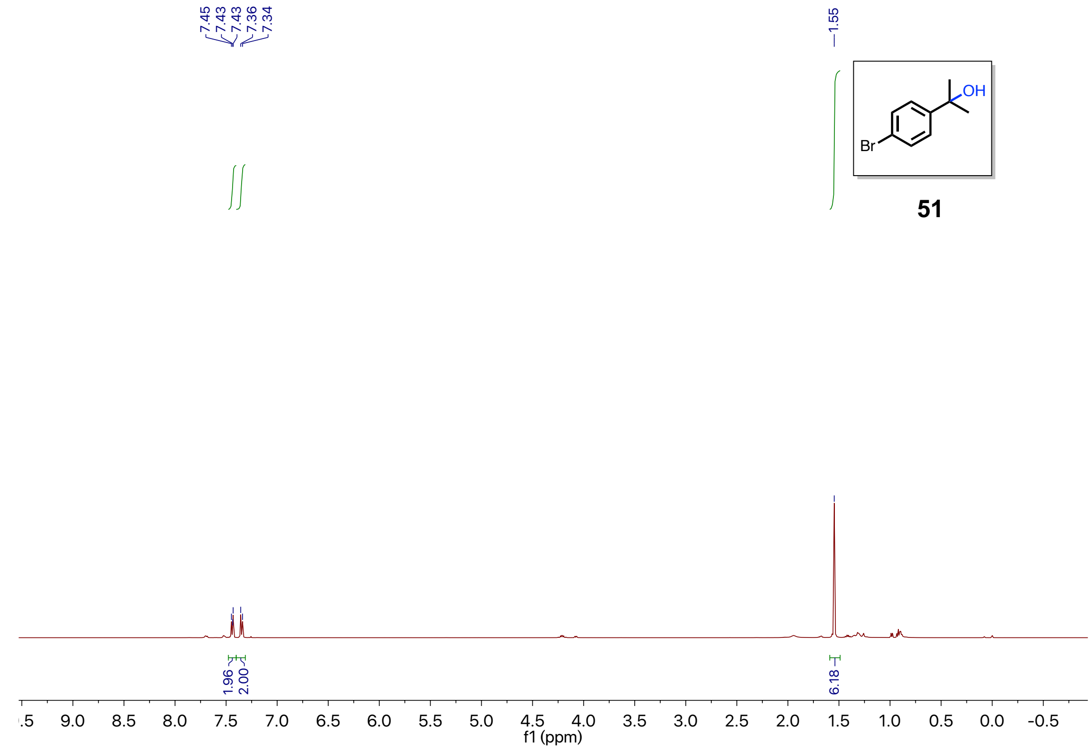

^{13}C NMR spectrum of **51** (126 MHz, $CDCl_3$)

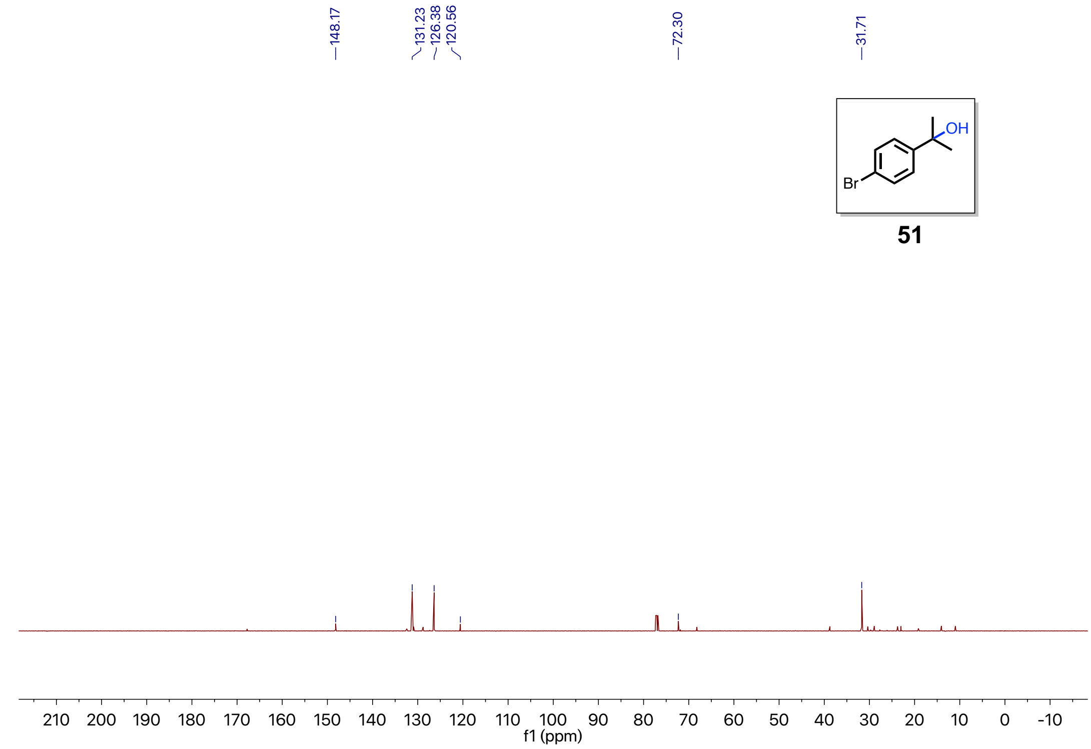

^{1}H NMR spectrum of **51b** (500 MHz, $CDCl_3$)

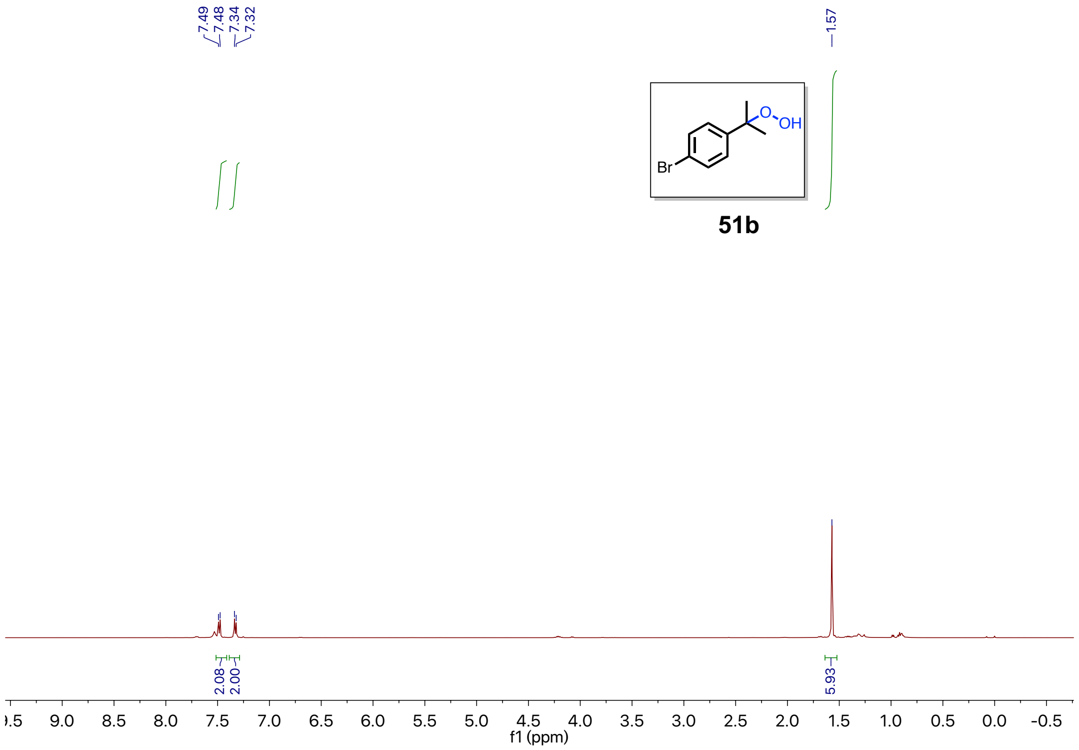

^{13}C NMR spectrum of **51b** (126 MHz, $CDCl_3$)

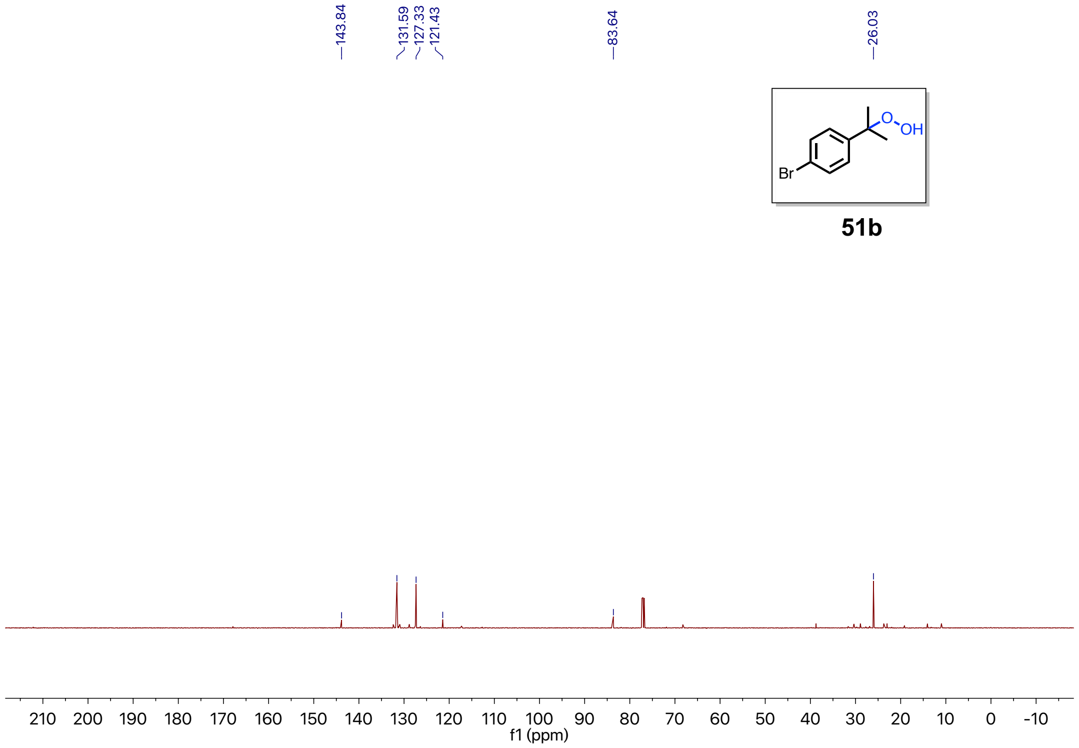

^{1}H NMR spectrum of **52** (400 MHz, $CDCl_3$)

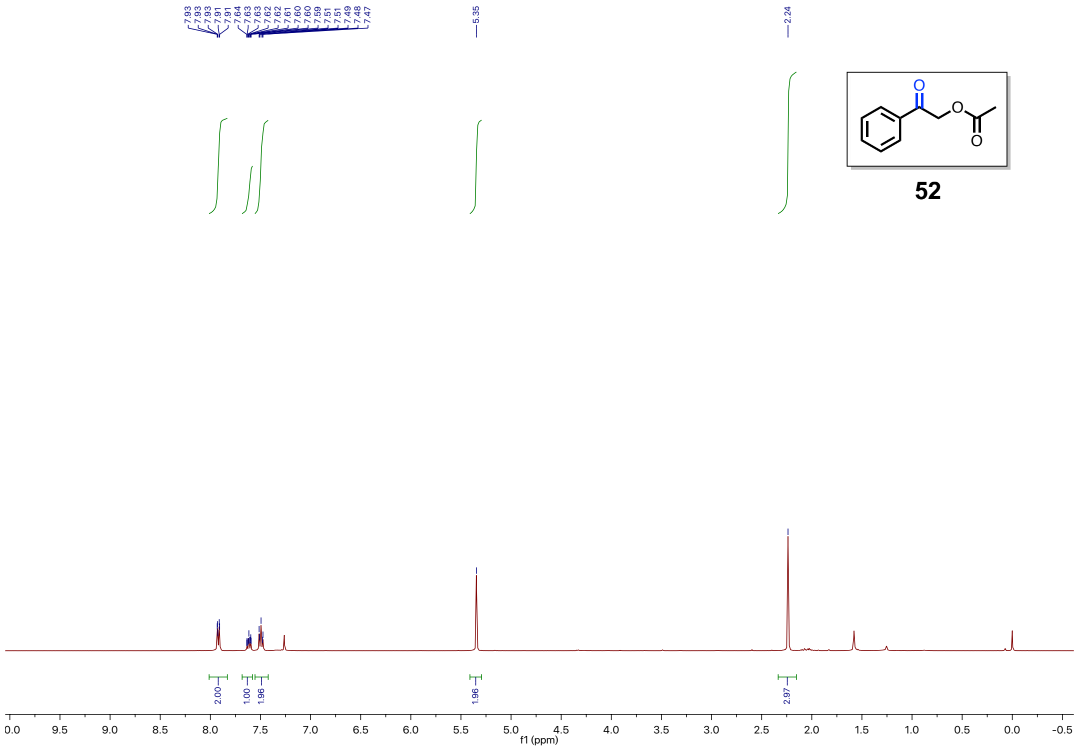

^{13}C NMR spectrum of **52** (126 MHz, $CDCl_3$)

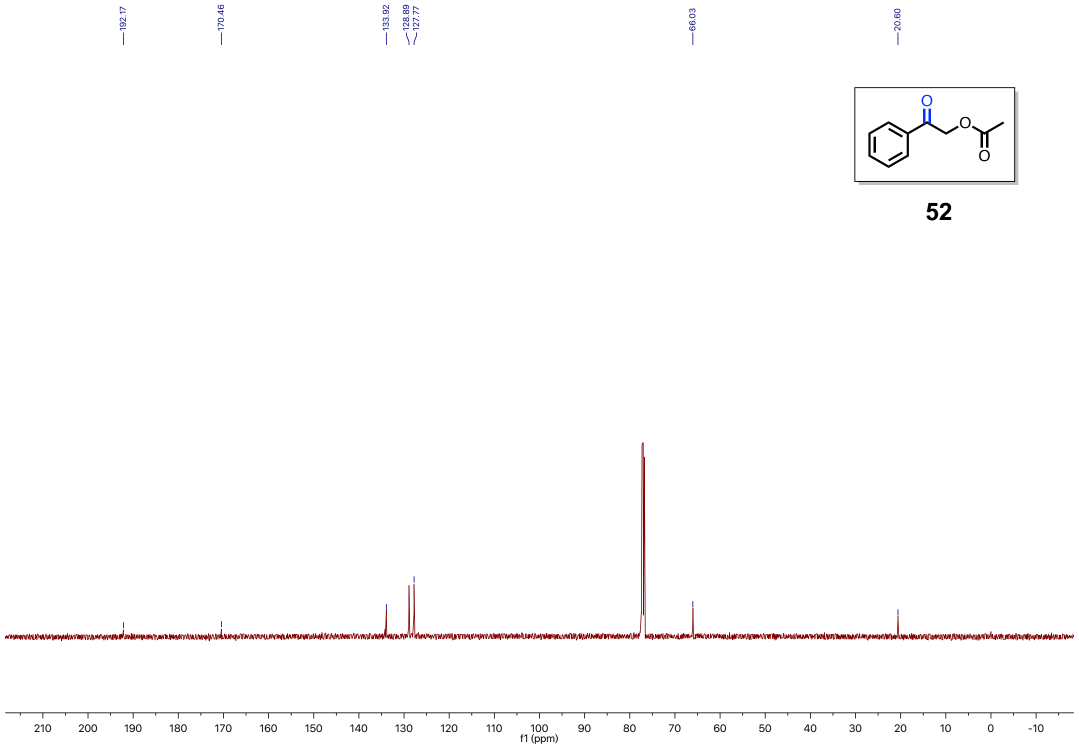

^{1}H NMR spectrum of **53** (400 MHz, $CDCl_3$)

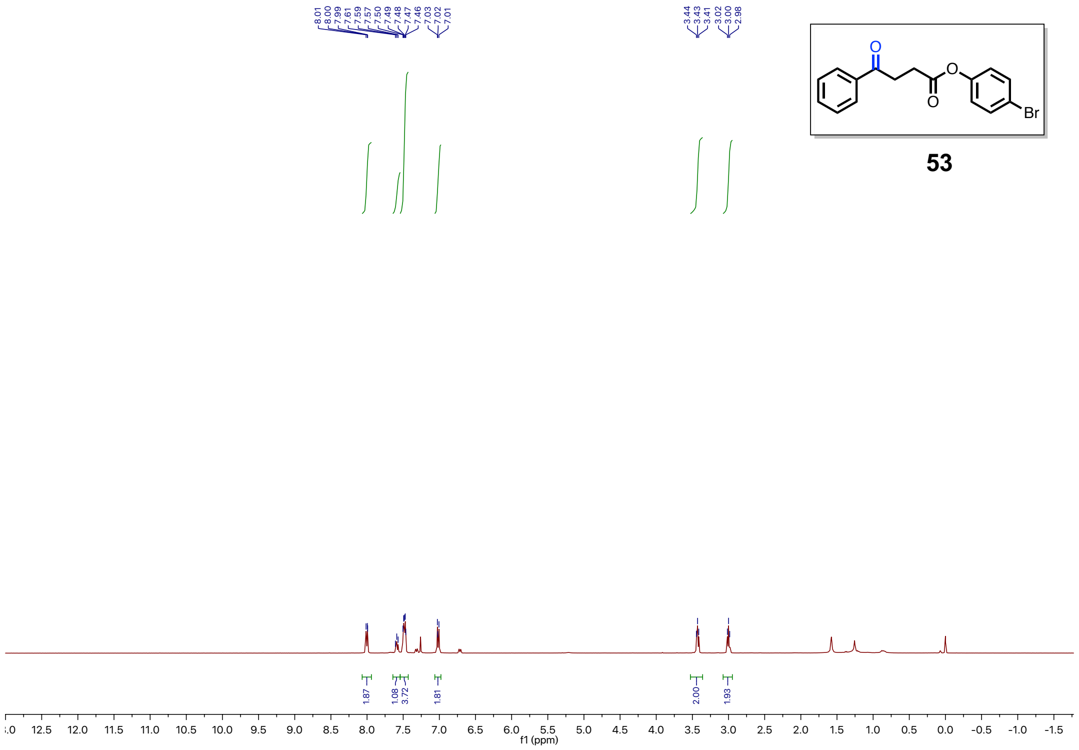

^{13}C NMR spectrum of **53** (126 MHz, $CDCl_3$)

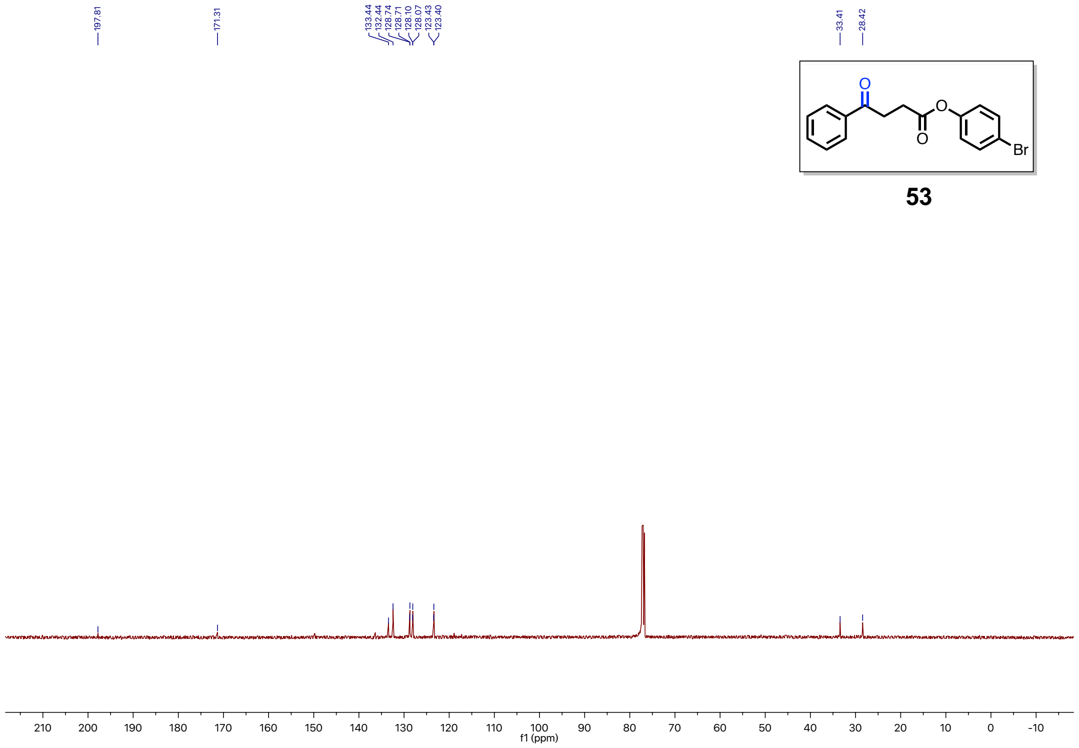

^1H NMR spectrum of **54** (400 MHz, $CDCl_3$)

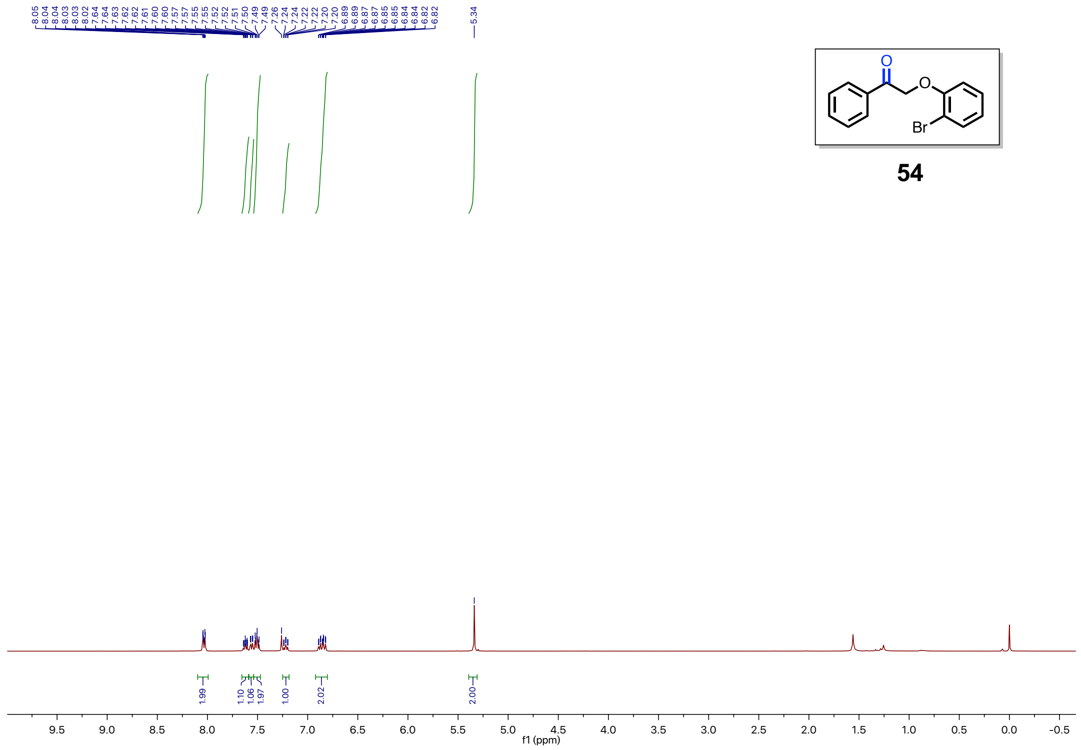

54

^{13}C NMR spectrum of **54** (126 MHz, $CDCl_3$)

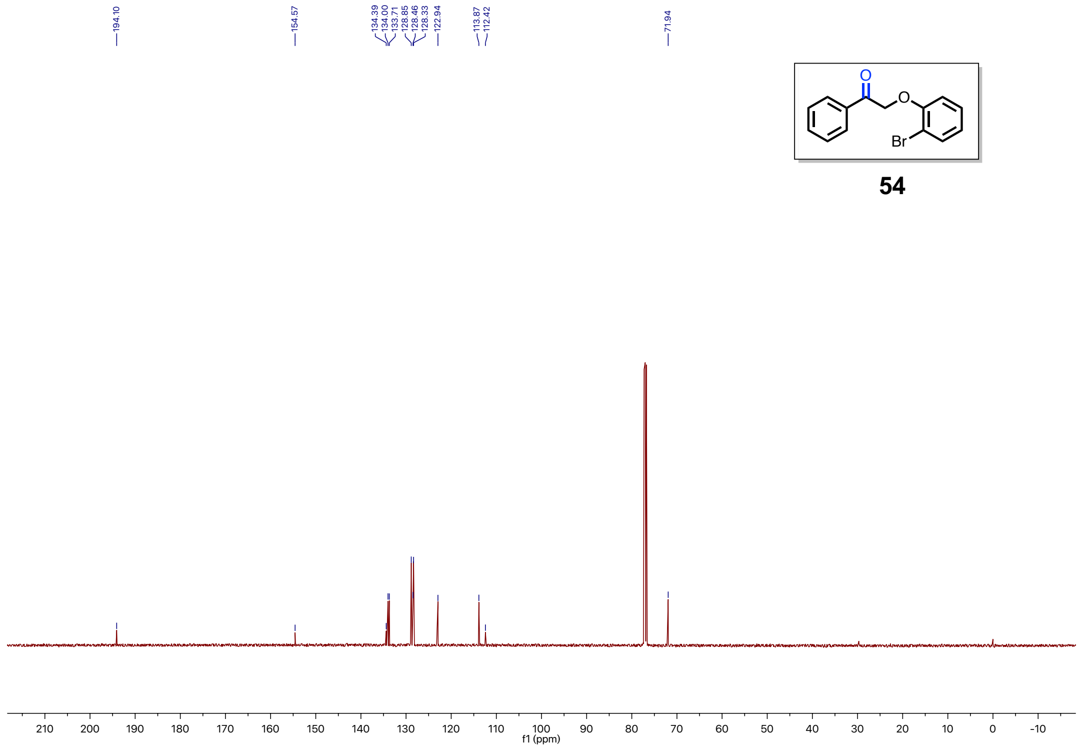

54

^{1}H NMR spectrum of **55** (400 MHz, $CDCl_3$)

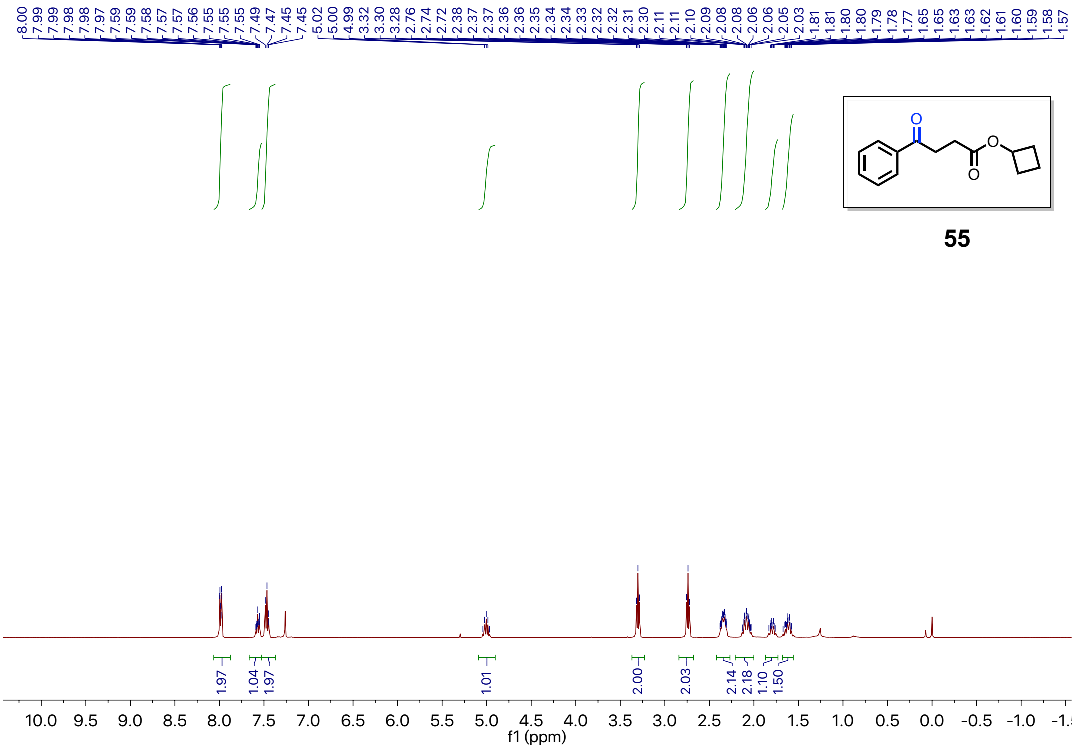

55

^{13}C NMR spectrum of **55** (126 MHz, $CDCl_3$)

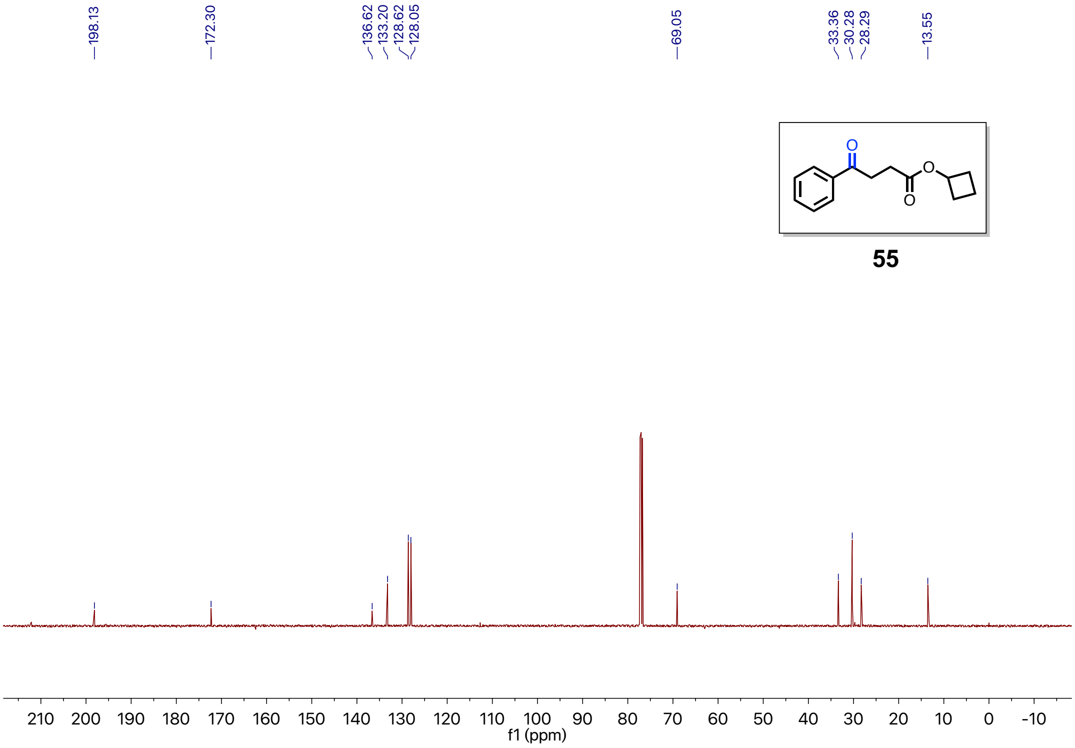

55

^{1}H NMR spectrum of **56** (400 MHz, $CDCl_3$)

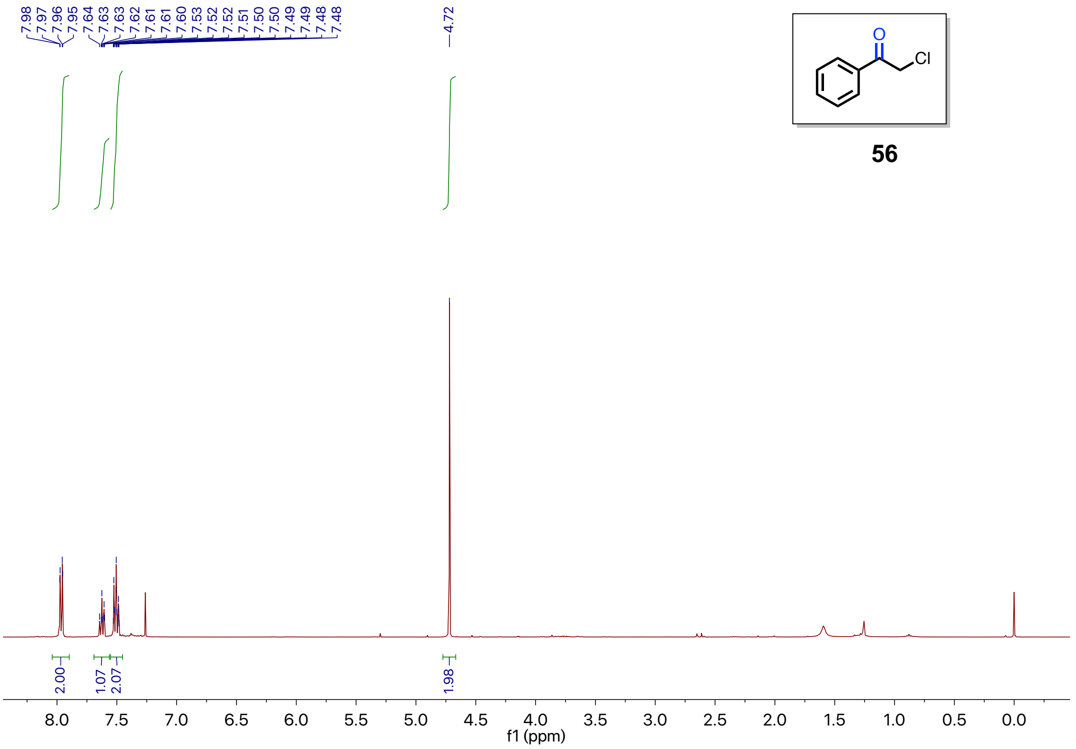

^{13}C NMR spectrum of **56** (101 MHz, $CDCl_3$)

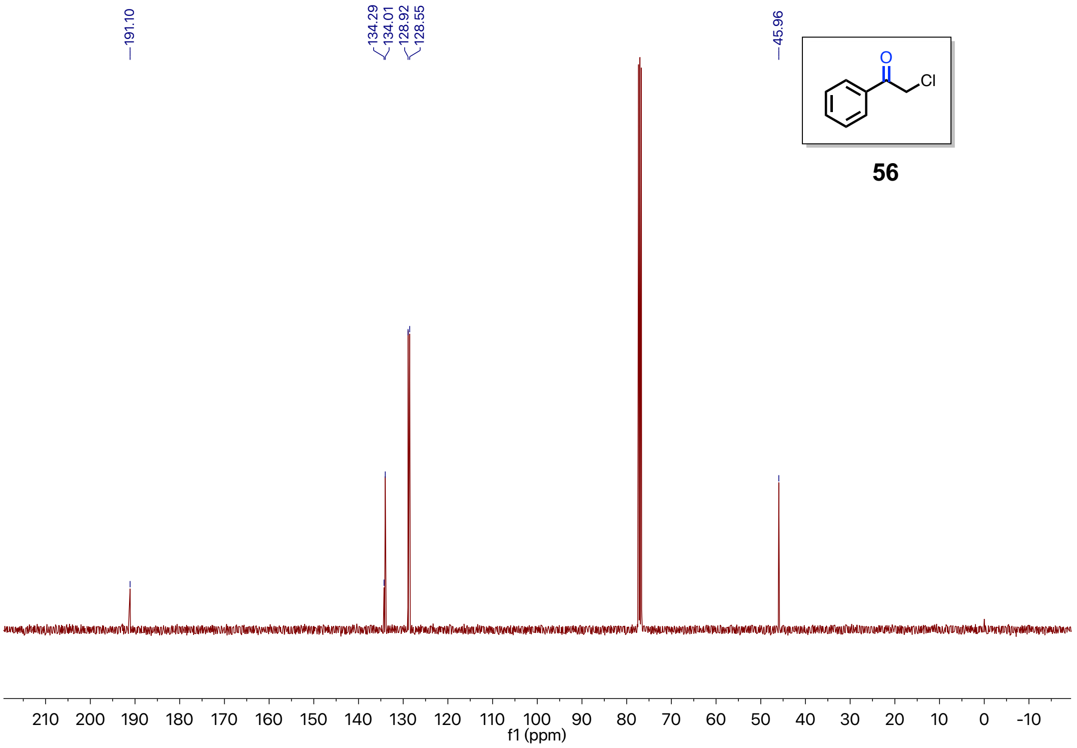

^{1}H NMR spectrum of **57** (400 MHz, $CDCl_3$)

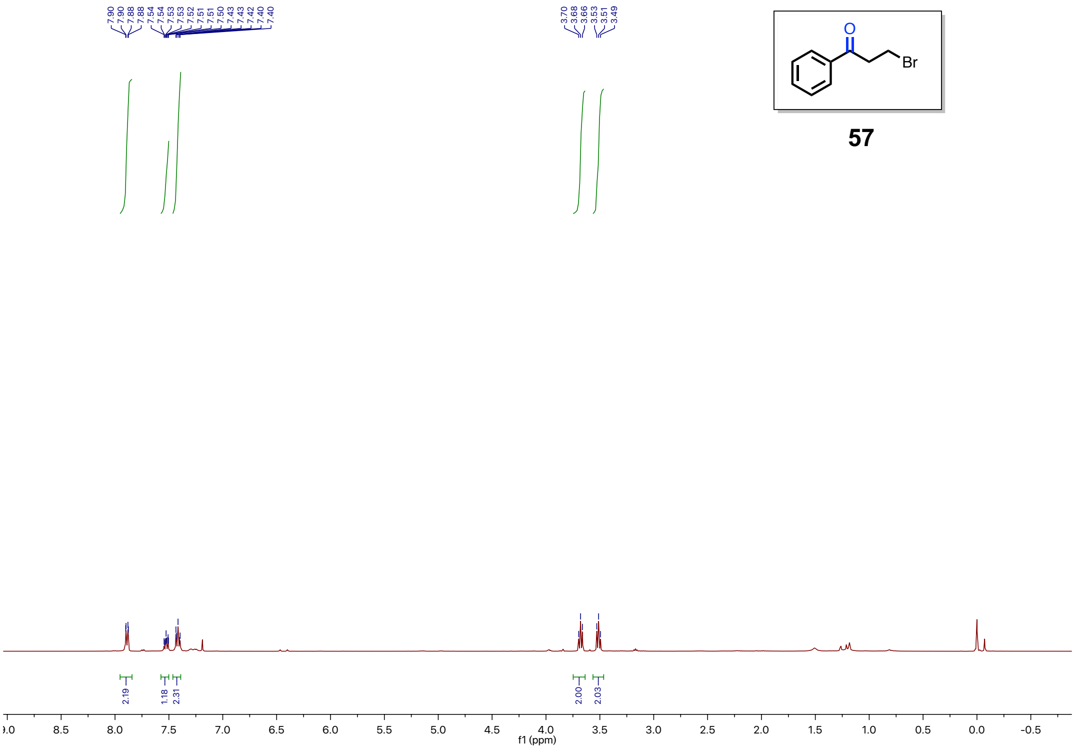

57

^{13}C NMR spectrum of **57** (101 MHz, $CDCl_3$)

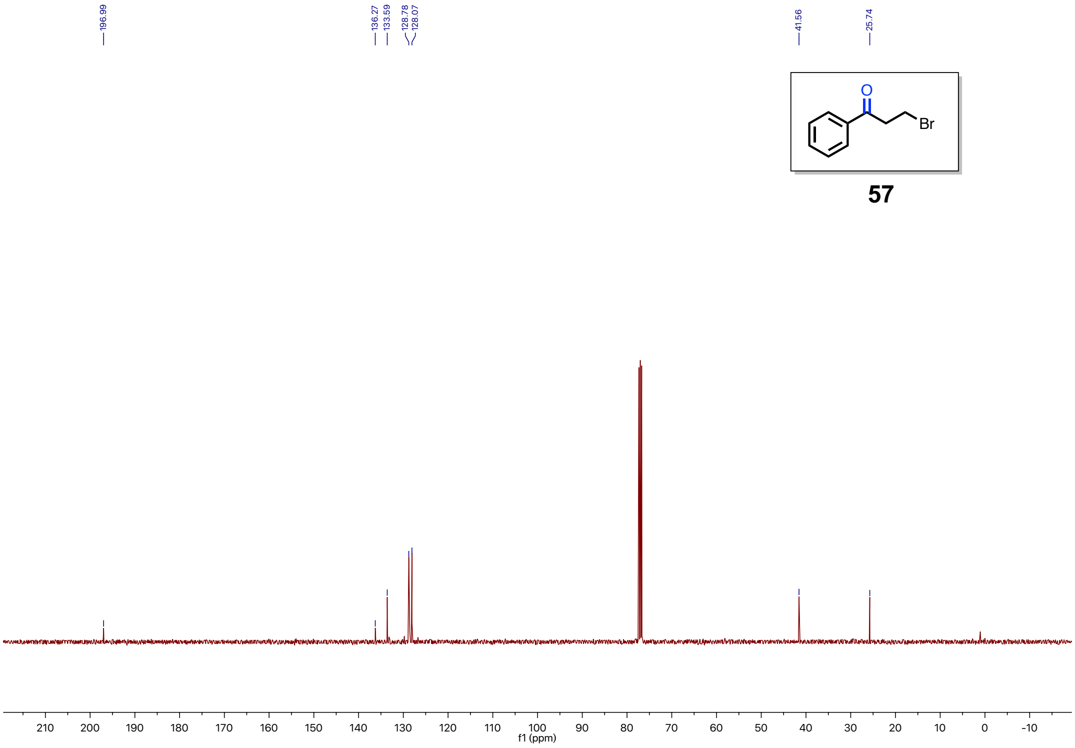

57

^{1}H NMR spectrum of **58** (400 MHz, $CDCl_3$)

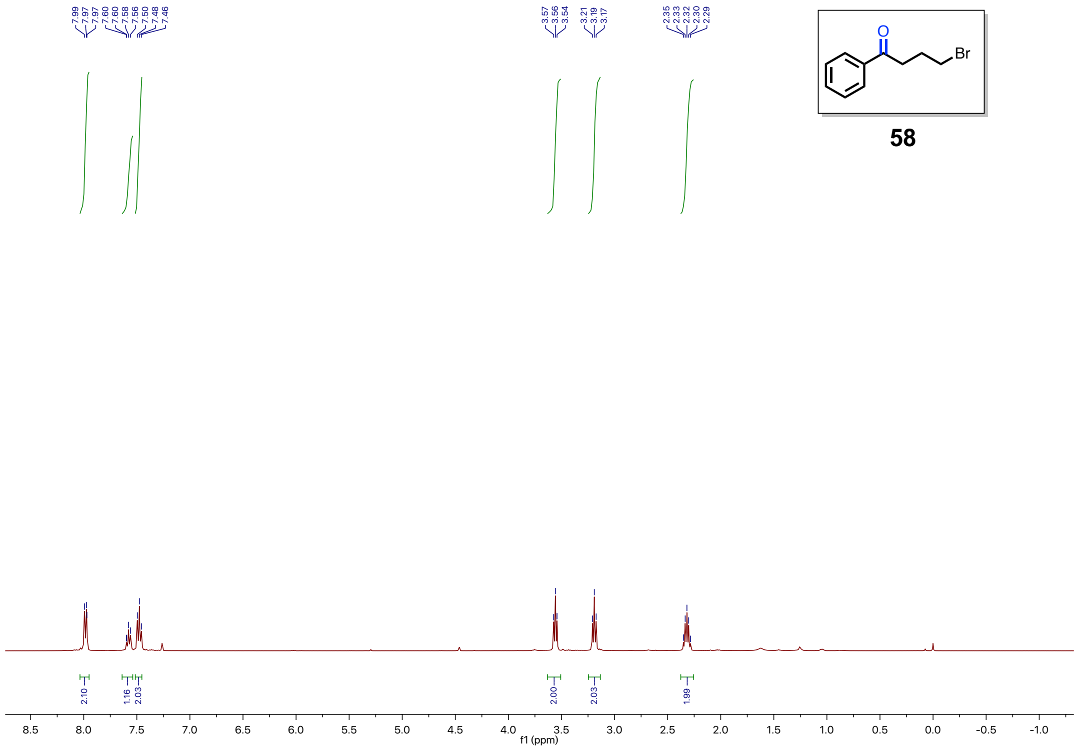

^{13}C NMR spectrum of **58** (126 MHz, $CDCl_3$)

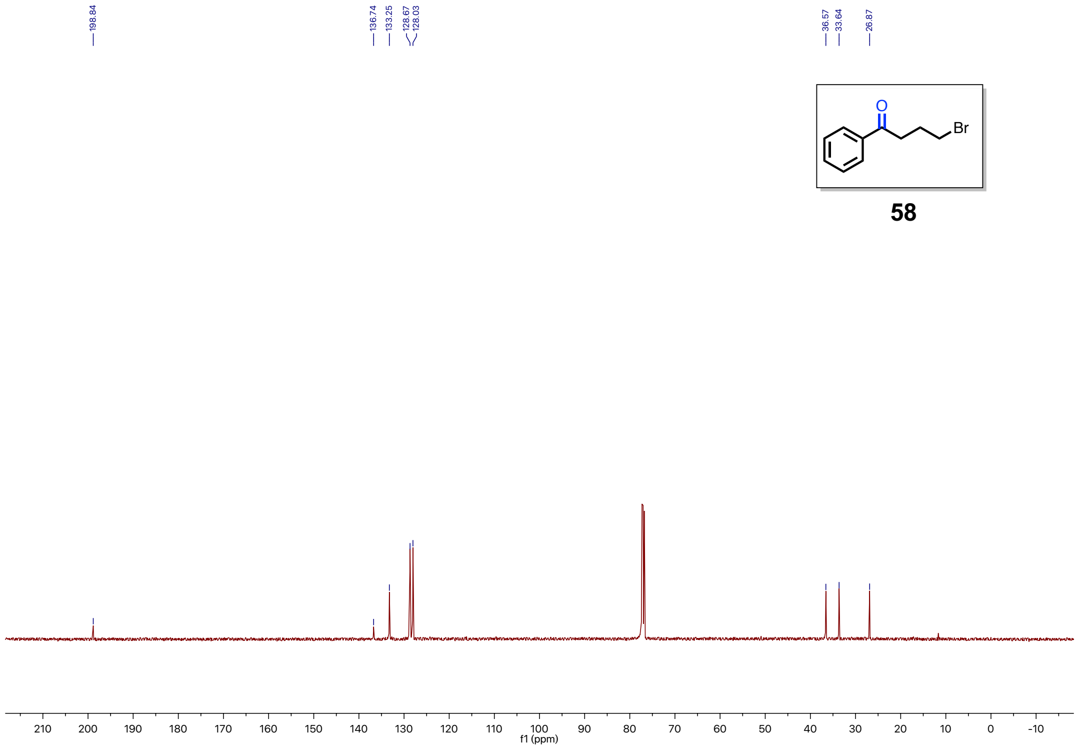

^{1}H NMR spectrum of **59** (400 MHz, $CDCl_3$)

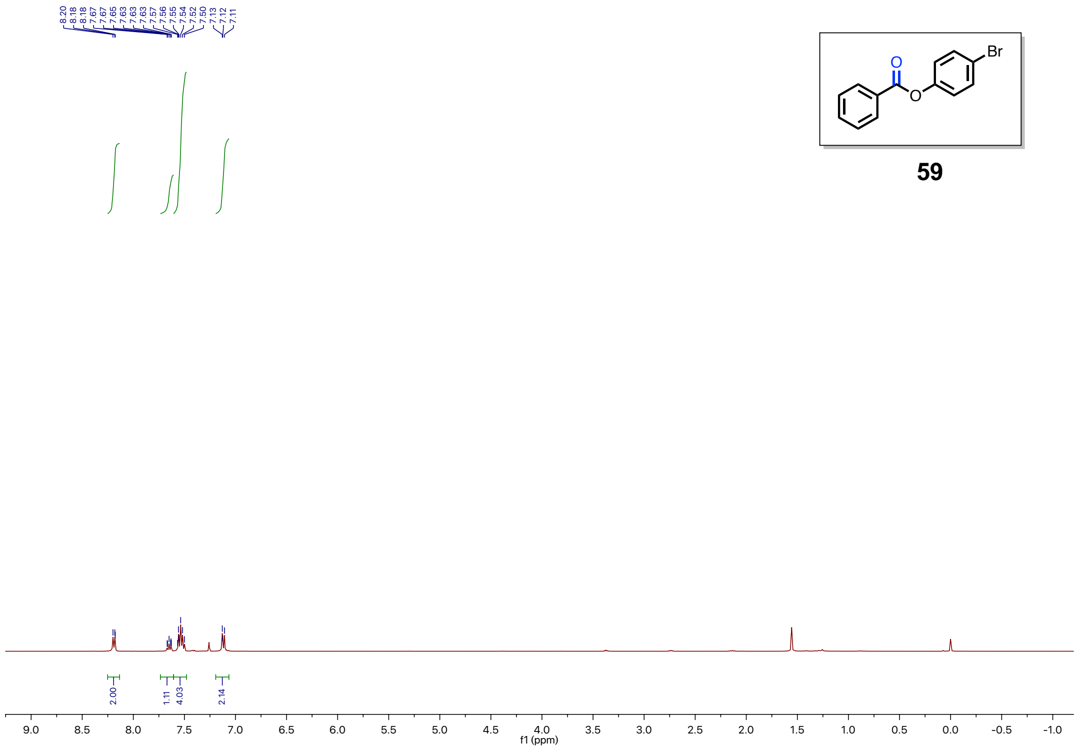

59

^{13}C NMR spectrum of **59** (101 MHz, $CDCl_3$)

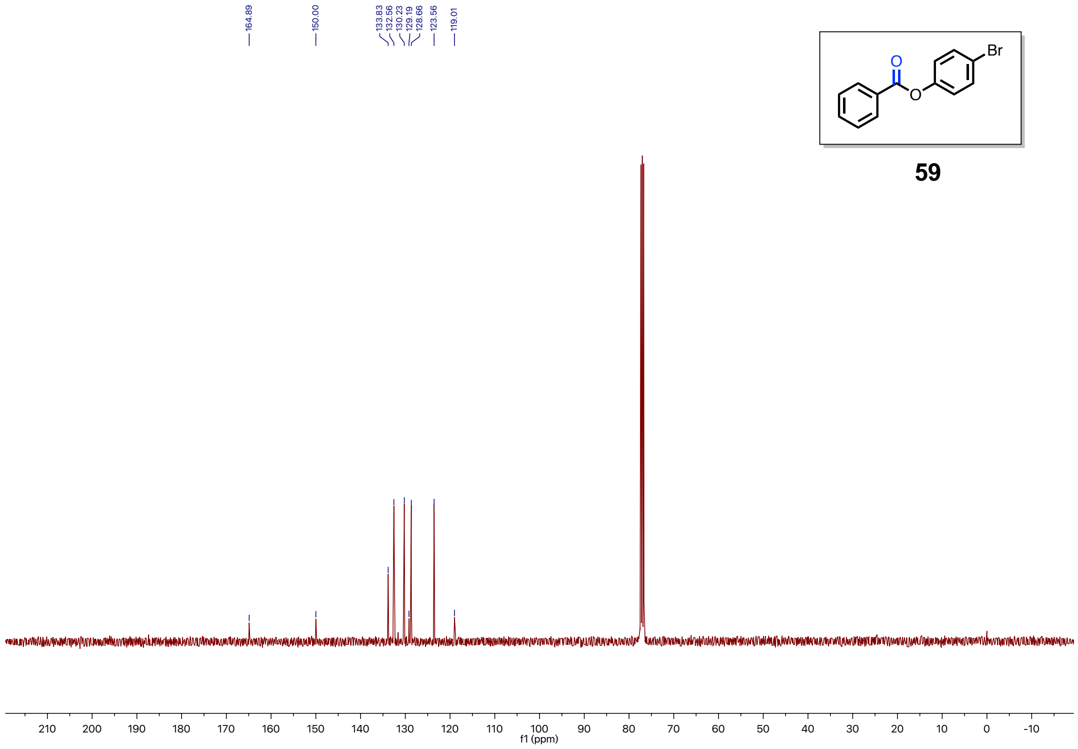

59

^{1}H NMR spectrum of **60** (400 MHz, $CDCl_3$)

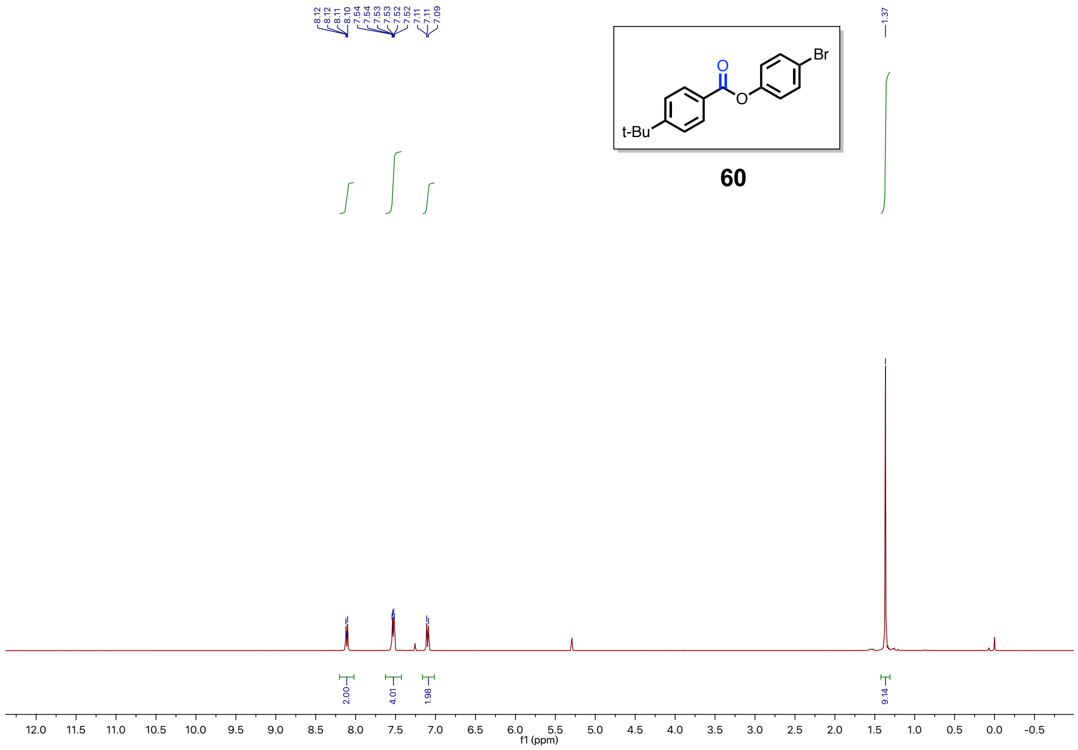

^{13}C NMR spectrum of **60** (126 MHz, $CDCl_3$)

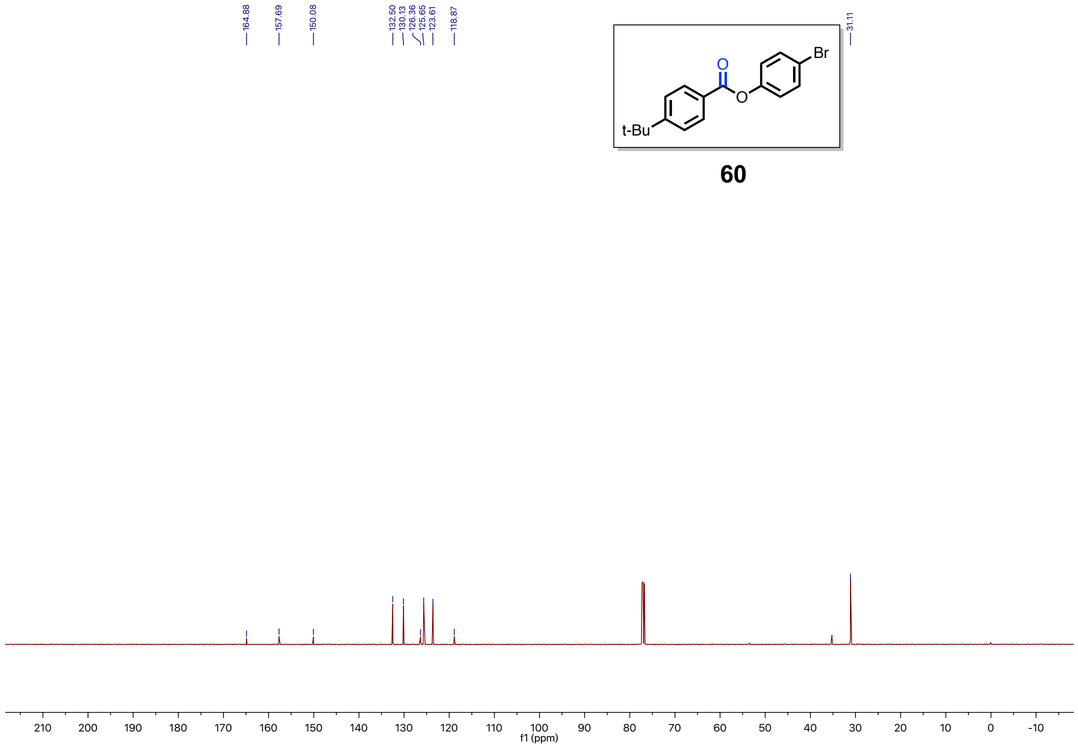

^{1}H NMR spectrum of **61** (400 MHz, $CDCl_3$)

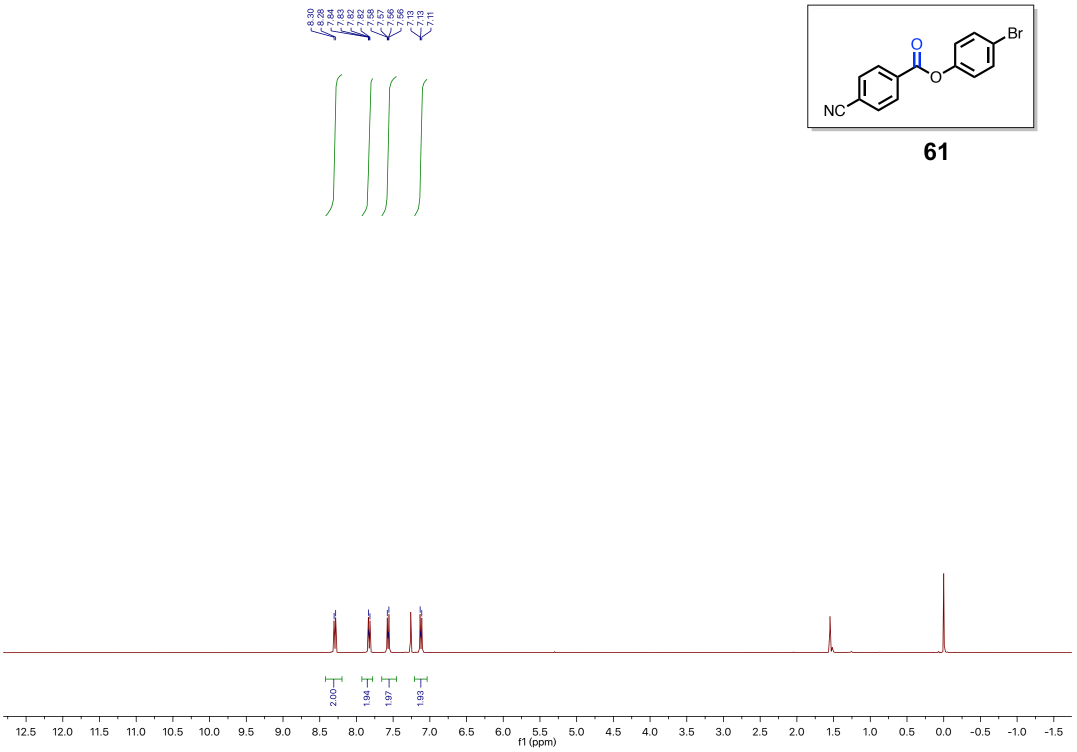

^{13}C NMR spectrum of **61** (126 MHz, $CDCl_3$)

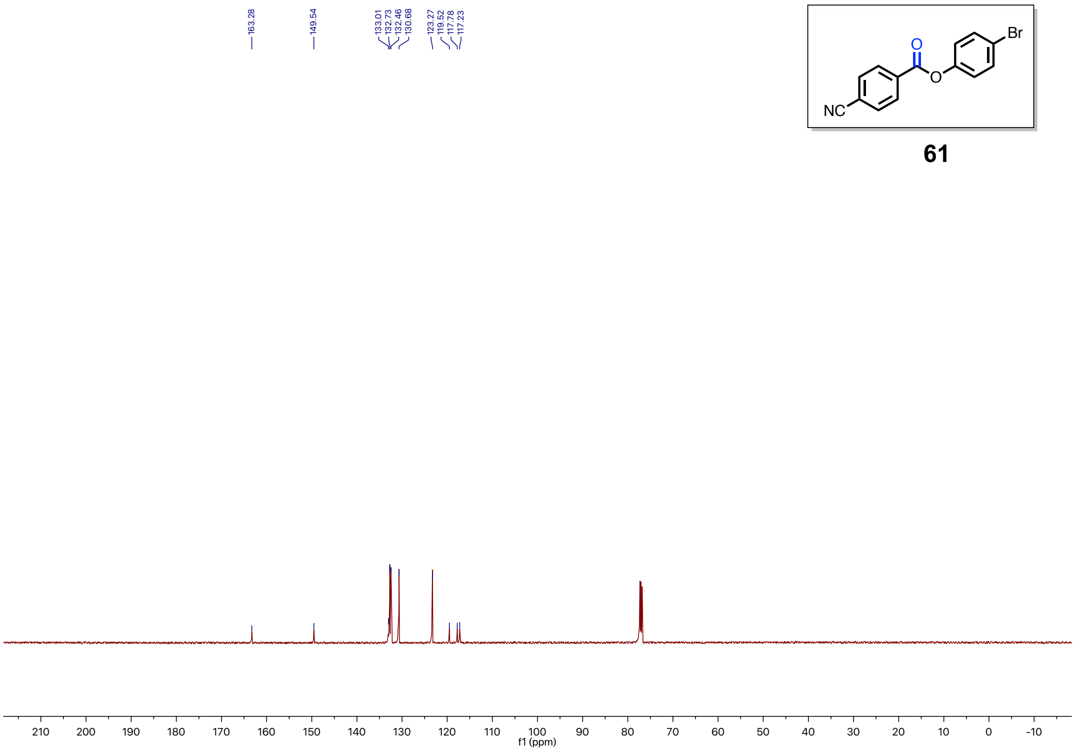

^{1}H NMR spectrum of **62** (400 MHz, $CDCl_3$)

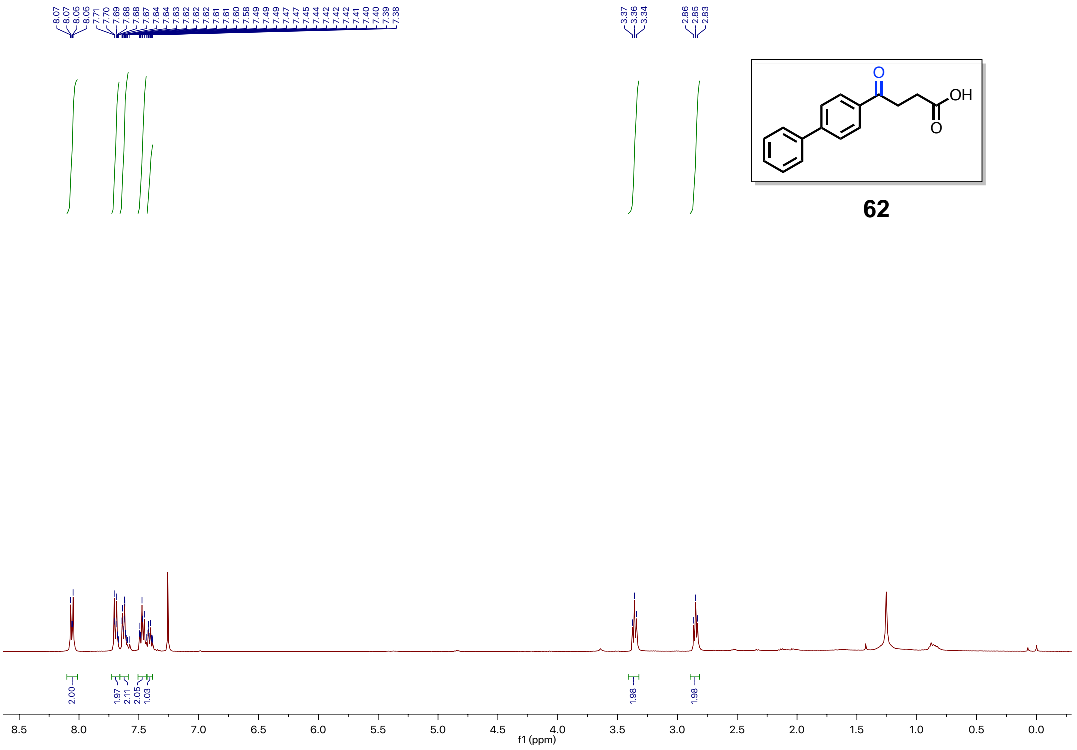

^{13}C NMR spectrum of **62** (101 MHz, $CDCl_3$)

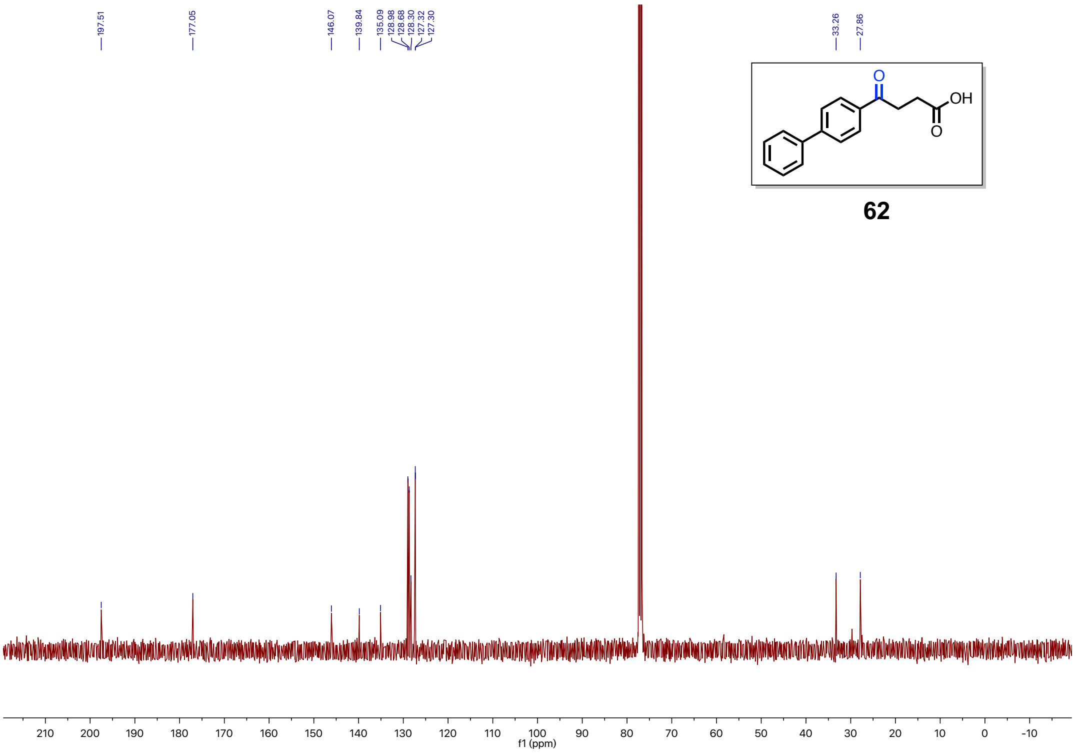

^{1}H NMR spectrum of **63** (400 MHz, $CDCl_3$)

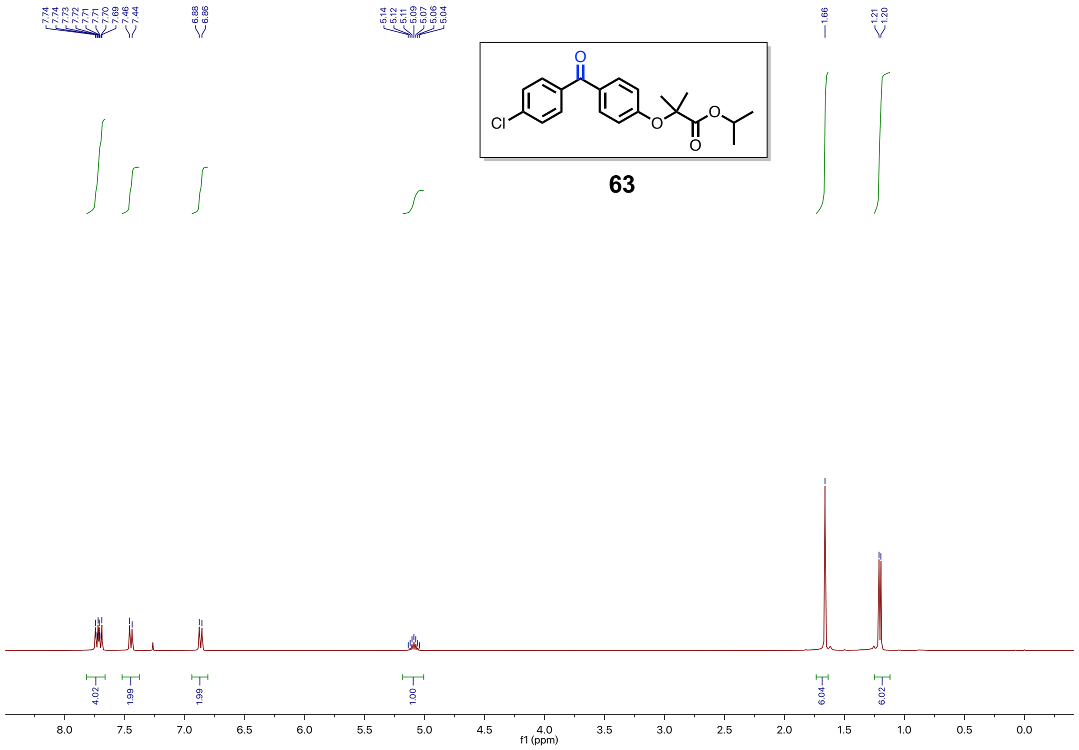

^{13}C NMR spectrum of **63** (101 MHz, $CDCl_3$)

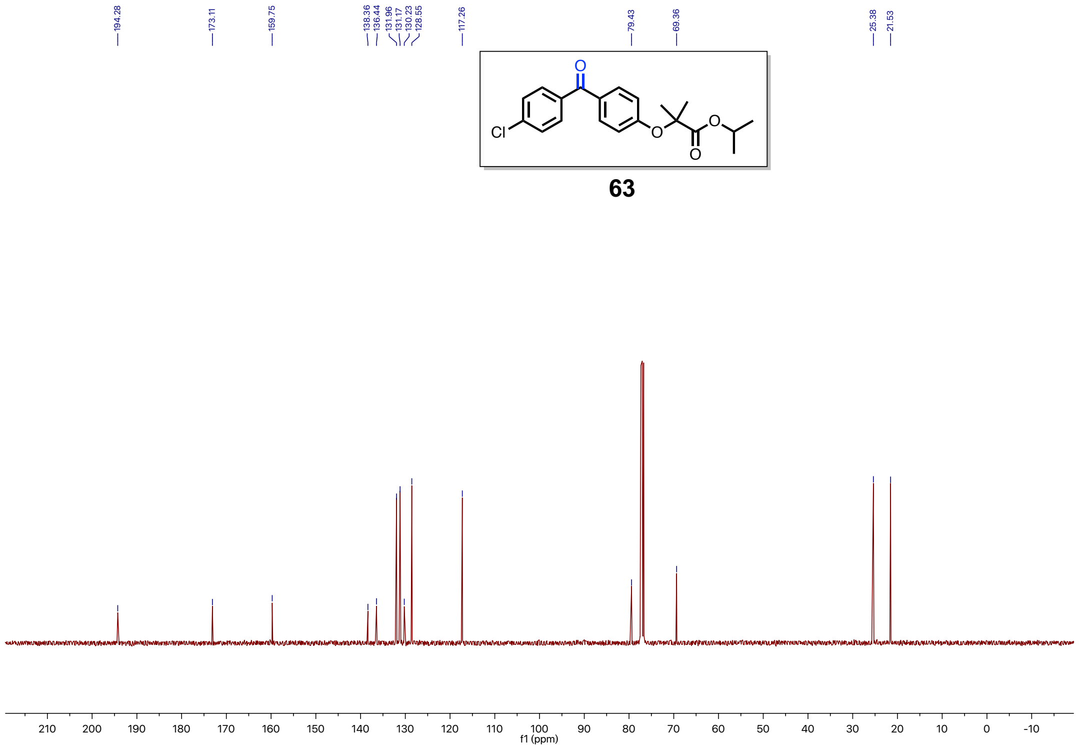

^{1}H NMR spectrum of **64** (500 MHz, $CDCl_3$)

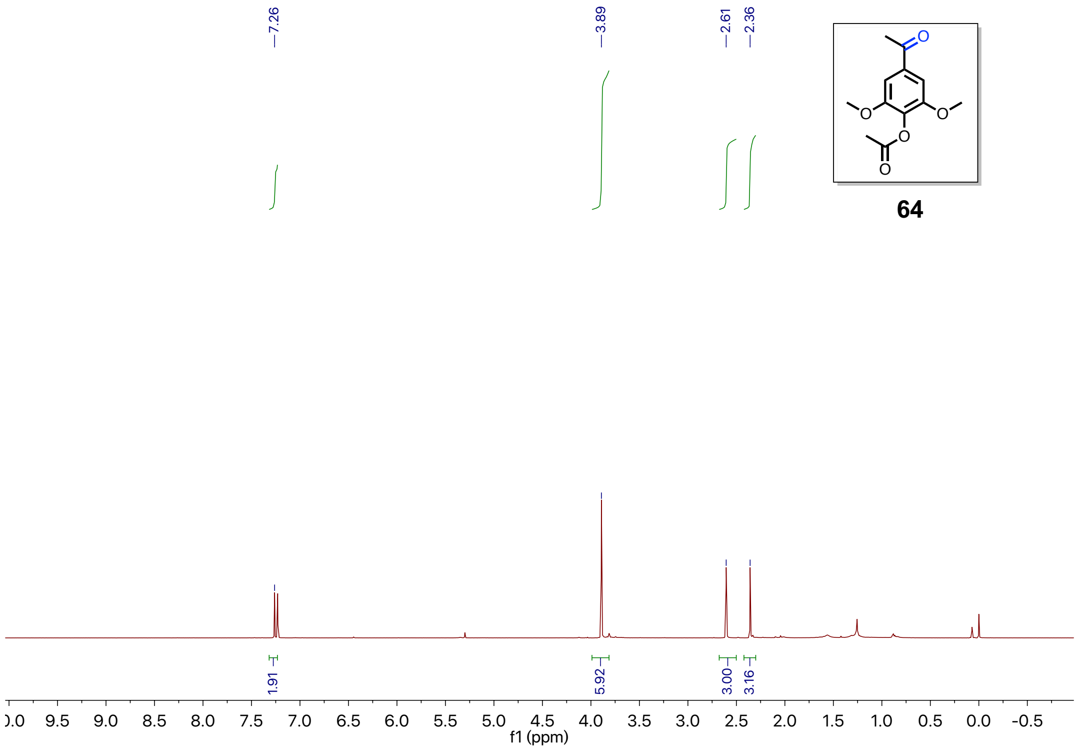

^{13}C NMR spectrum of **64** (126 MHz, $CDCl_3$)

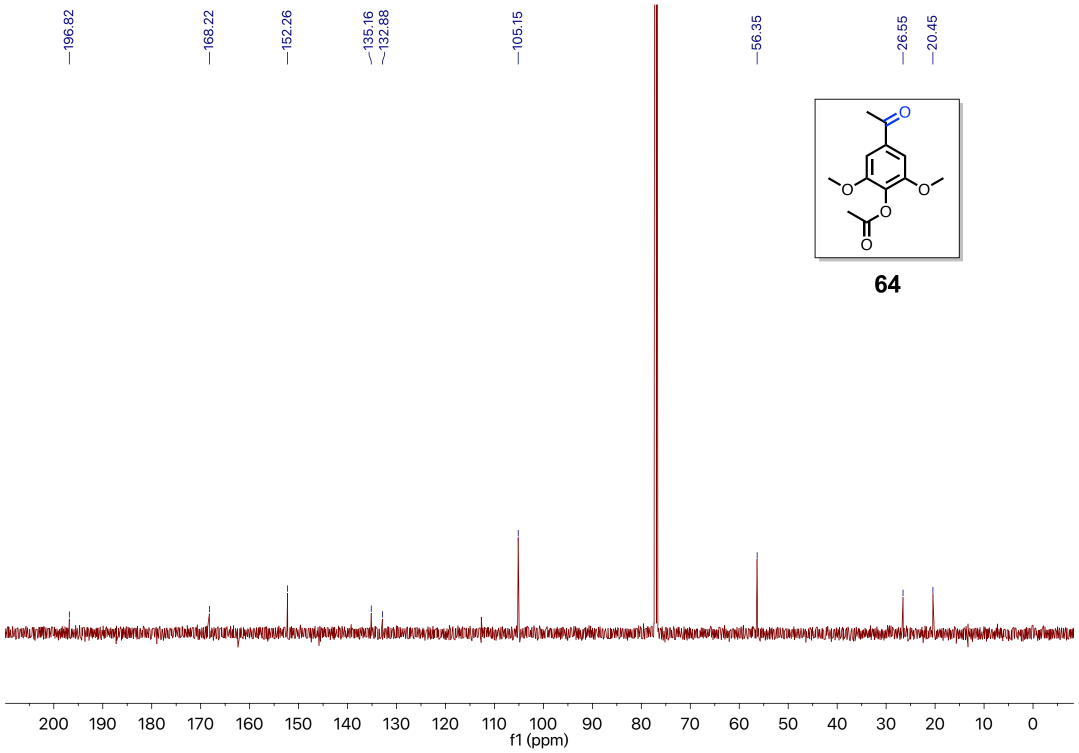

^{1}H NMR spectrum of **65** (500 MHz, $CDCl_3$)

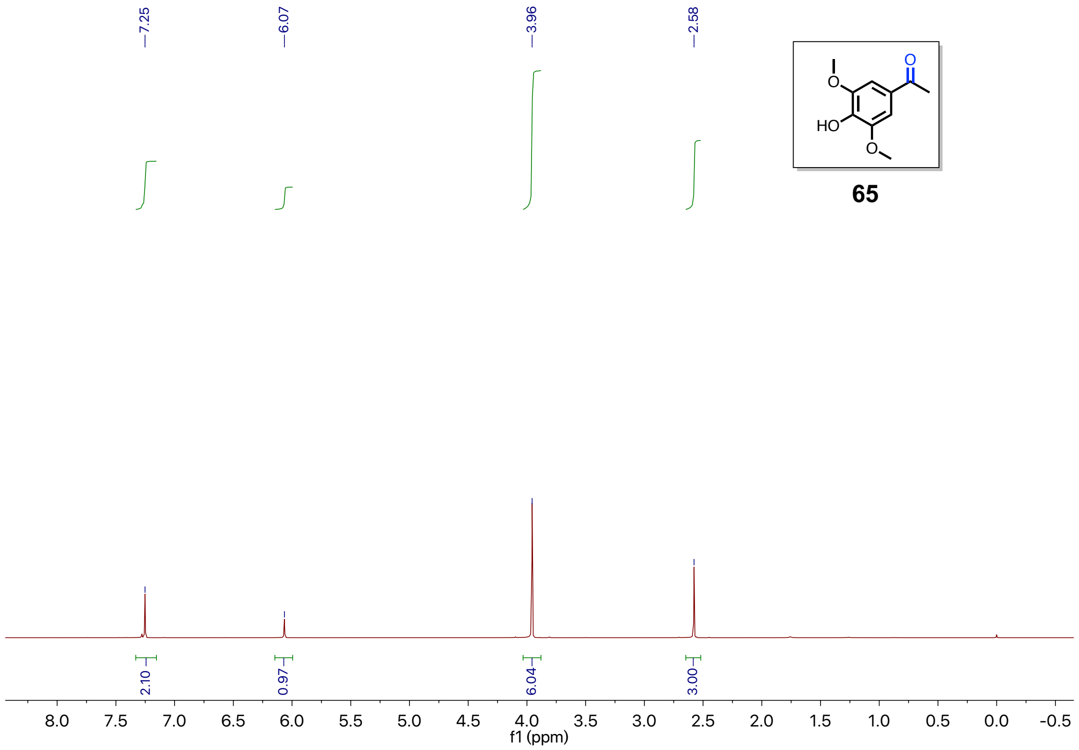

^{13}C NMR spectrum of **65** (101 MHz, $CDCl_3$)

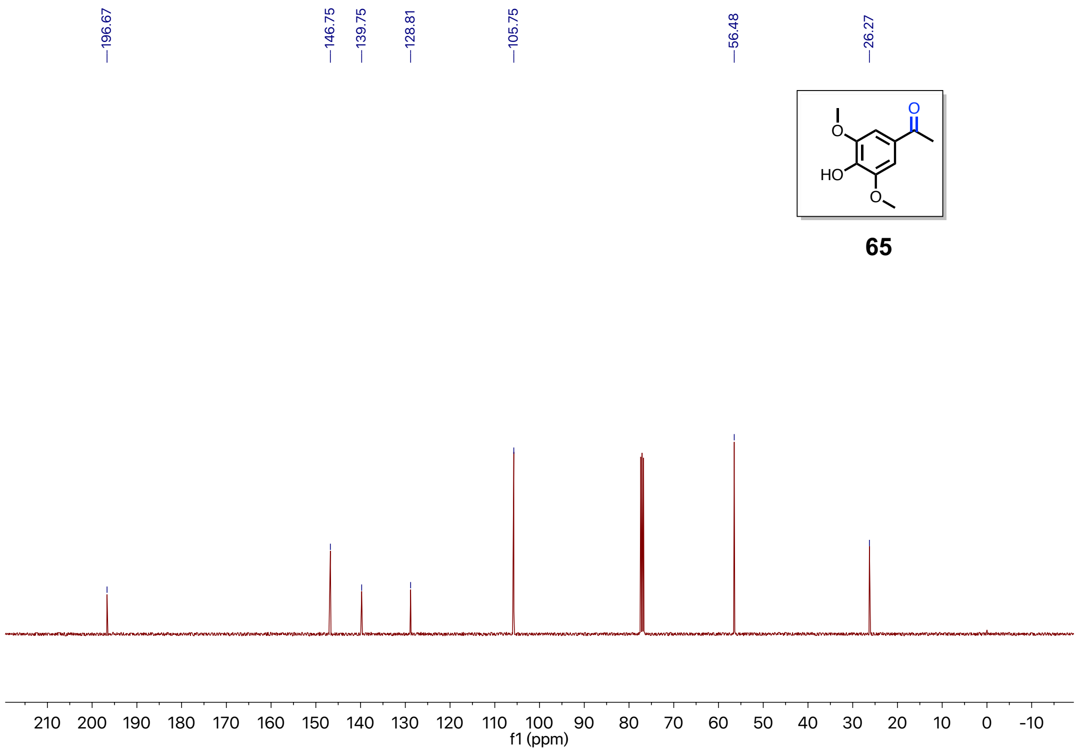

^{1}H NMR spectrum of **66** (400 MHz, $CDCl_3$)

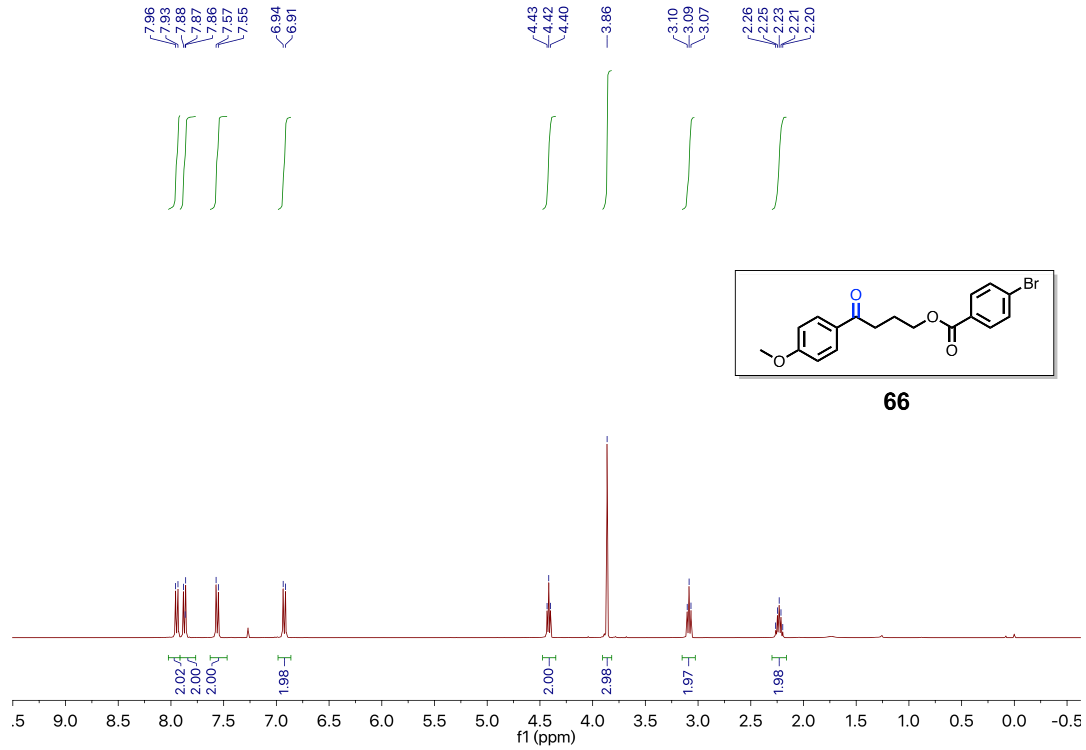

^{13}C NMR spectrum of **66** (126 MHz, $CDCl_3$)

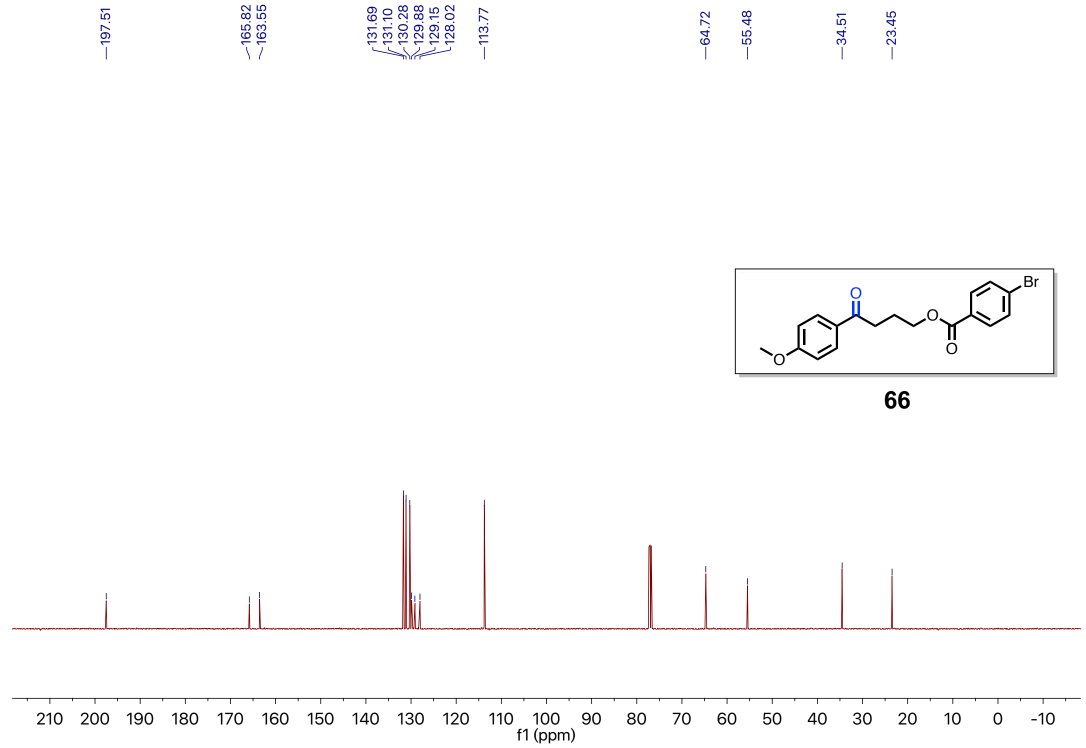

^{1}H NMR spectrum of **67** (400 MHz, $CDCl_3$)

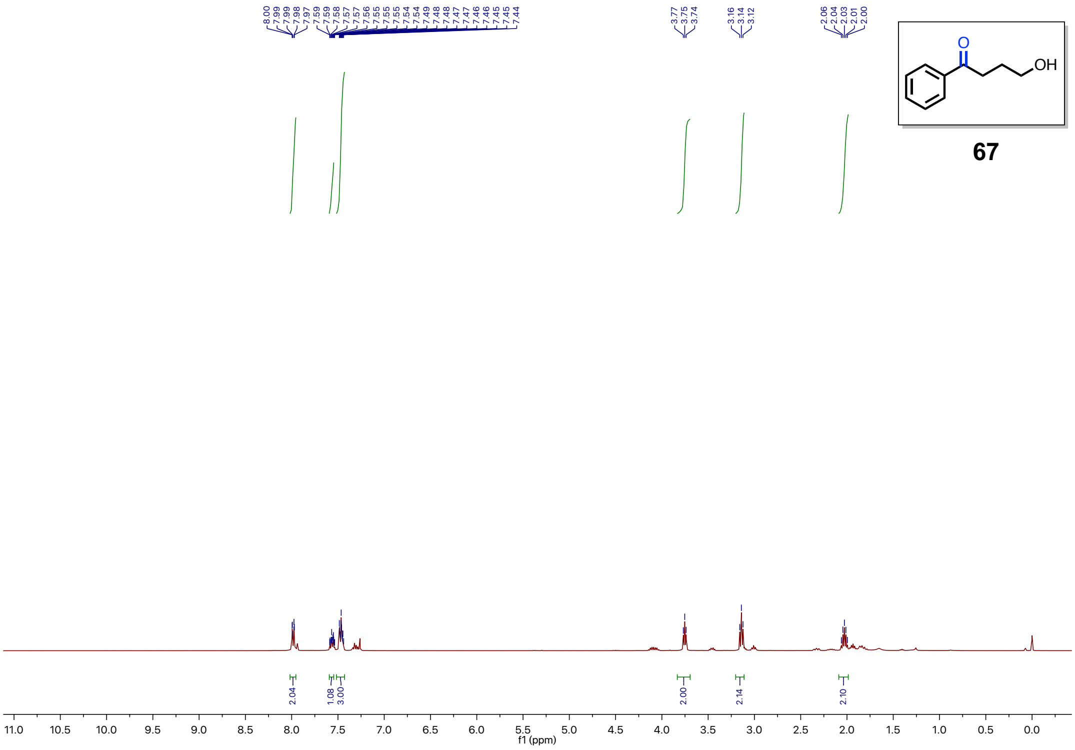

^{13}C NMR spectrum of **67** (126 MHz, $CDCl_3$)

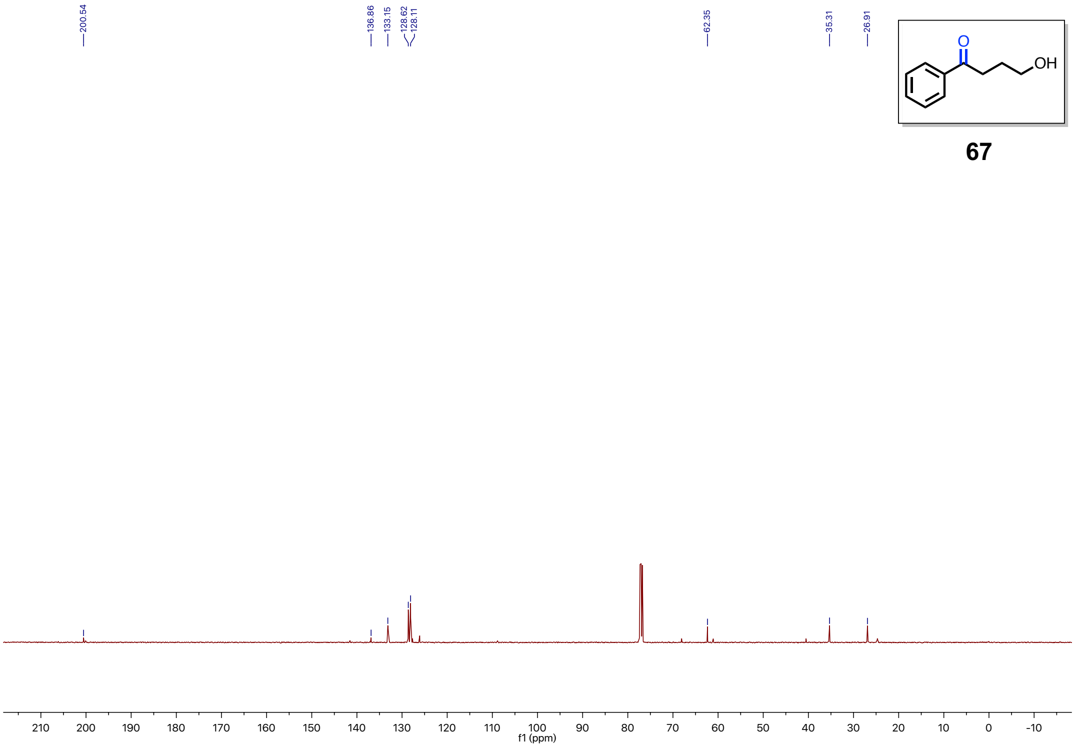

^{1}H NMR spectrum of **68** (400 MHz, $CDCl_3$)

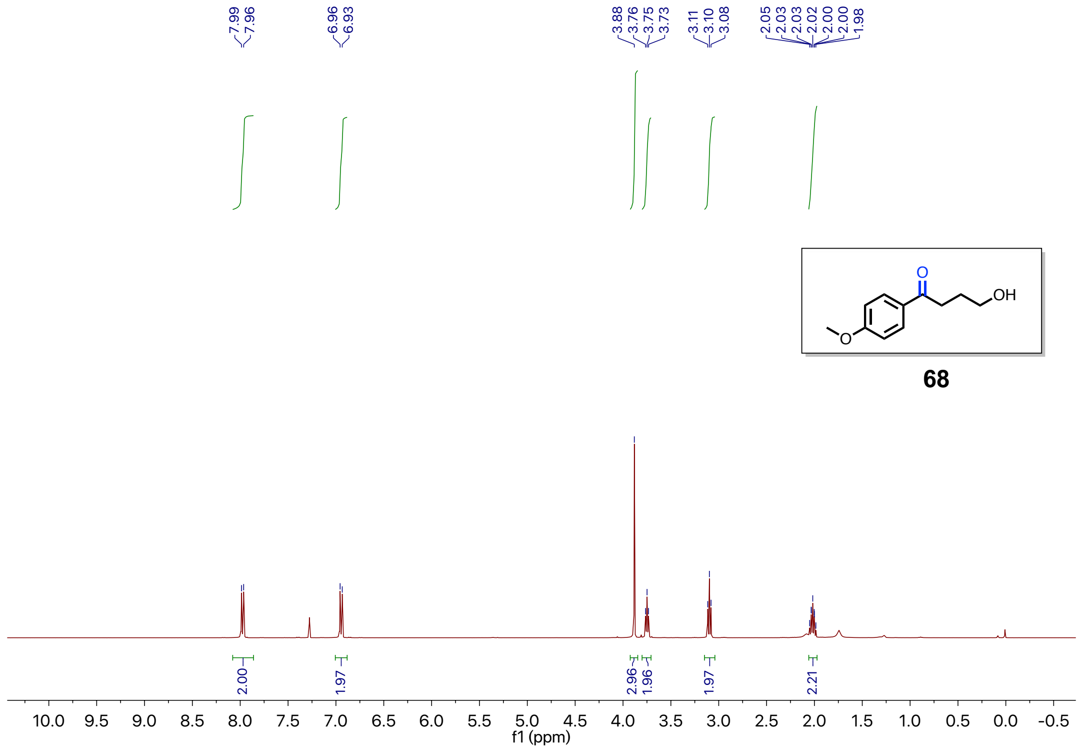

^{13}C NMR spectrum of **68** (101 MHz, $CDCl_3$)

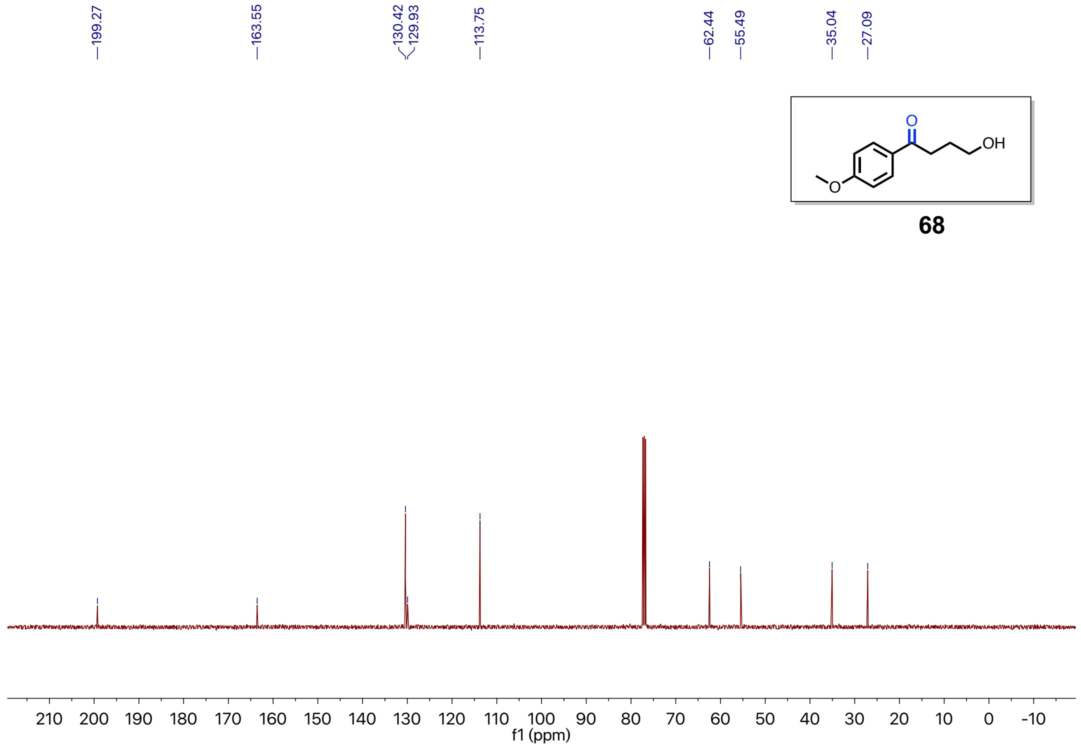

^{1}H NMR spectrum of **69** (400 MHz, $CDCl_3$)

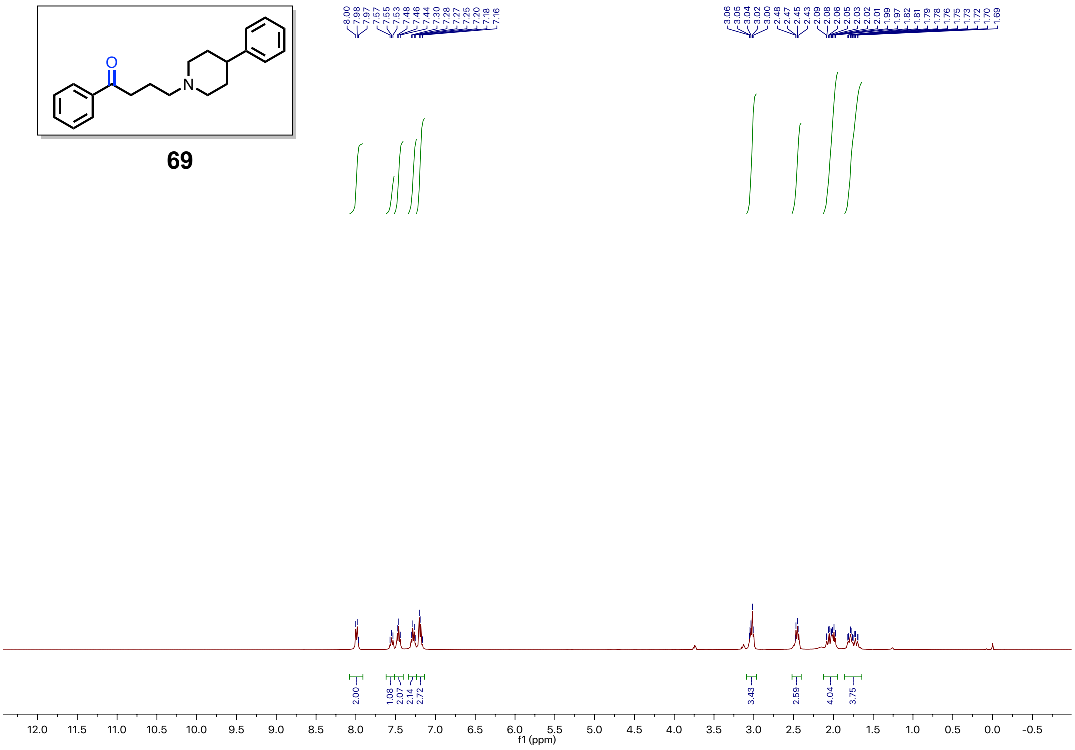

^{13}C NMR spectrum of **69** (126 MHz, $CDCl_3$)

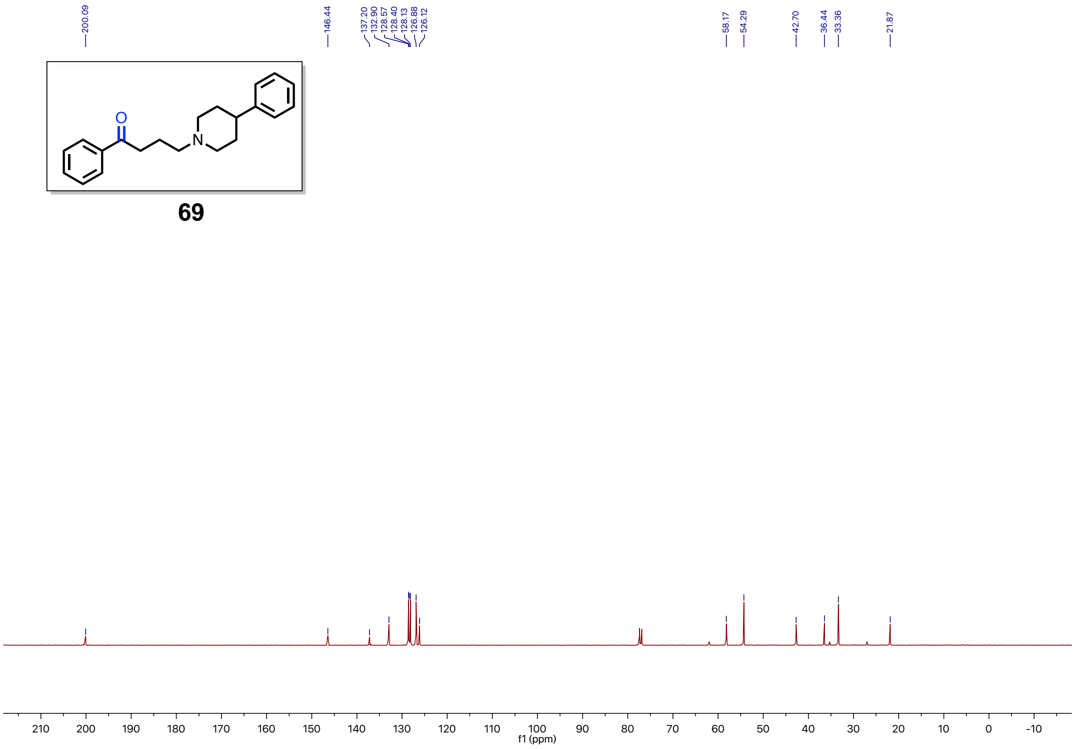

^{1}H NMR spectrum of **70** (400 MHz, $CDCl_3$)

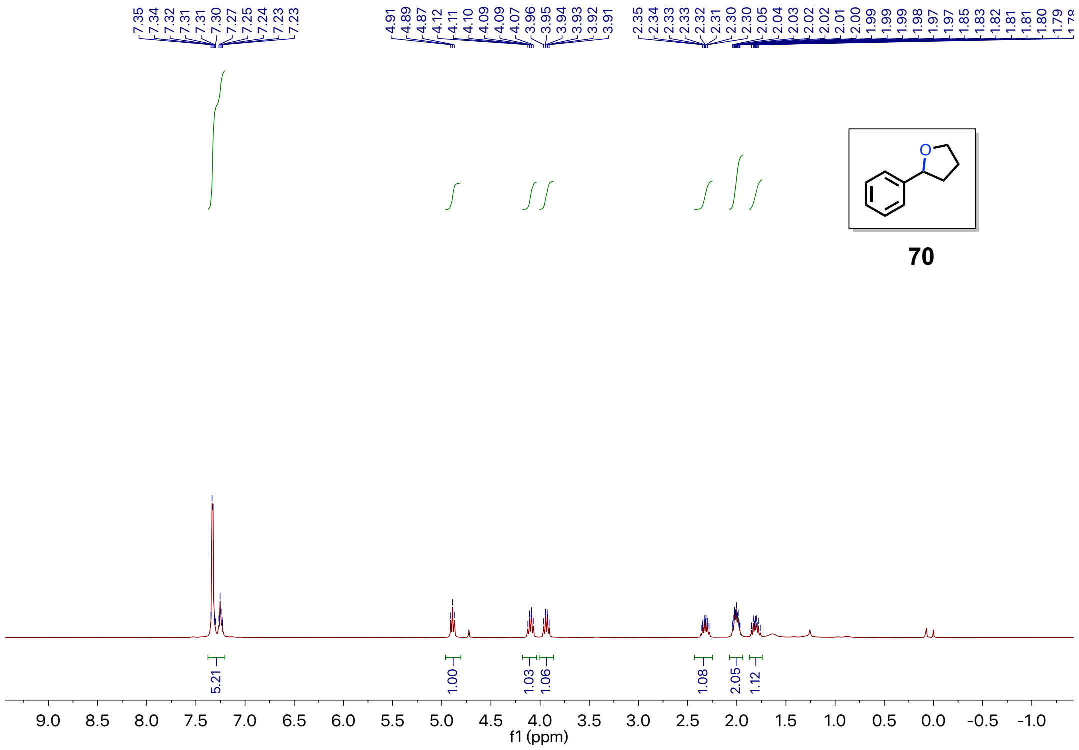

^{13}C NMR spectrum of **70** (126 MHz, $CDCl_3$)

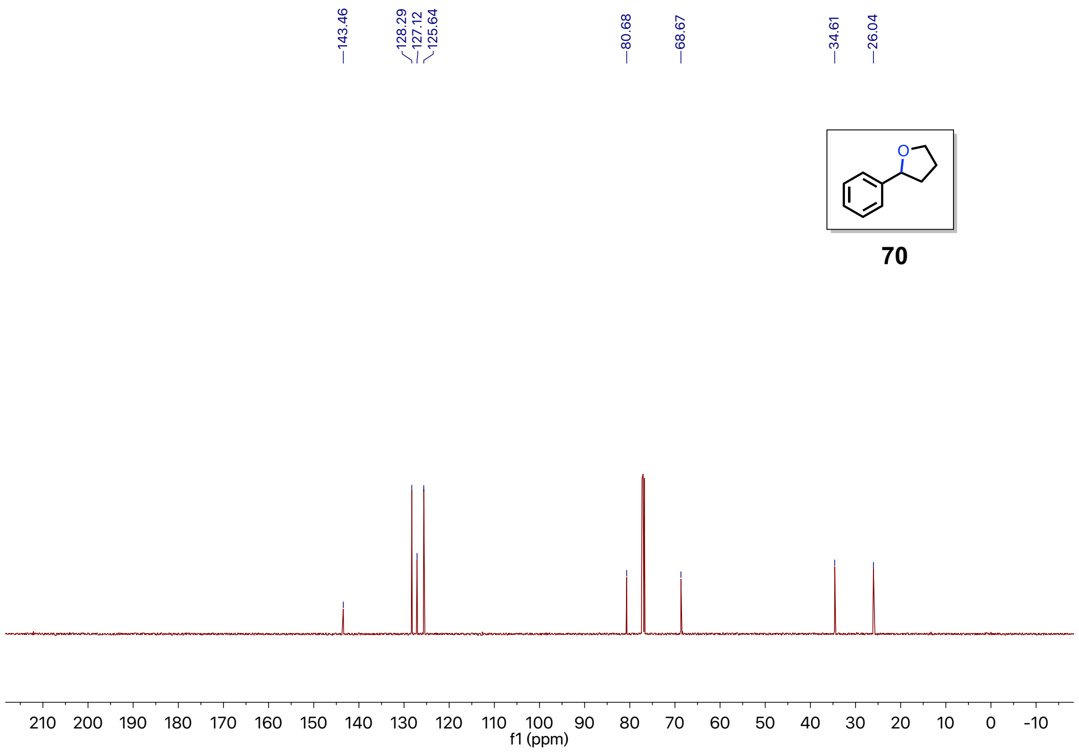

^{1}H NMR spectrum of **71** (400 MHz, $CDCl_3$)

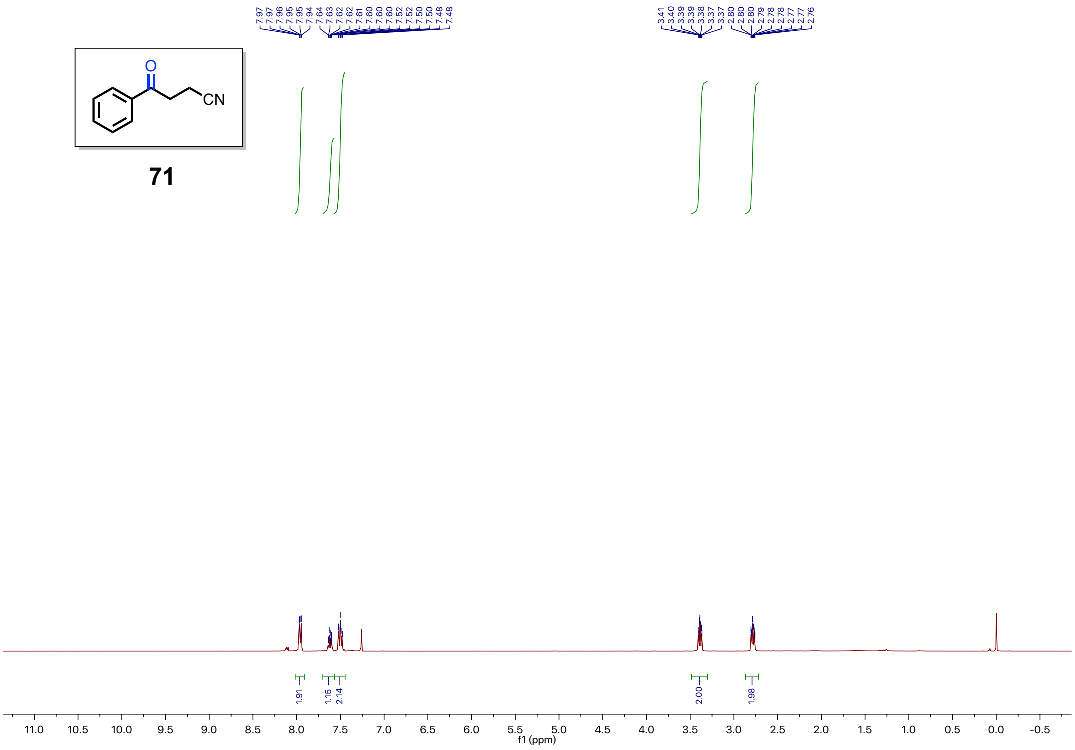

^{13}C NMR spectrum of **71** (101 MHz, $CDCl_3$)

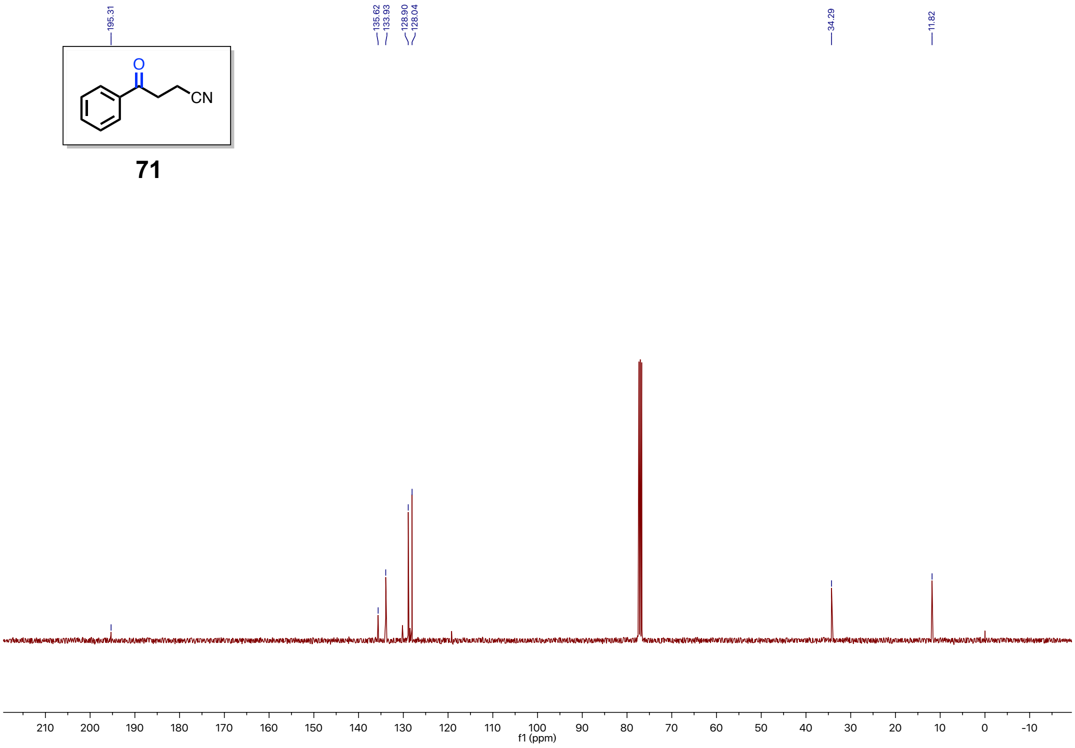

^{1}H NMR spectrum of **72** (400 MHz, $CDCl_3$)

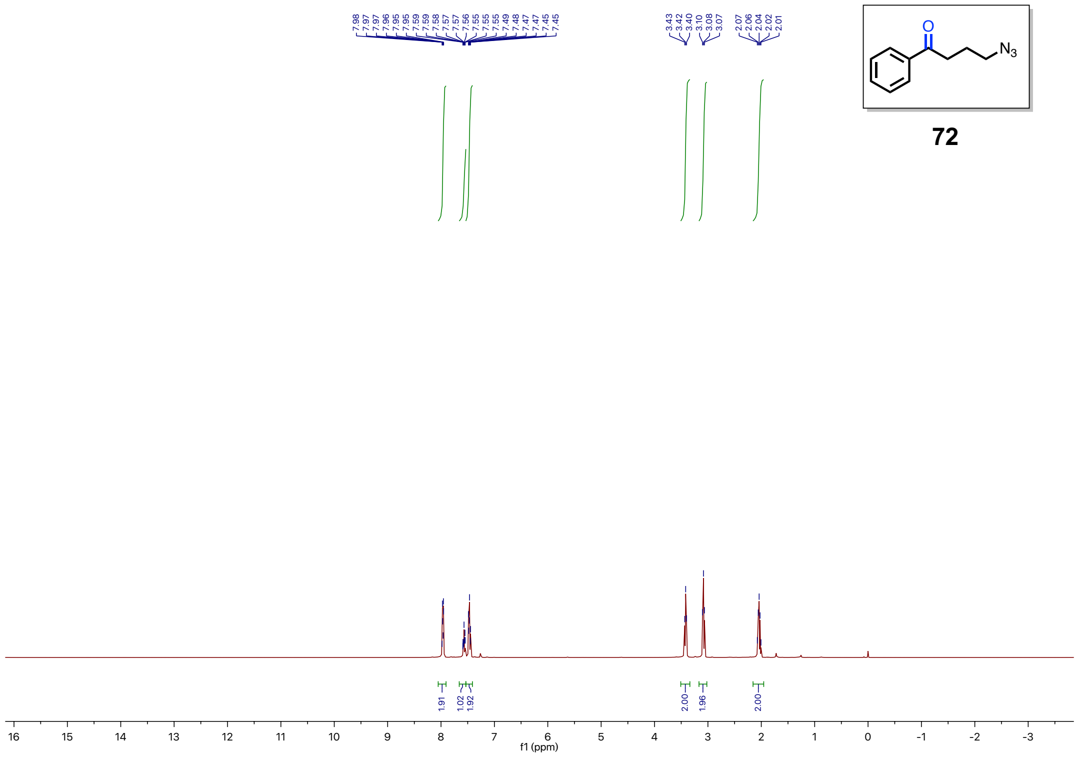

72

^{13}C NMR spectrum of **72** (126 MHz, $CDCl_3$)

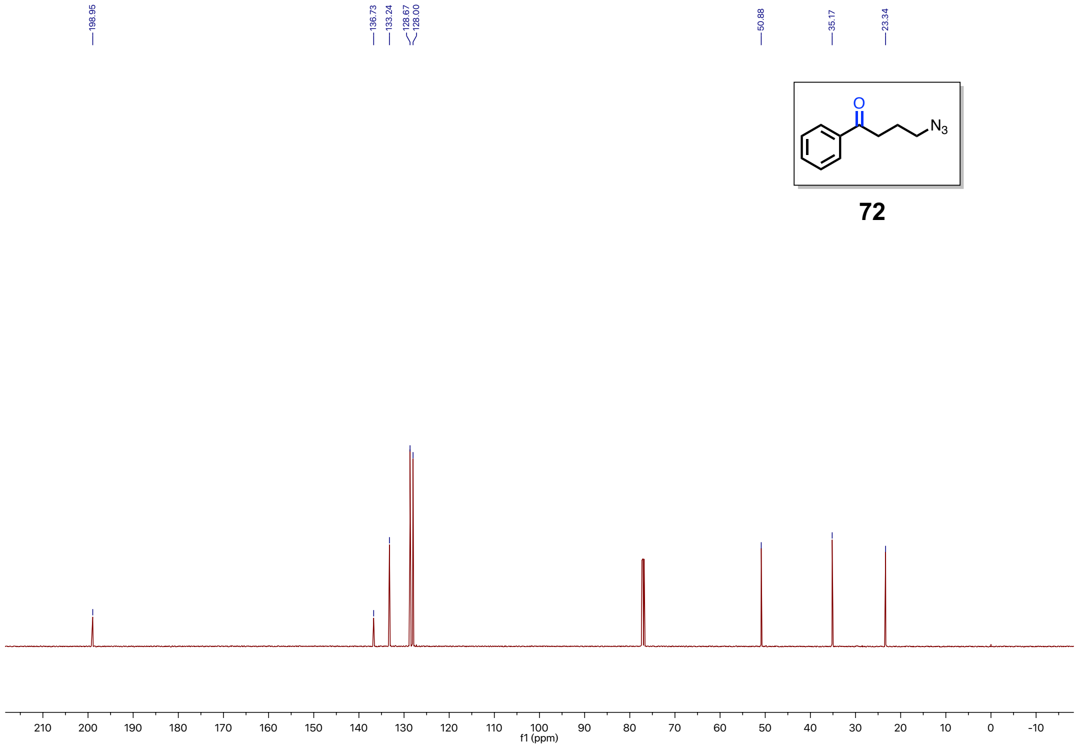

72

^{1}H NMR spectrum of **2b** (500 MHz, $CDCl_3$)

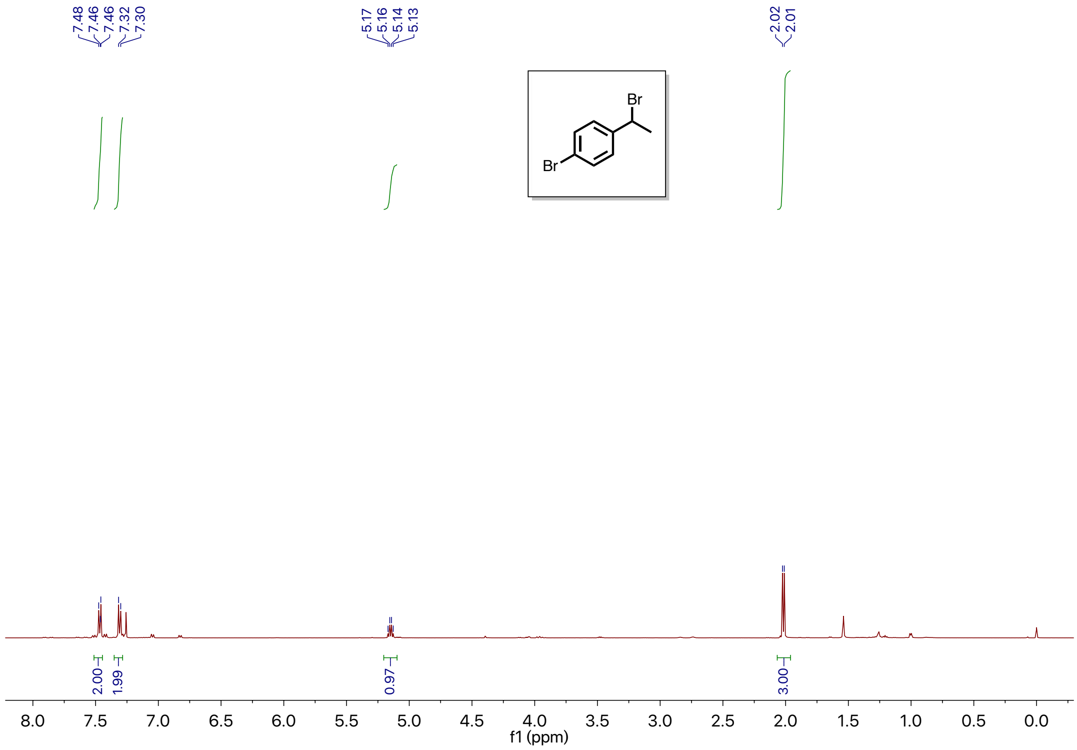

^{13}C NMR spectrum of **2b** (101 MHz, $CDCl_3$)

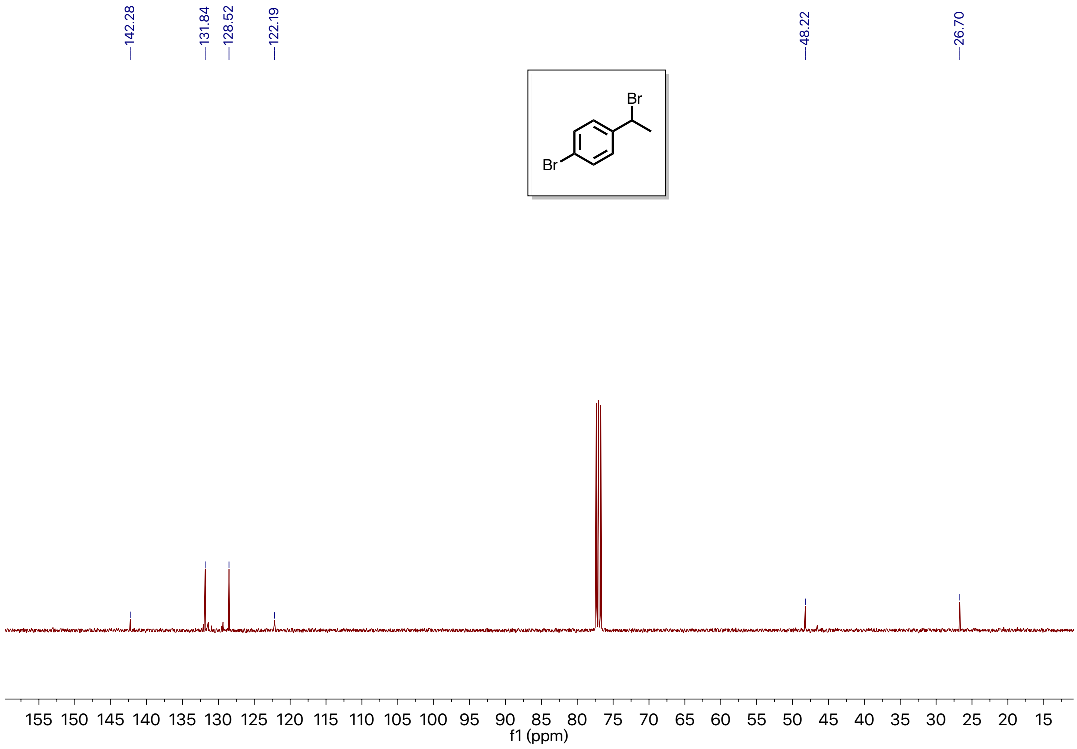

14.Reference

1. C. Y. Cai, X. L. Lai, Y. Wang, Nat Catal, 2022, 5, 943–951.
2. R. F. Nystrom, C. R. Berger, J. Am. Chem. Soc. 1958, 80, 2896-2898.
3. K. C. Agrawal. J. Med. Chem. 1967, 10, 99-101.
4. W. Zhang, P. Chen, G. Liu, J. Am. Chem. Soc. 2017, 139, 7709–7712.
5. H. Chen, W. Jin, S. Yu, Org. Lett, 2020, 22, 5910-5914.
6. L. A. Clarke, A. Ring, A. Ford, A. S. Sinha, S. E. Lawrence, A. R. Maguire, Org. Biomol. Chem. 2014, 12, 7612-7628.
7. C. E. Ayala, A. Villalpando, A. L. Nguyen, G. T. McCandless, R. Kartika, Org. Lett. 2012, 14, 3676-3679.
8. R. Kundu, Z. T. Ball, Org. Lett, 2010, 12, 2460-2463.
9. A. Horn, P. H. Dussault, RSC Adv., 2020, 10, 44408-44429.
10. A. S. Marques, M. Giardinetti, J. Marrot, V. Coeffard, X. Moreau, C. Greck, Org. Biomol. Chem. 2016, 14, 2828-2832.
11. L. Bering, K. Jeyakumar, A. P. Antonchick, Org. Lett. 2018, 20, 13, 3911–3914.
12. T. Chen, H. Long, Y. Gao, H. Xu, Angew. Chem. Int. Ed. 2023, 62, e202310138.
13. J. Zhao, H. Sun, Y. Lu, J. Li, Z. Yu, Q. Meng, X. Peng, Green Chem., 2022, 24, 8503–8511.
14. K. A. Margrey, A. Levens, D. A. Nicewicz, Angew. Chem. Int. Ed. 2017, 56, 15644
15. Q. Meng, T. E. Schirmer, A. L. Berger, K. Donabauer, B. Konig, J. Am. Chem. Soc. 2019, 141, 29, 11393–11397.
16. S. Chen, D. Yan, M. Xue, Y. Hong, Y. Yao, Q. Shen, Org. Lett. 2017, 19, 3382–3385.
17. J. Inoa, M. Patel, G. Dominici, R. Eldabagh, A. Patel, J. Lee, X. Xing, J. Org. Chem. 2020, 85, 9, 6181–6187.
18. P. Jensen, J. Arnbjerg, L. P. Tolbod, R. Toftegaard, P. R. Ogilby, J. Phys. Chem. A., 2009, 113, 9965-9973.
19. J. Zhou, M, Jia, M. Song, Z. Huang, A. Steiner, Q. An, J. Ma, Z. Guo, Q. Zhang, H. Sun, C. Robertson, J. Bacsa, J. Xiao, C. Li, Angew. Chem. Int. Ed. 2022, 61, e202205983.
20. X. Zhu, Y. Liu, C. Liu, H. Yang, H. Fu, Green Chem., 2020, 22, 4357-4363.
21. P. Huang, Y. Yu, H. Song, J. Wang, J. Wang, J. Li, B. Sun, C. Jin, Green Chem., 2024,26, 9241-9249.
22. X. Wang, C. Wang, Y. Liu, J. Xiao, Green Chem., 2016,18, 4605-4610.
23. Y. Zhang, M. Xia, M. Li, Q. Ping, Z. Yuan, X. Liu, H. Yin, S. Huang, Y. Rao, J. Org. Chem. 2021, 86, 15284–15297.
24. P. Geng, Y. Tang, G. Pan, W. Wang, J. Hu, Y. Cai, Green Chem., 2019,21, 6116-6122.
25. X. Li, F. Bai, C. Liu, X. Ma, C. Gu, B. Dai, Org. Lett. 2021, 23, 19, 7445–7449.
26. J. Wu, Y. Liu, X. Ma, P. Liu, C. Gu, B. Dai, Chin. J. Chem. 2017, 35, 1391-1395
27. P. E. Krach, A. Dewanji, T. Yuan, M. Rueping, Chem. Commun., 2020,56, 6082-6085
28. X. Zhou, H. Ding, P. Chen, L. Liu, Q. Sun, X. Wang, P. Wang, Z. Lv, M. Li, Angew. Chem. Int. Ed. 2020, 59, 4138 – 4144.
29. S. Tang, S. Wang, D. Zhang, X. Zhang, G. Yang, Y. Wang, Y. Qiu, Chin. Chem. Lett, 2024, 35, 108660.
30. A. A. Kiryanov, P. Sampsom, A. J. Seed, J. Mater. Chem., 2001,11, 3068-3077.
31. Q. Mou, T. Han, M. Liu, Org. Lett. 2024, 26, 2169-2174.

32. Q. Sun, Y. Peng, Y. Wang, X. Bao, Org. Lett. 2023, 25, 6613-6617.
33. H. Gao, J. Chen, Z. Peng, L. Feng, C. Tung, W. Wang, J. Org. Chem. 2022, 87, 10848–10857.
34. S. Zhang, X. Wang, Z. Yu, Org. Lett. 2017, 19, 3139-3142.
35. S. Zhai, S. Qiu, S. Yang, X. Gao, X. Feng, C. Yun, N. Han, Y. Niu, J. Wang, H. Zhai, Chin. Chem. Lett, 2023, 34, 107657.
36. X. Huang, J. Cai, Y. Zheng, C. Song, J. Li, Adv. Synth. Catal. 2024, 366, 201 – 206.
37. S. Maity, A. M. Szpilman, Org. Lett. 2023, 25, 1218-1222.
38. D. Hu, X. Jiang, Green Chem, 2022, 24, 124-129.
39. S. He, T. Xu, Q. Wan, K. Tang, X. Chen, D. Li, Y. Jiang, C. Zhai, C. Zhu, T. Shen, Angew. Chem. Int. Ed. 2025, 64, e202508166.
40. W. Wang, D. Xu, Q. Sun, W. Sun, Chem. Asian J. 2018, 13, 2458 – 2464.
41. Y. Li, W. Li, J. Tian, G. Huang, H. Lv, Org. Lett. 2020, 22, 14, 5353–5357.
42. S. Chun, Y. K. Chung, Org. Lett. 2017, 19, 14, 3787–3790.
43. J. Jiang, C. Chen, J. Huang, H. Liu, S. Cao, Y. Ji, Green Chem., 2014,16, 1248-1254.
44. H. Im, D. Kang, S. Choi, S. Shin, S. Hong, Org. Lett. 2018, 20, 7437–7441.
45. S. K. Murphy, V. M. Dong, J. Am. Chem. Soc. 2013, 135, 15, 5553–5556.
46. L. Tang, X. Guo, Y. Li, S. Zhang, Z. Zha, Z. Wang, Chem. Commun., 2013,49, 5213-5215.
47. Y. Sun, X. Li, M. Yang, W. Xu, J. Xie, M. Ding, Green Chem., 2020,22, 7543-7551.
48. G. Zhang, Z. Xu, B. Han, Y. Ji, S. Li, M. Zhou, M. Cao, X. Yu, L. Liu, Org. Lett. 2025, 27, 3720-3724.
49. J. Li, Z. Yu, J. Zhao, C. Ma, L. Duan, Z. Liu, H. Sun, G. Zhao, Q. Liu, Q. Meng, J. Org. Chem., 2025, 90, 1245–1255.
